# Supplementary material for: Are Homemade and Commercial Infant Foods Different? A Nutritional Profile and Food Variety Analysis in Spain
Source: Nutrients. 2021 Feb 27;13(3):777. doi: 10.3390/nu13030777 (PMC7997232; doi:10.3390/nu13030777)

FICHA INGREDIENTES/PESOS "PLATO HECHO EN CASA (HC)" **ID: BCN 01**

DÍA DEL CONSUMO: *9 / 10 / 2014* HORA DEL CONSUMO: *19:30*

LA ELABOARACIÓN DEL PLATO SE HACE EL MISMO DÍA DEL CONSUMO [X] Sí [ ] No

Si la fecha de elaboración del plato NO es el mismo día del consumo NO se adjuntará foto y el peso de los ingredientes puede ser aproximado. Indique la fecha de elaboración:

TIPO DE PLATO: *PURÉ DE VERDURAS CON PESCADO*

	Tipo de ingrediente	Nº	Peso (en gramos)
Ingrediente 1	*Patatas*	*4*	*600 gr*
Ingrediente 2	*Zanahoria*	*1*	*15 gr*
Ingrediente 3	*Calabaza*	*¼*	*150 gr*
Ingrediente 4	*Cebolla*	*1*	*220 gr*
Ingrediente 5	*Judías verdes*	*8*	*695 gr*
Ingrediente 6	*Aceite de oliva*	*1*	*205 gr*
Ingrediente 7	*Pescado (merluza)*	*1*	*218 gr*
Ingrediente 8	*Agua*	*1*	*350 gr*
Ingrediente 9			
Ingrediente 10			

Proceso culinario	Nº	Peso (en gramos)	Observaciones
1. Olla VACÍA		*1158 gr*	
2. Olla con COMIDA COCINADA		*2653 gr*	
**COMIDA COCINADA (2-1)**		*1495 gr*	
3. Fiambrera VACÍA	*1*	*35 gr*	*Relleno 5 fiambreras*
4. Fiambrera con COMIDA COCINADA		*318 gr*	
**COMIDA ALMACENADA (4-3)**		*283 gr*	
5. Plato VACÍO		*305 gr*	
6. Plato con COMIDA COCINADA		*617 gr*	
**COMIDA QUE SIRVE A SU HIJO (6-5)**		*312 gr*	
7. Plato COMIDA QUE SU HIJO NO HA COMIDO		*400 gr*	
**COMIDA QUE SU HIJO HA COMIDO (6-7)**		*217 gr*	

MÉTODO DE ELABORACIÓN:

Explicar detalladamente el proceso de elaboración del plato

FICHA INGREDIENTES/PESOS “PLATO HECHO EN CASA (HC)” **ID: BCN 01**

DÍA DEL CONSUMO: 9 / 10 / 2014 **HORA DEL CONSUMO:** 19:30

MÉTODO DE ELABORACIÓN:
Explicar detalladamente el proceso de elaboración del plato:

Pelar y cortar todas las verduras.
Añadir el agua y hervir las verduras durante 25 minutos.
Lava y trocea el pescado.
Hervir el pescado junto a las verduras durante 10 minutos.
Triturar todo.
Añadir el aceite al final.

Fotos:

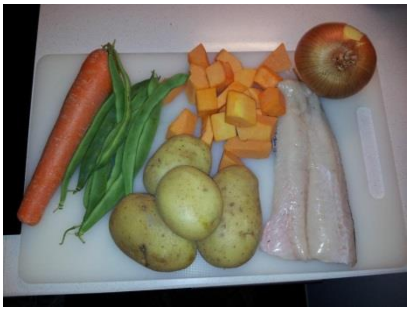

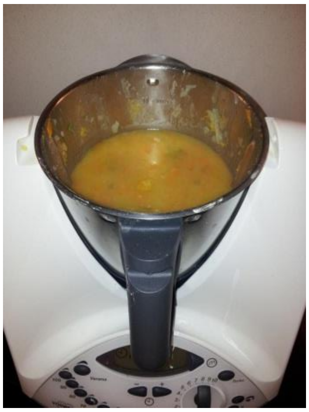

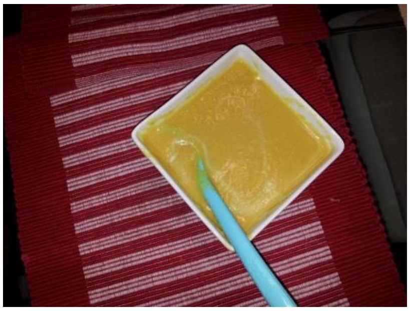

FICHA INGREDIENTES/PESOS “PLATO HECHO EN CASA (HC)” **ID: BCN 01**

DÍA DEL CONSUMO: *12 / 10 / 2014* HORA DEL CONSUMO: *12:30*

LA ELABOARACIÓN DEL PLATO SE HACE EL MISMO DÍA DEL CONSUMO [X] Sí [ ] No

Si la fecha de elaboración del plato NO es el mismo día del consumo NO se adjuntará foto y el peso de los ingredientes puede ser aproximado. Indique la fecha de elaboración:

TIPO DE PLATO: *PURÉ DE VERDURAS CON POLLO*

	Tipo de ingrediente	Nº	Peso (en gramos)
Ingrediente 1	*Patatas*	*3*	*907 gr*
Ingrediente 2	*Calabacín*	*1*	*340 gr*
Ingrediente 3	*Zanahoria*	*1*	*153 gr*
Ingrediente 4	*Cebolla*	*1*	*218 gr*
Ingrediente 5	*Judías verdes*	*7*	*62 gr*
Ingrediente 6	*Pollo*	*1*	*149 gr*
Ingrediente 7	*Agua*	*1*	*350 gr*
Ingrediente 8	*Aceite*	*1*	*20 gr*
Ingrediente 9			
Ingrediente 10			

Proceso culinario	Nº	Peso (en gramos)	Observaciones
1. Olla VACÍA		*1158 gr*	
2. Olla con COMIDA COCINADA		*2471 gr*	
**COMIDA COCINADA (2-1)**		*1313 gr*	
3. Fiambrera VACÍA	*1*	*35 gr*	*Relleno 4 fiambreras*
4. Fiambrera con COMIDA COCINADA		*281 gr*	
**COMIDA ALMACENADA (4-3)**		*246 gr*	
5. Plato VACÍO		*305 gr*	
6. Plato con COMIDA COCINADA		*606 gr*	
**COMIDA QUE SIRVE A SU HIJO (6-5)**		*301 gr*	
7. Plato COMIDA QUE SU HIJO NO HA COMIDO		*315 gr*	
**COMIDA QUE SU HIJO HA COMIDO (6-7)**		*291 gr*	

MÉTODO DE ELABORACIÓN:
Explicar detalladamente el proceso de elaboración del plato

FICHA INGREDIENTES/PESOS "PLATO HECHO EN CASA (HC)"

ID: BCN 01

DÍA DEL CONSUMO: 12 / 10 / 2014 **HORA DEL CONSUMO:** 12:30

MÉTODO DE ELABORACIÓN:

Explicar detalladamente el proceso de elaboración del plato:

Pelar y cortar todas las verduras.
Añadir el agua y hervir las verduras durante 30 minutos.
Lavar y trocear el pollo.
Hervir el pollo junto a las verduras durante 15 minutos.
Triturar todo.
Añadir el aceite al final.

Fotos:

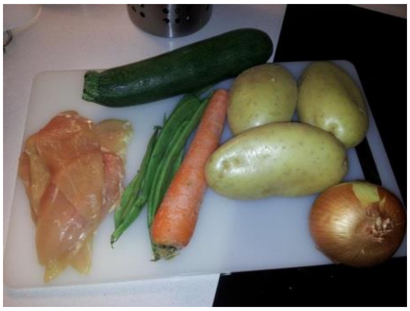

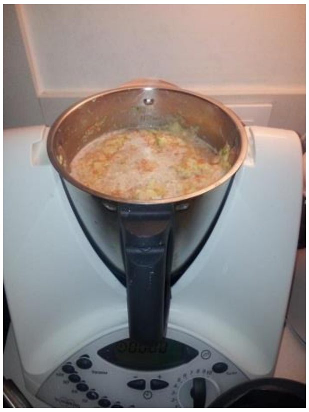

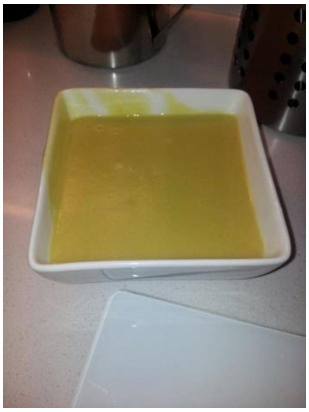

FICHA INGREDIENTES/PESOS "PLATO HECHO EN CASA (HC)" **ID: BCN 01**

DÍA DEL CONSUMO: *12 / 10 / 2014* HORA DEL CONSUMO: *16:00*

LA ELABOARACIÓN DEL PLATO SE HACE EL MISMO DÍA DEL CONSUMO [X] Sí [ ] No

Si la fecha de elaboración del plato NO es el mismo día del consumo NO se adjuntará foto y el peso de los ingredientes puede ser aproximado. Indique la fecha de elaboración:

TIPO DE PLATO: *PAPILLA DE FRUTAS*

	Tipo de ingrediente	Nº	Peso (en gramos)
Ingrediente 1	*Plátano*		*130 gr*
Ingrediente 2	*Pera*		*152 gr*
Ingrediente 3	*Naranja*		*203 gr*
Ingrediente 4	*Ciruela*		*67 gr*
Ingrediente 5			
Ingrediente 6			
Ingrediente 7			
Ingrediente 8			
Ingrediente 9			
Ingrediente 10			

Proceso culinario	Nº	Peso (en gramos)	Observaciones
1. Olla VACÍA		*145 gr*	
2. Olla con COMIDA COCINADA		*547 gr*	
**COMIDA COCINADA (2-1)**		*402 gr*	
3. Fiambrera VACÍA			
4. Fiambrera con COMIDA COCINADA			
**COMIDA ALMACENADA (4-3)**			
5. Plato VACÍO		*307 gr*	
6. Plato con COMIDA COCINADA		*515 gr*	
**COMIDA QUE SIRVE A SU HIJO (6-5)**		*208 gr*	
7. Plato COMIDA QUE SU HIJO NO HA COMIDO		*312 gr*	
**COMIDA QUE SU HIJO HA COMIDO (6-7)**		*203 gr*	

MÉTODO DE ELABORACIÓN:
Explicar detalladamente el proceso de elaboración del plato

FICHA INGREDIENTES/PESOS "PLATO HECHO EN CASA (HC)"

ID: BCN 01

DÍA DEL CONSUMO: 12 / 10 / 2014 **HORA DEL CONSUMO:** 16:00

MÉTODO DE ELABORACIÓN:

Explicar detalladamente el proceso de elaboración del plato:

Pelar y trocear las frutas.
Triturar y servir.

Fotos:

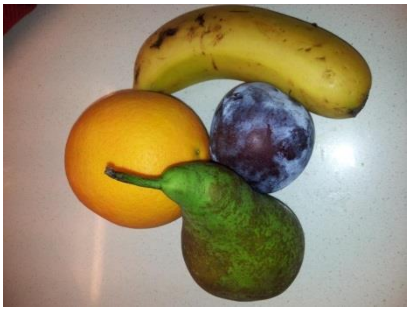

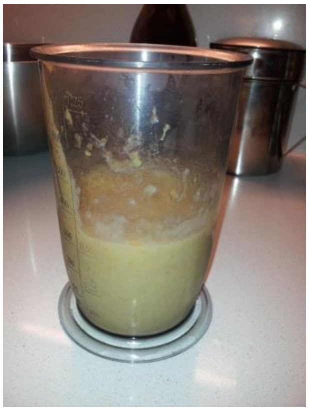

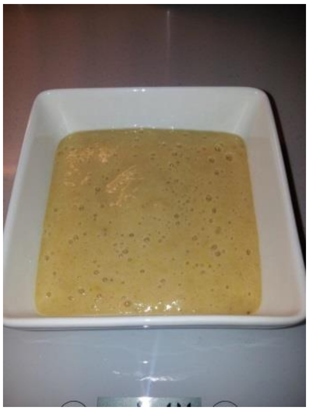

FICHA INGREDIENTES/PESOS “PLATO HECHO EN CASA (HC)” **ID: BCN 01**

DÍA DEL CONSUMO: *12 / 10 / 2014* HORA DEL CONSUMO: *20:00*

LA ELABOARACIÓN DEL PLATO SE HACE EL MISMO DÍA DEL CONSUMO ☐ Sí ☒ No

Si la fecha de elaboración del plato NO es el mismo día del consumo NO se adjuntará foto y el peso de los ingredientes puede ser aproximado. Indique la fecha de elaboración: 9/10/14

TIPO DE PLATO: *PURÉ DE VERDURAS CON PESCADO*

	Tipo de ingrediente	Nº	Peso (en gramos)
Ingrediente 1	*Patatas*	*4*	*600 gr*
Ingrediente 2	*Zanahorias*	*1*	*158 gr*
Ingrediente 3	*Calabaza*	*¼*	*150 gr*
Ingrediente 4	*Cebolla*	*1*	*220 gr*
Ingrediente 5	*Judías verdes*	*6-7*	*70 gr*
Ingrediente 6	*Aceite de oliva*	*1*	*20 gr*
Ingrediente 7	*Pescado (merluza)*		*218 gr*
Ingrediente 8	*Agua*	*1*	*350 gr*
Ingrediente 9			
Ingrediente 10			

Proceso culinario	Nº	Peso (en gramos)	Observaciones
1. Olla VACÍA			
2. Olla con COMIDA COCINADA			
**COMIDA COCINADA (2-1)**			
3. Fiambrera VACÍA			
4. Fiambrera con COMIDA COCINADA			
**COMIDA ALMACENADA (4-3)**			
5. Plato VACÍO		*305 gr*	
6. Plato con COMIDA COCINADA		*588 gr*	
**COMIDA QUE SIRVE A SU HIJO (6-5)**		*283 gr*	
7. Plato COMIDA QUE SU HIJO NO HA COMIDO		*355 gr*	
**COMIDA QUE SU HIJO HA COMIDO (6-7)**		*233 gr*	

MÉTODO DE ELABORACIÓN:
Explicar detalladamente el proceso de elaboración del plato

FICHA INGREDIENTES/PESOS "PLATO HECHO EN CASA (HC)"

ID: BCN 01

DÍA DEL CONSUMO: 12 / 10 / 2014 **HORA DEL CONSUMO:** 20:00

MÉTODO DE ELABORACIÓN:

Explicar detalladamente el proceso de elaboración del plato:

Pelar, lavar y cortar todas las verduras.
Añadir el agua y hervir 30 minutos.
Lavar y trocear el pescado.
Hervir junto con las verduras 10 minutos.
Triturar todo.
Añadir al final el aceite.

Fotos:

ALIMENTO PREPARADO ANTERIORMENTE Y SACADO DEL CONGELADOR

FICHA INGREDIENTES/PESOS “PLATO HECHO EN CASA (HC)” **ID: BCN 01**

DÍA DEL CONSUMO: *13 / 10 / 2014* HORA DEL CONSUMO: *19:30*

LA ELABOARACIÓN DEL PLATO SE HACE EL MISMO DÍA DEL CONSUMO [X] Sí [ ] No

Si la fecha de elaboración del plato NO es el mismo día del consumo NO se adjuntará foto y el peso de los ingredientes puede ser aproximado. Indique la fecha de elaboración: 9/10/14

TIPO DE PLATO: *PURÉ DE VERDURAS CON PESCADO*

	Tipo de ingrediente	Nº	Peso (en gramos)
Ingrediente 1	*Patatas*	*4*	*712 gr*
Ingrediente 2	*Calabacín*	*1*	*361 gr*
Ingrediente 3	*Calabaza*	*¼*	*198 gr*
Ingrediente 4	*Cebolla*	*1*	*290 gr*
Ingrediente 5	*Judías verdes*	*8*	*70 gr*
Ingrediente 6	*Pescado (lubina)*	*1*	*213 gr*
Ingrediente 7			
Ingrediente 8			
Ingrediente 9			
Ingrediente 10			

Proceso culinario	Nº	Peso (en gramos)	Observaciones
1. Olla VACÍA		*1158 gr*	
2. Olla con COMIDA COCINADA		*2603 gr*	
**COMIDA COCINADA (2-1)**		*1445 gr*	
3. Fiambrera VACÍA		*13 gr*	
4. Fiambrera con COMIDA COCINADA		*243 gr*	
**COMIDA ALMACENADA (4-3)**		*230 gr*	
5. Plato VACÍO		*314 gr*	
6. Plato con COMIDA COCINADA		*574 gr*	
**COMIDA QUE SIRVE A SU HIJO (6-5)**		*260 gr*	
7. Plato COMIDA QUE SU HIJO NO HA COMIDO		*331 gr*	
**COMIDA QUE SU HIJO HA COMIDO (6-7)**		*243 gr*	

MÉTODO DE ELABORACIÓN:
Explicar detalladamente el proceso de elaboración del plato

FICHA INGREDIENTES/PESOS "PLATO HECHO EN CASA (HC)"

ID: BCN 01

DÍA DEL CONSUMO: 13 / 10 / 2014 **HORA DEL CONSUMO:** 19:30

MÉTODO DE ELABORACIÓN:

Explicar detalladamente el proceso de elaboración del plato:

Pelar, lavar y cortar todas las verduras.
Añadir el agua y hervir las verduras durante 30 minutos.
Lavar y trocear el pescado.
Hervir el pescado junto a las verduras durante 10 minutos.
Triturar todo.
Añadir el aceite al final.

Fotos:

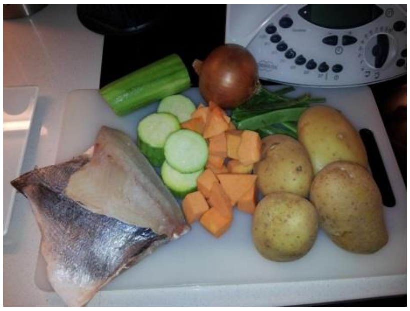

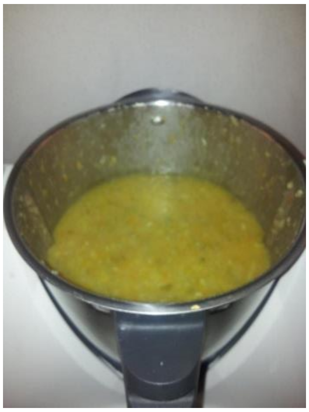

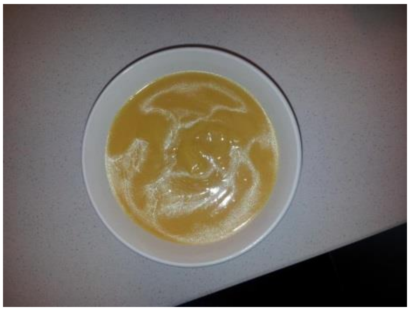

FICHA INGREDIENTES/PESOS “PLATO HECHO EN CASA (HC)” **ID: BCN 02**

DÍA DEL CONSUMO: *8 / 10 / 2014* HORA DEL CONSUMO: *13:30*

LA ELABOARACIÓN DEL PLATO SE HACE EL MISMO DÍA DEL CONSUMO [X] Sí [ ] No

Si la fecha de elaboración del plato NO es el mismo día del consumo NO se adjuntará foto y el peso de los ingredientes puede ser aproximado. Indique la fecha de elaboración: 8-10-14 Miércoles

TIPO DE PLATO: *PURÉ DE VERDURAS CON PESCADO*

	Tipo de ingrediente	Nº	Peso (en gramos)
Ingrediente 1	*Zanahoria*	*1*	*103 gr*
Ingrediente 2	*Judía verde*	*12*	*160 gr*
Ingrediente 3	*Puerro*	*½*	*63 gr*
Ingrediente 4	*Patata*	*1*	*125 gr*
Ingrediente 5	*Merluza*	*1 filete*	*97 gr*
Ingrediente 6	*Aceite*	*1*	*1 cucharada de café*
Ingrediente 7	*Agua*	*¾*	
Ingrediente 8			
Ingrediente 9			
Ingrediente 10			

Proceso culinario	Nº	Peso (en gramos)	Observaciones
1. Olla VACÍA		*934 gr*	
2. Olla con COMIDA COCINADA		*2005 gr*	
**COMIDA COCINADA (2-1)**		*1071 gr*	
3. Fiambrera VACÍA	*1*	*147 gr*	
4. Fiambrera con COMIDA COCINADA	*1*	*379 gr*	
**COMIDA ALMACENADA (4-3)**		*150 gr*	
5. Plato VACÍO		*89 gr*	
6. Plato con COMIDA COCINADA		*289 gr*	
**COMIDA QUE SIRVE A SU HIJO (6-5)**		*200 gr*	
7. Plato COMIDA QUE SU HIJO NO HA COMIDO		*89 gr*	
**COMIDA QUE SU HIJO HA COMIDO (6-7)**		*200 gr*	

MÉTODO DE ELABORACIÓN:
Explicar detalladamente el proceso de elaboración del plato

FICHA INGREDIENTES/PESOS "PLATO HECHO EN CASA (HC)"

ID: BCN 02

DÍA DEL CONSUMO: 8 / 10 / 2014 **HORA DEL CONSUMO:** 13:30

MÉTODO DE ELABORACIÓN:

Explicar detalladamente el proceso de elaboración del plato:

Pelar y cortar todas las verduras.
Descongelar el pescado.
Poner a hervir el agua.
Hervir las verduras durante 20 minutos.
Añadir la merluza, hervir 5 minutos más.
Triturarlo todo añadiendo el agua de la olla necesaria y añadir el aceite.

Fotos:

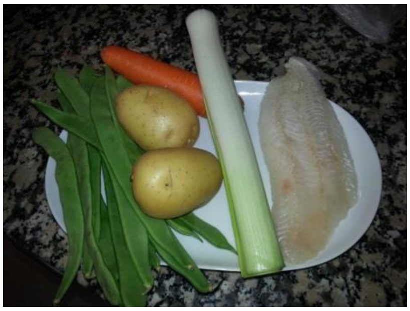

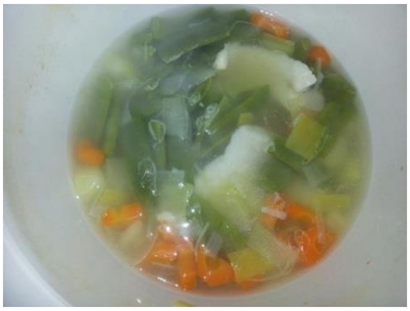

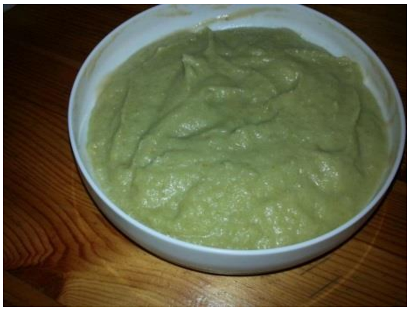

FICHA INGREDIENTES/PESOS "PLATO HECHO EN CASA (HC)" **ID: BCN 02**

DÍA DEL CONSUMO: *10 / 10 / 2014* HORA DEL CONSUMO: *13:30*

LA ELABOARACIÓN DEL PLATO SE HACE EL MISMO DÍA DEL CONSUMO [X] Sí [ ] No

Si la fecha de elaboración del plato NO es el mismo día del consumo NO se adjuntará foto y el peso de los ingredientes puede ser aproximado. Indique la fecha de elaboración: 10/10/14 Viernes

TIPO DE PLATO: *PURÉ DE VERDURAS CON CARNE DE CABALLO*

	Tipo de ingrediente	Nº	Peso (en gramos)
Ingrediente 1	*Calabacín*	*1*	*202 gr*
Ingrediente 2	*Puerro*	*½*	*105 gr*
Ingrediente 3	*Zanahoria*	*1*	*122 gr*
Ingrediente 4	*Judía verde*	*8*	*152 gr*
Ingrediente 5	*Carne de caballo*	*1 filete*	*48 gr*
Ingrediente 6			
Ingrediente 7			
Ingrediente 8			
Ingrediente 9			
Ingrediente 10			

Proceso culinario	Nº	Peso (en gramos)	Observaciones
1. Olla VACÍA		*934 gr*	
2. Olla con COMIDA COCINADA		*2117 gr*	
**COMIDA COCINADA (2-1)**		*1183 gr*	
3. Fiambrera VACÍA		*148 gr*	*Relleno 3 fiambreras*
4. Fiambrera con COMIDA COCINADA		*348 gr*	
**COMIDA ALMACENADA (4-3)**		*200 gr*	
5. Plato VACÍO		*90 gr*	
6. Plato con COMIDA COCINADA		*290 gr*	
**COMIDA QUE SIRVE A SU HIJO (6-5)**		*200 gr*	
7. Plato COMIDA QUE SU HIJO NO HA COMIDO		*90 gr*	
**COMIDA QUE SU HIJO HA COMIDO (6-7)**		*200 gr*	

MÉTODO DE ELABORACIÓN:
Explicar detalladamente el proceso de elaboración del plato

FICHA INGREDIENTES/PESOS "PLATO HECHO EN CASA (HC)" **ID: BCN 02**

DÍA DEL CONSUMO: 10 / 10 / 2014 **HORA DEL CONSUMO:** 13:30

MÉTODO DE ELABORACIÓN:
Explicar detalladamente el proceso de elaboración del plato:

Cortar y pelar las verduras.
Poner en una olla con un litro de agua y añadir la carne.
Cocer durante 20 minutos.
Triturar y añadir el aceite.

Fotos:

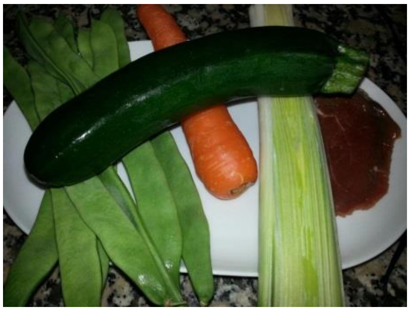

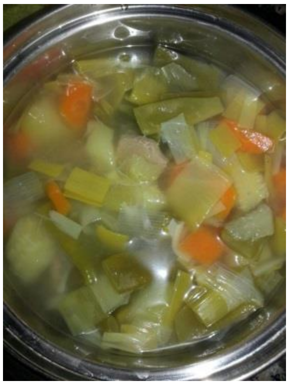

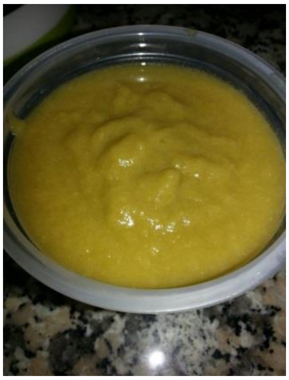

FICHA INGREDIENTES/PESOS "PLATO HECHO EN CASA (HC)" **ID: BCN 02**

DÍA DEL CONSUMO: *10 / 10 / 2014* HORA DEL CONSUMO: *17:30*

LA ELABOARACIÓN DEL PLATO SE HACE EL MISMO DÍA DEL CONSUMO [X] Sí [ ] No

Si la fecha de elaboración del plato NO es el mismo día del consumo NO se adjuntará foto y el peso de los ingredientes puede ser aproximado. Indique la fecha de elaboración: 10/10/14 Viernes

TIPO DE PLATO: *PAPILLA DE FRUTAS*

	Tipo de ingrediente	Nº	Peso (en gramos)
Ingrediente 1	*Pera*	*1*	*109 gr*
Ingrediente 2	*Plátano*	*1*	*157 gr*
Ingrediente 3	*Manzana*	*1*	*179 gr*
Ingrediente 4	*Mandarina*	*1*	*105 gr*
Ingrediente 5			
Ingrediente 6			
Ingrediente 7			
Ingrediente 8			
Ingrediente 9			
Ingrediente 10			

Proceso culinario	Nº	Peso (en gramos)	Observaciones
1. Olla VACÍA			
2. Olla con COMIDA COCINADA			
**COMIDA COCINADA (2-1)**			
3. Fiambrera VACÍA			
4. Fiambrera con COMIDA COCINADA			
**COMIDA ALMACENADA (4-3)**			
5. Plato VACÍO		*90 gr*	
6. Plato con COMIDA COCINADA		*345 gr*	
**COMIDA QUE SIRVE A SU HIJO (6-5)**		*255 gr*	
7. Plato COMIDA QUE SU HIJO NO HA COMIDO		*90 gr*	
**COMIDA QUE SU HIJO HA COMIDO (6-7)**		*255 gr*	

MÉTODO DE ELABORACIÓN:
Explicar detalladamente el proceso de elaboración del plato

FICHA INGREDIENTES/PESOS “PLATO HECHO EN CASA (HC)”

ID: BCN 02

DÍA DEL CONSUMO: 10 / 10 / 2014 **HORA DEL CONSUMO:** 17:30

MÉTODO DE ELABORACIÓN:

Explicar detalladamente el proceso de elaboración del plato:

Pelar y trocear las frutas.
Triturar y servir.

Fotos:

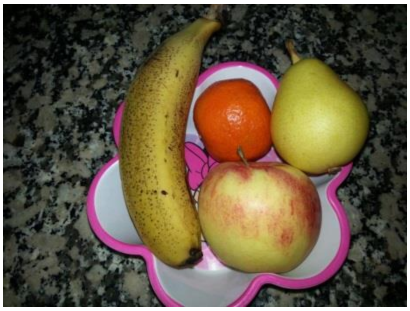

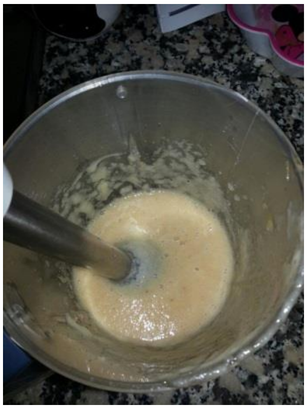

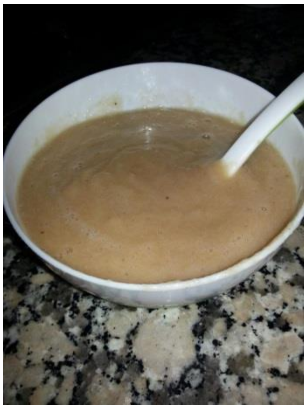

FICHA INGREDIENTES/PESOS "PLATO HECHO EN CASA (HC)" **ID: BCN 02**

DÍA DEL CONSUMO: *12 / 10 / 2014* HORA DEL CONSUMO: *14:00*

LA ELABOARACIÓN DEL PLATO SE HACE EL MISMO DÍA DEL CONSUMO ☒ Sí ☐ No

Si la fecha de elaboración del plato NO es el mismo día del consumo NO se adjuntará foto y el peso de los ingredientes puede ser aproximado. Indique la fecha de elaboración: 12/10/14 Domingo

TIPO DE PLATO: *PURÉ DE VERDURAS CON POLLO*

	Tipo de ingrediente	Nº	Peso (en gramos)
Ingrediente 1	*Calabacín*	*1*	*215 gr*
Ingrediente 2	*Zanahoria*	*1*	*127 gr*
Ingrediente 3	*Patata*	*1*	*84 gr*
Ingrediente 4	*Judía verde*	*10*	*169 gr*
Ingrediente 5	*Pollo*	*1 trozo*	*88 gr*
Ingrediente 6	*Aceite*	*1*	*Cucharita de café*
Ingrediente 7			
Ingrediente 8			
Ingrediente 9			
Ingrediente 10			

Proceso culinario	Nº	Peso (en gramos)	Observaciones
1. Olla VACÍA		*900 gr*	
2. Olla con COMIDA COCINADA		*1870 gr*	
**COMIDA COCINADA (2-1)**		*970 gr*	
3. Fiambrera VACÍA		*148 gr*	*Relleno 3 fiambreras*
4. Fiambrera con COMIDA COCINADA		*348 gr*	
**COMIDA ALMACENADA (4-3)**		*200 gr*	
5. Plato VACÍO		*90 gr*	
6. Plato con COMIDA COCINADA		*290 gr*	
**COMIDA QUE SIRVE A SU HIJO (6-5)**		*200 gr*	
7. Plato COMIDA QUE SU HIJO NO HA COMIDO		*90 gr*	
**COMIDA QUE SU HIJO HA COMIDO (6-7)**		*200 gr*	

MÉTODO DE ELABORACIÓN:

Explicar detalladamente el proceso de elaboración del plato

FICHA INGREDIENTES/PESOS "PLATO HECHO EN CASA (HC)"

ID: BCN 02

DÍA DEL CONSUMO: 12 / 10 / 2014 **HORA DEL CONSUMO:** 14:00

MÉTODO DE ELABORACIÓN:

Explicar detalladamente el proceso de elaboración del plato:

Pelar y cortar las verduras.
Poner en la olla un litro de agua y añadir las verduras y el pollo.
Cocer 20 minutos y añadir el aceite.
Triturar.

Fotos:

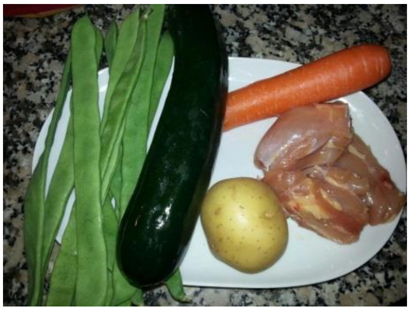

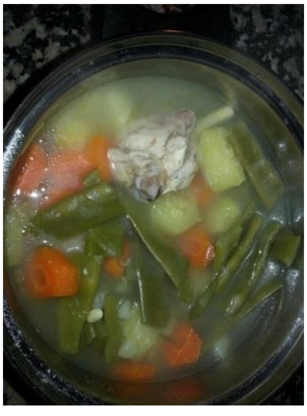

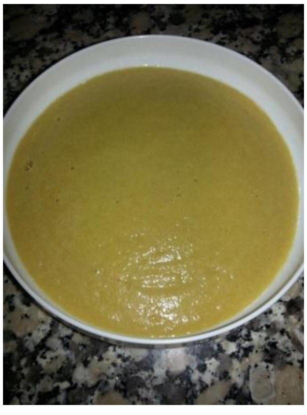

FICHA INGREDIENTES/PESOS "PLATO HECHO EN CASA (HC)" **ID: BCN 02**

DÍA DEL CONSUMO: *12 / 10 / 2014* HORA DEL CONSUMO: *17:30*

LA ELABOARACIÓN DEL PLATO SE HACE EL MISMO DÍA DEL CONSUMO [X] Sí [ ] No

Si la fecha de elaboración del plato NO es el mismo día del consumo NO se adjuntará foto y el peso de los ingredientes puede ser aproximado. Indique la fecha de elaboración: 12/10/14 Domingo

TIPO DE PLATO: *PAPILLA DE FRUTAS CON GALLETA*

	Tipo de ingrediente	Nº	Peso (en gramos)
Ingrediente 1	*Plátano*	*1*	*157 gr*
Ingrediente 2	*Manzana*	*1*	*144 gr*
Ingrediente 3	*Pera*	*1*	*136 gr*
Ingrediente 4	*Galleta Maria*	*1*	*4 gr*
Ingrediente 5	*Leche de continuación*		*30 ml de agua*
Ingrediente 6			*1 cacito de leche*
Ingrediente 7			
Ingrediente 8			
Ingrediente 9			
Ingrediente 10			

Proceso culinario	Nº	Peso (en gramos)	Observaciones
1. Olla VACÍA			
2. Olla con COMIDA COCINADA			
**COMIDA COCINADA (2-1)**			
3. Fiambrera VACÍA			
4. Fiambrera con COMIDA COCINADA			
**COMIDA ALMACENADA (4-3)**			
5. Plato VACÍO		*90 gr*	
6. Plato con COMIDA COCINADA		*310 gr*	
**COMIDA QUE SIRVE A SU HIJO (6-5)**		*220 gr*	
7. Plato COMIDA QUE SU HIJO NO HA COMIDO		*90 gr*	
**COMIDA QUE SU HIJO HA COMIDO (6-7)**		*220 gr*	

MÉTODO DE ELABORACIÓN:
Explicar detalladamente el proceso de elaboración del plato

FICHA INGREDIENTES/PESOS "PLATO HECHO EN CASA (HC)"

ID: BCN 02

DÍA DEL CONSUMO: 12 / 10 / 2014 **HORA DEL CONSUMO:** 17:30

MÉTODO DE ELABORACIÓN:

Explicar detalladamente el proceso de elaboración del plato:

Pelar y cortar las frutas.
Añadir la galleta y la leche.
Triturar.

Fotos:

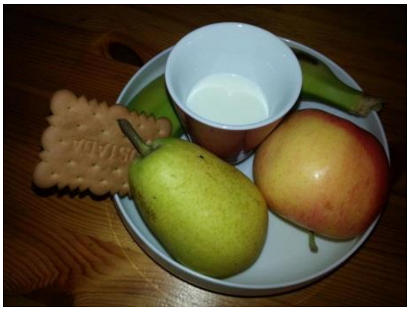

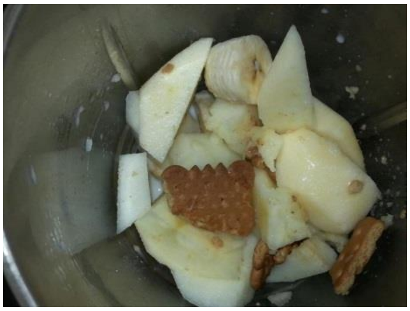

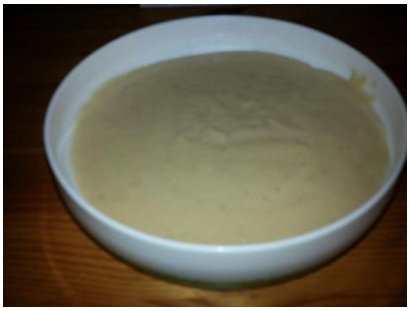

FICHA INGREDIENTES/PESOS “PLATO HECHO EN CASA (HC)” **ID: BCN 03**

DÍA DEL CONSUMO: *9 / 10 / 2014* HORA DEL CONSUMO: *13:00*

LA ELABOARACIÓN DEL PLATO SE HACE EL MISMO DÍA DEL CONSUMO [X] Sí [ ] No

Si la fecha de elaboración del plato NO es el mismo día del consumo NO se adjuntará foto y el peso de los ingredientes puede ser aproximado. Indique la fecha de elaboración: 9-10-14

TIPO DE PLATO: *PURÉ DE VERDURAS CON POLLO*

	Tipo de ingrediente	Nº	Peso (en gramos)
Ingrediente 1	*Calabacín*	*½*	*119 gr*
Ingrediente 2	*Patata*	*2*	*400 gr*
Ingrediente 3	*Judía verde*	*6*	*25,5 gr*
Ingrediente 4	*Calabaza*	*2 trozos*	*22,6 gr*
Ingrediente 5	*Pollo*	*½ pechuga*	*150 gr*
Ingrediente 6	*Puerro*	*½*	*90 gr*
Ingrediente 7	*Aceite de oliva Carrefour*		*1 chorrito*
Ingrediente 8	*Sal*		*1 pizca*
Ingrediente 9	*Agua del grifo*		*250 ml*
Ingrediente 10			

Proceso culinario	Nº	Peso (en gramos)	Observaciones
1. Olla VACÍA		*278 gr*	
2. Olla con COMIDA COCINADA		*423,1 gr*	
**COMIDA COCINADA (2-1)**		*145,1 gr*	
3. Fiambrera VACÍA			
4. Fiambrera con COMIDA COCINADA			
**COMIDA ALMACENADA (4-3)**			
5. Plato VACÍO		*99 gr*	
6. Plato con COMIDA COCINADA		*242,5 gr*	
**COMIDA QUE SIRVE A SU HIJO (6-5)**		*143,5 gr*	
7. Plato COMIDA QUE SU HIJO NO HA COMIDO		*105,7 gr*	
**COMIDA QUE SU HIJO HA COMIDO (6-7)**		*136,8 gr*	

MÉTODO DE ELABORACIÓN:
Explicar detalladamente el proceso de elaboración del plato

FICHA INGREDIENTES/PESOS “PLATO HECHO EN CASA (HC)”

ID: BCN 03

DÍA DEL CONSUMO: 9 / 10 / 2014 **HORA DEL CONSUMO:** 13:00

MÉTODO DE ELABORACIÓN:

Explicar detalladamente el proceso de elaboración del plato:

Cortar las verduras a trocitos.
Añadir agua, aceite y sal.
Cuando las verduras ya casi están, añadir el pollo.
Batir.

Fotos:

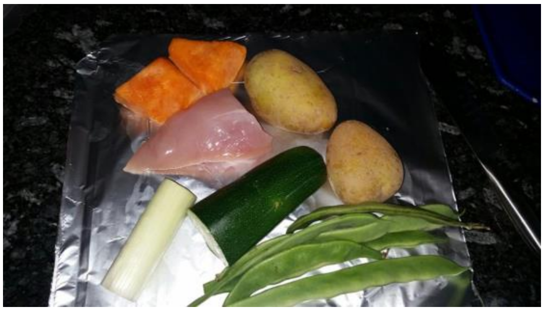

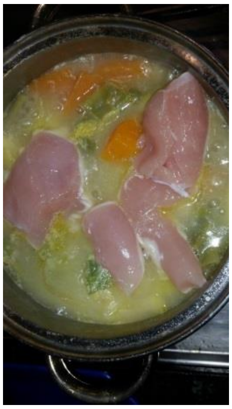

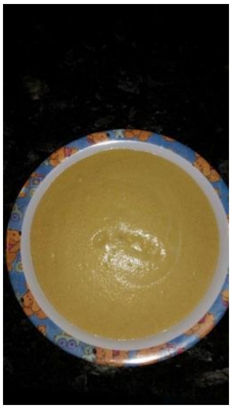

FICHA INGREDIENTES/PESOS "PLATO HECHO EN CASA (HC)" **ID: BCN 03**

DÍA DEL CONSUMO: *9 / 10 / 2014* HORA DEL CONSUMO: *18:00*

LA ELABOARACIÓN DEL PLATO SE HACE EL MISMO DÍA DEL CONSUMO [X] Sí [ ] No

Si la fecha de elaboración del plato NO es el mismo día del consumo NO se adjuntará foto y el peso de los ingredientes puede ser aproximado. Indique la fecha de elaboración: 9-10-14

TIPO DE PLATO: *PAPILLA DE FRUTAS*

	Tipo de ingrediente	Nº	Peso (en gramos)
Ingrediente 1	*Pera*	*½*	*83 gr*
Ingrediente 2	*Plátano*	*½*	*85 gr*
Ingrediente 3	*Naranja*	*1*	*199 gr*
Ingrediente 4	*Galleta "Gullón"*	*1*	*6 gr*
Ingrediente 5			
Ingrediente 6			
Ingrediente 7			
Ingrediente 8			
Ingrediente 9			
Ingrediente 10			

Proceso culinario	Nº	Peso (en gramos)	Observaciones
1. Olla VACÍA			
2. Olla con COMIDA COCINADA			
**COMIDA COCINADA (2-1)**			
3. Fiambrera VACÍA			
4. Fiambrera con COMIDA COCINADA			
**COMIDA ALMACENADA (4-3)**			
5. Plato VACÍO	*1*	*99 gr*	
6. Plato con COMIDA COCINADA	*1*	*373 gr*	
**COMIDA QUE SIRVE A SU HIJO (6-5)**		*274 gr*	
7. Plato COMIDA QUE SU HIJO NO HA COMIDO		*119 gr*	
**COMIDA QUE SU HIJO HA COMIDO (6-7)**		*254 gr*	

MÉTODO DE ELABORACIÓN:
Explicar detalladamente el proceso de elaboración del plato

FICHA INGREDIENTES/PESOS "PLATO HECHO EN CASA (HC)" **ID: BCN 03**

DÍA DEL CONSUMO: 9 / 10 / 2014 **HORA DEL CONSUMO:** 18:00

MÉTODO DE ELABORACIÓN:

Explicar detalladamente el proceso de elaboración del plato:

Trocear las frutas menos la naranja.
De la naranja echo solo el zumo.
Pasar las frutas y la galleta por la batidora.

Fotos:

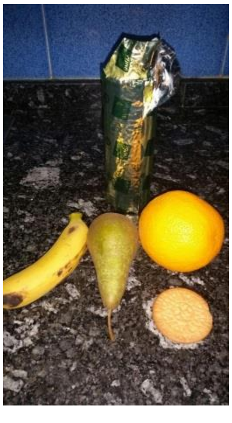

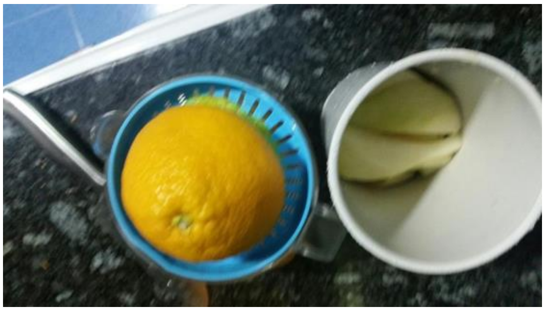

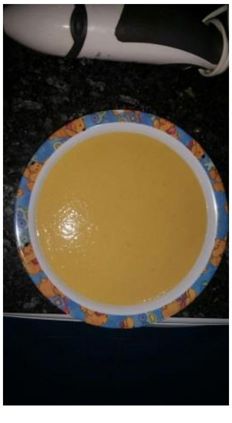

FICHA INGREDIENTES/PESOS "PLATO HECHO EN CASA (HC)" **ID: BCN 03**

DÍA DEL CONSUMO: *9 / 10 / 2014* HORA DEL CONSUMO: *21:00*

LA ELABOARACIÓN DEL PLATO SE HACE EL MISMO DÍA DEL CONSUMO [X] Sí [ ] No

Si la fecha de elaboración del plato NO es el mismo día del consumo NO se adjuntará foto y el peso de los ingredientes puede ser aproximado. Indique la fecha de elaboración: 9-10-14

TIPO DE PLATO: *PURÉ DE VERDURAS CON PESCADO*

	Tipo de ingrediente	Nº	Peso (en gramos)
Ingrediente 1	*Puerro*	*½*	*87 gr*
Ingrediente 2	*Tomate*	*1*	*147,3 gr*
Ingrediente 3	*Calabacín*	*½*	*204 gr*
Ingrediente 4	*Patata*	*2*	*433,5 gr*
Ingrediente 5	*Pescado (merluza)*	*1*	*204 gr*
Ingrediente 6	*Aceite de oliva Carrefour*		*1 chorrito*
Ingrediente 7	*Agua del grifo*		*300 ml*
Ingrediente 8			
Ingrediente 9			
Ingrediente 10			

Proceso culinario	Nº	Peso (en gramos)	Observaciones
1. Olla VACÍA		*278 gr*	
2. Olla con COMIDA COCINADA		*607,5 gr*	
**COMIDA COCINADA (2-1)**		*329,5*	
3. Fiambrera VACÍA			
4. Fiambrera con COMIDA COCINADA			
**COMIDA ALMACENADA (4-3)**			
5. Plato VACÍO		*99 gr*	
6. Plato con COMIDA COCINADA		*320 gr*	
**COMIDA QUE SIRVE A SU HIJO (6-5)**		*221 gr*	
7. Plato COMIDA QUE SU HIJO NO HA COMIDO		*181,7 gr*	
**COMIDA QUE SU HIJO HA COMIDO (6-7)**		*138,3 gr*	

MÉTODO DE ELABORACIÓN:
Explicar detalladamente el proceso de elaboración del plato

FICHA INGREDIENTES/PESOS "PLATO HECHO EN CASA (HC)"

ID: BCN 03

DÍA DEL CONSUMO: 9 / 10 / 2014 **HORA DEL CONSUMO:** 21:00

MÉTODO DE ELABORACIÓN:

Explicar detalladamente el proceso de elaboración del plato:

Cortar las verduras en trocitos.
Añadir agua del grifo hasta cubrir.
Añadir un chorrito de aceite.
Añadir una pizca de sal.
Cuando las verduras casi están, añadir el pescado.
Batir cuando el pescado está hecho.

Fotos:

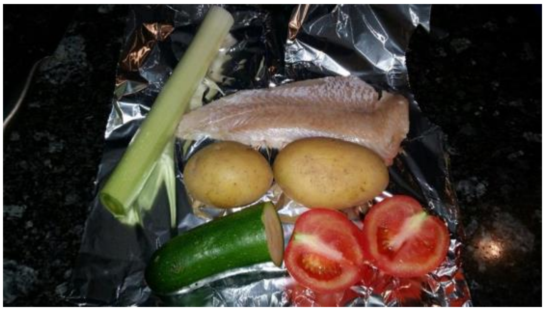

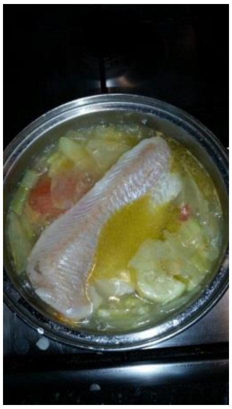

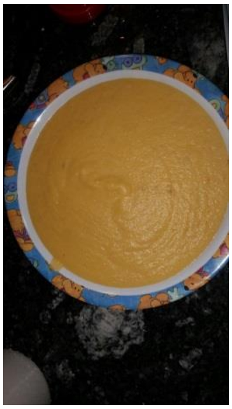

FICHA INGREDIENTES/PESOS “PLATO HECHO EN CASA (HC)” **ID: BCN 03**

DÍA DEL CONSUMO: *11 / 10 / 2014* HORA DEL CONSUMO: *13:00*

LA ELABOARACIÓN DEL PLATO SE HACE EL MISMO DÍA DEL CONSUMO [X] Sí [ ] No

Si la fecha de elaboración del plato NO es el mismo día del consumo NO se adjuntará foto y el peso de los ingredientes puede ser aproximado. Indique la fecha de elaboración: 11-10-14

TIPO DE PLATO: *PURÉ DE VERDURAS CON TERNERA*

	Tipo de ingrediente	Nº	Peso (en gramos)
Ingrediente 1	*Patatas*	*2*	*396 gr*
Ingrediente 2	*Puerro*	*½*	*77,3 gr*
Ingrediente 3	*Calabacín*	*½*	*104,8 gr*
Ingrediente 4	*Calabaza*	*2 trozos*	*201,1 gr*
Ingrediente 5	*Ternera (aguja)*	*1*	*90 gr*
Ingrediente 6	*Agua del grifo*		*250 ml*
Ingrediente 7	*Aceite de oliva Carrefour*	*1 chorrito*	*1 chorrito*
Ingrediente 8	*Sal*		*1 pizca*
Ingrediente 9	*Judías verdes*	*4*	*90,6 gr*
Ingrediente 10			

Proceso culinario	Nº	Peso (en gramos)	Observaciones
1. Olla VACÍA		*278 gr*	
2. Olla con COMIDA COCINADA		*567,8 gr*	
**COMIDA COCINADA (2-1)**		*289,8 gr*	
3. Fiambrera VACÍA			
4. Fiambrera con COMIDA COCINADA			
**COMIDA ALMACENADA (4-3)**			
5. Plato VACÍO		*99 gr*	
6. Plato con COMIDA COCINADA		*357,6 gr*	
**COMIDA QUE SIRVE A SU HIJO (6-5)**		*258,6 gr*	
7. Plato COMIDA QUE SU HIJO NO HA COMIDO		*156,7 gr*	
**COMIDA QUE SU HIJO HA COMIDO (6-7)**		*200,9 gr*	

MÉTODO DE ELABORACIÓN:
Explicar detalladamente el proceso de elaboración del plato

FICHA INGREDIENTES/PESOS “PLATO HECHO EN CASA (HC)”

ID: BCN 03

DÍA DEL CONSUMO: 11 / 10 / 2014 **HORA DEL CONSUMO:** 13:00

MÉTODO DE ELABORACIÓN:
Explicar detalladamente el proceso de elaboración del plato:

Cortar las verduras y añadir agua.
Hervir con agua y al cabo del rato, cuando las patatas ya están blanditas, poner la carne a trocitos.
Batir.

Fotos:

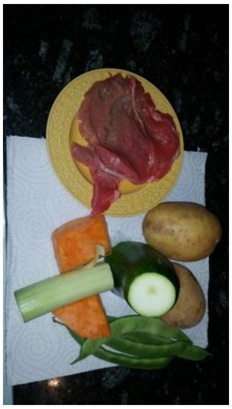

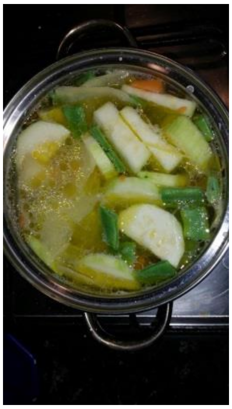

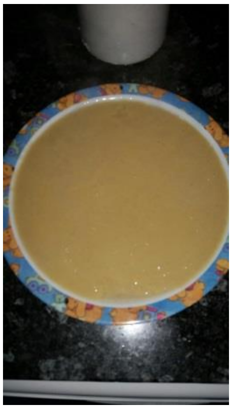

FICHA INGREDIENTES/PESOS "PLATO HECHO EN CASA (HC)" **ID: BCN 03**

DÍA DEL CONSUMO: *11 / 10 / 2014* HORA DEL CONSUMO: *18:15*

LA ELABOARACIÓN DEL PLATO SE HACE EL MISMO DÍA DEL CONSUMO [X] Sí [ ] No

Si la fecha de elaboración del plato NO es el mismo día del consumo NO se adjuntará foto y el peso de los ingredientes puede ser aproximado. Indique la fecha de elaboración: 11-10-14

TIPO DE PLATO: *PAPILLA DE FRUTAS*

	Tipo de ingrediente	Nº	Peso (en gramos)
Ingrediente 1	*Naranja*	*1*	*180 gr*
Ingrediente 2	*Plátano*	*½*	*96 gr*
Ingrediente 3	*Pera*	*½*	*89 gr*
Ingrediente 4	*Galleta "Gullón"*	*1*	*6 gr*
Ingrediente 5			
Ingrediente 6			
Ingrediente 7			
Ingrediente 8			
Ingrediente 9			
Ingrediente 10			

Proceso culinario	Nº	Peso (en gramos)	Observaciones
1. Olla VACÍA			
2. Olla con COMIDA COCINADA			
**COMIDA COCINADA (2-1)**			
3. Fiambrera VACÍA			
4. Fiambrera con COMIDA COCINADA			
**COMIDA ALMACENADA (4-3)**			
5. Plato VACÍO	*1*	*99 gr*	
6. Plato con COMIDA COCINADA	*1*	*368,5 gr*	
**COMIDA QUE SIRVE A SU HIJO (6-5)**		*269,5 gr*	
7. Plato COMIDA QUE SU HIJO NO HA COMIDO		*127,5 gr*	
**COMIDA QUE SU HIJO HA COMIDO (6-7)**		*241 gr*	

MÉTODO DE ELABORACIÓN:
Explicar detalladamente el proceso de elaboración del plato

FICHA INGREDIENTES/PESOS "PLATO HECHO EN CASA (HC)" **ID: BCN 03**

DÍA DEL CONSUMO: 11 / 10 / 2014 **HORA DEL CONSUMO:** 18:15

MÉTODO DE ELABORACIÓN:
Explicar detalladamente el proceso de elaboración del plato:

Trocear las frutas.
Hacer el zumo de naranja.
Añadir la galleta.
Batir todos los ingredientes.

Fotos:

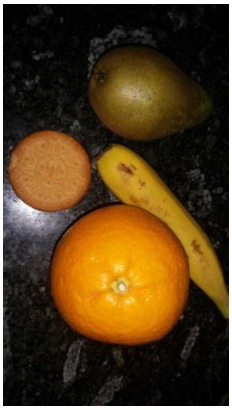

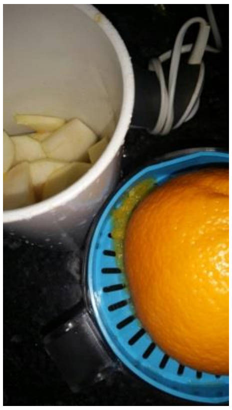

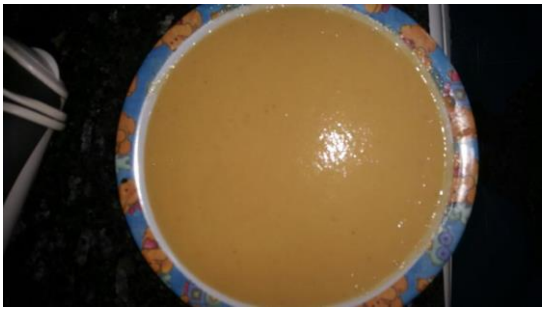

FICHA INGREDIENTES/PESOS "PLATO HECHO EN CASA (HC)" **ID: BCN 03**

DÍA DEL CONSUMO: *11 / 10 / 2014* **HORA DEL CONSUMO:** *21:00*

LA ELABOARACIÓN DEL PLATO SE HACE EL MISMO DÍA DEL CONSUMO [X] Sí [ ] No

Si la fecha de elaboración del plato NO es el mismo día del consumo NO se adjuntará foto y el peso de los ingredientes puede ser aproximado. Indique la fecha de elaboración: 11-10-14

TIPO DE PLATO: *PURÉ DE VERDURAS CON PESCADO*

	Tipo de ingrediente	Nº	Peso (en gramos)
Ingrediente 1	*Puerro*	*½*	*70,8 gr*
Ingrediente 2	*Tomate*	*1*	*158 gr*
Ingrediente 3	*Calabacín*	*½*	*113,3 gr*
Ingrediente 4	*Patata*	*2*	*340 gr*
Ingrediente 5	*Pescado (merluza)*	*1*	*189,8 gr*
Ingrediente 6	*Aceite de oliva Carrefour*		*1 chorrito*
Ingrediente 7	*Agua*		*300 ml*
Ingrediente 8			
Ingrediente 9			
Ingrediente 10			

Proceso culinario	Nº	Peso (en gramos)	Observaciones
1. Olla VACÍA		*278 gr*	
2. Olla con COMIDA COCINADA		*608,8 gr*	
**COMIDA COCINADA (2-1)**		*330,8 gr*	
3. Fiambrera VACÍA			
4. Fiambrera con COMIDA COCINADA			
**COMIDA ALMACENADA (4-3)**			
5. Plato VACÍO		*99 gr*	
6. Plato con COMIDA COCINADA		*333 gr*	
**COMIDA QUE SIRVE A SU HIJO (6-5)**		*234 gr*	
7. Plato COMIDA QUE SU HIJO NO HA COMIDO		*120,6 gr*	
**COMIDA QUE SU HIJO HA COMIDO (6-7)**		*212,4 gr*	

MÉTODO DE ELABORACIÓN:
Explicar detalladamente el proceso de elaboración del plato

FICHA INGREDIENTES/PESOS “PLATO HECHO EN CASA (HC)” **ID: BCN 03**

DÍA DEL CONSUMO: 11 / 10 / 2014 **HORA DEL CONSUMO:** 21:00

MÉTODO DE ELABORACIÓN:

Explicar detalladamente el proceso de elaboración del plato:

Cortar las verduras.
Añadir agua del grifo.
Añadir un chorrito de aceite Carrefour de oliva.
Pizca de sal.
Cuando las verduras están hechas, añadir el pescado.
Batir.

Fotos:

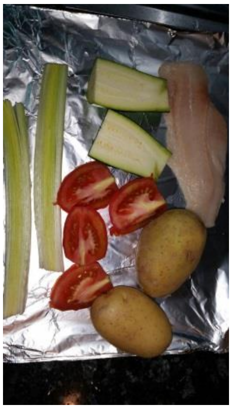

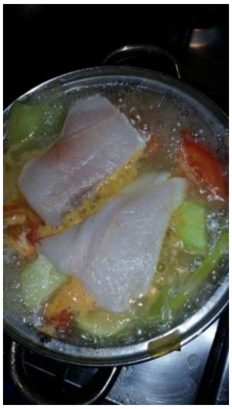

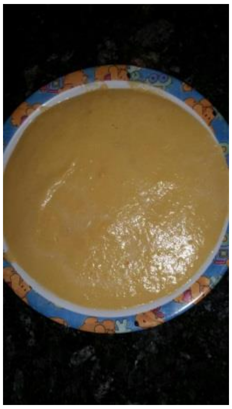

FICHA INGREDIENTES/PESOS “PLATO HECHO EN CASA (HC)” **ID: BCN 03**

DÍA DEL CONSUMO: *14 / 10 / 2014* HORA DEL CONSUMO: *13:15*

LA ELABOARACIÓN DEL PLATO SE HACE EL MISMO DÍA DEL CONSUMO [X] Sí [ ] No

Si la fecha de elaboración del plato NO es el mismo día del consumo NO se adjuntará foto y el peso de los ingredientes puede ser aproximado. Indique la fecha de elaboración: 14-10-14

TIPO DE PLATO: *PURÉ DE VERDURAS CON POLLO*

	Tipo de ingrediente	Nº	Peso (en gramos)
Ingrediente 1	*Patata*	*2*	*510 gr*
Ingrediente 2	*Calabacín*	*½*	*110,5 gr*
Ingrediente 3	*Judía verde*	*5*	*19,8 gr*
Ingrediente 4	*Calabaza*	*2 trozos*	*25,5 gr*
Ingrediente 5	*Puerro*	*½*	*82,1 gr*
Ingrediente 6	*Pollo*	*½ pechuga*	*120 gr*
Ingrediente 7	*Aceite de oliva Carrefour*	*1*	*1 chorrito*
Ingrediente 8	*Sal*		*1 pizca*
Ingrediente 9	*Agua del grifo*		*250 ml*
Ingrediente 10			

Proceso culinario	Nº	Peso (en gramos)	Observaciones
1. Olla VACÍA		*278 gr*	
2. Olla con COMIDA COCINADA		*674,4 gr*	
**COMIDA COCINADA (2-1)**		*396,4 gr*	
3. Fiambrera VACÍA			
4. Fiambrera con COMIDA COCINADA			
**COMIDA ALMACENADA (4-3)**			
5. Plato VACÍO		*99 gr*	
6. Plato con COMIDA COCINADA		*309 gr*	
**COMIDA QUE SIRVE A SU HIJO (6-5)**		*210 gr*	
7. Plato COMIDA QUE SU HIJO NO HA COMIDO		*185 gr*	
**COMIDA QUE SU HIJO HA COMIDO (6-7)**		*124 gr*	

MÉTODO DE ELABORACIÓN:

Explicar detalladamente el proceso de elaboración del plato

FICHA INGREDIENTES/PESOS "PLATO HECHO EN CASA (HC)"

ID: BCN 03

DÍA DEL CONSUMO: 14 / 10 / 2014 **HORA DEL CONSUMO:** 13:15

MÉTODO DE ELABORACIÓN:

Explicar detalladamente el proceso de elaboración del plato:

Cortar las verduras.
Añadir agua, sal y un chorrito de aceite.
Cuando las verduras ya casi están, añadir el pollo.
Batir.

Fotos:

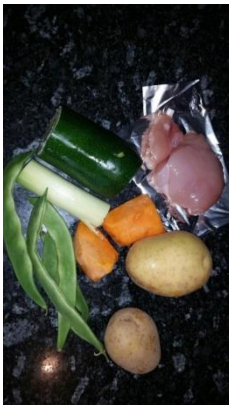

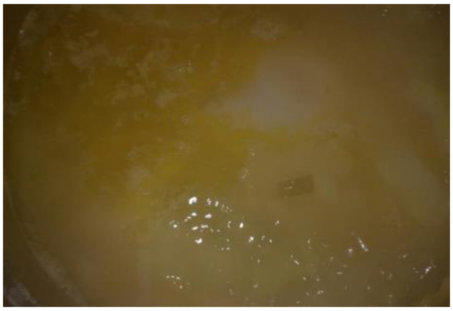

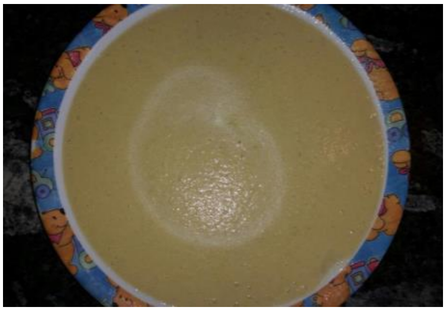

FICHA INGREDIENTES/PESOS “PLATO HECHO EN CASA (HC)” **ID: BCN 03**

DÍA DEL CONSUMO: *14 / 10 / 2014* HORA DEL CONSUMO: *18:00*

LA ELABOARACIÓN DEL PLATO SE HACE EL MISMO DÍA DEL CONSUMO [X] Sí [ ] No

Si la fecha de elaboración del plato NO es el mismo día del consumo NO se adjuntará foto y el peso de los ingredientes puede ser aproximado. Indique la fecha de elaboración: 14-10-14

TIPO DE PLATO: *PAPILLA DE FRUTAS*

	Tipo de ingrediente	Nº	Peso (en gramos)
Ingrediente 1	*Galleta*	*1*	*6 gr*
Ingrediente 2	*Naranja*	*1*	*195 gr*
Ingrediente 3	*Plátano*	*½*	*95 gr*
Ingrediente 4	*Pera*	*½*	*85 gr*
Ingrediente 5			
Ingrediente 6			
Ingrediente 7			
Ingrediente 8			
Ingrediente 9			
Ingrediente 10			

Proceso culinario	Nº	Peso (en gramos)	Observaciones
1. Olla VACÍA			
2. Olla con COMIDA COCINADA			
**COMIDA COCINADA (2-1)**			
3. Fiambrera VACÍA			
4. Fiambrera con COMIDA COCINADA			
**COMIDA ALMACENADA (4-3)**			
5. Plato VACÍO	*1*	*99 gr*	
6. Plato con COMIDA COCINADA	*1*	*369,1 gr*	
**COMIDA QUE SIRVE A SU HIJO (6-5)**		*270,1 gr*	
7. Plato COMIDA QUE SU HIJO NO HA COMIDO		*105 gr*	
**COMIDA QUE SU HIJO HA COMIDO (6-7)**		*264,1 gr*	

MÉTODO DE ELABORACIÓN:
Explicar detalladamente el proceso de elaboración del plato

FICHA INGREDIENTES/PESOS "PLATO HECHO EN CASA (HC)"

ID: BCN 03

DÍA DEL CONSUMO: 14 / 10 / 2014 **HORA DEL CONSUMO:** 18:00

MÉTODO DE ELABORACIÓN:
Explicar detalladamente el proceso de elaboración del plato:

Trocear las frutas.
Hacer zumo de naranja.
Añadir galleta.
Batir.

Fotos:

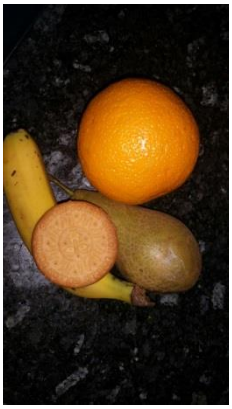

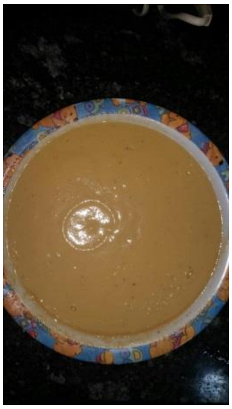

FICHA INGREDIENTES/PESOS “PLATO HECHO EN CASA (HC)” **ID: BCN 03**

DÍA DEL CONSUMO: *14 / 10 / 2014* HORA DEL CONSUMO: 21*:00*

LA ELABOARACIÓN DEL PLATO SE HACE EL MISMO DÍA DEL CONSUMO [X] Sí [ ] No

Si la fecha de elaboración del plato NO es el mismo día del consumo NO se adjuntará foto y el peso de los ingredientes puede ser aproximado. Indique la fecha de elaboración: 14-10-14

TIPO DE PLATO: *PURÉ DE VERDURAS CON PESCADO*

	Tipo de ingrediente	Nº	Peso (en gramos)
Ingrediente 1	*Puerro*	*½*	*90,6 gr*
Ingrediente 2	*Tomate*	*1*	*206,8 gr*
Ingrediente 3	*Patata*	*2*	*439,1 gr*
Ingrediente 4	*Calabacín*	*½*	*90,6 gr*
Ingrediente 5	*Aceite de oliva Carrefour*		*1 chorrito*
Ingrediente 6	*Pescado (merluza)*	*1*	*232,3 gr*
Ingrediente 7	*Agua del grifo*		*300 ml*
Ingrediente 8			
Ingrediente 9			
Ingrediente 10			

Proceso culinario	Nº	Peso (en gramos)	Observaciones
1. Olla VACÍA		*278 gr*	
2. Olla con COMIDA COCINADA		*615,4 gr*	
**COMIDA COCINADA (2-1)**		*337,4 gr*	
3. Fiambrera VACÍA			
4. Fiambrera con COMIDA COCINADA			
**COMIDA ALMACENADA (4-3)**			
5. Plato VACÍO		*99 gr*	
6. Plato con COMIDA COCINADA		*340 gr*	
**COMIDA QUE SIRVE A SU HIJO (6-5)**		*241 gr*	
7. Plato COMIDA QUE SU HIJO NO HA COMIDO		*110,7 gr*	
**COMIDA QUE SU HIJO HA COMIDO (6-7)**		*229,3 gr*	

MÉTODO DE ELABORACIÓN:
Explicar detalladamente el proceso de elaboración del plato

FICHA INGREDIENTES/PESOS "PLATO HECHO EN CASA (HC)"

ID: BCN 03

DÍA DEL CONSUMO: 14 / 10 / 2014 **HORA DEL CONSUMO:** 21:00

MÉTODO DE ELABORACIÓN:

Explicar detalladamente el proceso de elaboración del plato:

Cortar las verduras a trocitos.
Cubrir con agua.
Añadir chorrito de aceite y pizca de sal.
Cuando esté hecho, añadir el pescado y que hierva un poquito.
Batir todo.

Fotos:

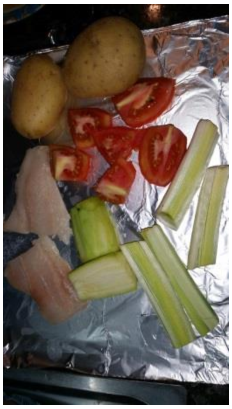

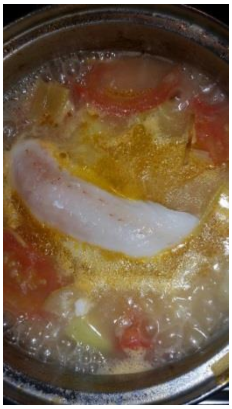

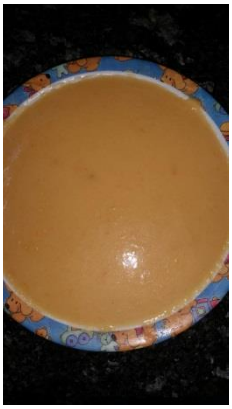

FICHA INGREDIENTES/PESOS "PLATO HECHO EN CASA (HC)" **ID: BCN 04**

DÍA DEL CONSUMO: *8 / 10 / 2014* HORA DEL CONSUMO: *12:37*

LA ELABOARACIÓN DEL PLATO SE HACE EL MISMO DÍA DEL CONSUMO [X] Sí [ ] No

Si la fecha de elaboración del plato NO es el mismo día del consumo NO se adjuntará foto y el peso de los ingredientes puede ser aproximado. Indique la fecha de elaboración:

TIPO DE PLATO: *PURÉ DE VERDURAS CON PESCADO*

	Tipo de ingrediente	Nº	Peso (en gramos)
Ingrediente 1	*Patata*	*1*	*232 gr*
Ingrediente 2	*Zanahoria*	*2*	*95 gr*
Ingrediente 3	*Calabaza*	*1 trozo*	*15 gr*
Ingrediente 4	*Judías verdes*	*1*	*6 gr*
Ingrediente 5	*Cebolla*	*¼*	*18 gr*
Ingrediente 6	*Pescado (gallo)*	*2 filetes*	*67 gr*
Ingrediente 7	*Aceite de oliva*	*1 chorrito*	
Ingrediente 8			
Ingrediente 9			
Ingrediente 10			

Proceso culinario	Nº	Peso (en gramos)	Observaciones
1. Olla VACÍA		*784 gr*	
2. Olla con COMIDA COCINADA		*1434 gr*	
**COMIDA COCINADA (2-1)**		*650 gr*	
3. Fiambrera VACÍA			
4. Fiambrera con COMIDA COCINADA			
**COMIDA ALMACENADA (4-3)**			
5. Plato VACÍO		*230 gr*	
6. Plato con COMIDA COCINADA		*434 gr*	
**COMIDA QUE SIRVE A SU HIJO (6-5)**		*204 gr*	
7. Plato COMIDA QUE SU HIJO NO HA COMIDO		*230 gr*	*Se lo ha comido todo*
**COMIDA QUE SU HIJO HA COMIDO (6-7)**		*204 gr*	*Se lo ha comido todo*

MÉTODO DE ELABORACIÓN:
Explicar detalladamente el proceso de elaboración del plato

FICHA INGREDIENTES/PESOS “PLATO HECHO EN CASA (HC)”

ID: BCN 04

DÍA DEL CONSUMO: 8 / 10 / 2014 **HORA DEL CONSUMO:** 12:37

MÉTODO DE ELABORACIÓN:

Explicar detalladamente el proceso de elaboración del plato:

Pelar y cortar las verduras.
Poner en una cazuela un poco menos de la mitad de agua.
Añadir las verdura, un chorrito de aceite de oliva virgen extra.
Cocer durante 20 minutos.
5 minutos antes de acabar la cocción, añadir el pescado.
Transcurrido el tiempo de cocción, triturar las verduras junto con el pescado.

Fotos:

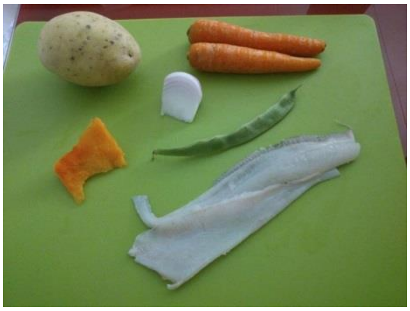

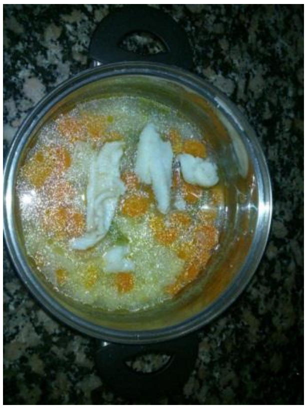

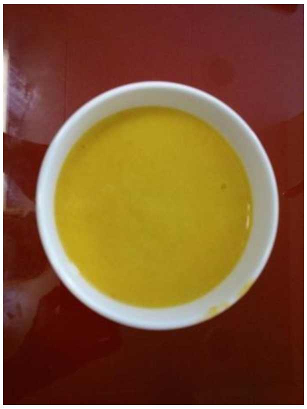

FICHA INGREDIENTES/PESOS "PLATO HECHO EN CASA (HC)" **ID: BCN 04**

DÍA DEL CONSUMO: *8 / 10 / 2014* HORA DEL CONSUMO: *18:45*

LA ELABOARACIÓN DEL PLATO SE HACE EL MISMO DÍA DEL CONSUMO [X] Sí [ ] No

Si la fecha de elaboración del plato NO es el mismo día del consumo NO se adjuntará foto y el peso de los ingredientes puede ser aproximado. Indique la fecha de elaboración:

TIPO DE PLATO: *PAPILLA DE FRUTAS*

	Tipo de ingrediente	Nº	Peso (en gramos)
Ingrediente 1	*Naranja*	*1*	*192 gr*
Ingrediente 2	*Pera*	*1*	*189 gr*
Ingrediente 3	*Manzana*	*1*	*188 gr*
Ingrediente 4	*Plátano*	*1*	*153 gr*
Ingrediente 5			
Ingrediente 6			
Ingrediente 7			
Ingrediente 8			
Ingrediente 9			
Ingrediente 10			

Proceso culinario	Nº	Peso (en gramos)	Observaciones
1. Olla VACÍA		*113 gr*	
2. Olla con COMIDA COCINADA		*510 gr*	
**COMIDA COCINADA (2-1)**		*397 gr*	
3. Fiambrera VACÍA			
4. Fiambrera con COMIDA COCINADA			
**COMIDA ALMACENADA (4-3)**			
5. Plato VACÍO		*66 gr*	
6. Plato con COMIDA COCINADA		*298 gr*	
**COMIDA QUE SIRVE A SU HIJO (6-5)**		*232 gr*	
7. Plato COMIDA QUE SU HIJO NO HA COMIDO		*85 gr*	
**COMIDA QUE SU HIJO HA COMIDO (6-7)**		*213 gr*	

MÉTODO DE ELABORACIÓN:
Explicar detalladamente el proceso de elaboración del plato

FICHA INGREDIENTES/PESOS "PLATO HECHO EN CASA (HC)"

ID: BCN 04

DÍA DEL CONSUMO: 8 / 10 / 2014 **HORA DEL CONSUMO:** 18:45

MÉTODO DE ELABORACIÓN:

Explicar detalladamente el proceso de elaboración del plato:

Pelar y cortar la fruta.
Echar en un recipiente el zumo de naranja y a continuación añadir el resto de la fruta cortada.
Triturar.

Fotos:

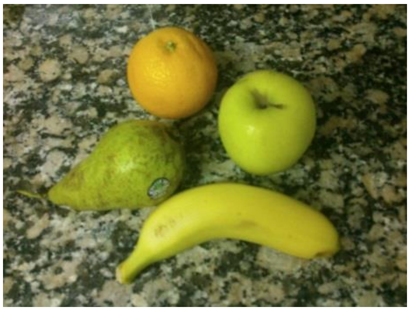

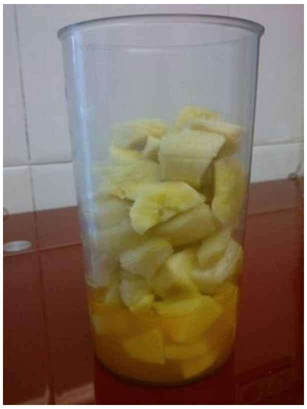

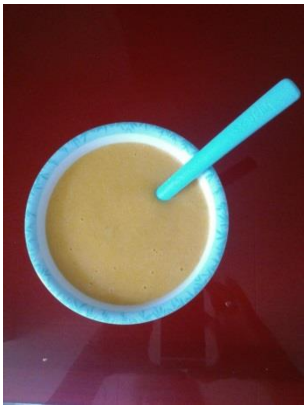

FICHA INGREDIENTES/PESOS "PLATO HECHO EN CASA (HC)" **ID: BCN 04**

DÍA DEL CONSUMO: *9 / 10 / 2014* HORA DEL CONSUMO: *13:00*

LA ELABOARACIÓN DEL PLATO SE HACE EL MISMO DÍA DEL CONSUMO ☒ Sí ☐ No

Si la fecha de elaboración del plato NO es el mismo día del consumo NO se adjuntará foto y el peso de los ingredientes puede ser aproximado. Indique la fecha de elaboración:

TIPO DE PLATO: *PÚRE DE VERDURAS CON POLLO*

	Tipo de ingrediente	Nº	Peso (en gramos)
Ingrediente 1	*Pollo*	*2 pechugas*	*221 gr*
Ingrediente 2	*Zanahoria*	*3*	*312 gr*
Ingrediente 3	*Judías verdes*	*2*	*37 gr*
Ingrediente 4	*Calabaza*	*1 trozo*	*72 gr*
Ingrediente 5	*Cebolla*	*½*	*38 gr*
Ingrediente 6	*Patata*	*2*	*610 gr*
Ingrediente 7	*Aceite de oliva virgen extra*	*1 chorrito*	
Ingrediente 8			
Ingrediente 9			
Ingrediente 10			

Proceso culinario	Nº	Peso (en gramos)	Observaciones
1. Olla VACÍA		*964 gr*	
2. Olla con COMIDA COCINADA		*3037 gr*	
**COMIDA COCINADA (2-1)**		*2073 gr*	
3. Fiambrera VACÍA (tarro de cristal)	*6*	*145 gr*	*Relleno 6 tarros*
4. Fiambrera con COMIDA COCINADA		*370 gr*	
**COMIDA ALMACENADA (4-3)**		*225 gr*	
5. Plato VACÍO		*230 gr*	
6. Plato con COMIDA COCINADA		*482 gr*	
**COMIDA QUE SIRVE A SU HIJO (6-5)**		*252 gr*	
7. Plato COMIDA QUE SU HIJO NO HA COMIDO		*230 gr*	*Se lo ha comido todo*
**COMIDA QUE SU HIJO HA COMIDO (6-7)**		*252 gr*	*Se lo ha comido todo*

MÉTODO DE ELABORACIÓN:
Explicar detalladamente el proceso de elaboración del plato

FICHA INGREDIENTES/PESOS "PLATO HECHO EN CASA (HC)" **ID: BCN 04**

DÍA DEL CONSUMO: 9 / 10 / 2014 **HORA DEL CONSUMO:** 13:00

MÉTODO DE ELABORACIÓN:

Explicar detalladamente el proceso de elaboración del plato:

Pelar y trocear las verduras.
Filetear el pollo.
Hervir durante 20 minutos.
Añadir un chorro de aceite.

Fotos:

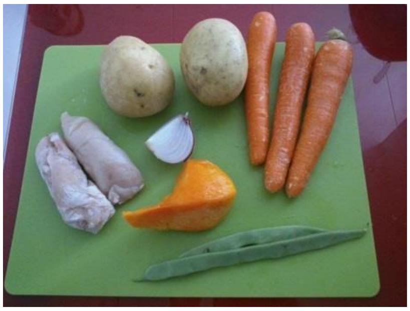

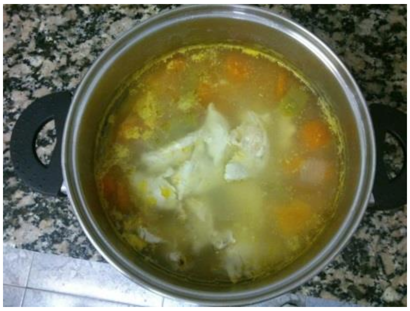

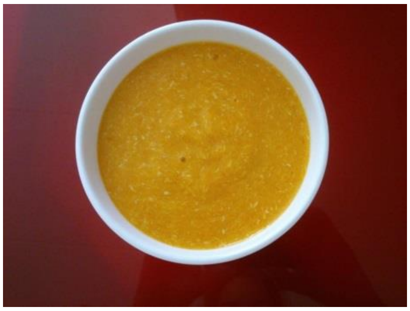

FICHA INGREDIENTES/PESOS “PLATO HECHO EN CASA (HC)” **ID: BCN 04**

DÍA DEL CONSUMO: *9 / 10 / 2014* HORA DEL CONSUMO: *18:00*

LA ELABOARACIÓN DEL PLATO SE HACE EL MISMO DÍA DEL CONSUMO [X] Sí [ ] No

Si la fecha de elaboración del plato NO es el mismo día del consumo NO se adjuntará foto y el peso de los ingredientes puede ser aproximado. Indique la fecha de elaboración:

TIPO DE PLATO: *PAPILLA DE FRUTAS*

	Tipo de ingrediente	Nº	Peso (en gramos)
Ingrediente 1	*Zumo de naranja*	*1*	*191 gr*
Ingrediente 2	*Pera*	*1*	*188 gr*
Ingrediente 3	*Manzana*	*1*	*187 gr*
Ingrediente 4	*Plátano*		*151 gr*
Ingrediente 5			
Ingrediente 6			
Ingrediente 7			
Ingrediente 8			
Ingrediente 9			
Ingrediente 10			

Proceso culinario	Nº	Peso (en gramos)	Observaciones
1. Olla VACÍA		*111 gr*	
2. Olla con COMIDA COCINADA		*522 gr*	
**COMIDA COCINADA (2-1)**		*411 gr*	
3. Fiambrera VACÍA			
4. Fiambrera con COMIDA COCINADA			
**COMIDA ALMACENADA (4-3)**			
5. Plato VACÍO		*66 gr*	
6. Plato con COMIDA COCINADA		*233 gr*	
**COMIDA QUE SIRVE A SU HIJO (6-5)**		*167 gr*	
7. Plato COMIDA QUE SU HIJO NO HA COMIDO		*126 gr*	
**COMIDA QUE SU HIJO HA COMIDO (6-7)**		*107 gr*	

MÉTODO DE ELABORACIÓN:
Explicar detalladamente el proceso de elaboración del plato

FICHA INGREDIENTES/PESOS "PLATO HECHO EN CASA (HC)"

ID: BCN 04

DÍA DEL CONSUMO: 9 / 10 / 2014 **HORA DEL CONSUMO:** 18:00

MÉTODO DE ELABORACIÓN:

Explicar detalladamente el proceso de elaboración del plato:

Pelar y trocear la fruta.
Exprimir el zumo de la naranja y añadir el resto de la fruta.
Triturar todo junto.

Fotos:

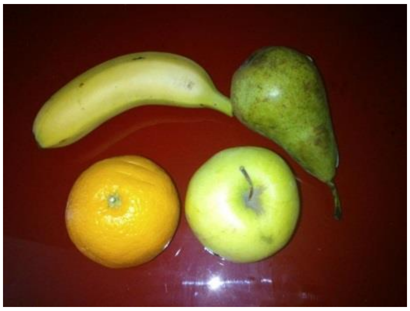

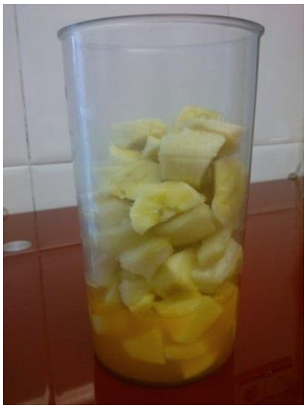

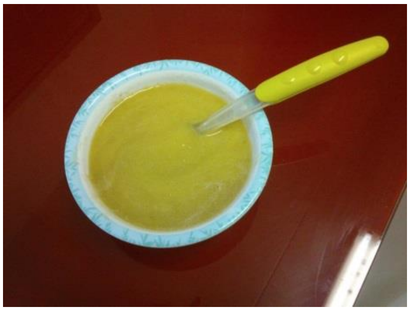

FICHA INGREDIENTES/PESOS "PLATO HECHO EN CASA (HC)" **ID: BCN 04**

DÍA DEL CONSUMO: *12 / 10 / 2014* HORA DEL CONSUMO: *13:00*

LA ELABOARACIÓN DEL PLATO SE HACE EL MISMO DÍA DEL CONSUMO [X] Sí [ ] No

Si la fecha de elaboración del plato NO es el mismo día del consumo NO se adjuntará foto y el peso de los ingredientes puede ser aproximado. Indique la fecha de elaboración:

TIPO DE PLATO: *PURÉ DE VERDURAS CON PESCADO*

	Tipo de ingrediente	Nº	Peso (en gramos)
Ingrediente 1	*Patatas*	*1*	*204 gr*
Ingrediente 2	*Zanahoria*	*1*	*125 gr*
Ingrediente 3	*Calabaza*	*2 trocitos*	*23 gr*
Ingrediente 4	*Judía verde*	*1*	*8 gr*
Ingrediente 5	*Aceite de oliva*	*1 chorrito*	
Ingrediente 6	*Pescado (gallo)*	*2 filete*	
Ingrediente 7			
Ingrediente 8			
Ingrediente 9			
Ingrediente 10			

Proceso culinario	Nº	Peso (en gramos)	Observaciones
1. Olla VACÍA		*786 gr*	
2. Olla con COMIDA COCINADA		*1275 gr*	
**COMIDA COCINADA (2-1)**		*489 gr*	
3. Fiambrera VACÍA			
4. Fiambrera con COMIDA COCINADA			
**COMIDA ALMACENADA (4-3)**			
5. Plato VACÍO		*66 gr*	
6. Plato con COMIDA COCINADA		*251 gr*	
**COMIDA QUE SIRVE A SU HIJO (6-5)**		*185 gr*	
7. Plato COMIDA QUE SU HIJO NO HA COMIDO		*66 gr*	*Se lo ha comido todo*
**COMIDA QUE SU HIJO HA COMIDO (6-7)**		*185 gr*	*Se lo ha comido todo*

MÉTODO DE ELABORACIÓN:

Explicar detalladamente el proceso de elaboración del plato

FICHA INGREDIENTES/PESOS "PLATO HECHO EN CASA (HC)"

ID: BCN 04

DÍA DEL CONSUMO: 12 / 10 / 2014 **HORA DEL CONSUMO:** 13:00

MÉTODO DE ELABORACIÓN:

Explicar detalladamente el proceso de elaboración del plato:

Pelar y trocear las verduras.
En una cazuela, poner menos de la mitad de agua.
Añadir las verduras junto con el chorro de aceite de oliva.
Cocer durante 20 minutos.
5 minutos antes de acabar la cocción, añadir el pescado.
Transcurrido el tiempo se tritura todo junto.

Fotos:

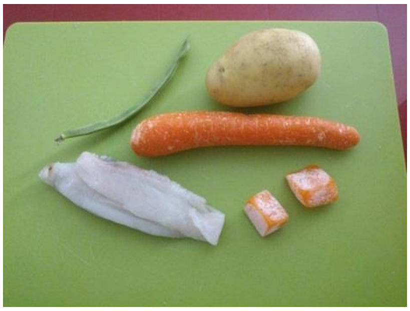

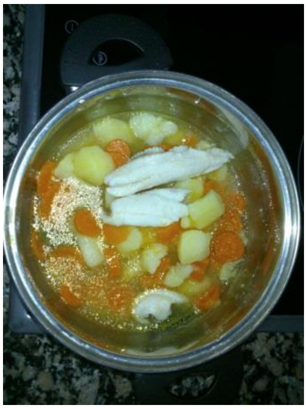

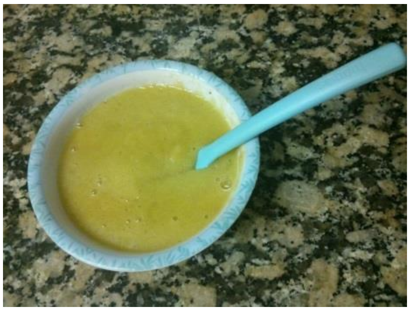

FICHA INGREDIENTES/PESOS "PLATO HECHO EN CASA (HC)" **ID: BCN 04**

DÍA DEL CONSUMO: *12 / 10 / 2014* HORA DEL CONSUMO: *18:00*

LA ELABOARACIÓN DEL PLATO SE HACE EL MISMO DÍA DEL CONSUMO [X] Sí [ ] No

Si la fecha de elaboración del plato NO es el mismo día del consumo NO se adjuntará foto y el peso de los ingredientes puede ser aproximado. Indique la fecha de elaboración:

TIPO DE PLATO: *PAPILLA DE FRUTAS*

	Tipo de ingrediente	Nº	Peso (en gramos)
Ingrediente 1	*Naranja*	*1*	*199 gr*
Ingrediente 2	*Manzana*	*1*	*283 gr*
Ingrediente 3	*Pera*	*1*	*219 gr*
Ingrediente 4	*Plátano*	*1*	*148 gr*
Ingrediente 5			
Ingrediente 6			
Ingrediente 7			
Ingrediente 8			
Ingrediente 9			
Ingrediente 10			

Proceso culinario	Nº	Peso (en gramos)	Observaciones
1. Olla VACÍA			
2. Olla con COMIDA COCINADA		*620 gr*	
**COMIDA COCINADA (2-1)**			
3. Fiambrera VACÍA			
4. Fiambrera con COMIDA COCINADA			
**COMIDA ALMACENADA (4-3)**			
5. Plato VACÍO		*66 gr*	
6. Plato con COMIDA COCINADA		*241 gr*	
**COMIDA QUE SIRVE A SU HIJO (6-5)**		*175 gr*	
7. Plato COMIDA QUE SU HIJO NO HA COMIDO		*66 gr*	*Se lo ha comido todo*
**COMIDA QUE SU HIJO HA COMIDO (6-7)**		*175 gr*	*Se lo ha comido todo*

MÉTODO DE ELABORACIÓN:
Explicar detalladamente el proceso de elaboración del plato

FICHA INGREDIENTES/PESOS "PLATO HECHO EN CASA (HC)"

ID: BCN 04

DÍA DEL CONSUMO: 12 / 10 / 2014 **HORA DEL CONSUMO:** 18:00

MÉTODO DE ELABORACIÓN:

Explicar detalladamente el proceso de elaboración del plato:

Se pela y trocea la fruta.
Se exprime la naranja y se añade el resto de la fruta.
Se tritura todo.

Fotos:

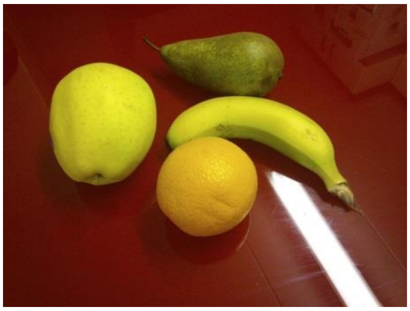

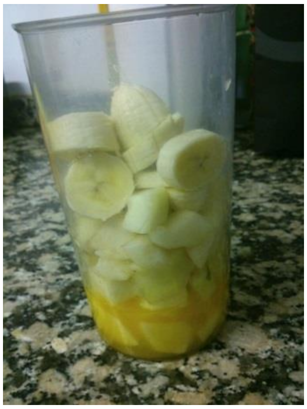

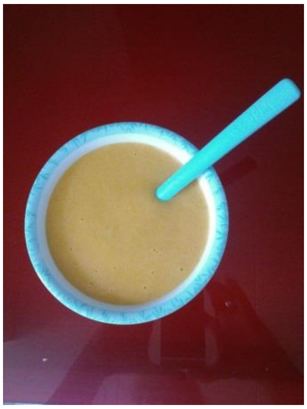

FICHA INGREDIENTES/PESOS “PLATO HECHO EN CASA (HC)” **ID: BCN 05**

DÍA DEL CONSUMO: *9 / 10 / 2014* HORA DEL CONSUMO: *20:15*

LA ELABOARACIÓN DEL PLATO SE HACE EL MISMO DÍA DEL CONSUMO [X] Sí [ ] No

Si la fecha de elaboración del plato NO es el mismo día del consumo NO se adjuntará foto y el peso de los ingredientes puede ser aproximado. Indique la fecha de elaboración: 9/10/2014

TIPO DE PLATO: *PURÉ DE VERDURAS CON TERNERA*

	Tipo de ingrediente	Nº	Peso (en gramos)
Ingrediente 1	*Agua*		*½ litro*
Ingrediente 2	*Calabacín*	*1*	*214 gr*
Ingrediente 3	*Patatas*	*2*	*326 gr*
Ingrediente 4	*Zanahoria*	*1*	*100 gr*
Ingrediente 5	*Ternera*	*1*	*80 gr*
Ingrediente 6	*Aceite*	*1*	*Chorrito, cucharada sopera*
Ingrediente 7	*Sal*	*1*	*1 pizca*
Ingrediente 8			
Ingrediente 9			
Ingrediente 10			

Proceso culinario	Nº	Peso (en gramos)	Observaciones
1. Olla VACÍA		*537 gr*	
2. Olla con COMIDA COCINADA		*1.139 gr*	
**COMIDA COCINADA (2-1)**		*602 gr*	
3. Fiambrera VACÍA			
4. Fiambrera con COMIDA COCINADA			
**COMIDA ALMACENADA (4-3)**			
5. Plato VACÍO		*73 gr*	
6. Plato con COMIDA COCINADA		*392 gr*	
**COMIDA QUE SIRVE A SU HIJO (6-5)**		*319 gr*	
7. Plato COMIDA QUE SU HIJO NO HA COMIDO		*131 gr*	
**COMIDA QUE SU HIJO HA COMIDO (6-7)**		*261 gr*	

MÉTODO DE ELABORACIÓN:
Explicar detalladamente el proceso de elaboración del plato

FICHA INGREDIENTES/PESOS “PLATO HECHO EN CASA (HC)”

ID: BCN 05

DÍA DEL CONSUMO: 9 / 10 / 2014 **HORA DEL CONSUMO:** 20:15

MÉTODO DE ELABORACIÓN:

Explicar detalladamente el proceso de elaboración del plato:

Poner agua en la olla, ¾ partes de agua, llevar a hervir.
Añadir la zanahoria a rodajas, que hierva 5 minutos.
Añadir la patata.
Añadir el calabacín también troceado.
Añadir un chorrito de aceite y una pizca de sal.
Por último añadir la ternera.
Dejar cocer de 15 a 20 minutos.
Sacar un poco de caldo antes de triturar para que no quede demasiado líquida, si no siempre estamos a tiempo de añadirle más caldo.

Fotos:

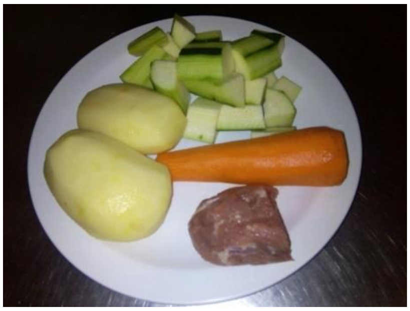

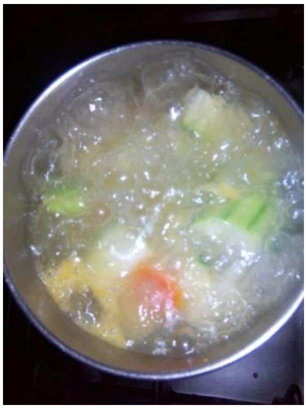

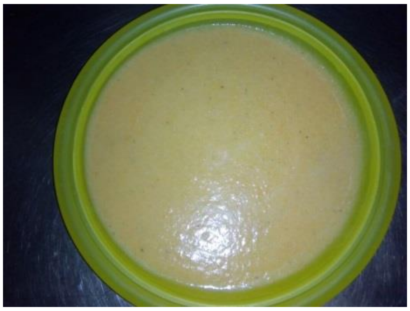

FICHA INGREDIENTES/PESOS "PLATO HECHO EN CASA (HC)" **ID: BCN 05**

DÍA DEL CONSUMO: *10 / 10 / 2014* HORA DEL CONSUMO: *20:05*

LA ELABOARACIÓN DEL PLATO SE HACE EL MISMO DÍA DEL CONSUMO [X] Sí [ ] No

Si la fecha de elaboración del plato NO es el mismo día del consumo NO se adjuntará foto y el peso de los ingredientes puede ser aproximado. Indique la fecha de elaboración: 10/10/2014

TIPO DE PLATO: *PURÉ DE VERDURAS CON PESCADO*

	Tipo de ingrediente	Nº	Peso (en gramos)
Ingrediente 1	*Agua*	*½ litro*	
Ingrediente 2	*Zanahoria*	*1*	*146 gr*
Ingrediente 3	*Calabacín*	*1*	*250 gr*
Ingrediente 4	*Judía verde*	*Puñado*	*158 gr*
Ingrediente 5	*Aceite*	*1*	*Chorrito*
Ingrediente 6	*Sal*	*1*	*1 pizca*
Ingrediente 7	*Pescado (panga)*	*1*	*135 gr*
Ingrediente 8			
Ingrediente 9			
Ingrediente 10			

Proceso culinario	Nº	Peso (en gramos)	Observaciones
1. Olla VACÍA		*537 gr*	
2. Olla con COMIDA COCINADA		*1.124 gr*	
**COMIDA COCINADA (2-1)**		*587 gr*	
3. Fiambrera VACÍA			
4. Fiambrera con COMIDA COCINADA			
**COMIDA ALMACENADA (4-3)**			
5. Plato VACÍO		*73 gr*	
6. Plato con COMIDA COCINADA		*409 gr*	
**COMIDA QUE SIRVE A SU HIJO (6-5)**		*336 gr*	
7. Plato COMIDA QUE SU HIJO NO HA COMIDO		*168 gr*	
**COMIDA QUE SU HIJO HA COMIDO (6-7)**		*241 gr*	

MÉTODO DE ELABORACIÓN:
Explicar detalladamente el proceso de elaboración del plato

FICHA INGREDIENTES/PESOS "PLATO HECHO EN CASA (HC)"

ID: BCN 05

DÍA DEL CONSUMO: 10 / 10 / 2014 **HORA DEL CONSUMO:** 20:05

MÉTODO DE ELABORACIÓN:

Explicar detalladamente el proceso de elaboración del plato:

Poner al fuego la olla, ¾ partes de agua, cuando hierva añadir un chorrito de aceite y una pizca de sal.

Añadir la zanahoria y las judías verdes, cocer de 10 a 15 minutos.

Añadir el calabacín, hervir 5 minutos más.

Añadir el pescado, cocer 10 minutos más.

Retirar un poco de caldo antes de triturar para poder añadirle si hace falta y no quede demasiado líquido.

Fotos:

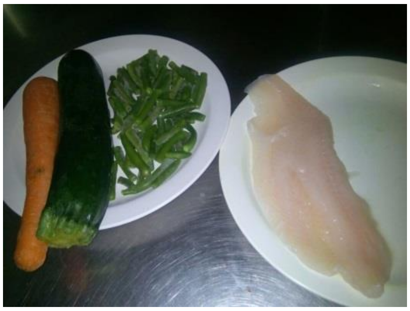

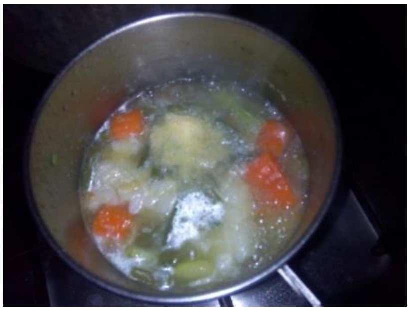

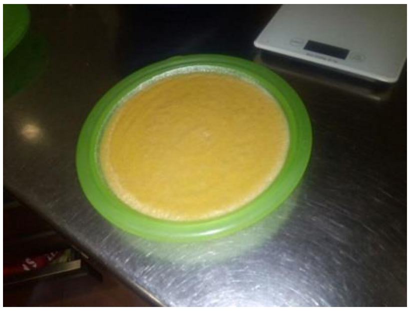

FICHA INGREDIENTES/PESOS "PLATO HECHO EN CASA (HC)" **ID: BCN 05**

DÍA DEL CONSUMO: *11 / 10 / 2014* HORA DEL CONSUMO: *13:35*

LA ELABOARACIÓN DEL PLATO SE HACE EL MISMO DÍA DEL CONSUMO [X] Sí [ ] No

Si la fecha de elaboración del plato NO es el mismo día del consumo NO se adjuntará foto y el peso de los ingredientes puede ser aproximado. Indique la fecha de elaboración: 11/10/2014

TIPO DE PLATO: *ARROZ CALDOSO CON POLLO Y VERDURAS*

	Tipo de ingrediente	Nº	Peso (en gramos)
Ingrediente 1	*Puerro*	*½*	*165 gr*
Ingrediente 2	*Zanahoria*	*1*	*107 gr*
Ingrediente 3	*Arroz*	*Puñado*	*100 gr*
Ingrediente 4	*Muslo de pollo*	*¼*	*200 gr*
Ingrediente 5	*Aceite*	*1 cucharada de postre*	
Ingrediente 6	*Sal*	*1 pizca*	
Ingrediente 7	*Agua*		*½ litro*
Ingrediente 8			
Ingrediente 9			
Ingrediente 10			

Proceso culinario	Nº	Peso (en gramos)	Observaciones
1. Olla VACÍA		*537 gr*	
2. Olla con COMIDA COCINADA		*1.460 gr*	
**COMIDA COCINADA (2-1)**		*537 gr*	
3. Fiambrera VACÍA			
4. Fiambrera con COMIDA COCINADA			
**COMIDA ALMACENADA (4-3)**			
5. Plato VACÍO		*73 gr*	
6. Plato con COMIDA COCINADA		*386 gr*	
**COMIDA QUE SIRVE A SU HIJO (6-5)**		*313 gr*	
7. Plato COMIDA QUE SU HIJO NO HA COMIDO		*108 gr*	
**COMIDA QUE SU HIJO HA COMIDO (6-7)**		*278 gr*	

MÉTODO DE ELABORACIÓN:

Explicar detalladamente el proceso de elaboración del plato

FICHA INGREDIENTES/PESOS "PLATO HECHO EN CASA (HC)"

ID: BCN 05

DÍA DEL CONSUMO: 11 / 10 / 2014 **HORA DEL CONSUMO:** 13:35

MÉTODO DE ELABORACIÓN:

Explicar detalladamente el proceso de elaboración del plato:

Poner agua a hervir en una olla al fuego.
Cuando el agua está hirviendo, añadir el pollo, ¼ entero, dejar cocer 5 minutos.
Añadir zanahoria troceada a rodajas finas.
Añadir el puerro troceado.
Una pizca de sal y un chorrito de aceite.
Dejar cocer de 10 a 15 minutos, quitar las impurezas que vaya soltando el caldo.
Pasado este tiempo, añadir el arroz, dejar cocer 10 minutos más.
Finalizado el tiempo, apagar el fuego, tapar la olla, dejar 3-5 minutos reposando.
Ya está listo.
Separar el pollo en trocitos pequeños, añadir al caldo con el arroz.

Fotos:

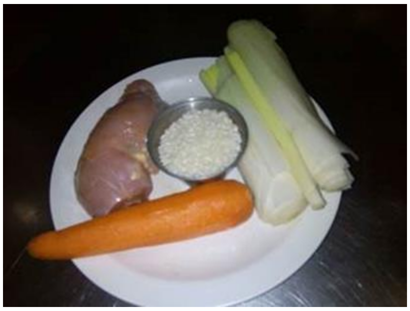

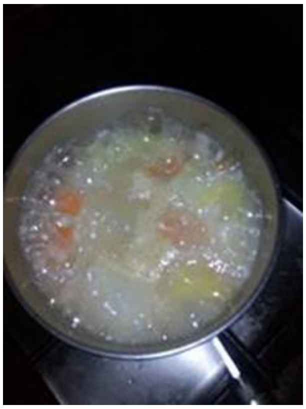

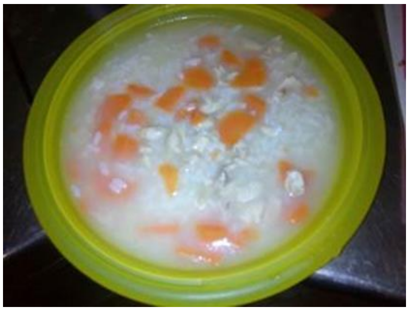

FICHA INGREDIENTES/PESOS "PLATO HECHO EN CASA (HC)" **ID: BCN 06**

DÍA DEL CONSUMO: *10 / 10 / 2014* HORA DEL CONSUMO: *13:00*

LA ELABOARACIÓN DEL PLATO SE HACE EL MISMO DÍA DEL CONSUMO ☒ Sí ☐ No

Si la fecha de elaboración del plato NO es el mismo día del consumo NO se adjuntará foto y el peso de los ingredientes puede ser aproximado. Indique la fecha de elaboración: 10/10/2014

TIPO DE PLATO: *PURÉ DE VERDURAS CON MERLUZA*

	Tipo de ingrediente	Nº	Peso (en gramos)
Ingrediente 1	*Judías*	*3*	*60 gr*
Ingrediente 2	*Patatas*	*2*	*135 gr*
Ingrediente 3	*Calabaza*	*½*	*100 gr*
Ingrediente 4	*Medio calabacín*	*½*	*175 gr*
Ingrediente 5	*Chorro de aceite de oliva*	*1*	*1 chorro*
Ingrediente 6	*Pescado*	*1*	*87 gr*
Ingrediente 7			
Ingrediente 8			
Ingrediente 9			
Ingrediente 10			

Proceso culinario	Nº	Peso (en gramos)	Observaciones
1. Olla VACÍA		*670 gr*	
2. Olla con COMIDA COCINADA		*1420 gr*	
**COMIDA COCINADA (2-1)**		*750 gr*	
3. Fiambrera VACÍA			
4. Fiambrera con COMIDA COCINADA			
**COMIDA ALMACENADA (4-3)**		*161 gr*	
5. Plato VACÍO		*77 gr*	
6. Plato con COMIDA COCINADA		*222 gr*	
**COMIDA QUE SIRVE A SU HIJO (6-5)**		*145 gr*	
7. Plato COMIDA QUE SU HIJO NO HA COMIDO		*77 gr*	
**COMIDA QUE SU HIJO HA COMIDO (6-7)**		*145 gr*	

MÉTODO DE ELABORACIÓN:
Explicar detalladamente el proceso de elaboración del plato

FICHA INGREDIENTES/PESOS "PLATO HECHO EN CASA (HC)"

ID: BCN 06

DÍA DEL CONSUMO: 10 / 10 / 2014 **HORA DEL CONSUMO:** 13:00

MÉTODO DE ELABORACIÓN:

Explicar detalladamente el proceso de elaboración del plato:

Pelar y cortar la verdura.
Trocear el pescado.
Hervirlo todo durante 30 minutos.
Añadir aceite.
Triturar todo.

Fotos:

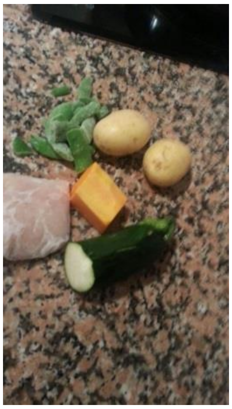

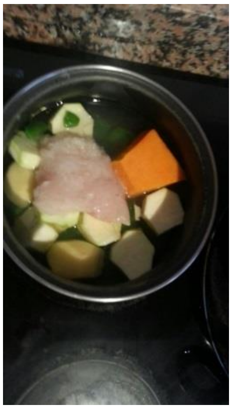

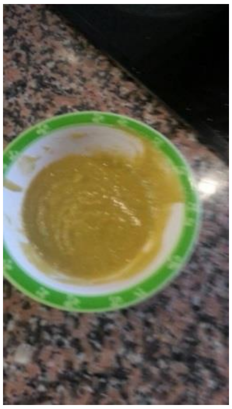

FICHA INGREDIENTES/PESOS "PLATO HECHO EN CASA (HC)" **ID: BCN 06**

DÍA DEL CONSUMO: *12 / 10 / 2014* HORA DEL CONSUMO: *13:00*

LA ELABOARACIÓN DEL PLATO SE HACE EL MISMO DÍA DEL CONSUMO [X] Sí [ ] No

Si la fecha de elaboración del plato NO es el mismo día del consumo NO se adjuntará foto y el peso de los ingredientes puede ser aproximado. Indique la fecha de elaboración: 12/10/2014

TIPO DE PLATO: *PURÉ DE VERDURAS CON TERNERA*

	Tipo de ingrediente	Nº	Peso (en gramos)
Ingrediente 1	*Ternera*		*90 gr*
Ingrediente 2	*Patata*	*2*	*350 gr*
Ingrediente 3	*Judía*	*3*	*100 gr*
Ingrediente 4	*Calabacín*	*½*	*270 gr*
Ingrediente 5	*Chorro de aceite*		
Ingrediente 6	*Calabaza*	*½*	*130 gr*
Ingrediente 7	*Tomate*	*½*	*99 gr*
Ingrediente 8			
Ingrediente 9			
Ingrediente 10			

Proceso culinario	Nº	Peso (en gramos)	Observaciones
1. Olla VACÍA		*670 gr*	
2. Olla con COMIDA COCINADA		*2660 gr*	
**COMIDA COCINADA (2-1)**		*1990 gr*	
3. Fiambrera VACÍA			
4. Fiambrera con COMIDA COCINADA			
**COMIDA ALMACENADA (4-3)**		*147 gr*	
5. Plato VACÍO		*77 gr*	
6. Plato con COMIDA COCINADA		*222 gr*	
**COMIDA QUE SIRVE A SU HIJO (6-5)**		*145 gr*	
7. Plato COMIDA QUE SU HIJO NO HA COMIDO		*77 gr*	
**COMIDA QUE SU HIJO HA COMIDO (6-7)**		*145 gr*	

MÉTODO DE ELABORACIÓN:

Explicar detalladamente el proceso de elaboración del plato

FICHA INGREDIENTES/PESOS “PLATO HECHO EN CASA (HC)” **ID: BCN 06**

DÍA DEL CONSUMO: 12 / 10 / 2014 **HORA DEL CONSUMO:** 13:00

MÉTODO DE ELABORACIÓN:
Explicar detalladamente el proceso de elaboración del plato:

Pelar y cortar la verdura.
Poner el trozo de ternera.
Hervirlo todo durante 30 minutos.
Añadir aceite.
Triturar todo.

Fotos:

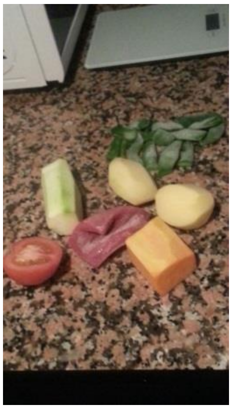

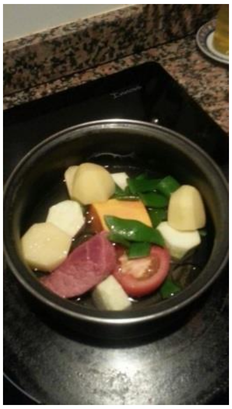

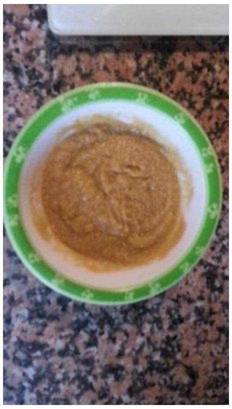

FICHA INGREDIENTES/PESOS "PLATO HECHO EN CASA (HC)" **ID: BCN 06**

DÍA DEL CONSUMO: *16 / 10 / 2014* HORA DEL CONSUMO: *13:30*

LA ELABOARACIÓN DEL PLATO SE HACE EL MISMO DÍA DEL CONSUMO [X] Sí [ ] No

Si la fecha de elaboración del plato NO es el mismo día del consumo NO se adjuntará foto y el peso de los ingredientes puede ser aproximado. Indique la fecha de elaboración: 16/10/2014

TIPO DE PLATO: *PURÉ DE VERDURAS CON POLLO*

	Tipo de ingrediente	Nº	Peso (en gramos)
Ingrediente 1	*Pollo*	*1 trozo*	*37 gr*
Ingrediente 2	*Judía*	*2*	*50 gr*
Ingrediente 3	*Patata*	*1*	*130 gr*
Ingrediente 4	*Puerro*	*½*	*37 gr*
Ingrediente 5	*Calabaza*	*½*	*30 gr*
Ingrediente 6	*Zanahoria*	*½*	*37 gr*
Ingrediente 7	*Aceite de oliva*	*1 chorrito*	
Ingrediente 8			
Ingrediente 9			
Ingrediente 10			

Proceso culinario	Nº	Peso (en gramos)	Observaciones
1. Olla VACÍA		*669 gr*	
2. Olla con COMIDA COCINADA		*1313 gr*	
**COMIDA COCINADA (2-1)**		*644 gr*	
3. Fiambrera VACÍA			
4. Fiambrera con COMIDA COCINADA			
**COMIDA ALMACENADA (4-3)**			
5. Plato VACÍO		*83 gr*	
6. Plato con COMIDA COCINADA		*198 gr*	
**COMIDA QUE SIRVE A SU HIJO (6-5)**		*115 gr*	
7. Plato COMIDA QUE SU HIJO NO HA COMIDO		*83 gr*	
**COMIDA QUE SU HIJO HA COMIDO (6-7)**		*115 gr*	

MÉTODO DE ELABORACIÓN:
Explicar detalladamente el proceso de elaboración del plato

FICHA INGREDIENTES/PESOS "PLATO HECHO EN CASA (HC)" **ID: BCN 06**

DÍA DEL CONSUMO: 16 / 10 / 2014 **HORA DEL CONSUMO:** 13:30

MÉTODO DE ELABORACIÓN:

Explicar detalladamente el proceso de elaboración del plato:

Pelar y cortas las verduras.
Trocear el pollo.
Hervir durante 30 minutos.
Añadir aceite.
Triturar.

Fotos:

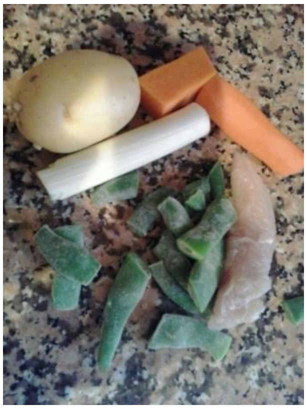

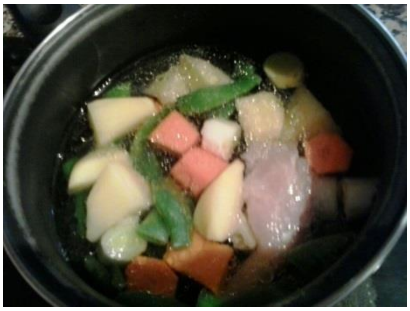

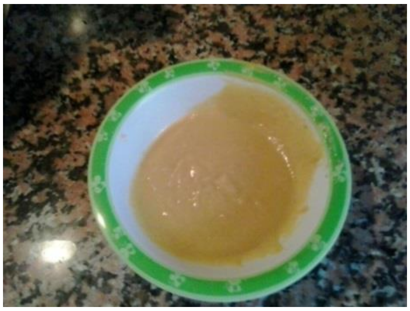

FICHA INGREDIENTES/PESOS “PLATO HECHO EN CASA (HC)” **ID: BCN 06**

DÍA DEL CONSUMO: *16 / 10 / 2014* HORA DEL CONSUMO: *21:00*

LA ELABOARACIÓN DEL PLATO SE HACE EL MISMO DÍA DEL CONSUMO [X] Sí [ ] No

Si la fecha de elaboración del plato NO es el mismo día del consumo NO se adjuntará foto y el peso de los ingredientes puede ser aproximado. Indique la fecha de elaboración: Hecho a medio día

TIPO DE PLATO: *CALDO DE POLLO*

	Tipo de ingrediente	Nº	Peso (en gramos)
Ingrediente 1	*Trozo de pechuga*	*2*	*90 gr*
Ingrediente 2	*Muslo de pollo*	*2*	*420 gr*
Ingrediente 3	*Zanahoria*	*6*	*700 gr*
Ingrediente 4	*Hueso blanco*	*2*	*240 gr*
Ingrediente 5	*Apio*	*1*	*35 gr*
Ingrediente 6	*Puerro*	*1*	*100 gr*
Ingrediente 7	*Nabo*	*1*	*45 gr*
Ingrediente 8	*Chirivía*	*1*	*190 gr*
Ingrediente 9			
Ingrediente 10			

Proceso culinario	Nº	Peso (en gramos)	Observaciones
1. Olla VACÍA		*2000 gr*	
2. Olla con COMIDA COCINADA		*5000 gr*	
**COMIDA COCINADA (2-1)**		*3000 gr*	
3. Fiambrera VACÍA			
4. Fiambrera con COMIDA COCINADA			
**COMIDA ALMACENADA (4-3)**			
5. Plato VACÍO		*58 gr*	
6. Plato con COMIDA COCINADA		*184 gr*	
**COMIDA QUE SIRVE A SU HIJO (6-5)**		*126 gr*	
7. Plato COMIDA QUE SU HIJO NO HA COMIDO		*84 gr*	
**COMIDA QUE SU HIJO HA COMIDO (6-7)**		*100 gr*	

MÉTODO DE ELABORACIÓN:

Explicar detalladamente el proceso de elaboración del plato

FICHA INGREDIENTES/PESOS “PLATO HECHO EN CASA (HC)” **ID: BCN 06**

DÍA DEL CONSUMO: 16 / 10 / 2014 **HORA DEL CONSUMO:** 21:00

MÉTODO DE ELABORACIÓN:
Explicar detalladamente el proceso de elaboración del plato:

Lavar y cortar la verdura.
Añadir agua.
Poner el pollo y los huesos blancos.
Lo hiervo durante 3 horas a fuego lento.
Cuando está lo cuelo, le pongo la sopa y el resto lo congelo.

Fotos:

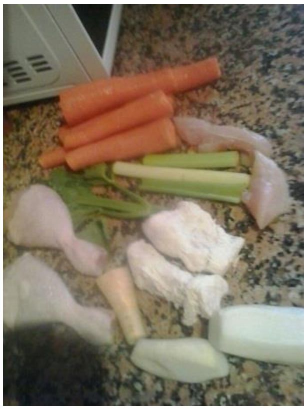

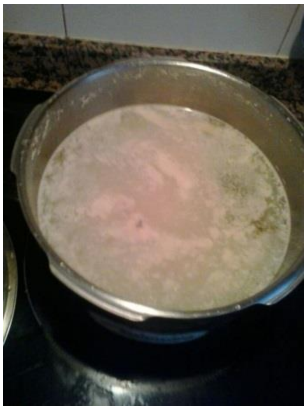

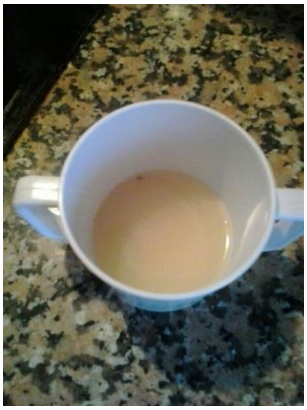

FICHA INGREDIENTES/PESOS "PLATO HECHO EN CASA (HC)" **ID: BCN 07**

DÍA DEL CONSUMO: *11 / 10 / 2014* HORA DEL CONSUMO: *14:30*

LA ELABOARACIÓN DEL PLATO SE HACE EL MISMO DÍA DEL CONSUMO [X] Sí [ ] No

Si la fecha de elaboración del plato NO es el mismo día del consumo NO se adjuntará foto y el peso de los ingredientes puede ser aproximado. Indique la fecha de elaboración: 11/10/2014

TIPO DE PLATO: *VERDURAS CON POLLO*

	Tipo de ingrediente	Nº	Peso (en gramos)
Ingrediente 1	*Calabaza*		*45 gr*
Ingrediente 2	*Calabacín*		*59 gr*
Ingrediente 3	*Pollo*		*50 gr*
Ingrediente 4	*Aceite*		*1 chorro*
Ingrediente 5	*Leche materna*		*1 chorro*
Ingrediente 6			
Ingrediente 7			
Ingrediente 8			
Ingrediente 9			
Ingrediente 10			

Proceso culinario	Nº	Peso (en gramos)	Observaciones
1. Olla VACÍA		*438 gr*	
2. Olla con COMIDA COCINADA		*966 gr*	
**COMIDA COCINADA (2-1)**		*528 gr*	
3. Fiambrera VACÍA			
4. Fiambrera con COMIDA COCINADA			
**COMIDA ALMACENADA (4-3)**			
5. Plato VACÍO		*349 gr*	
6. Plato con COMIDA COCINADA		*482 gr*	
**COMIDA QUE SIRVE A SU HIJO (6-5)**		*133 gr*	
7. Plato COMIDA QUE SU HIJO NO HA COMIDO		*349 gr*	
**COMIDA QUE SU HIJO HA COMIDO (6-7)**		*133 gr*	

MÉTODO DE ELABORACIÓN:

Explicar detalladamente el proceso de elaboración del plato

FICHA INGREDIENTES/PESOS "PLATO HECHO EN CASA (HC)"

ID: BCN 07

DÍA DEL CONSUMO: 11 / 10 / 2014 **HORA DEL CONSUMO:** 14:30

MÉTODO DE ELABORACIÓN:

Explicar detalladamente el proceso de elaboración del plato:

Pelar el trozo de calabaza.
Trocear en dos porciones el calabacín (con piel).
Hervir agua en un cazo.
Añadir al cabo el calabacín.
Añadir la calabaza (a media cocción del calabacín).
Añadir el pollo (cuando queden unos dos minutos de cocción).
Trocear los ingredientes y triturar.
Añadir un chorro de aceite y otro de leche materna.

Fotos:

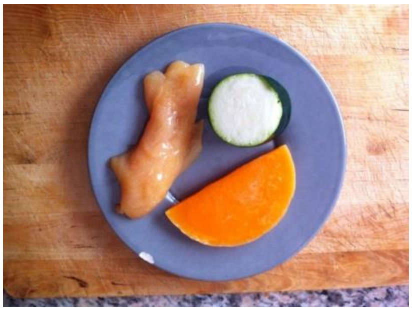

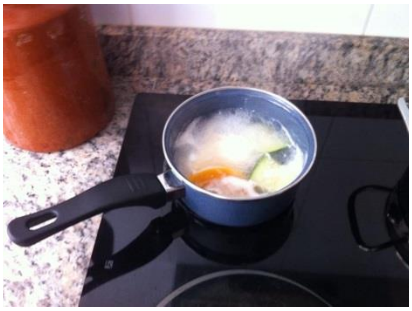

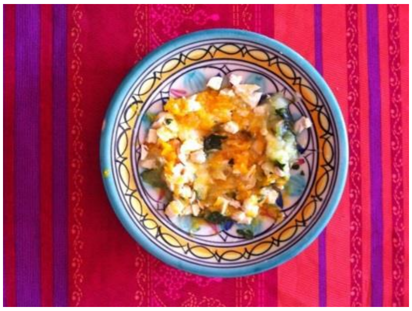

FICHA INGREDIENTES/PESOS "PLATO HECHO EN CASA (HC)" **ID: BCN 07**

DÍA DEL CONSUMO: *13 / 10 / 2014* HORA DEL CONSUMO: *14:50*

LA ELABOARACIÓN DEL PLATO SE HACE EL MISMO DÍA DEL CONSUMO [X] Sí [ ] No

Si la fecha de elaboración del plato NO es el mismo día del consumo NO se adjuntará foto y el peso de los ingredientes puede ser aproximado. Indique la fecha de elaboración: 13/10/2014

TIPO DE PLATO: *BRÓCOLI CON PATATA*

	Tipo de ingrediente	Nº	Peso (en gramos)
Ingrediente 1	*Brócoli*		*99 gr*
Ingrediente 2	*Patata*		*89 gr*
Ingrediente 3	*Aceite*		*1 chorro*
Ingrediente 4	*Leche materna*		*1 chorro*
Ingrediente 5			
Ingrediente 6			
Ingrediente 7			
Ingrediente 8			
Ingrediente 9			
Ingrediente 10			

Proceso culinario	Nº	Peso (en gramos)	Observaciones
1. Olla VACÍA		*438 gr*	
2. Olla con COMIDA COCINADA		*921 gr*	
**COMIDA COCINADA (2-1)**		*483 gr*	
3. Fiambrera VACÍA			
4. Fiambrera con COMIDA COCINADA			
**COMIDA ALMACENADA (4-3)**			
5. Plato VACÍO		*349 gr*	
6. Plato con COMIDA COCINADA		*521 gr*	
**COMIDA QUE SIRVE A SU HIJO (6-5)**		*172 gr*	
7. Plato COMIDA QUE SU HIJO NO HA COMIDO		*390 gr*	
**COMIDA QUE SU HIJO HA COMIDO (6-7)**		*131 gr*	

MÉTODO DE ELABORACIÓN:
Explicar detalladamente el proceso de elaboración del plato

FICHA INGREDIENTES/PESOS "PLATO HECHO EN CASA (HC)"

ID: BCN 07

DÍA DEL CONSUMO: 13 / 10 / 2014 **HORA DEL CONSUMO:** 14:50

MÉTODO DE ELABORACIÓN:

Explicar detalladamente el proceso de elaboración del plato:

Pelar la patata.
Trocear la patata.
Separar varios troncos de brócoli.
Hervir agua en un cazo.
Añadir las verduras al agua hirviendo.
Añadir un chorro de aceite y otro de leche materna.

Fotos:

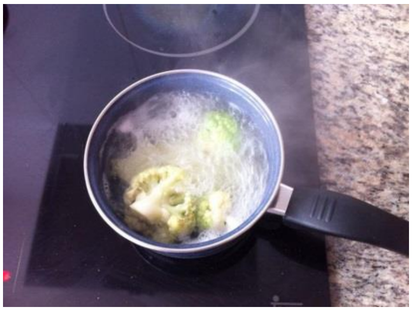

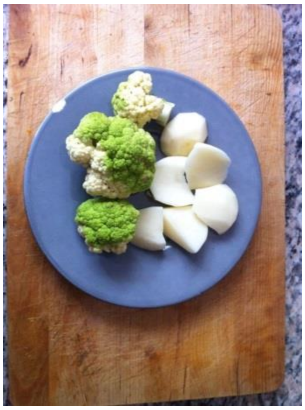

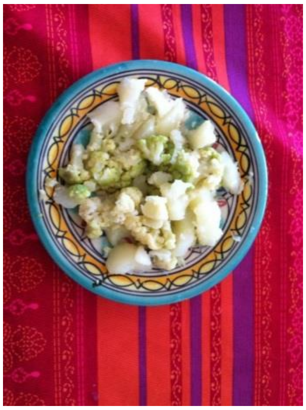

FICHA INGREDIENTES/PESOS "PLATO HECHO EN CASA (HC)" **ID: BCN 07**

DÍA DEL CONSUMO: *13 / 10 / 2014* HORA DEL CONSUMO: *18:30*

LA ELABOARACIÓN DEL PLATO SE HACE EL MISMO DÍA DEL CONSUMO [X] Sí [ ] No

Si la fecha de elaboración del plato NO es el mismo día del consumo NO se adjuntará foto y el peso de los ingredientes puede ser aproximado. Indique la fecha de elaboración: 13/10/2014

TIPO DE PLATO: *PAPILLA DE FRUTAS*

	Tipo de ingrediente	Nº	Peso (en gramos)
Ingrediente 1	*Plátano*	*1*	*83 gr*
Ingrediente 2	*Pera*	*1*	*103 gr*
Ingrediente 3	*Naranja*	*½*	*70 ml (zumo)*
Ingrediente 4			
Ingrediente 5			
Ingrediente 6			
Ingrediente 7			
Ingrediente 8			
Ingrediente 9			
Ingrediente 10			

Proceso culinario	Nº	Peso (en gramos)	Observaciones
1. Olla VACÍA			
2. Olla con COMIDA COCINADA			
**COMIDA COCINADA (2-1)**			
3. Fiambrera VACÍA			
4. Fiambrera con COMIDA COCINADA			
**COMIDA ALMACENADA (4-3)**			
5. Plato VACÍO		*91 gr*	
6. Plato con COMIDA COCINADA		*243 gr*	
**COMIDA QUE SIRVE A SU HIJO (6-5)**		*152 gr*	
7. Plato COMIDA QUE SU HIJO NO HA COMIDO		*162 gr*	
**COMIDA QUE SU HIJO HA COMIDO (6-7)**		*81 gr*	

MÉTODO DE ELABORACIÓN:
Explicar detalladamente el proceso de elaboración del plato

FICHA INGREDIENTES/PESOS "PLATO HECHO EN CASA (HC)"

ID: BCN 07

DÍA DEL CONSUMO: 13 / 10 / 2014 **HORA DEL CONSUMO:** 18:30

MÉTODO DE ELABORACIÓN:

Explicar detalladamente el proceso de elaboración del plato:

Pelar las frutas y trocearlas.
Exprimir media naranja.
Triturarlo todo.

Fotos:

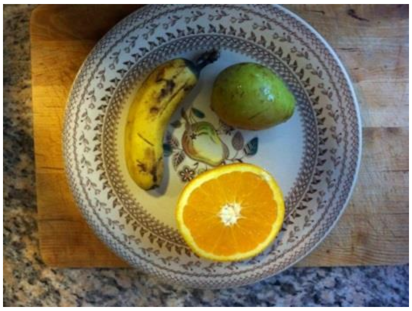

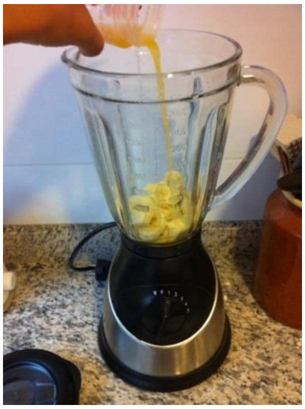

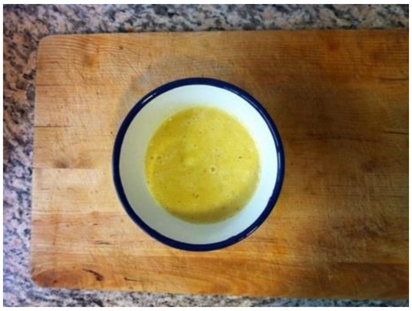

FICHA INGREDIENTES/PESOS "PLATO HECHO EN CASA (HC)" **ID: BCN 07**

DÍA DEL CONSUMO: *14 / 10 / 2014* HORA DEL CONSUMO: *14:40*

LA ELABOARACIÓN DEL PLATO SE HACE EL MISMO DÍA DEL CONSUMO [X] Sí [ ] No

Si la fecha de elaboración del plato NO es el mismo día del consumo NO se adjuntará foto y el peso de los ingredientes puede ser aproximado. Indique la fecha de elaboración: 14/10/2014

TIPO DE PLATO: *ARROZ CON CALABAZA Y TERNERA*

	Tipo de ingrediente	Nº	Peso (en gramos)
Ingrediente 1	*Arroz*		*15 gr*
Ingrediente 2	*Calabaza*		*77 gr*
Ingrediente 3	*Ternera*		*43 gr*
Ingrediente 4	*Aceite*		*1 chorrito*
Ingrediente 5			
Ingrediente 6			
Ingrediente 7			
Ingrediente 8			
Ingrediente 9			
Ingrediente 10			

Proceso culinario	Nº	Peso (en gramos)	Observaciones
1. Olla VACÍA		*438 gr*	
2. Olla con COMIDA COCINADA		*850 gr*	
**COMIDA COCINADA (2-1)**		*412 gr*	
3. Fiambrera VACÍA			
4. Fiambrera con COMIDA COCINADA			
**COMIDA ALMACENADA (4-3)**			
5. Plato VACÍO		*349 gr*	
6. Plato con COMIDA COCINADA		*490 gr*	
**COMIDA QUE SIRVE A SU HIJO (6-5)**		*141 gr*	
7. Plato COMIDA QUE SU HIJO NO HA COMIDO		*349 gr*	
**COMIDA QUE SU HIJO HA COMIDO (6-7)**		*141 gr*	

MÉTODO DE ELABORACIÓN:
Explicar detalladamente el proceso de elaboración del plato

FICHA INGREDIENTES/PESOS "PLATO HECHO EN CASA (HC)" **ID: BCN 07**

DÍA DEL CONSUMO: 14 / 10 / 2014 **HORA DEL CONSUMO:** 14:40

MÉTODO DE ELABORACIÓN:
Explicar detalladamente el proceso de elaboración del plato:

Pelar la calabaza.
Hervir agua en un cazo.
Añadir el arroz.
Añadir la calabaza.
Añadir la ternera (a pocos minutos de acabar la cocción).
Trocear los ingredientes y triturarlos.
Añadir chorro de aceite.

Fotos:

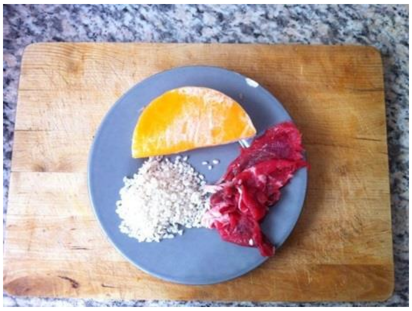

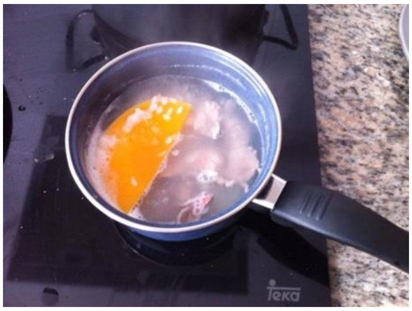

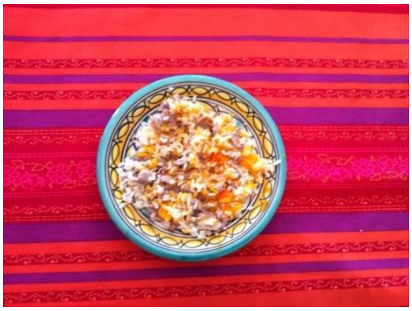

FICHA INGREDIENTES/PESOS "PLATO HECHO EN CASA (HC)" **ID: BCN 08**

DÍA DEL CONSUMO: *12 / 10 / 2014* HORA DEL CONSUMO: *17:00*

LA ELABOARACIÓN DEL PLATO SE HACE EL MISMO DÍA DEL CONSUMO ☒ Sí ☐ No

Si la fecha de elaboración del plato NO es el mismo día del consumo NO se adjuntará foto y el peso de los ingredientes puede ser aproximado. Indique la fecha de elaboración: 12/10/2014

TIPO DE PLATO: *PAPILLA DE FRUTAS*

	Tipo de ingrediente	Nº	Peso (en gramos)
Ingrediente 1	*Pera*	*1*	*244 gr*
Ingrediente 2	*Manzana*	*1*	*263 gr*
Ingrediente 3	*Naranja (*)*	*1*	*18 g (chorro)*
Ingrediente 4			
Ingrediente 5			
Ingrediente 6			
Ingrediente 7			
Ingrediente 8			
Ingrediente 9			
Ingrediente 10			

Proceso culinario	Nº	Peso (en gramos)	Observaciones
1. Olla VACÍA			
2. Olla con COMIDA COCINADA			
**COMIDA COCINADA (2-1)**			
3. Fiambrera VACÍA			
4. Fiambrera con COMIDA COCINADA			
**COMIDA ALMACENADA (4-3)**			
5. Plato VACÍO		*56 gr*	
6. Plato con COMIDA COCINADA		*252 gr*	
**COMIDA QUE SIRVE A SU HIJO (6-5)**		*196 gr*	*La muestra a parte*
7. Plato COMIDA QUE SU HIJO NO HA COMIDO		*149 gr*	
**COMIDA QUE SU HIJO HA COMIDO (6-7)**		*103 gr*	*Después leche materna*

MÉTODO DE ELABORACIÓN:
Explicar detalladamente el proceso de elaboración del plato

FICHA INGREDIENTES/PESOS "PLATO HECHO EN CASA (HC)" **ID: BCN 08**

DÍA DEL CONSUMO: 12 / 10 / 2014 **HORA DEL CONSUMO:** 17:00

MÉTODO DE ELABORACIÓN:
Explicar detalladamente el proceso de elaboración del plato:

Fruta del tiempo: naranja, manzana y pera.
Pelar la fruta.
Lavar.
Cortar.
Triturar.

(*) La naranja exprimida.

Fotos:

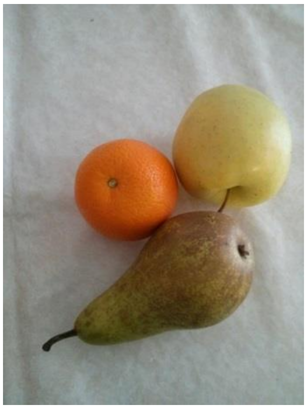

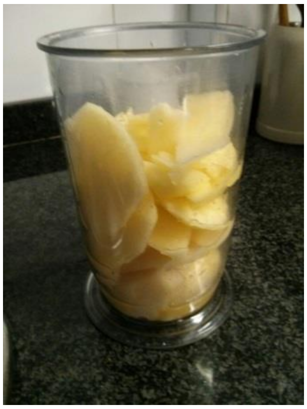

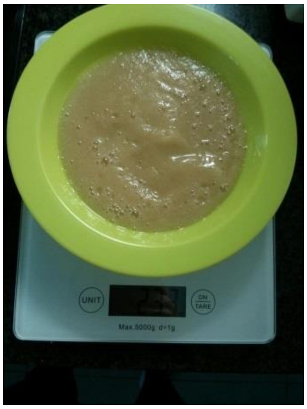

FICHA INGREDIENTES/PESOS “PLATO HECHO EN CASA (HC)” **ID: BCN 08**

DÍA DEL CONSUMO: *12 / 10 / 2014* HORA DEL CONSUMO: *21:00*

LA ELABOARACIÓN DEL PLATO SE HACE EL MISMO DÍA DEL CONSUMO ☒ Sí ☐ No

Si la fecha de elaboración del plato NO es el mismo día del consumo NO se adjuntará foto y el peso de los ingredientes puede ser aproximado. Indique la fecha de elaboración: 12/10/2014

TIPO DE PLATO: *PAPILLA DE CEREALES*

	Tipo de ingrediente	Nº	Peso (en gramos)
Ingrediente 1	*Agua*		*150 ml*
Ingrediente 2	*Leche en polvo*		*3 cazos*
Ingrediente 3	*Cereales*		*2 cazos*
Ingrediente 4			
Ingrediente 5			
Ingrediente 6			
Ingrediente 7			
Ingrediente 8			
Ingrediente 9			
Ingrediente 10			

Proceso culinario	Nº	Peso (en gramos)	Observaciones
1. Olla VACÍA			
2. Olla con COMIDA COCINADA			
**COMIDA COCINADA (2-1)**			
3. Fiambrera VACÍA			
4. Fiambrera con COMIDA COCINADA			
**COMIDA ALMACENADA (4-3)**			
5. Plato VACÍO		*56 gr*	
6. Plato con COMIDA COCINADA		*206 gr*	
**COMIDA QUE SIRVE A SU HIJO (6-5)**		*150 gr*	
7. Plato COMIDA QUE SU HIJO NO HA COMIDO		*56 gr*	
**COMIDA QUE SU HIJO HA COMIDO (6-7)**		*150 gr*	

MÉTODO DE ELABORACIÓN:
Explicar detalladamente el proceso de elaboración del plato

FICHA INGREDIENTES/PESOS “PLATO HECHO EN CASA (HC)” **ID: BCN 08**

DÍA DEL CONSUMO: 12 / 10 / 2014 **HORA DEL CONSUMO:** 21:00

MÉTODO DE ELABORACIÓN:

Explicar detalladamente el proceso de elaboración del plato:

Caliento el agua en el microondas.
Le añado los 3 cacitos de lecha y los 2 de cereales en el plato.
Lo mezclo.

(*) Las medias las pongo en mililitros y no en gramos porque me baso en las medidas líquidas del biberón, para calcular cuanto toma y cuanto se deja (aunque se lo administro en el plato).

Fotos:

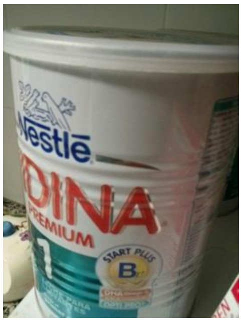

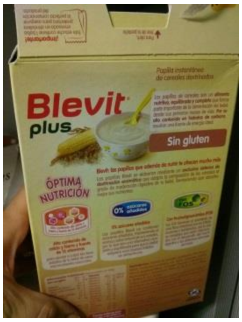

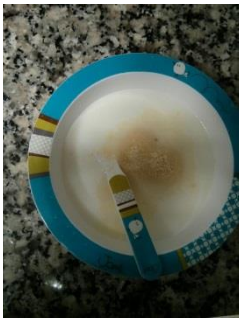

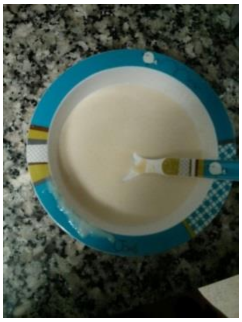

FICHA INGREDIENTES/PESOS "PLATO HECHO EN CASA (HC)" **ID: BCN 08**

DÍA DEL CONSUMO: *13 / 10 / 2014* HORA DEL CONSUMO: *17:00*

LA ELABOARACIÓN DEL PLATO SE HACE EL MISMO DÍA DEL CONSUMO [X] Sí [ ] No

Si la fecha de elaboración del plato NO es el mismo día del consumo NO se adjuntará foto y el peso de los ingredientes puede ser aproximado. Indique la fecha de elaboración: 13/10/2014

TIPO DE PLATO: *PAPILLA DE FRUTAS*

	Tipo de ingrediente	Nº	Peso (en gramos)
Ingrediente 1	*Manzana*	*1*	*220 gr*
Ingrediente 2	*Pera*	*1*	*230 gr*
Ingrediente 3	*Naranja (*)*	*1*	*195 gr*
Ingrediente 4			
Ingrediente 5			
Ingrediente 6			
Ingrediente 7			
Ingrediente 8			
Ingrediente 9			
Ingrediente 10			

Proceso culinario	Nº	Peso (en gramos)	Observaciones
1. Olla VACÍA			
2. Olla con COMIDA COCINADA			
**COMIDA COCINADA (2-1)**			
3. Fiambrera VACÍA			
4. Fiambrera con COMIDA COCINADA			
**COMIDA ALMACENADA (4-3)**			
5. Plato VACÍO		*56 gr*	
6. Plato con COMIDA COCINADA		*266 gr*	
**COMIDA QUE SIRVE A SU HIJO (6-5)**		*210 gr*	*La muestra a parte*
7. Plato COMIDA QUE SU HIJO NO HA COMIDO		*115 gr*	
**COMIDA QUE SU HIJO HA COMIDO (6-7)**		*151 gr*	*Después leche materna*

MÉTODO DE ELABORACIÓN:
Explicar detalladamente el proceso de elaboración del plato

FICHA INGREDIENTES/PESOS "PLATO HECHO EN CASA (HC)" **ID: BCN 08**

DÍA DEL CONSUMO: 13 / 10 / 2014 **HORA DEL CONSUMO:** 17:00

MÉTODO DE ELABORACIÓN:
Explicar detalladamente el proceso de elaboración del plato:

Pelar la fruta del tiempo.
Cortar la fruta.
Triturar.
(*) La naranja exprimida.

Fotos:

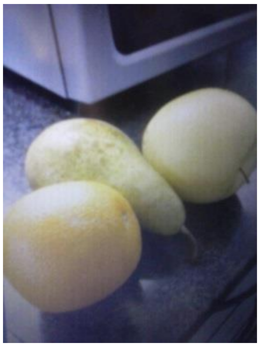

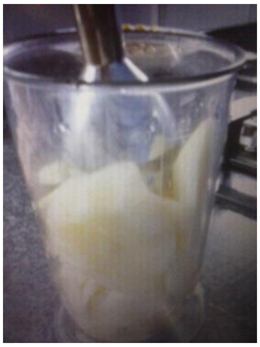

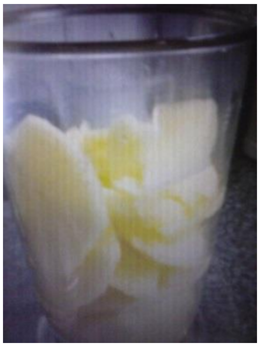

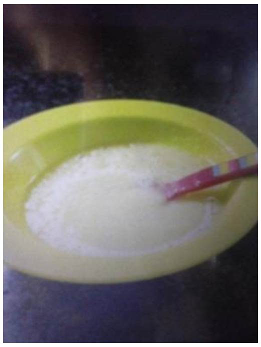

FICHA INGREDIENTES/PESOS "PLATO HECHO EN CASA (HC)" **ID: BCN 08**

DÍA DEL CONSUMO: *14 / 10 / 2014* HORA DEL CONSUMO: *17:15*

LA ELABOARACIÓN DEL PLATO SE HACE EL MISMO DÍA DEL CONSUMO ☒ Sí ☐ No

Si la fecha de elaboración del plato NO es el mismo día del consumo NO se adjuntará foto y el peso de los ingredientes puede ser aproximado. Indique la fecha de elaboración: 14/10/2014

TIPO DE PLATO: *PAPILLA DE FRUTAS*

	Tipo de ingrediente	Nº	Peso (en gramos)
Ingrediente 1	*Plátano*	*½*	*60 gr*
Ingrediente 2	*Manzana*	*1*	*230 gr*
Ingrediente 3	*Pera*	*1*	*233 gr*
Ingrediente 4	*Naranja (*)*	*1/3*	*140 gr*
Ingrediente 5			
Ingrediente 6			
Ingrediente 7			
Ingrediente 8			
Ingrediente 9			
Ingrediente 10			

Proceso culinario	Nº	Peso (en gramos)	Observaciones
1. Olla VACÍA			
2. Olla con COMIDA COCINADA			
**COMIDA COCINADA (2-1)**			
3. Fiambrera VACÍA			
4. Fiambrera con COMIDA COCINADA			
**COMIDA ALMACENADA (4-3)**			
5. Plato VACÍO		*56 gr*	
6. Plato con COMIDA COCINADA		*289 gr*	
**COMIDA QUE SIRVE A SU HIJO (6-5)**		*233 gr*	*Las muestras a parte*
7. Plato COMIDA QUE SU HIJO NO HA COMIDO		*167 gr*	
**COMIDA QUE SU HIJO HA COMIDO (6-7)**		*122 gr*	*Después leche materna*

MÉTODO DE ELABORACIÓN:
Explicar detalladamente el proceso de elaboración del plato

FICHA INGREDIENTES/PESOS "PLATO HECHO EN CASA (HC)"

ID: BCN 08

DÍA DEL CONSUMO: 14 / 10 / 2014 **HORA DEL CONSUMO:** 17:15

MÉTODO DE ELABORACIÓN:

Explicar detalladamente el proceso de elaboración del plato:

Pelar la fruta del tiempo.
Lavar.
Cortar la fruta.
Triturar.
Mezclar.

(*) La naranja exprimida.

Fotos:

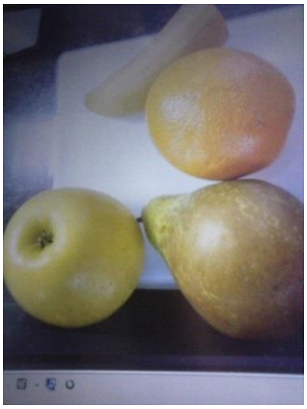

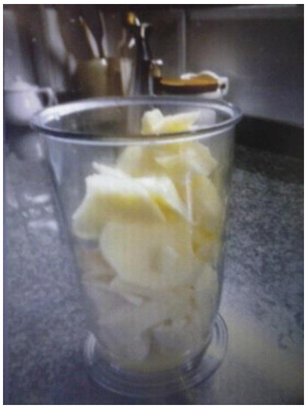

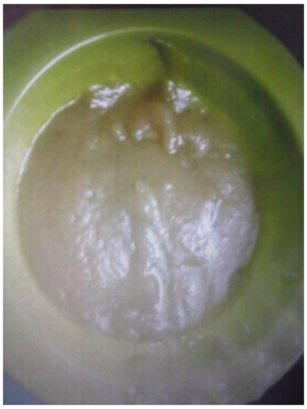

FICHA INGREDIENTES/PESOS “PLATO HECHO EN CASA (HC)” **ID: MAD 01**

DÍA DEL CONSUMO: *11 / 10 / 2014* HORA DEL CONSUMO: *12:30*

LA ELABOARACIÓN DEL PLATO SE HACE EL MISMO DÍA DEL CONSUMO [X] Sí [ ] No

Si la fecha de elaboración del plato NO es el mismo día del consumo NO se adjuntará foto y el peso de los ingredientes puede ser aproximado. Indique la fecha de elaboración:

TIPO DE PLATO: *PURÉ DE VERDURAS CON MERLUZA*

	Tipo de ingrediente	Nº	Peso (en gramos)
Ingrediente 1	*Patata*	*1*	*340 gr*
Ingrediente 2	*Judía verde*	*4*	*55 gr*
Ingrediente 3	*Zanahoria*	*1*	*91 gr*
Ingrediente 4	*Calabacín*	*½*	*140 gr*
Ingrediente 5	*Puerro*		*40 gr*
Ingrediente 6	*Merluza congelada*	*1 cola*	*187 gr*
Ingrediente 7	*Aceite*	*2 cucharadas*	
Ingrediente 8			
Ingrediente 9			
Ingrediente 10			

Proceso culinario	Nº	Peso (en gramos)	Observaciones
1. Olla VACÍA		*915 gr*	
2. Olla con COMIDA COCINADA		*1839 gr*	
**COMIDA COCINADA (2-1)**		*924 gr*	
3. Fiambrera VACÍA	*1*	*333 gr*	
4. Fiambrera con COMIDA COCINADA	*1*	*761 gr*	
**COMIDA ALMACENADA (4-3)**		*428 gr*	
5. Plato VACÍO		*35 gr*	
6. Plato con COMIDA COCINADA		*375 gr*	
**COMIDA QUE SIRVE A SU HIJO (6-5)**		*340 gr*	
7. Plato COMIDA QUE SU HIJO NO HA COMIDO		*55 gr*	
**COMIDA QUE SU HIJO HA COMIDO (6-7)**		*320 gr*	

MÉTODO DE ELABORACIÓN:
Explicar detalladamente el proceso de elaboración del plato

FICHA INGREDIENTES/PESOS "PLATO HECHO EN CASA (HC)" **ID: MAD 01**

DÍA DEL CONSUMO: 11 / 10 / 2014 **HORA DEL CONSUMO:** 12:30

MÉTODO DE ELABORACIÓN:
Explicar detalladamente el proceso de elaboración del plato:

Pelo: la patata, judía verde (quito el borde), zanahoria, medio calabacín y puerro (quito la última capa).
Lo echo en la cazuela cortado en trozos.
Cubro de agua.
Cuezo en 50 minutos.
Trituro con 2 cucharadas de postre de aceite.
Reparto.

Fotos:

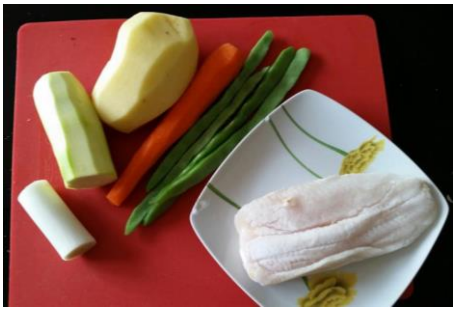

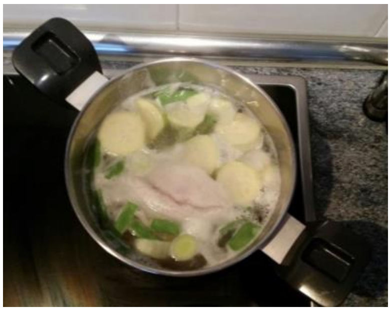

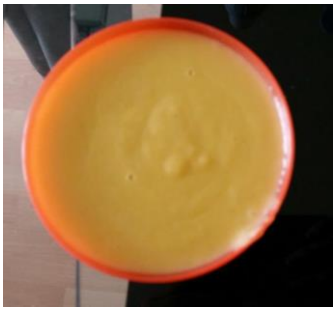

FICHA INGREDIENTES/PESOS "PLATO HECHO EN CASA (HC)" **ID: MAD 01**

DÍA DEL CONSUMO: *11 / 10 / 2014* HORA DEL CONSUMO: *18:00*

LA ELABOARACIÓN DEL PLATO SE HACE EL MISMO DÍA DEL CONSUMO [X] Sí [ ] No

Si la fecha de elaboración del plato NO es el mismo día del consumo NO se adjuntará foto y el peso de los ingredientes puede ser aproximado. Indique la fecha de elaboración:

TIPO DE PLATO: *COMPOTA DE FRUTAS*

	Tipo de ingrediente	Nº	Peso (en gramos)
Ingrediente 1	*Pera conferencia*	*1*	*162 gr*
Ingrediente 2	*Manzana Golden*	*1*	*176 gr*
Ingrediente 3	*Plátano*	*1*	*104 gr*
Ingrediente 4	*Galleta maría*	*2*	*13 gr*
Ingrediente 5	*Queso fresco bajo en sal (Hacendado)*	*1*	*58 gr*
Ingrediente 6			
Ingrediente 7			
Ingrediente 8			
Ingrediente 9			
Ingrediente 10			

Proceso culinario	Nº	Peso (en gramos)	Observaciones
1. Olla VACÍA		*476 gr*	
2. Olla con COMIDA COCINADA		*970 gr*	
**COMIDA COCINADA (2-1)**		*494 gr*	
3. Fiambrera VACÍA	*1*	*17 gr*	
4. Fiambrera con COMIDA COCINADA	*1*	*215 gr*	
**COMIDA ALMACENADA (4-3)**		*198 gr*	
5. Plato VACÍO		*17 gr*	
6. Plato con COMIDA COCINADA		*219 gr*	
**COMIDA QUE SIRVE A SU HIJO (6-5)**		*202 gr*	
7. Plato COMIDA QUE SU HIJO NO HA COMIDO		*22 gr*	
**COMIDA QUE SU HIJO HA COMIDO (6-7)**		*197 gr*	

MÉTODO DE ELABORACIÓN:
Explicar detalladamente el proceso de elaboración del plato

FICHA INGREDIENTES/PESOS "PLATO HECHO EN CASA (HC)"

ID: MAD 01

DÍA DEL CONSUMO: 11 / 10 / 2014 **HORA DEL CONSUMO:** 18:00

MÉTODO DE ELABORACIÓN:

Explicar detalladamente el proceso de elaboración del plato:

Pelo: una manzana, una pera y un plátano.
Corto en trozos.
Hago en cazuela en su propio jugo (pera abajo).
Cuezo 10 minutos.
Echo dos galletas y un queso fresco bajo en sal (Hacendado).
Trituro.
Reparto.

Fotos:

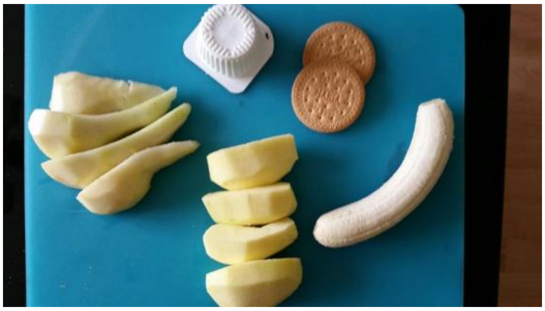

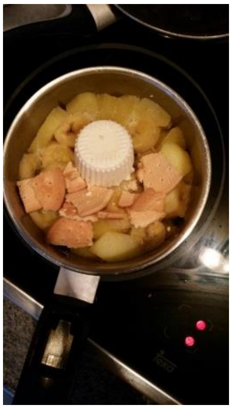

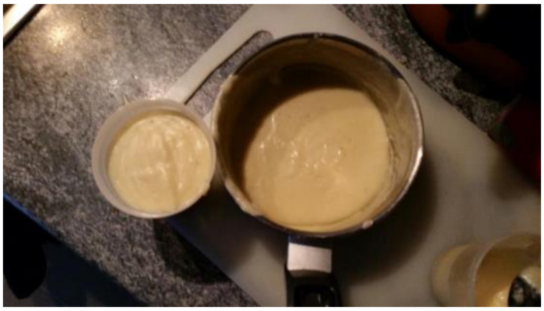

FICHA INGREDIENTES/PESOS “PLATO HECHO EN CASA (HC)” **ID: MAD 02**

DÍA DEL CONSUMO: *9 / 10 / 2014* HORA DEL CONSUMO: *13:00*

LA ELABOARACIÓN DEL PLATO SE HACE EL MISMO DÍA DEL CONSUMO ☒ Sí ☐ No

Si la fecha de elaboración del plato NO es el mismo día del consumo NO se adjuntará foto y el peso de los ingredientes puede ser aproximado. Indique la fecha de elaboración:

TIPO DE PLATO: *PURÉ DE POLLO*

	Tipo de ingrediente	Nº	Peso (en gramos)
Ingrediente 1	*Puerro*	*1*	*126 gr*
Ingrediente 2	*Acelgas*	*1*	*412 gr*
Ingrediente 3	*Calabacín*	*2*	*649 gr*
Ingrediente 4	*Patatas*	*2*	*481 gr*
Ingrediente 5	*Judías verdes*	*1*	*396 gr*
Ingrediente 6	*Zanahorias*	*4*	*380 gr*
Ingrediente 7	*Pollo (pechuga)*	*1*	*340 gr*
Ingrediente 8	*Aceite (cucharadas soperas)*	*2*	
Ingrediente 9	*Pizca de sal*	*1*	
Ingrediente 10			

Proceso culinario	Nº	Peso (en gramos)	Observaciones
1. Olla VACÍA	*1*	*1571 gr*	
2. Olla con COMIDA COCINADA	*1*	*3450 gr*	
**COMIDA COCINADA (2-1)**		*1879 gr*	
3. Fiambrera VACÍA		*22 gr*	
4. Fiambrera con COMIDA COCINADA		*297 gr*	
**COMIDA ALMACENADA (4-3)**		*275 gr*	
5. Plato VACÍO	*1*	*416 gr*	
6. Plato con COMIDA COCINADA		*686 gr*	
**COMIDA QUE SIRVE A SU HIJO (6-5)**		*270 gr*	
7. Plato COMIDA QUE SU HIJO NO HA COMIDO		*416 gr*	
**COMIDA QUE SU HIJO HA COMIDO (6-7)**		*270 gr*	

MÉTODO DE ELABORACIÓN:
Explicar detalladamente el proceso de elaboración del plato

FICHA INGREDIENTES/PESOS "PLATO HECHO EN CASA (HC)" **ID: MAD 02**

DÍA DEL CONSUMO: 9 / 10 / 2014 **HORA DEL CONSUMO:** 13:00

MÉTODO DE ELABORACIÓN:
Explicar detalladamente el proceso de elaboración del plato:

Se pelan y lavan las verduras.
Se trocean y se introducen en la olla exprés, unos 15 minutos junto con el pollo.
Se tritura con la batidora.
Se echa medio vaso de agua para la cocción.

Fotos:

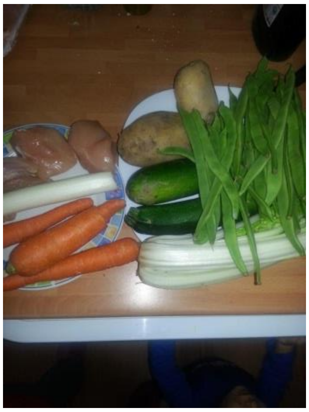

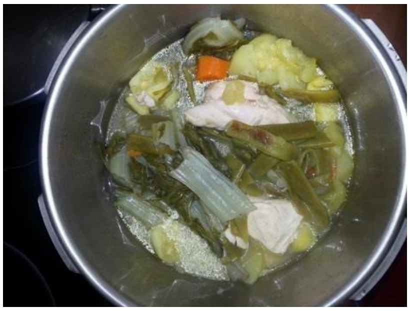

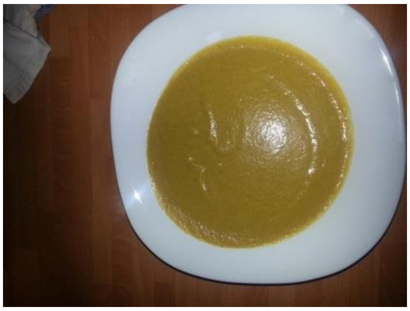

FICHA INGREDIENTES/PESOS "PLATO HECHO EN CASA (HC)" **ID: MAD 02**

DÍA DEL CONSUMO: *9 / 10 / 2014* HORA DEL CONSUMO: *16:30*

LA ELABOARACIÓN DEL PLATO SE HACE EL MISMO DÍA DEL CONSUMO [X] Sí [ ] No

Si la fecha de elaboración del plato NO es el mismo día del consumo NO se adjuntará foto y el peso de los ingredientes puede ser aproximado. Indique la fecha de elaboración:

TIPO DE PLATO: *PAPILLA DE FRUTAS*

	Tipo de ingrediente	Nº	Peso (en gramos)
Ingrediente 1	*Plátano*	*2*	*148 gr*
Ingrediente 2	*Manzana*	*1*	*212 gr*
Ingrediente 3	*Galleta María*	*1*	*6 gr*
Ingrediente 4	*Zumo de naranja*	*2*	*100 ml*
Ingrediente 5			
Ingrediente 6			
Ingrediente 7			
Ingrediente 8			
Ingrediente 9			
Ingrediente 10			

Proceso culinario	Nº	Peso (en gramos)	Observaciones
1. Olla VACÍA	*1*	*117 gr*	
2. Olla con COMIDA COCINADA		*501 gr*	
**COMIDA COCINADA (2-1)**		*384 gr*	
3. Fiambrera VACÍA			
4. Fiambrera con COMIDA COCINADA			
**COMIDA ALMACENADA (4-3)**			
5. Plato VACÍO		*245 gr*	
6. Plato con COMIDA COCINADA		*450 gr*	
**COMIDA QUE SIRVE A SU HIJO (6-5)**		*205 gr*	
7. Plato COMIDA QUE SU HIJO NO HA COMIDO		*245 gr*	
**COMIDA QUE SU HIJO HA COMIDO (6-7)**		*205 gr*	

MÉTODO DE ELABORACIÓN:
Explicar detalladamente el proceso de elaboración del plato

FICHA INGREDIENTES/PESOS “PLATO HECHO EN CASA (HC)”

ID: MAD 02

DÍA DEL CONSUMO: 9 / 10 / 2014 **HORA DEL CONSUMO:** 16:30

MÉTODO DE ELABORACIÓN:

Explicar detalladamente el proceso de elaboración del plato:

Exprimir las naranjas.
Pelar el resto de la fruta y triturar.

Fotos:

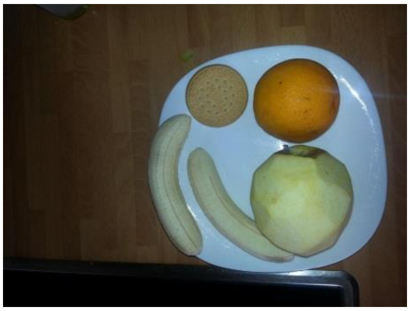

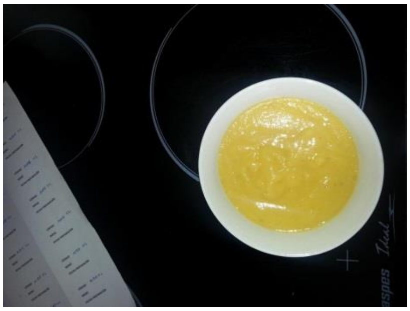

FICHA INGREDIENTES/PESOS "PLATO HECHO EN CASA (HC)" **ID: MAD 02**

DÍA DEL CONSUMO: *10 / 10 / 2014* HORA DEL CONSUMO: *17:00*

LA ELABOARACIÓN DEL PLATO SE HACE EL MISMO DÍA DEL CONSUMO [X] Sí [ ] No

Si la fecha de elaboración del plato NO es el mismo día del consumo NO se adjuntará foto y el peso de los ingredientes puede ser aproximado. Indique la fecha de elaboración:

TIPO DE PLATO: *PAPILLA DE FRUTAS*

	Tipo de ingrediente	Nº	Peso (en gramos)
Ingrediente 1	*Plátanos*	*2*	*282 gr*
Ingrediente 2	*Manzana*	*1*	*245 gr*
Ingrediente 3	*Galleta María*	*1*	*6 gr*
Ingrediente 4	*Zumo de naranja*	*2*	*98 ml*
Ingrediente 5			
Ingrediente 6			
Ingrediente 7			
Ingrediente 8			
Ingrediente 9			
Ingrediente 10			

Proceso culinario	Nº	Peso (en gramos)	Observaciones
1. Olla VACÍA	*1*	*117 gr*	
2. Olla con COMIDA COCINADA		*502 gr*	
**COMIDA COCINADA (2-1)**		*619 gr*	
3. Fiambrera VACÍA			
4. Fiambrera con COMIDA COCINADA			
**COMIDA ALMACENADA (4-3)**			
5. Plato VACÍO		*300 gr*	
6. Plato con COMIDA COCINADA		*507 gr*	
**COMIDA QUE SIRVE A SU HIJO (6-5)**		*207 gr*	
7. Plato COMIDA QUE SU HIJO NO HA COMIDO		*375 gr*	
**COMIDA QUE SU HIJO HA COMIDO (6-7)**		*132 gr*	

MÉTODO DE ELABORACIÓN:
Explicar detalladamente el proceso de elaboración del plato

FICHA INGREDIENTES/PESOS "PLATO HECHO EN CASA (HC)" **ID: MAD 02**

DÍA DEL CONSUMO: 11 / 10 / 2014 **HORA DEL CONSUMO:** 12:30

MÉTODO DE ELABORACIÓN:
Explicar detalladamente el proceso de elaboración del plato:

Exprimir las naranjas.
Se tritura la fruta y la galleta.

Fotos:

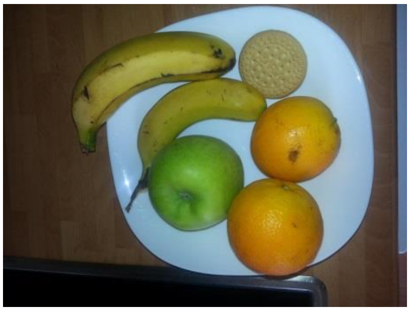

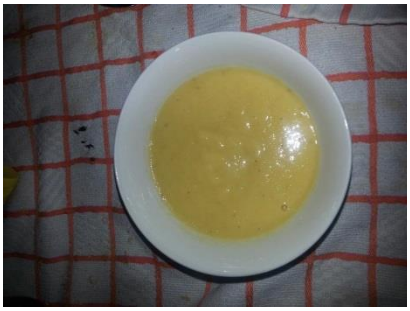

FICHA INGREDIENTES/PESOS "PLATO HECHO EN CASA (HC)" **ID: MAD 02**

DÍA DEL CONSUMO: *12 / 10 / 2014* HORA DEL CONSUMO: *13:00*

LA ELABOARACIÓN DEL PLATO SE HACE EL MISMO DÍA DEL CONSUMO [X] Sí [ ] No

Si la fecha de elaboración del plato NO es el mismo día del consumo NO se adjuntará foto y el peso de los ingredientes puede ser aproximado. Indique la fecha de elaboración:

TIPO DE PLATO: *PURÉ DE LENTEJAS*

	Tipo de ingrediente	Nº	Peso (en gramos)
Ingrediente 1	*Lentejas*		*200 gr*
Ingrediente 2	*Morcilla*	*1*	*64 gr*
Ingrediente 3	*Tocino*	*1*	*101 gr*
Ingrediente 4	*Chorizo*	*1*	*97 gr*
Ingrediente 5	*Cebolla*	*1*	*157 gr*
Ingrediente 6	*Chorro de aceite de oliva*		
Ingrediente 7	*Pizca de sal*		
Ingrediente 8			
Ingrediente 9			
Ingrediente 10			

Proceso culinario	Nº	Peso (en gramos)	Observaciones
1. Olla VACÍA	*1*	*1325 gr*	
2. Olla con COMIDA COCINADA	*1*	*3150 gr*	
**COMIDA COCINADA (2-1)**		*1825 gr*	
3. Fiambrera VACÍA		*25 gr*	
4. Fiambrera con COMIDA COCINADA		*307 gr*	
**COMIDA ALMACENADA (4-3)**		*282 gr*	
5. Plato VACÍO		*242 gr*	
6. Plato con COMIDA COCINADA		*576 gr*	
**COMIDA QUE SIRVE A SU HIJO (6-5)**		*334 gr*	
7. Plato COMIDA QUE SU HIJO NO HA COMIDO		*362 gr*	
**COMIDA QUE SU HIJO HA COMIDO (6-7)**		*214 gr*	

MÉTODO DE ELABORACIÓN:
Explicar detalladamente el proceso de elaboración del plato

FICHA INGREDIENTES/PESOS "PLATO HECHO EN CASA (HC)"

ID: MAD 02

DÍA DEL CONSUMO: 12 / 10 / 2014 **HORA DEL CONSUMO:** 13:00

MÉTODO DE ELABORACIÓN:

Explicar detalladamente el proceso de elaboración del plato:

Se pela la cebolla.
Se introducen los alimentos en la olla cubriéndolos de agua con la sal y el aceite.
Se cuece 15 minutos.
Después se tritura.

Fotos:

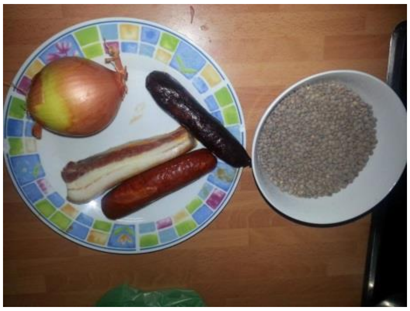

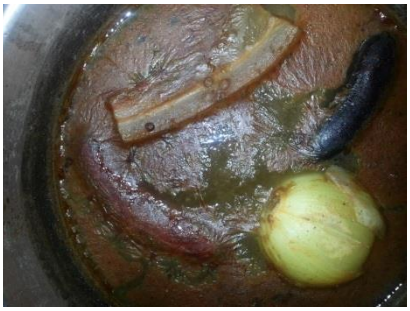

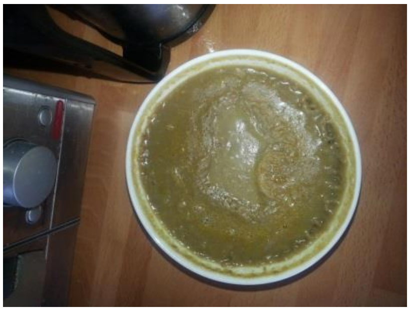

FICHA INGREDIENTES/PESOS "PLATO HECHO EN CASA (HC)" **ID: MAD 03**

DÍA DEL CONSUMO: *12 / 10 / 2014* HORA DEL CONSUMO: *21:10*

LA ELABOARACIÓN DEL PLATO SE HACE EL MISMO DÍA DEL CONSUMO [X] Sí [ ] No

Si la fecha de elaboración del plato NO es el mismo día del consumo NO se adjuntará foto y el peso de los ingredientes puede ser aproximado. Indique la fecha de elaboración:

TIPO DE PLATO: *CREMA DE VERDURAS*

	Tipo de ingrediente	Nº	Peso (en gramos)
Ingrediente 1	*Calabaza*	*½*	*600 gr*
Ingrediente 2	*Calabacín*	*1*	*547 gr*
Ingrediente 3	*Zanahoria*	*5*	*240 gr*
Ingrediente 4	*Patata*	*3*	*390 gr*
Ingrediente 5	*Puerro*	*2*	*265 gr*
Ingrediente 6	*Agua*		*1 litro*
Ingrediente 7	*Sal*	*1 pizca*	
Ingrediente 8	*Pimienta*	*1 pizca*	
Ingrediente 9	*Laurel*	*2 hojas*	
Ingrediente 10	*Leche*		*250 ml*
Ingrediente 11	*Queso de untar*		*60 gr*

Proceso culinario	Nº	Peso (en gramos)	Observaciones
1. Olla VACÍA		*1147 gr*	
2. Olla con COMIDA COCINADA		*4067 gr*	
**COMIDA COCINADA (2-1)**		*2920 gr*	
3. Fiambrera VACÍA			*Comemos todos*
4. Fiambrera con COMIDA COCINADA			
**COMIDA ALMACENADA (4-3)**			
5. Plato VACÍO		*100 gr*	
6. Plato con COMIDA COCINADA		*350 gr*	
**COMIDA QUE SIRVE A SU HIJO (6-5)**		*250 gr*	
7. Plato COMIDA QUE SU HIJO NO HA COMIDO		*110 gr*	
**COMIDA QUE SU HIJO HA COMIDO (6-7)**		*240 gr*	

MÉTODO DE ELABORACIÓN:
Explicar detalladamente el proceso de elaboración del plato

FICHA INGREDIENTES/PESOS "PLATO HECHO EN CASA (HC)"

ID: MAD 03

DÍA DEL CONSUMO: 12/ 10 / 2014 **HORA DEL CONSUMO:** 21:10

MÉTODO DE ELABORACIÓN:

Explicar detalladamente el proceso de elaboración del plato:

Se lavan y pelan las verduras (el calabacín no lo pelo, lo lavo bien), y se pican todas en la cacerola.
Añado 1 litro de agua, sal, laurel y pimienta negra molida, se pone a hervir.
A la media hora de hervir, añadimos el queso de untar y la leche.
Dejamos cocer unos 5 minutos más.
A partamos del fuego y batimos con la batidora.
Listo para servir.

Fotos:

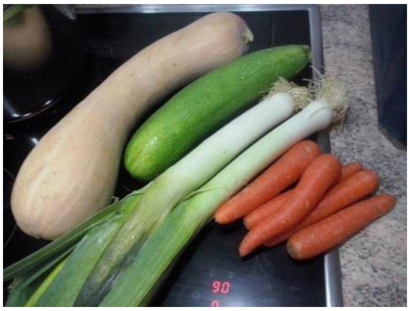

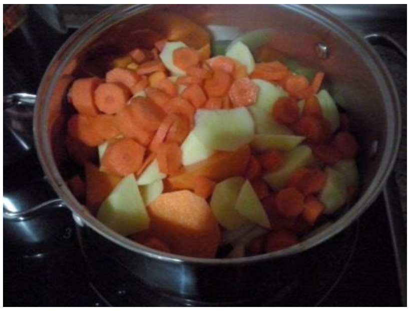

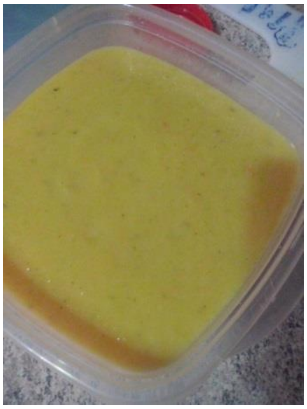

FICHA INGREDIENTES/PESOS “PLATO HECHO EN CASA (HC)” **ID: MAD 04**

DÍA DEL CONSUMO: *12 / 10 / 2014* HORA DEL CONSUMO: *13:30*

LA ELABOARACIÓN DEL PLATO SE HACE EL MISMO DÍA DEL CONSUMO [X] Sí [ ] No

Si la fecha de elaboración del plato NO es el mismo día del consumo NO se adjuntará foto y el peso de los ingredientes puede ser aproximado. Indique la fecha de elaboración:

TIPO DE PLATO: *PURÉ DE VERDURAS CON PESCADO*

	Tipo de ingrediente	Nº	Peso (en gramos)
Ingrediente 1	*Patata*	*1*	*110 gr*
Ingrediente 2	*Judías verdes (congeladas)*		*136 gr*
Ingrediente 3	*Calabacín (congelado)*		*149 gr*
Ingrediente 4	*Calabaza (congelada)*		*149 gr*
Ingrediente 5	*Puerro*		*60 gr*
Ingrediente 6	*Arroz*		*55 gr*
Ingrediente 7	*Pescado (abadejo)*		*141 gr*
Ingrediente 8	*Aceite de oliva*		*2 cucharadas soperas*
Ingrediente 9			
Ingrediente 10			

Proceso culinario	Nº	Peso (en gramos)	Observaciones
1. Olla VACÍA		*256 gr*	
2. Olla con COMIDA COCINADA		*2340 gr*	
**COMIDA COCINADA (2-1)**		*2084 gr*	
3. Fiambrera VACÍA		*64 gr*	*Relleno una fiambrera*
4. Fiambrera con COMIDA COCINADA		*393 gr*	
**COMIDA ALMACENADA (4-3)**		*329 gr*	
5. Plato VACÍO		*252 gr*	
6. Plato con COMIDA COCINADA		*644 gr*	
**COMIDA QUE SIRVE A SU HIJO (6-5)**		*392 gr*	
7. Plato COMIDA QUE SU HIJO NO HA COMIDO		*272 gr*	
**COMIDA QUE SU HIJO HA COMIDO (6-7)**		*372 gr*	

MÉTODO DE ELABORACIÓN:
Explicar detalladamente el proceso de elaboración del plato

FICHA INGREDIENTES/PESOS "PLATO HECHO EN CASA (HC)" **ID: MAD 04**

DÍA DEL CONSUMO: 12/ 10 / 2014 **HORA DEL CONSUMO:** 13:30

MÉTODO DE ELABORACIÓN:
Explicar detalladamente el proceso de elaboración del plato:

Previamente, las judías verdes, calabaza y calabacín ya están lavados y pelados para su congelación.
El peso de estos ingredientes es congelado.
Pelar y lavar la patata y el puerro.
Ponerlo todo en la cazuela cuando el agua empiece a hervir.
Dejarlo durante 20 minutos y otros 20 minutos de reposo antes de triturarlo.
Triturarlo todo con un poco de agua de cocción y añadiendo el aceite en crudo.
Servir una ración y el resto congelarlo para otra comida.

(Hago dos raciones, una la que se come y otra la congelo).

Fotos:

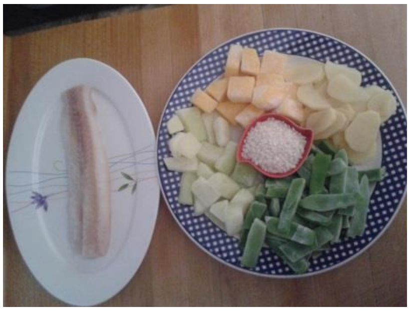

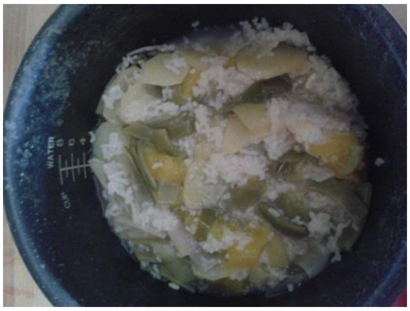

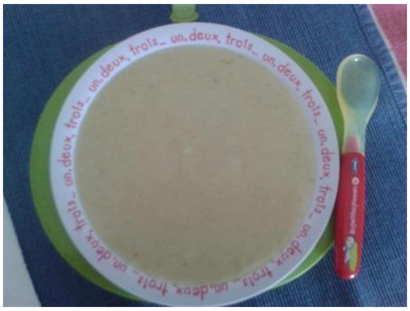

FICHA INGREDIENTES/PESOS “PLATO HECHO EN CASA (HC)” **ID: MAD 04**

DÍA DEL CONSUMO: *12 / 10 / 2014* HORA DEL CONSUMO: *17:45*

LA ELABOARACIÓN DEL PLATO SE HACE EL MISMO DÍA DEL CONSUMO [X] Sí [ ] No

Si la fecha de elaboración del plato NO es el mismo día del consumo NO se adjuntará foto y el peso de los ingredientes puede ser aproximado. Indique la fecha de elaboración:

TIPO DE PLATO: *PAPILLA DE FRUTAS*

	Tipo de ingrediente	Nº	Peso (en gramos)
Ingrediente 1	*Naranja*		*158 gr*
Ingrediente 2	*Manzana*		*90 gr*
Ingrediente 3	*Pera*		*87 gr*
Ingrediente 4	*Plátano*		*81 gr*
Ingrediente 5	*Galleta (María)*		*2 unidades*
Ingrediente 6			
Ingrediente 7			
Ingrediente 8			
Ingrediente 9			
Ingrediente 10			

Proceso culinario	Nº	Peso (en gramos)	Observaciones
1. Olla VACÍA			
2. Olla con COMIDA COCINADA			
**COMIDA COCINADA (2-1)**			
3. Fiambrera VACÍA			
4. Fiambrera con COMIDA COCINADA			
**COMIDA ALMACENADA (4-3)**			
5. Plato VACÍO		*98 gr*	
6. Plato con COMIDA COCINADA		*320 gr*	
**COMIDA QUE SIRVE A SU HIJO (6-5)**		*222 gr*	
7. Plato COMIDA QUE SU HIJO NO HA COMIDO		*162 gr*	
**COMIDA QUE SU HIJO HA COMIDO (6-7)**		*158 gr*	

MÉTODO DE ELABORACIÓN:
Explicar detalladamente el proceso de elaboración del plato

FICHA INGREDIENTES/PESOS "PLATO HECHO EN CASA (HC)" **ID: MAD 04**

DÍA DEL CONSUMO: 12/ 10 / 2014 **HORA DEL CONSUMO:** 17:45

MÉTODO DE ELABORACIÓN:
Explicar detalladamente el proceso de elaboración del plato:

El peso de la fruta es sin pelar.

Pelamos toda la fruta, corto en dados y la echo en el vaso de la batidora.
Troceo la galleta y lo bato todo junto.
Una vez batido listo para servir.

Fotos:

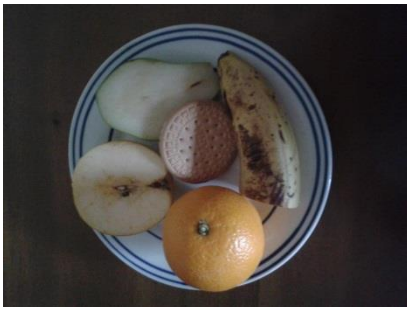

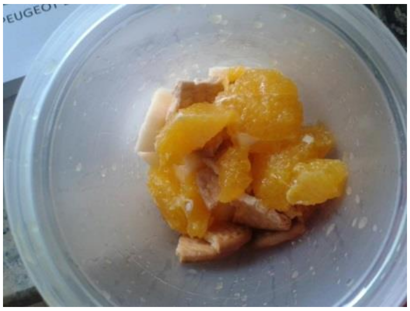

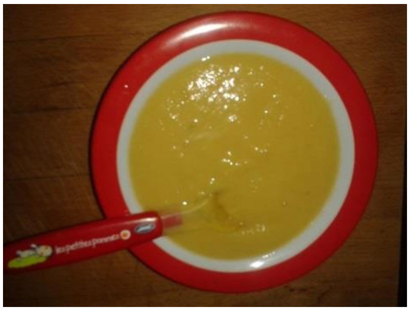

FICHA INGREDIENTES/PESOS "PLATO HECHO EN CASA (HC)" **ID: MAD 04**

DÍA DEL CONSUMO: *14 / 10 / 2014* HORA DEL CONSUMO: *13:10*

LA ELABOARACIÓN DEL PLATO SE HACE EL MISMO DÍA DEL CONSUMO [X] Sí [ ] No

Si la fecha de elaboración del plato NO es el mismo día del consumo NO se adjuntará foto y el peso de los ingredientes puede ser aproximado. Indique la fecha de elaboración:

TIPO DE PLATO: *PURÉ DE VERDURAS CON POLLO*

	Tipo de ingrediente	Nº	Peso (en gramos)
Ingrediente 1	*Patata*	*1*	*80 gr*
Ingrediente 2	*Judías verdes (congeladas)*		*133 gr*
Ingrediente 3	*Calabacín (congelado)*		*127 gr*
Ingrediente 4	*Calabaza*		*126 gr*
Ingrediente 5	*Puerro*		*57 gr*
Ingrediente 6	*Arroz*		*38 gr*
Ingrediente 7	*Pollo*		*116 gr*
Ingrediente 8	*Aceite de oliva*		*2 cucharadas soperas*
Ingrediente 9			
Ingrediente 10			

Proceso culinario	Nº	Peso (en gramos)	Observaciones
1. Olla VACÍA		*256 gr*	
2. Olla con COMIDA COCINADA		*2243 gr*	
**COMIDA COCINADA (2-1)**		*1987 gr*	
3. Fiambrera VACÍA		*64 gr*	*Relleno una fiambrera*
4. Fiambrera con COMIDA COCINADA		*390 gr*	
**COMIDA ALMACENADA (4-3)**		*326 gr*	
5. Plato VACÍO		*252 gr*	
6. Plato con COMIDA COCINADA		*585 gr*	
**COMIDA QUE SIRVE A SU HIJO (6-5)**		*333 gr*	
7. Plato COMIDA QUE SU HIJO NO HA COMIDO		*265 gr*	
**COMIDA QUE SU HIJO HA COMIDO (6-7)**		*320 gr*	

MÉTODO DE ELABORACIÓN:
Explicar detalladamente el proceso de elaboración del plato

FICHA INGREDIENTES/PESOS "PLATO HECHO EN CASA (HC)"

ID: MAD 04

DÍA DEL CONSUMO: 14/ 10 / 2014 **HORA DEL CONSUMO:** 13:10

MÉTODO DE ELABORACIÓN:

Explicar detalladamente el proceso de elaboración del plato:

Las judías verdes y el calabacín ya están pelados y lavados para su congelación.
El peso de estos ingredientes es congelado.
Pelar y lavar la patata, puerro y calabaza.
Ponerlo todo en la cazuela cuando el agua empieza a hervir.
Dejarlo cocer 20 minutos y otros 20 minutos en reposo.
Triturarlo todo con un poco de agua de cocción añadiendo el aceite de oliva.
Servir una ración y el resto guardarlo para otro día.

(Siempre hago para dos días, el día que lo hago y otra ración que congelo).

Fotos:

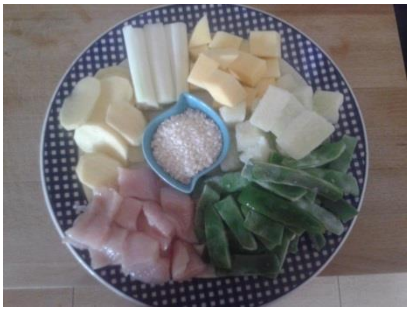

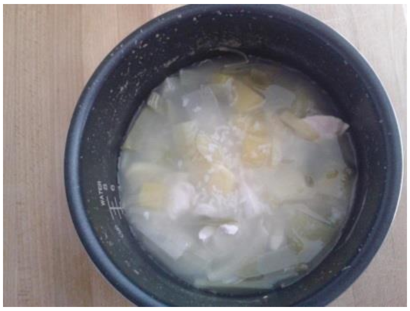

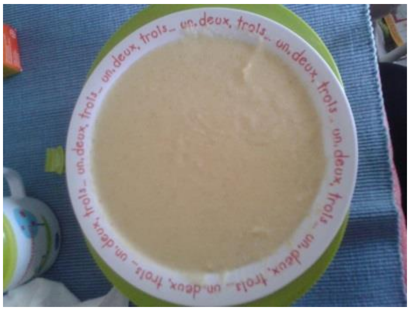

FICHA INGREDIENTES/PESOS "PLATO HECHO EN CASA (HC)" **ID: MAD 04**

DÍA DEL CONSUMO: *14 / 10 / 2014* HORA DEL CONSUMO: *17:30*

LA ELABOARACIÓN DEL PLATO SE HACE EL MISMO DÍA DEL CONSUMO [X] Sí [ ] No

Si la fecha de elaboración del plato NO es el mismo día del consumo NO se adjuntará foto y el peso de los ingredientes puede ser aproximado. Indique la fecha de elaboración:

TIPO DE PLATO: *PAPILLA DE FRUTAS*

	Tipo de ingrediente	Nº	Peso (en gramos)
Ingrediente 1	*Manzana*		*68 gr*
Ingrediente 2	*Pera*		*87 gr*
Ingrediente 3	*Plátano*		*55 gr*
Ingrediente 4	*Naranja*		*71 gr*
Ingrediente 5	*Galleta (María)*	*2*	
Ingrediente 6			
Ingrediente 7			
Ingrediente 8			
Ingrediente 9			
Ingrediente 10			

Proceso culinario	Nº	Peso (en gramos)	Observaciones
1. Olla VACÍA			
2. Olla con COMIDA COCINADA			
**COMIDA COCINADA (2-1)**			
3. Fiambrera VACÍA			
4. Fiambrera con COMIDA COCINADA			
**COMIDA ALMACENADA (4-3)**			
5. Plato VACÍO		*98 gr*	
6. Plato con COMIDA COCINADA		*232 gr*	
**COMIDA QUE SIRVE A SU HIJO (6-5)**		*134 gr*	
7. Plato COMIDA QUE SU HIJO NO HA COMIDO		*100 gr*	
**COMIDA QUE SU HIJO HA COMIDO (6-7)**		*132 gr*	

MÉTODO DE ELABORACIÓN:
Explicar detalladamente el proceso de elaboración del plato

FICHA INGREDIENTES/PESOS "PLATO HECHO EN CASA (HC)"

ID: MAD 04

DÍA DEL CONSUMO: 14/ 10 / 2014 **HORA DEL CONSUMO:** 17:30

MÉTODO DE ELABORACIÓN:

Explicar detalladamente el proceso de elaboración del plato:

El peso de la fruta es ya pelado y listo para batir.
Limpio bien las frutas guitando la cáscara y el corazón.
La parto a trocitos y la pongo en el vaso medidor.
Añado la galleta troceada.
Bato todo y listo para consumir.

Fotos:

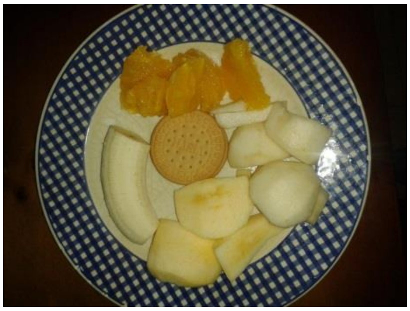
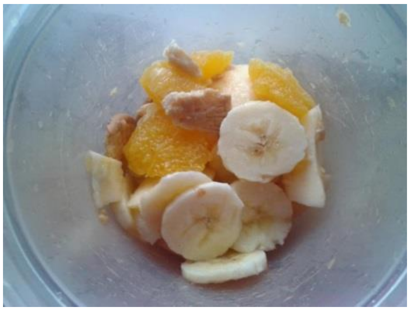
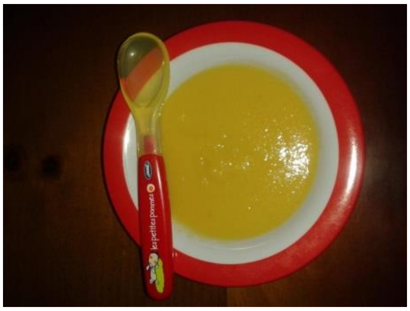

FICHA INGREDIENTES/PESOS "PLATO HECHO EN CASA (HC)" **ID: MAD 04**

DÍA DEL CONSUMO: *16 / 10 / 2014* HORA DEL CONSUMO: *13:10*

LA ELABOARACIÓN DEL PLATO SE HACE EL MISMO DÍA DEL CONSUMO [X] Sí [ ] No

Si la fecha de elaboración del plato NO es el mismo día del consumo NO se adjuntará foto y el peso de los ingredientes puede ser aproximado. Indique la fecha de elaboración:

TIPO DE PLATO: *PURÉ DE VERDURAS CON TERNERA*

	Tipo de ingrediente	Nº	Peso (en gramos)
Ingrediente 1	*Patata*	*1*	*77 gr*
Ingrediente 2	*Zanahoria*		*68 gr*
Ingrediente 3	*Judías verdes*		*80 gr*
Ingrediente 4	*Calabaza (congelado)*		*100 gr*
Ingrediente 5	*Calabacín (congelado)*		*98 gr*
Ingrediente 6	*Puerro*		*49 gr*
Ingrediente 7	*Arroz*		*32 gr*
Ingrediente 8	*Ternera*		*118 gr*
Ingrediente 9	*Aceite de oliva*		*2 cucharadas soperas*
Ingrediente 10			

Proceso culinario	Nº	Peso (en gramos)	Observaciones
1. Olla VACÍA		*256 gr*	
2. Olla con COMIDA COCINADA		*2079 gr*	
**COMIDA COCINADA (2-1)**		*1823 gr*	
3. Fiambrera VACÍA		*64 gr*	
4. Fiambrera con COMIDA COCINADA		*340 gr*	
**COMIDA ALMACENADA (4-3)**		*276 gr*	
5. Plato VACÍO		*252 gr*	
6. Plato con COMIDA COCINADA		*586 gr*	
**COMIDA QUE SIRVE A SU HIJO (6-5)**		*334 gr*	
7. Plato COMIDA QUE SU HIJO NO HA COMIDO		*258 gr*	
**COMIDA QUE SU HIJO HA COMIDO (6-7)**		*328 gr*	

MÉTODO DE ELABORACIÓN:
Explicar detalladamente el proceso de elaboración del plato

FICHA INGREDIENTES/PESOS “PLATO HECHO EN CASA (HC)”

ID: MAD 04

DÍA DEL CONSUMO: 16/ 10 / 2014 **HORA DEL CONSUMO:** 13:10

MÉTODO DE ELABORACIÓN:

Explicar detalladamente el proceso de elaboración del plato:

La calabaza y el calabacín ya están lavados y pelados para su congelación.
Limpiamos todas las verduras y las troceamos para echarlas a la cazuela cuando el agua esté hirviendo.
Posteriormente añado el arroz y por ultimo la ternera.
Una vez cocido durante 20 minutos, lo dejo reposar otros 20 minutos.
Después lo echo todo en el vasa para batirlo añadiendo un poco de agua de cocción más dos cucharadas soperas de aceite de oliva.
Una vez batido, servimos y listo.

Fotos:

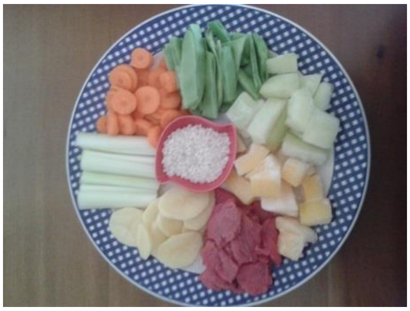

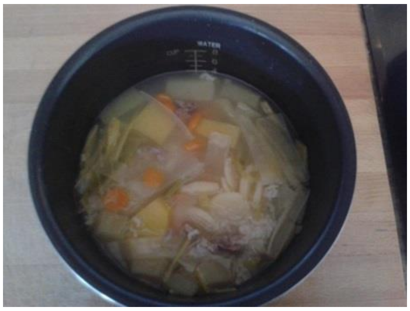

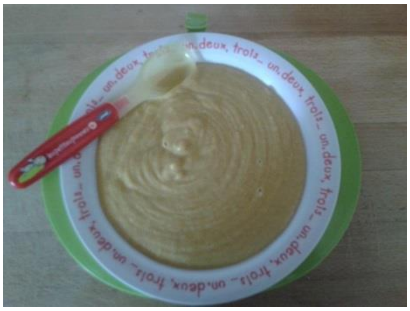

FICHA INGREDIENTES/PESOS "PLATO HECHO EN CASA (HC)" **ID: MAD 04**

DÍA DEL CONSUMO: *16 / 10 / 2014* HORA DEL CONSUMO: *17:30*

LA ELABOARACIÓN DEL PLATO SE HACE EL MISMO DÍA DEL CONSUMO ☒ Sí ☐ No

Si la fecha de elaboración del plato NO es el mismo día del consumo NO se adjuntará foto y el peso de los ingredientes puede ser aproximado. Indique la fecha de elaboración:

TIPO DE PLATO: *PAPILLA DE FRUTAS*

	Tipo de ingrediente	Nº	Peso (en gramos)
Ingrediente 1	*Manzana*		*86 gr*
Ingrediente 2	*Pera*		*81 gr*
Ingrediente 3	*Plátano*		*46 gr*
Ingrediente 4	*Naranja*		*111 gr*
Ingrediente 5	*Galleta (María)*		*2 galletas*
Ingrediente 6			
Ingrediente 7			
Ingrediente 8			
Ingrediente 9			
Ingrediente 10			

Proceso culinario	Nº	Peso (en gramos)	Observaciones
1. Olla VACÍA			
2. Olla con COMIDA COCINADA			
**COMIDA COCINADA (2-1)**			
3. Fiambrera VACÍA			
4. Fiambrera con COMIDA COCINADA			
**COMIDA ALMACENADA (4-3)**			
5. Plato VACÍO		*98 gr*	
6. Plato con COMIDA COCINADA		*283 gr*	
**COMIDA QUE SIRVE A SU HIJO (6-5)**		*185 gr*	
7. Plato COMIDA QUE SU HIJO NO HA COMIDO		*98 gr*	
**COMIDA QUE SU HIJO HA COMIDO (6-7)**		*185 gr*	

MÉTODO DE ELABORACIÓN:
Explicar detalladamente el proceso de elaboración del plato

FICHA INGREDIENTES/PESOS “PLATO HECHO EN CASA (HC)” **ID: MAD 04**

DÍA DEL CONSUMO: 16/ 10 / 2014 **HORA DEL CONSUMO:** 17:30

MÉTODO DE ELABORACIÓN:

Explicar detalladamente el proceso de elaboración del plato:

El peso de la fruta es ya pelado y listo para batir.
Quito la cáscara y el corazón de la manzana, pera y naranja.
Pelo el plátano y hago cuatro trozos con las galletas.
Lo meto todo en el vaso, lo bato y listo para consumir.

Fotos:

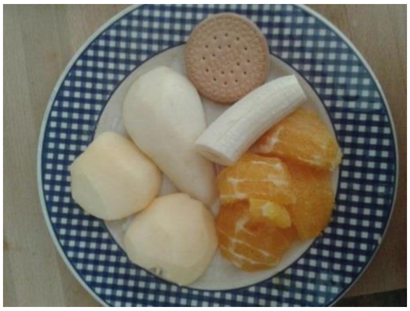

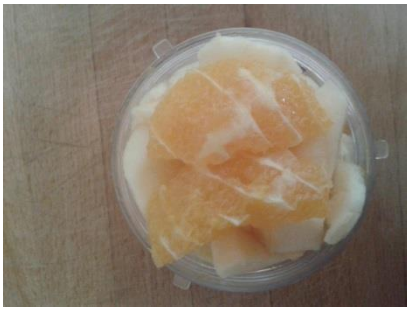

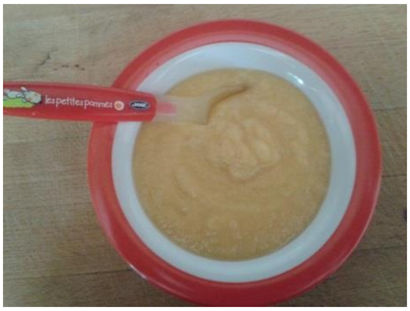

FICHA INGREDIENTES/PESOS “PLATO HECHO EN CASA (HC)” **ID: MAD 05**

DÍA DEL CONSUMO: *11 / 10 / 2014* HORA DEL CONSUMO: *11:50*

LA ELABOARACIÓN DEL PLATO SE HACE EL MISMO DÍA DEL CONSUMO ☒ Sí ☐ No

Si la fecha de elaboración del plato NO es el mismo día del consumo NO se adjuntará foto y el peso de los ingredientes puede ser aproximado. Indique la fecha de elaboración:

TIPO DE PLATO: *PURÉ DE VERDURAS CON PESCADO*

	Tipo de ingrediente	Nº	Peso (en gramos)
Ingrediente 1	*Judías verdes*	*5*	*90 gr*
Ingrediente 2	*Zanahorias*	*4*	*331 gr*
Ingrediente 3	*Patata*	*1*	*327 gr*
Ingrediente 4	*Calabacín*	*1*	*372 gr*
Ingrediente 5	*Puerro*	*1*	*118 gr*
Ingrediente 6	*Lenguado*	*1*	*88 gr*
Ingrediente 7	*Aceite de oliva*		*1 cucharada sopera*
Ingrediente 8	*Agua*		
Ingrediente 9			
Ingrediente 10			

Proceso culinario	Nº	Peso (en gramos)	Observaciones
1. Olla VACÍA	*1*	*786 gr*	
2. Olla con COMIDA COCINADA		*1788 gr*	
**COMIDA COCINADA (2-1)**		*1002 gr*	
3. Fiambrera VACÍA	*1*	*650 gr*	
4. Fiambrera con COMIDA COCINADA		*1050 gr*	
**COMIDA ALMACENADA (4-3)**		*400 gr*	
5. Plato VACÍO		*53 gr*	
6. Plato con COMIDA COCINADA		*270 gr*	
**COMIDA QUE SIRVE A SU HIJO (6-5)**		*217 gr*	
7. Plato COMIDA QUE SU HIJO NO HA COMIDO		*130 gr*	
**COMIDA QUE SU HIJO HA COMIDO (6-7)**		*140 gr*	

MÉTODO DE ELABORACIÓN:
Explicar detalladamente el proceso de elaboración del plato

FICHA INGREDIENTES/PESOS "PLATO HECHO EN CASA (HC)"

ID: MAD 05

DÍA DEL CONSUMO: 11/ 10 / 2014 **HORA DEL CONSUMO:** 11:50

MÉTODO DE ELABORACIÓN:

Explicar detalladamente el proceso de elaboración del plato:

Pelamos la verdura y la cortamos.
Lo ponemos en una olla con una cucharada sopera de aceite de oliva y echamos agua para cubrir los alimentos.
Lo ponemos a cocer durante aproximadamente 40 minutos.
Echamos también el pescado junto con la verdura.
Una vez cocido lo pasamos por la batidora y seguidamente por el pasapuré.
Ya está listo para servir.

Fotos:

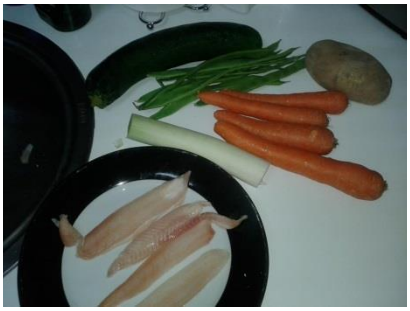

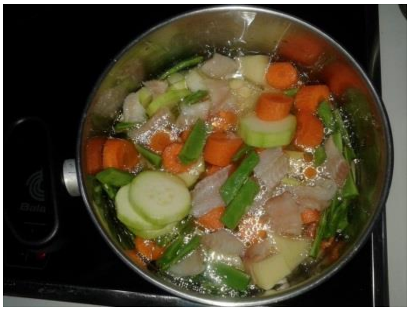

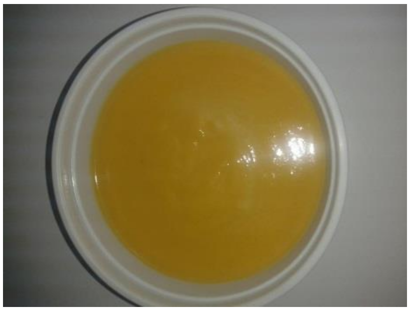

FICHA INGREDIENTES/PESOS “PLATO HECHO EN CASA (HC)” **ID: MAD 05**

DÍA DEL CONSUMO: *11 / 10 / 2014* HORA DEL CONSUMO: *17:40*

LA ELABOARACIÓN DEL PLATO SE HACE EL MISMO DÍA DEL CONSUMO [X] Sí [ ] No

Si la fecha de elaboración del plato NO es el mismo día del consumo NO se adjuntará foto y el peso de los ingredientes puede ser aproximado. Indique la fecha de elaboración:

TIPO DE PLATO: *PAPILLA DE FRUTAS*

	Tipo de ingrediente	Nº	Peso (en gramos)
Ingrediente 1	*Zumo de naranjas*	*2*	*311 gr*
Ingrediente 2	*Manzana roja*	*1*	*132 gr*
Ingrediente 3	*Manzana amarilla*	*1*	*133 gr*
Ingrediente 4	*Peras*	*2*	*270 gr*
Ingrediente 5	*Galleta*	*2*	*12 gr*
Ingrediente 6			
Ingrediente 7			
Ingrediente 8			
Ingrediente 9			
Ingrediente 10			

Proceso culinario	Nº	Peso (en gramos)	Observaciones
1. Olla VACÍA	*1*	*141 gr*	
2. Olla con COMIDA COCINADA		*607 gr*	
**COMIDA COCINADA (2-1)**		*466 gr*	
3. Fiambrera VACÍA			
4. Fiambrera con COMIDA COCINADA			
**COMIDA ALMACENADA (4-3)**			
5. Plato VACÍO		*53 gr*	
6. Plato con COMIDA COCINADA		*273 gr*	
**COMIDA QUE SIRVE A SU HIJO (6-5)**		*220 gr*	
7. Plato COMIDA QUE SU HIJO NO HA COMIDO		*70 gr*	
**COMIDA QUE SU HIJO HA COMIDO (6-7)**		*203 gr*	

MÉTODO DE ELABORACIÓN:
Explicar detalladamente el proceso de elaboración del plato

FICHA INGREDIENTES/PESOS "PLATO HECHO EN CASA (HC)"

ID: MAD 05

DÍA DEL CONSUMO: 11/ 10 / 2014 **HORA DEL CONSUMO:** 17:40

MÉTODO DE ELABORACIÓN:
Explicar detalladamente el proceso de elaboración del plato:

Se hace el zumo con las naranjas.
Lo colamos.
Echamos las galletas para que se vayan deshaciendo.
Pelamos el resto de la fruta, la cortamos y se lo añadimos al zumo con galletas.
Pasamos todo por la batidora y listo.

Fotos:

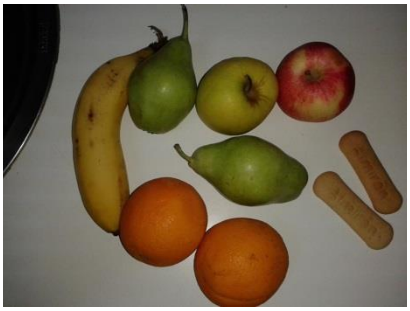

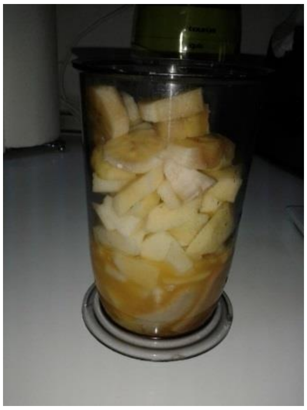

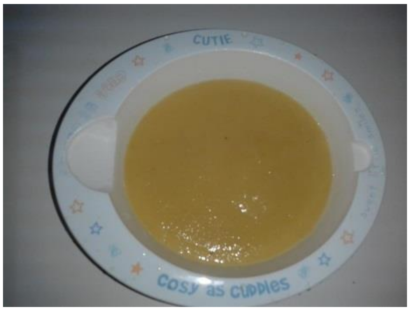

FICHA INGREDIENTES/PESOS "PLATO HECHO EN CASA (HC)" **ID: MAD 06**

DÍA DEL CONSUMO: *12 / 10 / 2014* HORA DEL CONSUMO: *12:00*

LA ELABOARACIÓN DEL PLATO SE HACE EL MISMO DÍA DEL CONSUMO [X] Sí [ ] No

Si la fecha de elaboración del plato NO es el mismo día del consumo NO se adjuntará foto y el peso de los ingredientes puede ser aproximado. Indique la fecha de elaboración: 12-10-14

TIPO DE PLATO: *ARROZ CON POLLO TIERNO*

	Tipo de ingrediente	Nº	Peso (en gramos)
Ingrediente 1	*Aceite de oliva*	*1*	*1 chorro*
Ingrediente 2	*Patata mediana*	*1*	*277 gr*
Ingrediente 3	*Zanahoria pequeña*	*1*	*66 gr*
Ingrediente 4	*Agua*	*1*	*1 litro*
Ingrediente 5	*Muslo de pollo*	*1*	*62 gr*
Ingrediente 6	*Sal*	*1*	*1 pizca*
Ingrediente 7	*Arroz*	*1*	*10 gr*
Ingrediente 8			
Ingrediente 9			
Ingrediente 10			

Proceso culinario	Nº	Peso (en gramos)	Observaciones
1. Olla VACÍA	*1*	*513 gr*	
2. Olla con COMIDA COCINADA	*1*	*1786 gr*	
**COMIDA COCINADA (2-1)**		*1273 gr*	
3. Fiambrera VACÍA	*1*	*87 gr*	
4. Fiambrera con COMIDA COCINADA	*1*	*885 gr*	
**COMIDA ALMACENADA (4-3)**		*798 gr*	
5. Plato VACÍO	*1*	*89 gr*	
6. Plato con COMIDA COCINADA	*1*	*289 gr*	
**COMIDA QUE SIRVE A SU HIJO (6-5)**		*200 gr*	
7. Plato COMIDA QUE SU HIJO NO HA COMIDO	*1*	*139 gr*	
**COMIDA QUE SU HIJO HA COMIDO (6-7)**		*150 gr*	

MÉTODO DE ELABORACIÓN:
Explicar detalladamente el proceso de elaboración del plato

FICHA INGREDIENTES/PESOS "PLATO HECHO EN CASA (HC)"

ID: MAD 06

DÍA DEL CONSUMO: 12 / 10 / 2014 **HORA DEL CONSUMO:** 12:00

MÉTODO DE ELABORACIÓN:

Explicar detalladamente el proceso de elaboración del plato:

Pelar y cortar todas las verduras.
Trocear el pollo.
Echar el arroz.
Hervir todo durante 25 minutos.
Triturar todo con un poco del agua de cocción.
Añadir sal y aceite al final.

Fotos:

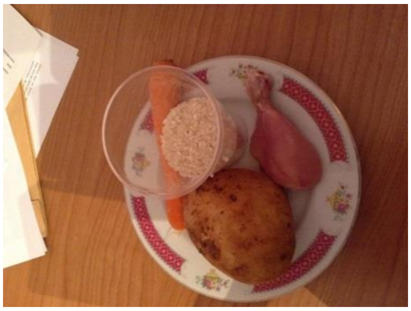

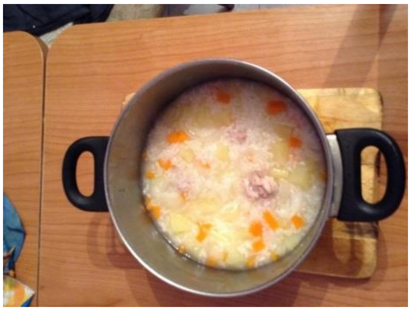

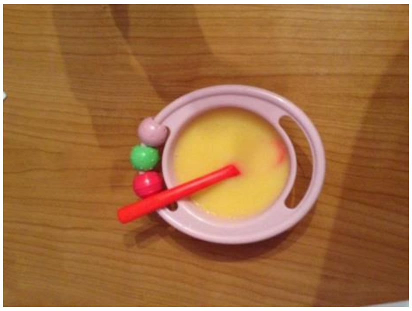

FICHA INGREDIENTES/PESOS “PLATO HECHO EN CASA (HC)” **ID: MAD 07**

DÍA DEL CONSUMO: *13 / 10 / 2014* HORA DEL CONSUMO: *16:00*

LA ELABOARACIÓN DEL PLATO SE HACE EL MISMO DÍA DEL CONSUMO [X] Sí [ ] No

Si la fecha de elaboración del plato NO es el mismo día del consumo NO se adjuntará foto y el peso de los ingredientes puede ser aproximado. Indique la fecha de elaboración:

TIPO DE PLATO: *PAPILLA DE FRUTAS*

	Tipo de ingrediente	Nº	Peso (en gramos) / pelado
Ingrediente 1	*Plátano*	*1*	*148 gr / 85 gr*
Ingrediente 2	*Pera*	*1*	*132 gr / 100 gr*
Ingrediente 3	*Galletas*	*4*	*21 gr / 21 gr*
Ingrediente 4	*Mandarina*	*1*	*165 gr / 124 gr*
Ingrediente 5			
Ingrediente 6			
Ingrediente 7			
Ingrediente 8			
Ingrediente 9			
Ingrediente 10			

Proceso culinario	Nº	Peso (en gramos)	Observaciones
1. Olla VACÍA		*3824 gr*	
2. Olla con COMIDA COCINADA		*4108 gr*	
**COMIDA COCINADA (2-1)**		*284 gr*	
3. Fiambrera VACÍA		*162 gr*	
4. Fiambrera con COMIDA COCINADA		*229 gr*	
**COMIDA ALMACENADA (4-3)**		*67 gr*	
5. Plato VACÍO		*167 gr*	
6. Plato con COMIDA COCINADA		*384 gr*	
**COMIDA QUE SIRVE A SU HIJO (6-5)**		*217 gr*	
7. Plato COMIDA QUE SU HIJO NO HA COMIDO		*178 gr*	
**COMIDA QUE SU HIJO HA COMIDO (6-7)**		*206 gr*	

MÉTODO DE ELABORACIÓN:
Explicar detalladamente el proceso de elaboración del plato

FICHA INGREDIENTES/PESOS "PLATO HECHO EN CASA (HC)" **ID: MAD 07**

DÍA DEL CONSUMO: 13 / 10 / 2014 **HORA DEL CONSUMO:** 16:00

MÉTODO DE ELABORACIÓN:
Explicar detalladamente el proceso de elaboración del plato:

Pelar y cortar las frutas.
Trocear las galletas.
Triturar todo.

Fotos:

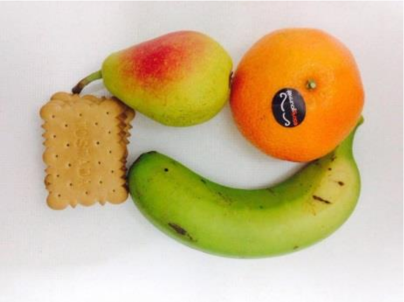

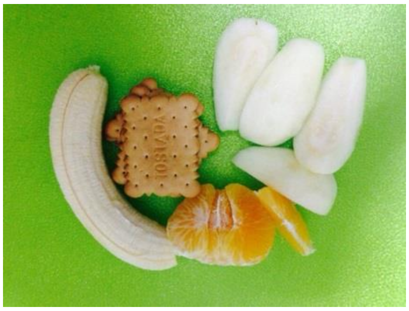

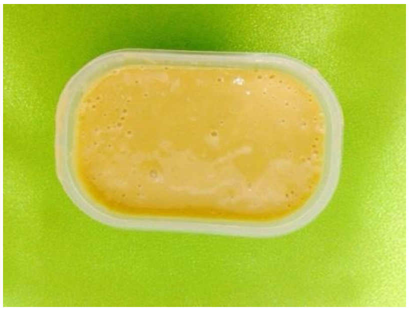

FICHA INGREDIENTES/PESOS "PLATO HECHO EN CASA (HC)" **ID: MAD 07**

DÍA DEL CONSUMO: *13 / 10 / 2014* HORA DEL CONSUMO: *20:00*

LA ELABOARACIÓN DEL PLATO SE HACE EL MISMO DÍA DEL CONSUMO [X] Sí [ ] No

Si la fecha de elaboración del plato NO es el mismo día del consumo NO se adjuntará foto y el peso de los ingredientes puede ser aproximado. Indique la fecha de elaboración:

TIPO DE PLATO: *PURÉ DE PESCADO*

	Tipo de ingrediente	Nº	Peso (en gramos)
Ingrediente 1	*Dorada*	*1*	*152 gr*
Ingrediente 2	*Patata*	*1*	*92 gr*
Ingrediente 3	*Zanahoria*	*1*	*78 gr*
Ingrediente 4	*Calabacín*	*½*	*202 gr*
Ingrediente 5	*Sal*	*1*	*Pizca*
Ingrediente 6	*Aceite*	*1*	*Chorrito*
Ingrediente 7			
Ingrediente 8			
Ingrediente 9			
Ingrediente 10			

Proceso culinario	Nº	Peso (en gramos)	Observaciones
1. Olla VACÍA		*3824 gr*	
2. Olla con COMIDA COCINADA		*4133 gr*	
**COMIDA COCINADA (2-1)**		*309 gr*	
3. Fiambrera VACÍA		*162 gr*	
4. Fiambrera con COMIDA COCINADA		*224 gr*	
**COMIDA ALMACENADA (4-3)**		*62 gr*	
5. Plato VACÍO		*800 gr*	
6. Plato con COMIDA COCINADA		*1046 gr*	
**COMIDA QUE SIRVE A SU HIJO (6-5)**		*246 gr*	
7. Plato COMIDA QUE SU HIJO NO HA COMIDO		*819 gr*	
**COMIDA QUE SU HIJO HA COMIDO (6-7)**		*227 gr*	

MÉTODO DE ELABORACIÓN:
Explicar detalladamente el proceso de elaboración del plato

FICHA INGREDIENTES/PESOS "PLATO HECHO EN CASA (HC)"

ID: MAD 07

DÍA DEL CONSUMO: 13 / 10 / 2014 **HORA DEL CONSUMO:** 20:00

MÉTODO DE ELABORACIÓN:

Explicar detalladamente el proceso de elaboración del plato:

Pelar y cortar las verduras.
Trocear la dorada.
Hervir durante 25 minutos.
Triturar todo con un chorrito de aceite y una pizca de sal.

Fotos:

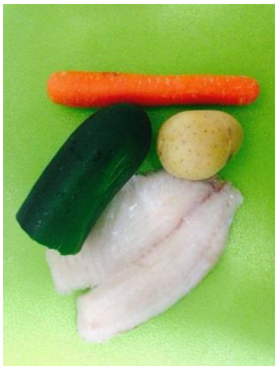

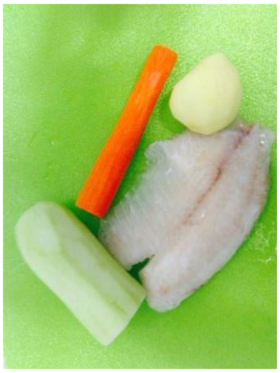

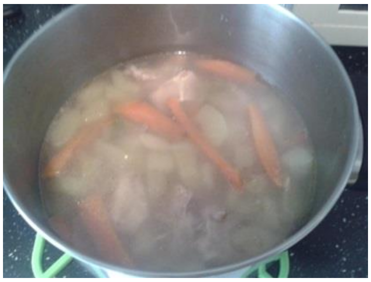

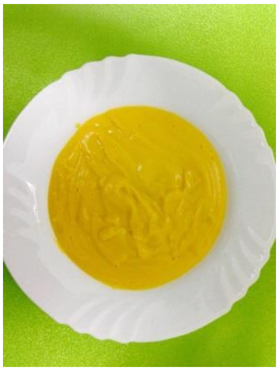

FICHA INGREDIENTES/PESOS "PLATO HECHO EN CASA (HC)" **ID: MAD 07**

DÍA DEL CONSUMO: *14 / 10 / 2014* HORA DEL CONSUMO: *16:00*

LA ELABOARACIÓN DEL PLATO SE HACE EL MISMO DÍA DEL CONSUMO [X] Sí [ ] No

Si la fecha de elaboración del plato NO es el mismo día del consumo NO se adjuntará foto y el peso de los ingredientes puede ser aproximado. Indique la fecha de elaboración:

TIPO DE PLATO: *PAPILLA DE FRUTAS*

	Tipo de ingrediente	Nº	Peso (en gramos) / pelada
Ingrediente 1	*Zumo de naranja*	*1*	*100 gr / 100 gr*
Ingrediente 2	*Manzana*	*½*	*120 gr / 92 gr*
Ingrediente 3	*Galletas*	*10*	*50 gr / 50 gr*
Ingrediente 4	*Pera*	*1*	*109 gr / 88 gr*
Ingrediente 5	*Plátano*	*1*	*217 gr / 101 gr*
Ingrediente 6			
Ingrediente 7			
Ingrediente 8			
Ingrediente 9			
Ingrediente 10			

Proceso culinario	Nº	Peso (en gramos)	Observaciones
1. Olla VACÍA		*3824 gr*	
2. Olla con COMIDA COCINADA		*4240 gr*	
**COMIDA COCINADA (2-1)**		*416 gr*	
3. Fiambrera VACÍA		*162 gr*	
4. Fiambrera con COMIDA COCINADA		*288 gr*	
**COMIDA ALMACENADA (4-3)**		*126 gr*	
5. Plato VACÍO		*167 gr*	
6. Plato con COMIDA COCINADA		*457 gr*	
**COMIDA QUE SIRVE A SU HIJO (6-5)**		*290 gr*	
7. Plato COMIDA QUE SU HIJO NO HA COMIDO		*199 gr*	
**COMIDA QUE SU HIJO HA COMIDO (6-7)**		*258 gr*	

MÉTODO DE ELABORACIÓN:
Explicar detalladamente el proceso de elaboración del plato

FICHA INGREDIENTES/PESOS “PLATO HECHO EN CASA (HC)” **ID: MAD 07**

DÍA DEL CONSUMO: 14 / 10 / 2014 **HORA DEL CONSUMO:** 16:00

MÉTODO DE ELABORACIÓN:
Explicar detalladamente el proceso de elaboración del plato:

Pelar, cortar y triturar todas las frutas añadiendo el zumo de naranja y las galletas troceadas.

Fotos:

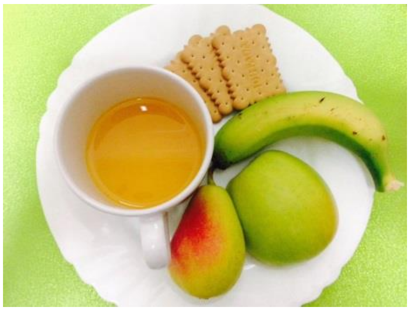
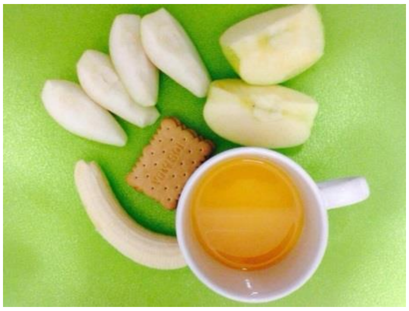
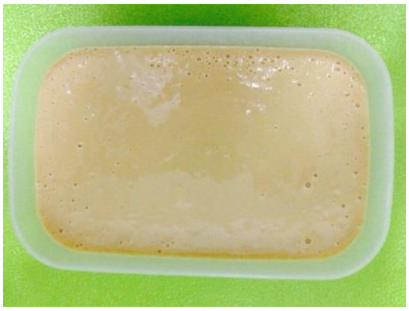

FICHA INGREDIENTES/PESOS "PLATO HECHO EN CASA (HC)" **ID: MAD 07**

DÍA DEL CONSUMO: *14 / 10 / 2014* HORA DEL CONSUMO: *20:00*

LA ELABOARACIÓN DEL PLATO SE HACE EL MISMO DÍA DEL CONSUMO [X] Sí [ ] No

Si la fecha de elaboración del plato NO es el mismo día del consumo NO se adjuntará foto y el peso de los ingredientes puede ser aproximado. Indique la fecha de elaboración:

TIPO DE PLATO: *PURÉ DE VERDURAS*

	Tipo de ingrediente	Nº	Peso (en gramos)
Ingrediente 1	*Judías verdes*	*1*	*128 gr*
Ingrediente 2	*Zanahorias*	*1*	*119 gr*
Ingrediente 3	*Guisantes*	*1*	*43 gr*
Ingrediente 4	*Brócoli*	*1*	*99 gr*
Ingrediente 5	*Patata*	*1*	*97 gr*
Ingrediente 6	*Sal*	*1*	*Pizca*
Ingrediente 7	*Aceite*	*1*	*Chorrito*
Ingrediente 8	*Agua*	*1*	*50 gr*
Ingrediente 9			
Ingrediente 10			

Proceso culinario	Nº	Peso (en gramos)	Observaciones
1. Olla VACÍA		*3824 gr*	
2. Olla con COMIDA COCINADA		*4234 gr*	
**COMIDA COCINADA (2-1)**		*410 gr*	
3. Fiambrera VACÍA		*162 gr*	
4. Fiambrera con COMIDA COCINADA		*286 gr*	
**COMIDA ALMACENADA (4-3)**		*124 gr*	
5. Plato VACÍO		*800 gr*	
6. Plato con COMIDA COCINADA		*1086 gr*	
**COMIDA QUE SIRVE A SU HIJO (6-5)**		*286 gr*	
7. Plato COMIDA QUE SU HIJO NO HA COMIDO		*832 gr*	
**COMIDA QUE SU HIJO HA COMIDO (6-7)**		*254 gr*	

MÉTODO DE ELABORACIÓN:
Explicar detalladamente el proceso de elaboración del plato

FICHA INGREDIENTES/PESOS "PLATO HECHO EN CASA (HC)"

ID: MAD 07

DÍA DEL CONSUMO: 14 / 10 / 2014 **HORA DEL CONSUMO:** 20:00

MÉTODO DE ELABORACIÓN:

Explicar detalladamente el proceso de elaboración del plato:

Pelar y cortar todas las verduras.

Hervir todo añadiendo el agua, la sal y el chorrito de aceite durante 20 minutos.

Triturar todo y añadir un chorrito pequeño de aceite.

Fotos:

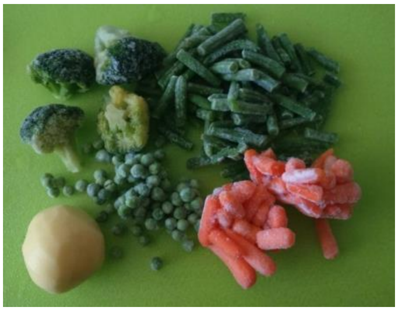

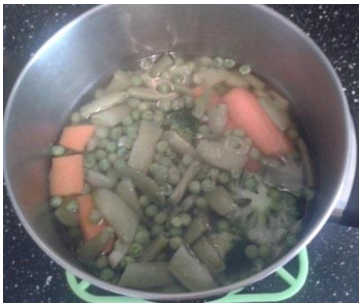

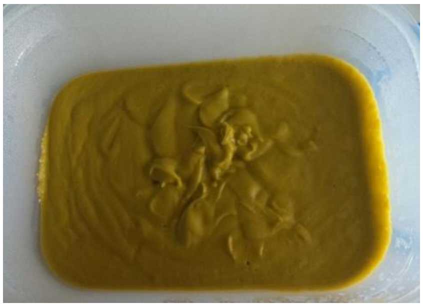

FICHA INGREDIENTES/PESOS "PLATO HECHO EN CASA (HC)" **ID: MAD 07**

DÍA DEL CONSUMO: *18 / 10 / 2014* HORA DEL CONSUMO: *13:30*

LA ELABOARACIÓN DEL PLATO SE HACE EL MISMO DÍA DEL CONSUMO [X] Sí [ ] No

Si la fecha de elaboración del plato NO es el mismo día del consumo NO se adjuntará foto y el peso de los ingredientes puede ser aproximado. Indique la fecha de elaboración:

TIPO DE PLATO: *PURÉ DE POLLO*

	Tipo de ingrediente	Nº	Peso (en gramos)
Ingrediente 1	*Filete de pollo*	*1*	*103 gr*
Ingrediente 2	*Calabacín*	*½*	*179 gr*
Ingrediente 3	*Zanahoria*	*1*	*49 gr*
Ingrediente 4	*Patata*	*1*	*70 gr*
Ingrediente 5	*Lechuga (hojas)*	*2*	*46 gr*
Ingrediente 6	*Agua*	*1*	*85 gr*
Ingrediente 7	*Sal*	*1*	*Pizca*
Ingrediente 8	*Aceite*	*1*	*Chorrito*
Ingrediente 9			
Ingrediente 10			

Proceso culinario	Nº	Peso (en gramos)	Observaciones
1. Olla VACÍA		*3824 gr*	
2. Olla con COMIDA COCINADA		*4152 gr*	
**COMIDA COCINADA (2-1)**		*328 gr*	
3. Fiambrera VACÍA		*162 gr*	
4. Fiambrera con COMIDA COCINADA		*221 gr*	
**COMIDA ALMACENADA (4-3)**		*59 gr*	
5. Plato VACÍO		*800 gr*	
6. Plato con COMIDA COCINADA		*1069 gr*	
**COMIDA QUE SIRVE A SU HIJO (6-5)**		*269 gr*	
7. Plato COMIDA QUE SU HIJO NO HA COMIDO		*813 gr*	
**COMIDA QUE SU HIJO HA COMIDO (6-7)**		*256 gr*	

MÉTODO DE ELABORACIÓN:
Explicar detalladamente el proceso de elaboración del plato

FICHA INGREDIENTES/PESOS "PLATO HECHO EN CASA (HC)"

ID: MAD 07

DÍA DEL CONSUMO: 18 / 10 / 2014 **HORA DEL CONSUMO:** 13:30

MÉTODO DE ELABORACIÓN:

Explicar detalladamente el proceso de elaboración del plato:

Pelar y cortar las verduras.
Trocear el filete de pollo.
Hervir añadiendo el agua, aceite y sal durante 25 minutos.
Triturar todo.

Fotos:

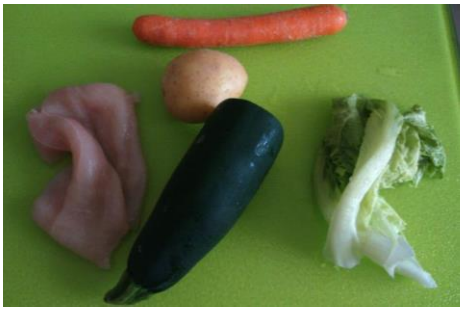

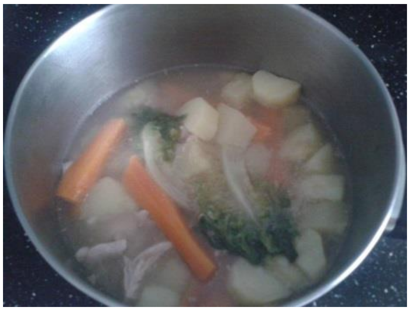

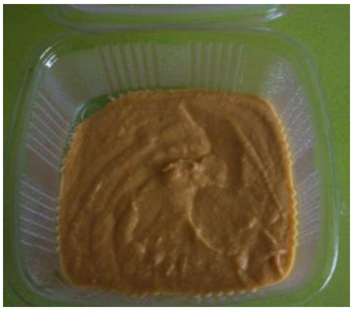

FICHA INGREDIENTES/PESOS "PLATO HECHO EN CASA (HC)" **ID: MAD 07**

DÍA DEL CONSUMO: *18 / 10 / 2014* HORA DEL CONSUMO: *17:00*

LA ELABOARACIÓN DEL PLATO SE HACE EL MISMO DÍA DEL CONSUMO [X] Sí [ ] No

Si la fecha de elaboración del plato NO es el mismo día del consumo NO se adjuntará foto y el peso de los ingredientes puede ser aproximado. Indique la fecha de elaboración:

TIPO DE PLATO: *PAPILLA DE FRUTAS*

	Tipo de ingrediente	Nº	Peso (en gramos) / pelada
Ingrediente 1	*Zumo de naranja*	*1*	*90 gr / 90 gr*
Ingrediente 2	*Galletas*	*6*	*30 gr / 30 gr*
Ingrediente 3	*Plátano*	*1*	*155 gr / 103 gr*
Ingrediente 4	*Manzana*	*1/2*	*120 gr / 102 gr*
Ingrediente 5			
Ingrediente 6			
Ingrediente 7			
Ingrediente 8			
Ingrediente 9			
Ingrediente 10			

Proceso culinario	Nº	Peso (en gramos)	Observaciones
1. Olla VACÍA		*3824 gr*	
2. Olla con COMIDA COCINADA		*4140 gr*	
**COMIDA COCINADA (2-1)**		*316 gr*	
3. Fiambrera VACÍA		*162 gr*	
4. Fiambrera con COMIDA COCINADA		*233 gr*	
**COMIDA ALMACENADA (4-3)**		*71 gr*	
5. Plato VACÍO		*167 gr*	
6. Plato con COMIDA COCINADA		*412 gr*	
**COMIDA QUE SIRVE A SU HIJO (6-5)**		*245 gr*	
7. Plato COMIDA QUE SU HIJO NO HA COMIDO		*180 gr*	
**COMIDA QUE SU HIJO HA COMIDO (6-7)**		*232 gr*	

MÉTODO DE ELABORACIÓN:
Explicar detalladamente el proceso de elaboración del plato

FICHA INGREDIENTES/PESOS "PLATO HECHO EN CASA (HC)"

ID: MAD 07

DÍA DEL CONSUMO: 18 / 10 / 2014 **HORA DEL CONSUMO:** 17:00

MÉTODO DE ELABORACIÓN:

Explicar detalladamente el proceso de elaboración del plato:

Pelar, cortar y triturar todas las frutas junto con los trocitos de galletas y el zumo de naranja.

Fotos:

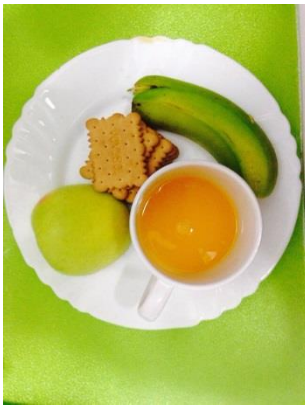

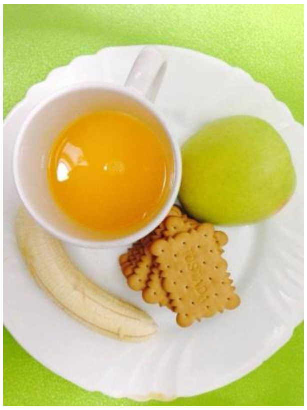

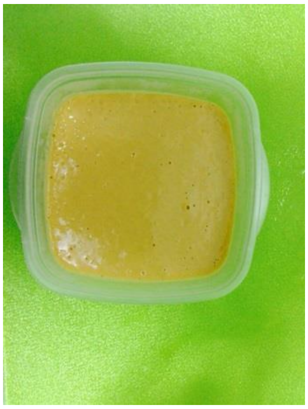

FICHA INGREDIENTES/PESOS “PLATO HECHO EN CASA (HC)” **ID: MAD 08**

DÍA DEL CONSUMO: *13 / 10 / 2014* HORA DEL CONSUMO: *12:10*

LA ELABOARACIÓN DEL PLATO SE HACE EL MISMO DÍA DEL CONSUMO [X] Sí [ ] No

Si la fecha de elaboración del plato NO es el mismo día del consumo NO se adjuntará foto y el peso de los ingredientes puede ser aproximado. Indique la fecha de elaboración:

TIPO DE PLATO: *PURÉ DE VERDURAS Y POLLO*

	Tipo de ingrediente	Nº	Peso (en gramos)
Ingrediente 1	*Calabacín*	*1*	*357 gr*
Ingrediente 2	*Patata*	*1*	*187 gr*
Ingrediente 3	*Zanahoria*	*2*	*142 gr*
Ingrediente 4	*Pollo*	*1/3*	*92 gr*
Ingrediente 5	*Aceite de oliva*	*1*	*1 chorrito*
Ingrediente 6			
Ingrediente 7			
Ingrediente 8			
Ingrediente 9			
Ingrediente 10			

Proceso culinario	Nº	Peso (en gramos)	Observaciones
1. Olla VACÍA		*812 gr*	
2. Olla con COMIDA COCINADA		*1674 gr*	
**COMIDA COCINADA (2-1)**		*862 gr*	
3. Fiambrera VACÍA	*2*	*237 gr*	*Sin tapa*
4. Fiambrera con COMIDA COCINADA		*450 gr*	
**COMIDA ALMACENADA (4-3)**		*213 gr*	
5. Plato VACÍO		*237 gr*	
6. Plato con COMIDA COCINADA		*464 gr*	
**COMIDA QUE SIRVE A SU HIJO (6-5)**		*227 gr*	
7. Plato COMIDA QUE SU HIJO NO HA COMIDO		*245 gr*	
**COMIDA QUE SU HIJO HA COMIDO (6-7)**		*219 gr*	

MÉTODO DE ELABORACIÓN:
Explicar detalladamente el proceso de elaboración del plato

FICHA INGREDIENTES/PESOS "PLATO HECHO EN CASA (HC)"

ID: MAD 08

DÍA DEL CONSUMO: 13 / 10 / 2014 **HORA DEL CONSUMO:** 12:10

MÉTODO DE ELABORACIÓN:

Explicar detalladamente el proceso de elaboración del plato:

Pelo la verdura.
La lavo y después la troceo y la echo en la olla.
Troceo el pollo y lo echo en la olla.
Echo el agua y pongo a cocer unos 25 minutos.
Paso por la batidora con un poco de agua de cocción y un chorro de aceite.

Fotos:

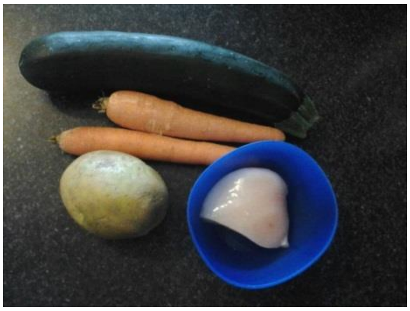

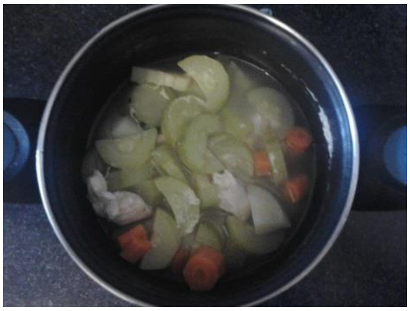

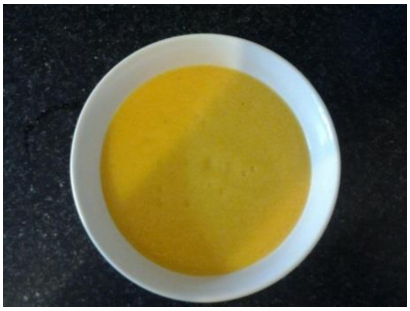

FICHA INGREDIENTES/PESOS "PLATO HECHO EN CASA (HC)" **ID: MAD 08**

DÍA DEL CONSUMO: *13 / 10 / 2014* HORA DEL CONSUMO: *16:00*

LA ELABOARACIÓN DEL PLATO SE HACE EL MISMO DÍA DEL CONSUMO [X] Sí [ ] No

Si la fecha de elaboración del plato NO es el mismo día del consumo NO se adjuntará foto y el peso de los ingredientes puede ser aproximado. Indique la fecha de elaboración:

TIPO DE PLATO: *PAPILLA DE FRUTAS*

	Tipo de ingrediente	Nº	Peso (en gramos)
Ingrediente 1	*Pera*	*1*	*140 gr*
Ingrediente 2	*Manzana*	*1*	*229 gr*
Ingrediente 3	*Plátano*	*1*	*129 gr*
Ingrediente 4	*Galletas*	*2*	*11 gr*
Ingrediente 5			
Ingrediente 6			
Ingrediente 7			
Ingrediente 8			
Ingrediente 9			
Ingrediente 10			

Proceso culinario	Nº	Peso (en gramos)	Observaciones
1. Olla VACÍA		*150 gr*	
2. Olla con COMIDA COCINADA		*622 gr*	
**COMIDA COCINADA (2-1)**		*472 gr*	
3. Fiambrera VACÍA	*2*	*10 gr*	*Sin tapa*
4. Fiambrera con COMIDA COCINADA		*104 gr*	
**COMIDA ALMACENADA (4-3)**		*94 gr*	
5. Plato VACÍO		*37 gr*	
6. Plato con COMIDA COCINADA		*253 gr*	
**COMIDA QUE SIRVE A SU HIJO (6-5)**		*216 gr*	
7. Plato COMIDA QUE SU HIJO NO HA COMIDO		*39 gr*	
**COMIDA QUE SU HIJO HA COMIDO (6-7)**		*214 gr*	

MÉTODO DE ELABORACIÓN:
Explicar detalladamente el proceso de elaboración del plato

FICHA INGREDIENTES/PESOS “PLATO HECHO EN CASA (HC)” **ID: MAD 08**

DÍA DEL CONSUMO: 13 / 10 / 2014 **HORA DEL CONSUMO:** 16:00

MÉTODO DE ELABORACIÓN:

Explicar detalladamente el proceso de elaboración del plato:

Pelo la fruta y la troceo.
La echo en el vaso de la batidora.
Hago en trozos las galletas y las echo en el vaso.
Paso la batidora y sirvo.

Fotos:

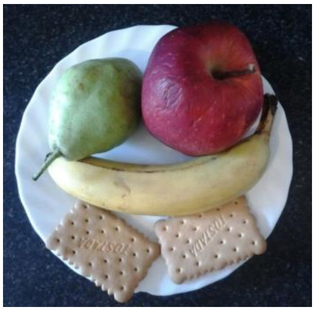

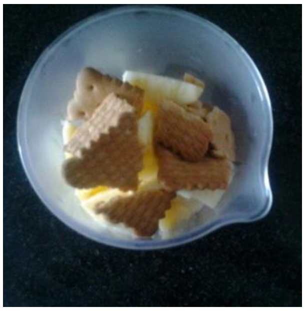

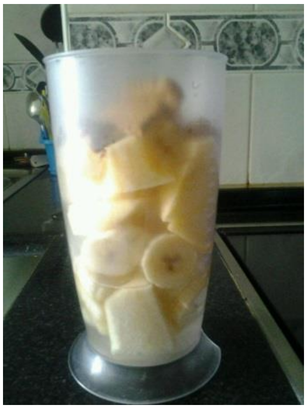

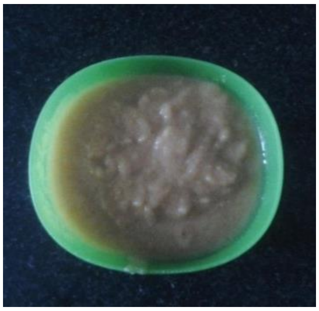

FICHA INGREDIENTES/PESOS “PLATO HECHO EN CASA (HC)” **ID: MAD 08**

DÍA DEL CONSUMO: *15 / 10 / 2014* HORA DEL CONSUMO: *12:10*

LA ELABOARACIÓN DEL PLATO SE HACE EL MISMO DÍA DEL CONSUMO [X] Sí [ ] No

Si la fecha de elaboración del plato NO es el mismo día del consumo NO se adjuntará foto y el peso de los ingredientes puede ser aproximado. Indique la fecha de elaboración:

TIPO DE PLATO: *PURÉ DE VERDURAS Y PESCADO*

	Tipo de ingrediente	Nº	Peso (en gramos)
Ingrediente 1	*Calabacín*	*½*	*280 gr*
Ingrediente 2	*Zanahoria*	*2*	*161 gr*
Ingrediente 3	*Judías verdes*	*5*	*64 gr*
Ingrediente 4	*Acelga*	*1*	*56 gr*
Ingrediente 5	*Patata*	*1*	*224 gr*
Ingrediente 6	*Merluza*	*1*	*70 gr*
Ingrediente 7	*Puerro*	*½*	*76 gr*
Ingrediente 8			
Ingrediente 9			
Ingrediente 10			

Proceso culinario	Nº	Peso (en gramos)	Observaciones
1. Olla VACÍA		*812 gr*	
2. Olla con COMIDA COCINADA		*1907 gr*	
**COMIDA COCINADA (2-1)**		*1095 gr*	
3. Fiambrera VACÍA	*2*	*237 gr*	*Sin tapa*
4. Fiambrera con COMIDA COCINADA		*485 gr*	
**COMIDA ALMACENADA (4-3)**		*248 gr*	
5. Plato VACÍO		*237 gr*	
6. Plato con COMIDA COCINADA		*476 gr*	
**COMIDA QUE SIRVE A SU HIJO (6-5)**		*239 gr*	
7. Plato COMIDA QUE SU HIJO NO HA COMIDO		*263 gr*	
**COMIDA QUE SU HIJO HA COMIDO (6-7)**		*213 gr*	

MÉTODO DE ELABORACIÓN:

Explicar detalladamente el proceso de elaboración del plato

FICHA INGREDIENTES/PESOS "PLATO HECHO EN CASA (HC)"

ID: MAD 08

DÍA DEL CONSUMO: 15 / 10 / 2014 **HORA DEL CONSUMO:** 12:10

MÉTODO DE ELABORACIÓN:

Explicar detalladamente el proceso de elaboración del plato:

Pelo la verdura, la lavo y la troceo.
Quito las hebras a las acelgas, las lavo y las troceo.
Echo toda la verdura en el cazo.
Después echo la merluza en el cazo.
Pongo a cocer 20 minutos.
Lo paso por la batidora y lo sirvo.

Fotos:

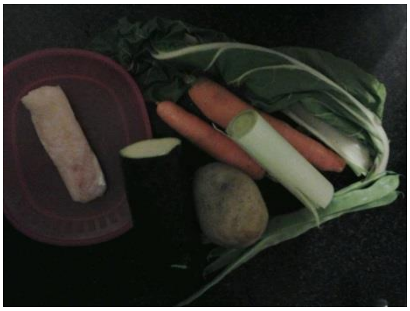

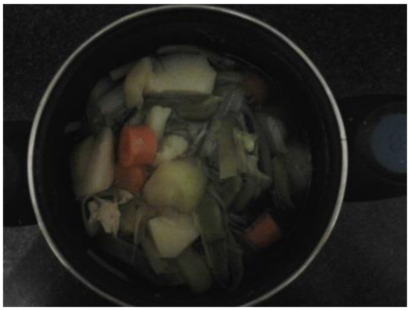

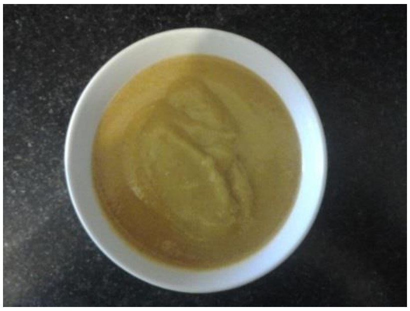

FICHA INGREDIENTES/PESOS “PLATO HECHO EN CASA (HC)” **ID: MAD 08**

DÍA DEL CONSUMO: *15 / 10 / 2014* HORA DEL CONSUMO: *16:00*

LA ELABOARACIÓN DEL PLATO SE HACE EL MISMO DÍA DEL CONSUMO [X] Sí [ ] No

Si la fecha de elaboración del plato NO es el mismo día del consumo NO se adjuntará foto y el peso de los ingredientes puede ser aproximado. Indique la fecha de elaboración:

TIPO DE PLATO: *PAPILLA DE FRUTAS Y GALLETAS*

	Tipo de ingrediente	Nº	Peso (en gramos)
Ingrediente 1	*Manzana*	*1*	*185 gr*
Ingrediente 2	*Pera*	*1*	*169 gr*
Ingrediente 3	*Plátano*	*½*	*85 gr*
Ingrediente 4	*Ciruela*	*1*	*96 gr*
Ingrediente 5	*Galletas*	*2*	*11 gr*
Ingrediente 6			
Ingrediente 7			
Ingrediente 8			
Ingrediente 9			
Ingrediente 10			

Proceso culinario	Nº	Peso (en gramos)	Observaciones
1. Olla VACÍA		*150 gr*	
2. Olla con COMIDA COCINADA		*630 gr*	
**COMIDA COCINADA (2-1)**		*480 gr*	
3. Fiambrera VACÍA	*2*	*10 gr*	*Sin tapa*
4. Fiambrera con COMIDA COCINADA		*112 gr*	
**COMIDA ALMACENADA (4-3)**		*102 gr*	
5. Plato VACÍO		*37 gr*	
6. Plato con COMIDA COCINADA		*265 gr*	
**COMIDA QUE SIRVE A SU HIJO (6-5)**		*228 gr*	
7. Plato COMIDA QUE SU HIJO NO HA COMIDO		*38 gr*	
**COMIDA QUE SU HIJO HA COMIDO (6-7)**		*227 gr*	

MÉTODO DE ELABORACIÓN:
Explicar detalladamente el proceso de elaboración del plato

FICHA INGREDIENTES/PESOS "PLATO HECHO EN CASA (HC)"

ID: MAD 08

DÍA DEL CONSUMO: 15 / 10 / 2014 **HORA DEL CONSUMO:** 16:00

MÉTODO DE ELABORACIÓN:

Explicar detalladamente el proceso de elaboración del plato:

Pelo la fruta, la troceo y la echo en el vaso de la batidora.
Echo también las galletas a trozos en el vaso de la batidora.
Batir y servir.

Fotos:

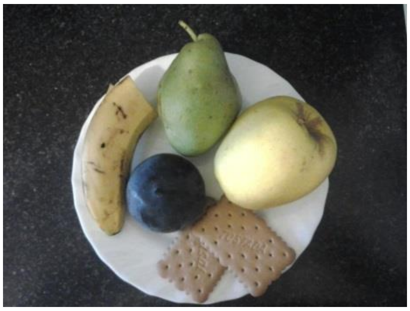

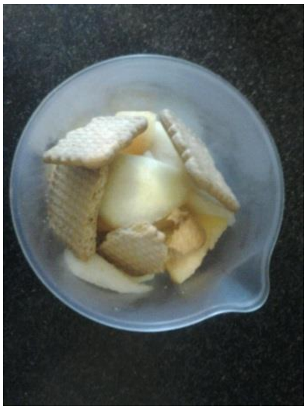

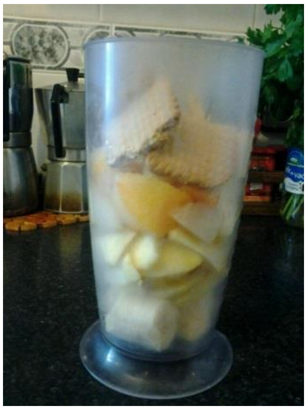

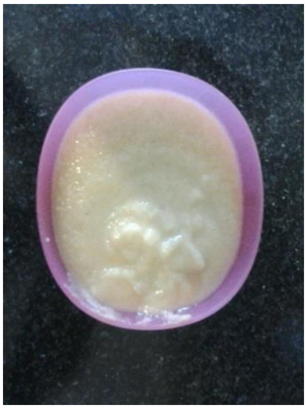

FICHA INGREDIENTES/PESOS "PLATO HECHO EN CASA (HC)" **ID: MAD 08**

DÍA DEL CONSUMO: *18 / 10 / 2014* HORA DEL CONSUMO: *12:30*

LA ELABOARACIÓN DEL PLATO SE HACE EL MISMO DÍA DEL CONSUMO ☒ Sí ☐ No

Si la fecha de elaboración del plato NO es el mismo día del consumo NO se adjuntará foto y el peso de los ingredientes puede ser aproximado. Indique la fecha de elaboración:

TIPO DE PLATO: *PURÉ DE VERDURAS Y TERNERA*

	Tipo de ingrediente	Nº	Peso (en gramos)
Ingrediente 1	*Calabaza*	*1 trozo*	*397 gr*
Ingrediente 2	*Cebolla*	*½*	*38 gr*
Ingrediente 3	*Patata*	*2*	*280 gr*
Ingrediente 4	*Zanahorias*	*1*	*102 gr*
Ingrediente 5	*Ternera*	*1*	*65 gr*
Ingrediente 6			
Ingrediente 7			
Ingrediente 8			
Ingrediente 9			
Ingrediente 10			

Proceso culinario	Nº	Peso (en gramos)	Observaciones
1. Olla VACÍA		*812 gr*	
2. Olla con COMIDA COCINADA		*1673 gr*	
**COMIDA COCINADA (2-1)**		*861 gr*	
3. Fiambrera VACÍA	*3*	*237 gr*	
4. Fiambrera con COMIDA COCINADA		*447 gr*	
**COMIDA ALMACENADA (4-3)**		*210 gr*	
5. Plato VACÍO		*237 gr*	
6. Plato con COMIDA COCINADA		*482 gr*	
**COMIDA QUE SIRVE A SU HIJO (6-5)**		*245 gr*	
7. Plato COMIDA QUE SU HIJO NO HA COMIDO		*252 gr*	
**COMIDA QUE SU HIJO HA COMIDO (6-7)**		*230 gr*	

MÉTODO DE ELABORACIÓN:
Explicar detalladamente el proceso de elaboración del plato

FICHA INGREDIENTES/PESOS "PLATO HECHO EN CASA (HC)" **ID: MAD 08**

DÍA DEL CONSUMO: 18 / 10 / 2014 **HORA DEL CONSUMO:** 12:30

MÉTODO DE ELABORACIÓN:
Explicar detalladamente el proceso de elaboración del plato:

Pelo la verdura, la lavo, la troceo y la echo en la olla.
Echo la ternera, el agua y pongo a cocer durante 35 minutos.
Echo en una jarra y paso por la batidora.
Cuando esté batido, sirvo.

Fotos:

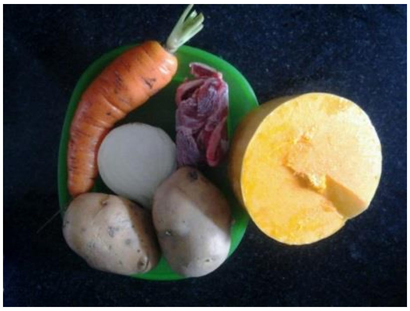

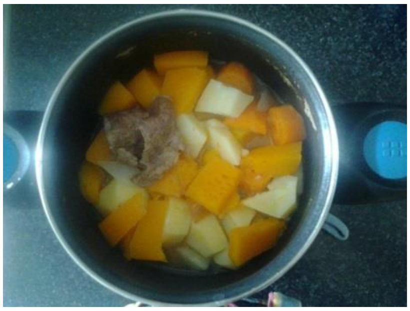

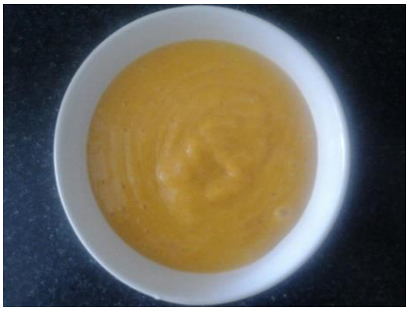

FICHA INGREDIENTES/PESOS "PLATO HECHO EN CASA (HC)" **ID: MAD 08**

DÍA DEL CONSUMO: *18 / 10 / 2014* HORA DEL CONSUMO: *16:15*

LA ELABOARACIÓN DEL PLATO SE HACE EL MISMO DÍA DEL CONSUMO ☒ Sí ☐ No

Si la fecha de elaboración del plato NO es el mismo día del consumo NO se adjuntará foto y el peso de los ingredientes puede ser aproximado. Indique la fecha de elaboración:

TIPO DE PLATO: *PAPILLA DE FRUTAS Y GALLETA*

	Tipo de ingrediente	Nº	Peso (en gramos)
Ingrediente 1	*Manzana*	*1 y ½*	*390 gr*
Ingrediente 2	*Plátano*	*1*	*140 gr*
Ingrediente 3	*Pera*	*1*	*157 gr*
Ingrediente 4	*Galletas*	*2*	*11 gr*
Ingrediente 5			
Ingrediente 6			
Ingrediente 7			
Ingrediente 8			
Ingrediente 9			
Ingrediente 10			

Proceso culinario	Nº	Peso (en gramos)	Observaciones
1. Olla VACÍA		*150 gr*	
2. Olla con COMIDA COCINADA		*570 gr*	
**COMIDA COCINADA (2-1)**		*420 gr*	
3. Fiambrera VACÍA	*2*	*10 gr*	
4. Fiambrera con COMIDA COCINADA		*128 gr*	
**COMIDA ALMACENADA (4-3)**		*118 gr*	
5. Plato VACÍO		*37 gr*	
6. Plato con COMIDA COCINADA		*239 gr*	
**COMIDA QUE SIRVE A SU HIJO (6-5)**		*202 gr*	
7. Plato COMIDA QUE SU HIJO NO HA COMIDO		*59 gr*	
**COMIDA QUE SU HIJO HA COMIDO (6-7)**		*180 gr*	

MÉTODO DE ELABORACIÓN:
Explicar detalladamente el proceso de elaboración del plato

FICHA INGREDIENTES/PESOS “PLATO HECHO EN CASA (HC)”

ID: MAD 08

DÍA DEL CONSUMO: 18 / 10 / 2014 **HORA DEL CONSUMO:** 16:15

MÉTODO DE ELABORACIÓN:

Explicar detalladamente el proceso de elaboración del plato:

Pelo la fruta.
Echo en el vaso de la batidora.
Troceo la galleta y la echo en el vaso de la batidora.
Trituro en la batidora y cuando esté lo sirvo.

Fotos:

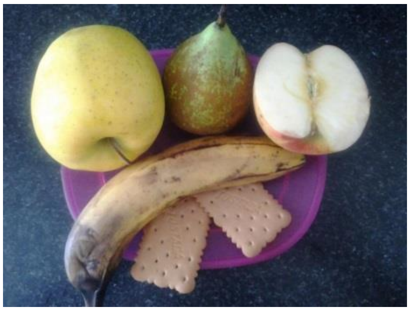

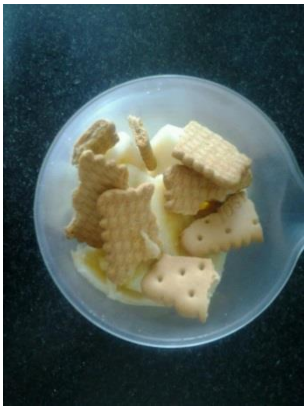

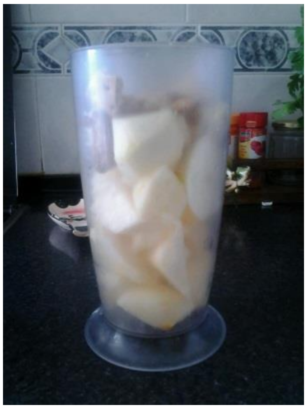

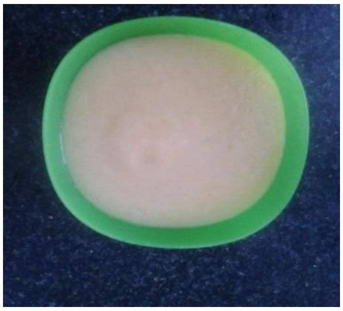

FICHA INGREDIENTES/PESOS “PLATO HECHO EN CASA (HC)” **ID: SEV 01**

DÍA DEL CONSUMO: *8 / 10 / 2014* HORA DEL CONSUMO: *14:00*

LA ELABOARACIÓN DEL PLATO SE HACE EL MISMO DÍA DEL CONSUMO [X] Sí [ ] No

Si la fecha de elaboración del plato NO es el mismo día del consumo NO se adjuntará foto y el peso de los ingredientes puede ser aproximado. Indique la fecha de elaboración: 8-10-14

TIPO DE PLATO: *PURÉ DE POLLO CON VERDURAS*

	Tipo de ingrediente	Nº	Peso (en gramos)
Ingrediente 1	*Sal*	*1*	*1 pizca*
Ingrediente 2	*Aceite de oliva*	*1*	*1 chorrito*
Ingrediente 3	*Zanahoria*	*4*	*401 gr*
Ingrediente 4	*Patata*	*3*	*238 gr*
Ingrediente 5	*Puerro*	*2*	*127 gr*
Ingrediente 6	*Judías verdes*	*5*	*70 gr*
Ingrediente 7	*Nabo*	*2*	*108 gr*
Ingrediente 8	*Pechuga de pollo*		*95 gr*
Ingrediente 9	*Calabacín*	*1*	*200 gr*
Ingrediente 10			

Proceso culinario	Nº	Peso (en gramos)	Observaciones
1. Olla VACÍA	*1*	*978 gr*	
2. Olla con COMIDA COCINADA	*1*	*2751 gr*	
**COMIDA COCINADA (2-1)**		*1773 gr*	
3. Fiambrera VACÍA	*12*	*9 gr*	*Relleno 12 fiambreras*
4. Fiambrera con COMIDA COCINADA		*154 gr*	
**COMIDA ALMACENADA (4-3)**		*145 gr*	
5. Plato VACÍO		*421 gr*	
6. Plato con COMIDA COCINADA		*532 gr*	
**COMIDA QUE SIRVE A SU HIJO (6-5)**		*111 gr*	
7. Plato COMIDA QUE SU HIJO NO HA COMIDO		*421 gr*	
**COMIDA QUE SU HIJO HA COMIDO (6-7)**		*111 gr*	

MÉTODO DE ELABORACIÓN:
Explicar detalladamente el proceso de elaboración del plato

FICHA INGREDIENTES/PESOS "PLATO HECHO EN CASA (HC)"

ID: SEV 01

DÍA DEL CONSUMO: 8 / 10 / 2014 **HORA DEL CONSUMO:** 14:00

MÉTODO DE ELABORACIÓN:

Explicar detalladamente el proceso de elaboración del plato:

Pelar las verduras, lavarlas y trocearlas.
Añadir todo a la olla junto con el pollo entero.
Añadir el agua hasta recubrirlo todo.
Sazonar con sal y echarle un chorro de aceite.
Dejar hervir 25 minutos.
Triturar todo salvo el nabo, que hay que tirarlo.
Añadirle según veamos agua de la cocción para que no quede muy espeso.

Fotos:

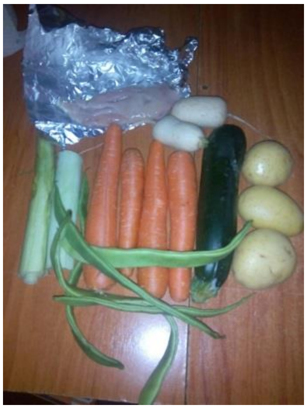

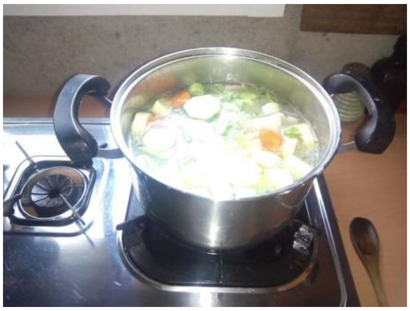

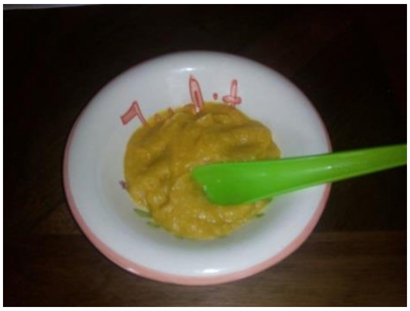

FICHA INGREDIENTES/PESOS "PLATO HECHO EN CASA (HC)" **ID: SEV 01**

DÍA DEL CONSUMO: *11 / 10 / 2014* HORA DEL CONSUMO: *14:45*

LA ELABOARACIÓN DEL PLATO SE HACE EL MISMO DÍA DEL CONSUMO ☐ Sí ☒ No

Si la fecha de elaboración del plato NO es el mismo día del consumo NO se adjuntará foto y el peso de los ingredientes puede ser aproximado. Indique la fecha de elaboración: 9-10-14

TIPO DE PLATO: *PURÉ DE VERDURAS CON MERLUZA*

	Tipo de ingrediente	Nº	Peso (en gramos)
Ingrediente 1	*Sal*	*1*	*1 pizca*
Ingrediente 2	*Aceite de oliva*	*1*	*1 chorrito*
Ingrediente 3	*Zanahoria*	*4*	*423 gr*
Ingrediente 4	*Patata*	*3*	*327 gr*
Ingrediente 5	*Puerro*	*2*	*102 gr*
Ingrediente 6	*Judías verdes*	*4*	*59 gr*
Ingrediente 7	*Nabo*	*2*	*99 gr*
Ingrediente 8	*Calabacín*	*1*	*230 gr*
Ingrediente 9	*Merluza*		*57 gr*
Ingrediente 10			

Proceso culinario	Nº	Peso (en gramos)	Observaciones
1. Olla VACÍA		*978 gr*	
2. Olla con COMIDA COCINADA		*2497 gr*	
**COMIDA COCINADA (2-1)**		*1519 gr*	
3. Fiambrera VACÍA	*10*	*9 gr*	*Relleno 10 fiambreras*
4. Fiambrera con COMIDA COCINADA		*143 gr*	
**COMIDA ALMACENADA (4-3)**		*134 gr*	
5. Plato VACÍO		*421 gr*	
6. Plato con COMIDA COCINADA		*558 gr*	
**COMIDA QUE SIRVE A SU HIJO (6-5)**		*137 gr*	
7. Plato COMIDA QUE SU HIJO NO HA COMIDO		*450 gr*	
**COMIDA QUE SU HIJO HA COMIDO (6-7)**		*108 gr*	

MÉTODO DE ELABORACIÓN:

Explicar detalladamente el proceso de elaboración del plato

FICHA INGREDIENTES/PESOS “PLATO HECHO EN CASA (HC)”

ID: SEV 01

DÍA DEL CONSUMO: 11 / 10 / 2014 **HORA DEL CONSUMO:** 14:45

MÉTODO DE ELABORACIÓN:

Explicar detalladamente el proceso de elaboración del plato:

Pelar las verduras, lavarlas y trocearlas.
Meterlo todo en la olla con la merluza y añadir agua.
Sazonar con sal y aceite.
Dejar hervir durante 20-25 minutos.
Retirar el nabo y batir todo lo demás.
Añadir agua de cocción si vemos que está espeso.

Fotos:

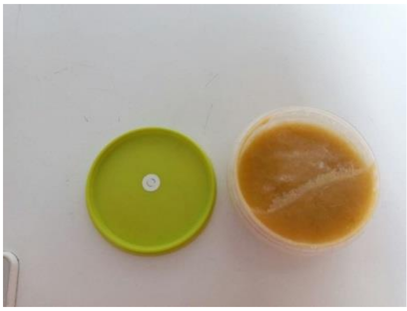

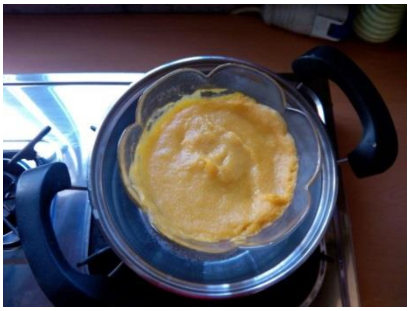

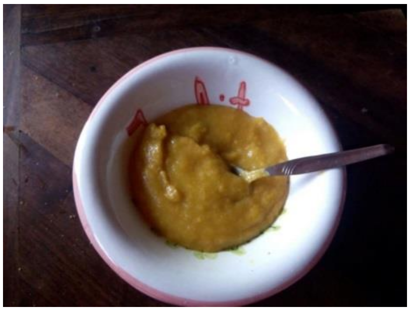

FICHA INGREDIENTES/PESOS "PLATO HECHO EN CASA (HC)" **ID: SEV 01**

DÍA DEL CONSUMO: *13/ 10 / 2014* HORA DEL CONSUMO: *13:45*

LA ELABOARACIÓN DEL PLATO SE HACE EL MISMO DÍA DEL CONSUMO [X] Sí [ ] No

Si la fecha de elaboración del plato NO es el mismo día del consumo NO se adjuntará foto y el peso de los ingredientes puede ser aproximado. Indique la fecha de elaboración: 13-10-14

TIPO DE PLATO: *PURÉ DE VERDURAS*

	Tipo de ingrediente	Nº	Peso (en gramos)
Ingrediente 1	*Aceite de oliva*		*1 chorrito*
Ingrediente 2	*Sal*		*1 pizca*
Ingrediente 3	*Calabacín*	*2*	*170 gr*
Ingrediente 4	*Tomate*	*1*	*108 gr*
Ingrediente 5	*Puerro*	*1*	*52 gr*
Ingrediente 6	*Zanahoria*	*1*	*108 gr*
Ingrediente 7	*Nata*		*1 chorrito*
Ingrediente 8			
Ingrediente 9			
Ingrediente 10			

Proceso culinario	Nº	Peso (en gramos)	Observaciones
1. Olla VACÍA		*978 gr*	
2. Olla con COMIDA COCINADA		*1620 gr*	
**COMIDA COCINADA (2-1)**		*642 gr*	
3. Fiambrera VACÍA	*4*	*9 gr*	*Relleno 4 fiambreras*
4. Fiambrera con COMIDA COCINADA		*152 gr*	
**COMIDA ALMACENADA (4-3)**		*143 gr*	
5. Plato VACÍO		*421 gr*	
6. Plato con COMIDA COCINADA		*533 gr*	
**COMIDA QUE SIRVE A SU HIJO (6-5)**		*112 gr*	
7. Plato COMIDA QUE SU HIJO NO HA COMIDO		*421 gr*	
**COMIDA QUE SU HIJO HA COMIDO (6-7)**		*112 gr*	

MÉTODO DE ELABORACIÓN:
Explicar detalladamente el proceso de elaboración del plato

FICHA INGREDIENTES/PESOS “PLATO HECHO EN CASA (HC)”

ID: SEV 01

DÍA DEL CONSUMO: 13 / 10 / 2014 **HORA DEL CONSUMO:** 13:45

MÉTODO DE ELABORACIÓN:

Explicar detalladamente el proceso de elaboración del plato:

Pelar las verduras, lavarlas y trocearlas.
Meter todo en la olla y cubrir todo con abundante agua.
Dejar hervir durante 20 minutos.
Batir todo y añadir un chorro de nata.

Fotos:

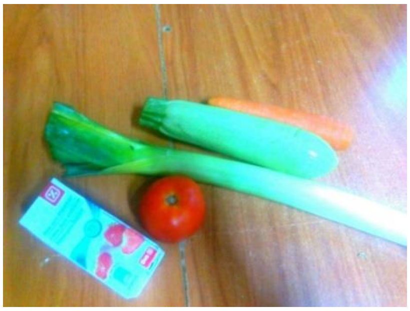

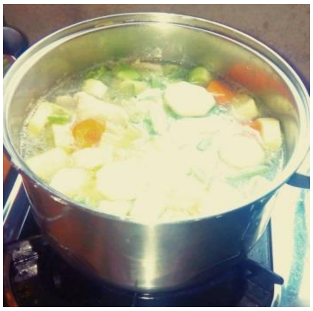

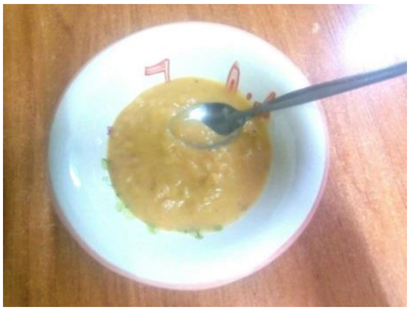

FICHA INGREDIENTES/PESOS "PLATO HECHO EN CASA (HC)" **ID: SEV 02**

DÍA DEL CONSUMO: *10 / 10 / 2014* HORA DEL CONSUMO: *15:17*

LA ELABOARACIÓN DEL PLATO SE HACE EL MISMO DÍA DEL CONSUMO [X] Sí [ ] No

Si la fecha de elaboración del plato NO es el mismo día del consumo NO se adjuntará foto y el peso de los ingredientes puede ser aproximado. Indique la fecha de elaboración: 10-10-14

TIPO DE PLATO: *PURÉ DE VERDURAS*

	Tipo de ingrediente	Nº	Peso (en gramos)
Ingrediente 1	*Patata*	*1*	*179 gr*
Ingrediente 2	*Zanahoria*	*2*	*339 gr*
Ingrediente 3	*Puerro*	*½*	*100 gr*
Ingrediente 4	*Judías verdes*	*6*	*79 gr*
Ingrediente 5	*Calabacín*	*1*	*347 gr*
Ingrediente 6	*Sal*	*1*	*1 pizca*
Ingrediente 7	*Aceite de oliva virgen extra*	*1*	*1 chorrito*
Ingrediente 8	*Calabaza*	*1*	*137 gr*
Ingrediente 9			
Ingrediente 10			

Proceso culinario	Nº	Peso (en gramos)	Observaciones
1. Olla VACÍA	*1*	*2008 gr*	
2. Olla con COMIDA COCINADA	*1*	*4242 gr*	
**COMIDA COCINADA (2-1)**		*2234 gr*	
3. Fiambrera VACÍA	*6*	*13 gr*	*Relleno 6 fiambreras*
4. Fiambrera con COMIDA COCINADA	*1*	*223 gr*	
**COMIDA ALMACENADA (4-3)**		*210 gr*	
5. Plato VACÍO		*152 gr*	
6. Plato con COMIDA COCINADA		*335 gr*	
**COMIDA QUE SIRVE A SU HIJO (6-5)**		*183 gr*	
7. Plato COMIDA QUE SU HIJO NO HA COMIDO		*228 gr*	
**COMIDA QUE SU HIJO HA COMIDO (6-7)**		*107 gr*	

MÉTODO DE ELABORACIÓN:
Explicar detalladamente el proceso de elaboración del plato

FICHA INGREDIENTES/PESOS "PLATO HECHO EN CASA (HC)" **ID: SEV 02**

DÍA DEL CONSUMO: 10 / 10 / 2014 **HORA DEL CONSUMO:** 15:17

MÉTODO DE ELABORACIÓN:
Explicar detalladamente el proceso de elaboración del plato:

Lavar muy bien todos los ingredientes.
Pelar y cortar todas las verduras.
Hervir todo durante 25-30 minutos.
Añadimos al agua un poco de sal y aceite.
Lo trituramos todo y le añadimos un poco del agua de cocción.

Fotos:

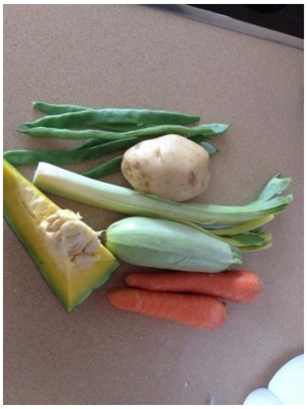

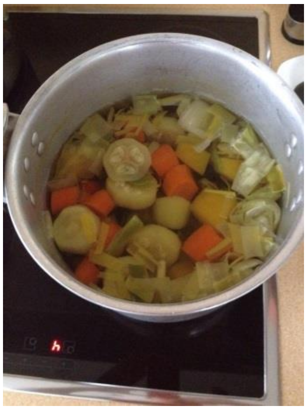

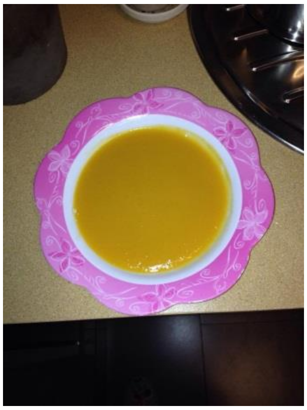

FICHA INGREDIENTES/PESOS "PLATO HECHO EN CASA (HC)" **ID: SEV 02**

DÍA DEL CONSUMO: *12 / 10 / 2014* HORA DEL CONSUMO: *15:00*

LA ELABOARACIÓN DEL PLATO SE HACE EL MISMO DÍA DEL CONSUMO [X] Sí [ ] No

Si la fecha de elaboración del plato NO es el mismo día del consumo NO se adjuntará foto y el peso de los ingredientes puede ser aproximado. Indique la fecha de elaboración: 12-10-14

TIPO DE PLATO: *PURÉ DE POLLO CON VERDURAS*

	Tipo de ingrediente	Nº	Peso (en gramos)
Ingrediente 1	*Patata*	*1*	*319 gr*
Ingrediente 2	*Zanahoria*	*2*	*296 gr*
Ingrediente 3	*Puerro*	*½*	*130 gr*
Ingrediente 4	*Judías verdes*	*6*	*91 gr*
Ingrediente 5	*Calabacín*	*1*	*278 gr*
Ingrediente 6	*Sal*	*1*	*1 pizca*
Ingrediente 7	*Aceite de oliva virgen extra*	*1*	*1 chorrito*
Ingrediente 8	*Calabaza*	*1*	*170 gr*
Ingrediente 9			
Ingrediente 10			

Proceso culinario	Nº	Peso (en gramos)	Observaciones
1. Olla VACÍA	*1*	*1443 gr*	
2. Olla con COMIDA COCINADA		*4309 gr*	
**COMIDA COCINADA (2-1)**		*2866 gr*	
3. Fiambrera VACÍA	*6*	*31 gr*	*Relleno 6 fiambreras*
4. Fiambrera con COMIDA COCINADA	*6*	*246 gr*	
**COMIDA ALMACENADA (4-3)**		*215 gr*	
5. Plato VACÍO		*26 gr*	
6. Plato con COMIDA COCINADA		*262 gr*	
**COMIDA QUE SIRVE A SU HIJO (6-5)**		*236 gr*	
7. Plato COMIDA QUE SU HIJO NO HA COMIDO		*109 gr*	
**COMIDA QUE SU HIJO HA COMIDO (6-7)**		*153 gr*	

MÉTODO DE ELABORACIÓN:
Explicar detalladamente el proceso de elaboración del plato

FICHA INGREDIENTES/PESOS "PLATO HECHO EN CASA (HC)" **ID: SEV 02**

DÍA DEL CONSUMO: 12 / 10 / 2014 **HORA DEL CONSUMO:** 15:00

MÉTODO DE ELABORACIÓN:

Explicar detalladamente el proceso de elaboración del plato:

Lavar muy bien todos los ingredientes.
Pelar y cortar todas las verduras.
Hervir todo durante 25-30 minutos.
Añadimos el agua un poco de sal y aceite.
Trituramos todo y añadimos un poco del agua de cocción.

Fotos:

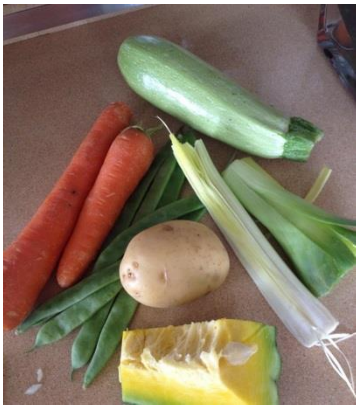

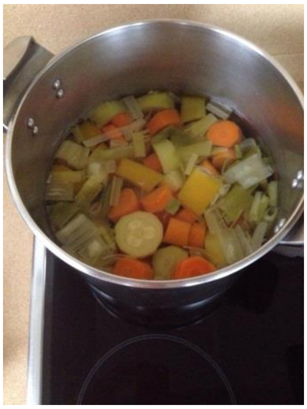

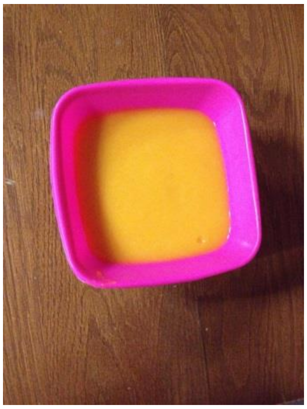

FICHA INGREDIENTES/PESOS "PLATO HECHO EN CASA (HC)" **ID: SEV 02**

DÍA DEL CONSUMO: *14 / 10 / 2014* HORA DEL CONSUMO: *15:15*

LA ELABOARACIÓN DEL PLATO SE HACE EL MISMO DÍA DEL CONSUMO ☐ Sí ☒ No

Si la fecha de elaboración del plato NO es el mismo día del consumo NO se adjuntará foto y el peso de los ingredientes puede ser aproximado. Indique la fecha de elaboración: 10-10-14

TIPO DE PLATO: *PURÉ DE VERDURAS*

	Tipo de ingrediente	Nº	Peso (en gramos)
Ingrediente 1	*Patata*	*1*	*179 gr*
Ingrediente 2	*Zanahoria*	*2*	*339 gr*
Ingrediente 3	*Puerro*	*½*	*100 gr*
Ingrediente 4	*Judías verdes*	*6*	*79 gr*
Ingrediente 5	*Calabacín*	*1*	*347 gr*
Ingrediente 6	*Sal*	*1*	*1 pizca*
Ingrediente 7	*Aceite de oliva virgen extra*	*1*	*1 chorro*
Ingrediente 8	*Calabaza*	*1*	*137 gr*
Ingrediente 9			
Ingrediente 10			

Proceso culinario	Nº	Peso (en gramos)	Observaciones
1. Olla VACÍA	*1*	*2008 gr*	
2. Olla con COMIDA COCINADA	*1*	*4242 gr*	
**COMIDA COCINADA (2-1)**		*2334 gr*	
3. Fiambrera VACÍA	*1*	*13 gr*	*Fiambrera descongelada primer día*
4. Fiambrera con COMIDA COCINADA	*1*	*223 gr*	
**COMIDA ALMACENADA (4-3)**		*210 gr*	
5. Plato VACÍO		*152 gr*	
6. Plato con COMIDA COCINADA		*335 gr*	
**COMIDA QUE SIRVE A SU HIJO (6-5)**		*183 gr*	
7. Plato COMIDA QUE SU HIJO NO HA COMIDO		*152 gr*	
**COMIDA QUE SU HIJO HA COMIDO (6-7)**		*183 gr*	

MÉTODO DE ELABORACIÓN:

Explicar detalladamente el proceso de elaboración del plato

FICHA INGREDIENTES/PESOS “PLATO HECHO EN CASA (HC)”

ID: SEV 02

DÍA DEL CONSUMO: 14/ 10 / 2014 **HORA DEL CONSUMO:** 15:15

MÉTODO DE ELABORACIÓN:

Explicar detalladamente el proceso de elaboración del plato:

ALIMENTO PREPARADO ANTERIORMENTE Y SACADO DEL CONGELADOR

Fotos:

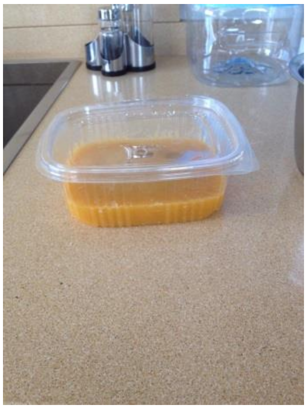

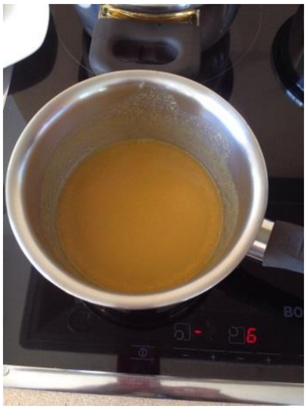

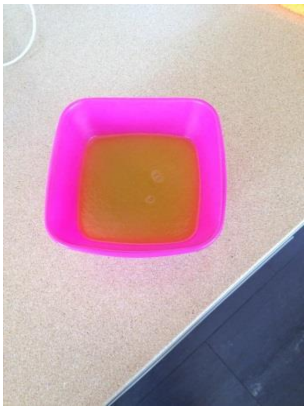

FICHA INGREDIENTES/PESOS "PLATO HECHO EN CASA (HC)" **ID: SEV 03**

DÍA DEL CONSUMO: *7 / 10 / 2014* HORA DEL CONSUMO: *14:30*

LA ELABOARACIÓN DEL PLATO SE HACE EL MISMO DÍA DEL CONSUMO ☒ Sí ☐ No

Si la fecha de elaboración del plato NO es el mismo día del consumo NO se adjuntará foto y el peso de los ingredientes puede ser aproximado. Indique la fecha de elaboración: 7-10-14

TIPO DE PLATO: *PURÉ DE VERDURAS CON POLLO*

	Tipo de ingrediente	Nº	Peso (en gramos)
Ingrediente 1	*Calabaza*	*1*	*107 gr*
Ingrediente 2	*Calabacín*	*1*	*207 gr*
Ingrediente 3	*Patata*	*2*	*295 gr*
Ingrediente 4	*Puerro*	*1*	*22 gr*
Ingrediente 5	*Zanahorias*	*2*	*175 gr*
Ingrediente 6	*Habichuelas*	*14*	*211 gr*
Ingrediente 7	*Apio*	*2*	*102 gr*
Ingrediente 8	*Tomate*	*1*	*76 gr*
Ingrediente 9	*Cebolla*	*1*	*72 gr*
Ingrediente 10	*Carne de pollo*	*1*	*325 gr*
Ingrediente 11	*Sal*		*1 pizca*
Ingrediente 12	*Aceite*		*1 chorrito*

Proceso culinario	Nº	Peso (en gramos)	Observaciones
1. Olla VACÍA	*1*	*1758 gr*	
2. Olla con COMIDA COCINADA	*1*	*3939 gr*	
**COMIDA COCINADA (2-1)**		*2181 gr*	
3. Fiambrera VACÍA	*8*	*10 gr*	
4. Fiambrera con COMIDA COCINADA	*1*	*201 gr*	*Relleno 8 fiambreras*
**COMIDA ALMACENADA (4-3)**		*191 gr*	
5. Plato VACÍO	*1*	*45 gr*	
6. Plato con COMIDA COCINADA	*1*	*235 gr*	
**COMIDA QUE SIRVE A SU HIJO (6-5)**		*190 gr*	
7. Plato COMIDA QUE SU HIJO NO HA COMIDO		*45 gr*	
**COMIDA QUE SU HIJO HA COMIDO (6-7)**		*190 gr*	

MÉTODO DE ELABORACIÓN:
Explicar detalladamente el proceso de elaboración del plato

FICHA INGREDIENTES/PESOS "PLATO HECHO EN CASA (HC)" **ID: SEV 03**

DÍA DEL CONSUMO: 7 / 10 / 2014 **HORA DEL CONSUMO:** 14:30

MÉTODO DE ELABORACIÓN:

Explicar detalladamente el proceso de elaboración del plato:

Enjuagar todas las verduras y el trozo de carne de pollo.
Pelar y cortar todas las verduras.
Trocear el pollo.
Echar todo en la olla con agua, con un chorrito de aceite y una pizca de sal.
Hervir todo durante unos 20 minutos y cuando esté tierno sacar todas las verduras y el pollo, se bate hasta tener una textura adecuada.
Una vez finalizado el proceso de trituración, se separa en tarritos adecuados para su posterior congelación.

Fotos:

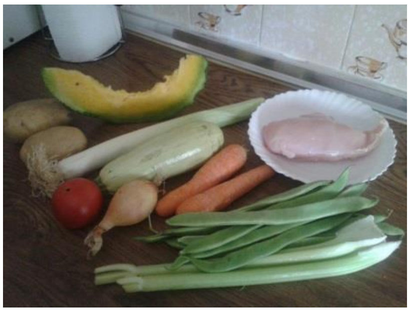

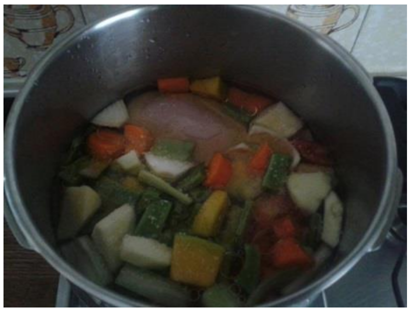

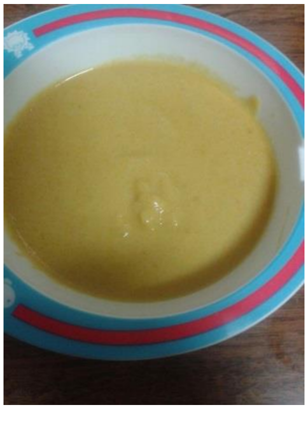

FICHA INGREDIENTES/PESOS "PLATO HECHO EN CASA (HC)" **ID: SEV 03**

DÍA DEL CONSUMO: *9 / 10 / 2014* HORA DEL CONSUMO: *14:35*

LA ELABOARACIÓN DEL PLATO SE HACE EL MISMO DÍA DEL CONSUMO [X] Sí [ ] No

Si la fecha de elaboración del plato NO es el mismo día del consumo NO se adjuntará foto y el peso de los ingredientes puede ser aproximado. Indique la fecha de elaboración: 9-10-14

TIPO DE PLATO: *PURÉ DE VERDURAS CON PESCADO*

	Tipo de ingrediente	Nº	Peso (en gramos)
Ingrediente 1	*Cebolla*	*1*	*190 gr*
Ingrediente 2	*Tomate*	*1*	*183 gr*
Ingrediente 3	*Patatas*	*2*	*622 gr*
Ingrediente 4	*Zanahorias*	*2*	*255 gr*
Ingrediente 5	*Puerro*	*1*	*165 gr*
Ingrediente 6	*Pescada-Merluza*	*2*	*247 gr*
Ingrediente 7	*Aceite de oliva*	*1*	*1 chorrito*
Ingrediente 8	*Sal*	*1*	*1 pizca*
Ingrediente 9			
Ingrediente 10			

Proceso culinario	Nº	Peso (en gramos)	Observaciones
1. Olla VACÍA	*1*	*1758 gr*	
2. Olla con COMIDA COCINADA	*1*	*3375 gr*	
**COMIDA COCINADA (2-1)**		*1617 gr*	
3. Fiambrera VACÍA	*1*	*10 gr*	*Relleno 7 fiambreras*
4. Fiambrera con COMIDA COCINADA	*7*	*201 gr*	
**COMIDA ALMACENADA (4-3)**		*191 gr*	
5. Plato VACÍO	*1*	*45 gr*	
6. Plato con COMIDA COCINADA	*1*	*234 gr*	
**COMIDA QUE SIRVE A SU HIJO (6-5)**		*189 gr*	
7. Plato COMIDA QUE SU HIJO NO HA COMIDO		*45 gr*	
**COMIDA QUE SU HIJO HA COMIDO (6-7)**		*189 gr*	

MÉTODO DE ELABORACIÓN:

Explicar detalladamente el proceso de elaboración del plato

FICHA INGREDIENTES/PESOS "PLATO HECHO EN CASA (HC)" **ID: SEV 03**

DÍA DEL CONSUMO: 9 / 10 / 2014 **HORA DEL CONSUMO:** 14:35

MÉTODO DE ELABORACIÓN:

Explicar detalladamente el proceso de elaboración del plato:

Se enjuagan todas las verduras y el pescado. Se le quita al pescado la piel.

Se trocean todas las verduras, una vez estén peladas.

Echar todas las verduras en la olla con agua, un chorrito de aceite y una pizca de sal.

Una vez estén tiernas las verduras, se le echa el pescado y posteriormente se sacan y se trituran.

Fotos:

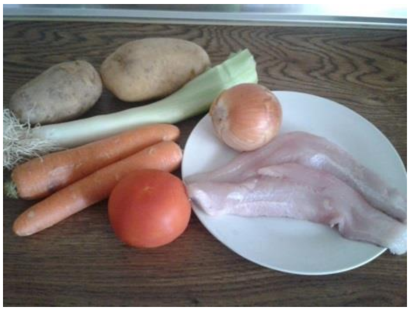

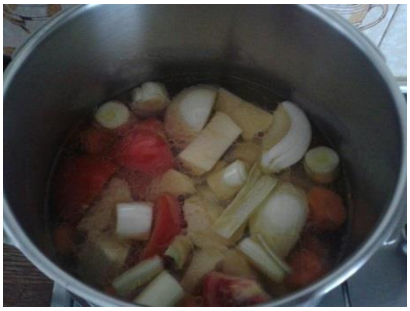

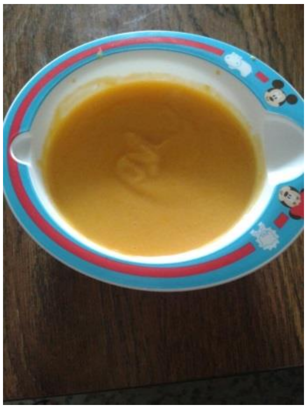

FICHA INGREDIENTES/PESOS “PLATO HECHO EN CASA (HC)” **ID: SEV 03**

DÍA DEL CONSUMO: *11 / 10 / 2014* HORA DEL CONSUMO: *14:30*

LA ELABOARACIÓN DEL PLATO SE HACE EL MISMO DÍA DEL CONSUMO ☐ Sí ☒ No

Si la fecha de elaboración del plato NO es el mismo día del consumo NO se adjuntará foto y el peso de los ingredientes puede ser aproximado. Indique la fecha de elaboración: 7-10-14

TIPO DE PLATO: *PURÉ DE VERDURAS CON POLLO*

	Tipo de ingrediente	Nº	Peso (en gramos)
Ingrediente 1	*Calabaza*	*1*	*107 gr*
Ingrediente 2	*Calabacín*	*1*	*207 gr*
Ingrediente 3	*Patatas*	*2*	*295 gr*
Ingrediente 4	*Puerros*	*1*	*22 gr*
Ingrediente 5	*Zanahorias*	*2*	*175 gr*
Ingrediente 6	*Habichuelas*	*14*	*211 gr*
Ingrediente 7	*Apio*	*2*	*102 gr*
Ingrediente 8	*Tomate*	*1*	*76 gr*
Ingrediente 9	*Cebolla*	*1*	*72 gr*
Ingrediente 10	*Carne de pollo*	*1*	*325 gr*
Ingrediente 11	*Sal*		*1 pizca*
Ingrediente 12	*Aceite*		*1 chorrito*

Proceso culinario	Nº	Peso (en gramos)	Observaciones
1. Olla VACÍA			
2. Olla con COMIDA COCINADA			
**COMIDA COCINADA (2-1)**			
3. Fiambrera VACÍA	*1*	*10 gr*	
4. Fiambrera con COMIDA COCINADA	*1*	*202 gr*	
**COMIDA ALMACENADA (4-3)**	*1*	*192 gr*	
5. Plato VACÍO	*1*	*45 gr*	
6. Plato con COMIDA COCINADA	*1*	*236 gr*	
**COMIDA QUE SIRVE A SU HIJO (6-5)**	*1*	*191 gr*	
7. Plato COMIDA QUE SU HIJO NO HA COMIDO		*45 gr*	
**COMIDA QUE SU HIJO HA COMIDO (6-7)**		*191 gr*	

MÉTODO DE ELABORACIÓN:
Explicar detalladamente el proceso de elaboración del plato

FICHA INGREDIENTES/PESOS "PLATO HECHO EN CASA (HC)"

ID: SEV 03

DÍA DEL CONSUMO: 11 / 10 / 2014 **HORA DEL CONSUMO:** 14:30

MÉTODO DE ELABORACIÓN:

Explicar detalladamente el proceso de elaboración del plato:

ALIMENTO PREPARADO ANTERIORMENTE Y SACADO DEL CONGELADOR

Fotos:

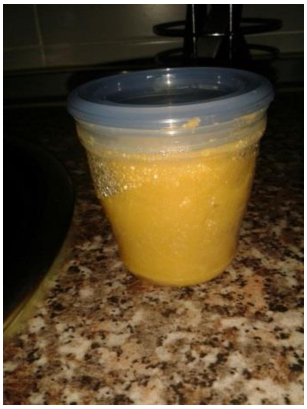

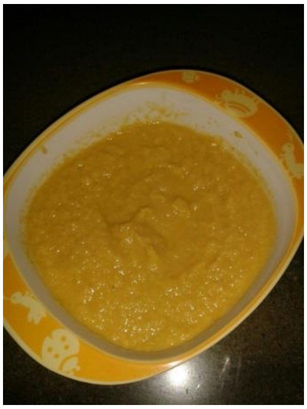

FICHA INGREDIENTES/PESOS "PLATO HECHO EN CASA (HC)" **ID: SEV 04**

DÍA DEL CONSUMO: *11 / 10 / 2014* HORA DEL CONSUMO: *14:00*

LA ELABOARACIÓN DEL PLATO SE HACE EL MISMO DÍA DEL CONSUMO [X] Sí [ ] No

Si la fecha de elaboración del plato NO es el mismo día del consumo NO se adjuntará foto y el peso de los ingredientes puede ser aproximado. Indique la fecha de elaboración: 11-10-14

TIPO DE PLATO: *PURÉ DE VERDURAS CON POLLO*

	Tipo de ingrediente	Nº	Peso (en gramos)
Ingrediente 1	*Aceite de oliva virgen extra*	*1*	*1 chorrito*
Ingrediente 2	*Patatas*	*1*	*128 gr*
Ingrediente 3	*Calabaza*	*1*	*184 gr*
Ingrediente 4	*Zanahoria*	*1*	*121 gr*
Ingrediente 5	*Puerro*	*1*	*66 gr*
Ingrediente 6	*Pechuga de pollo*	*½*	*108 gr*
Ingrediente 7	*Sal*	*1*	*1 pizca*
Ingrediente 8			
Ingrediente 9			
Ingrediente 10			

Proceso culinario	Nº	Peso (en gramos)	Observaciones
1. Olla VACÍA		*641 gr*	
2. Olla con COMIDA COCINADA		*1576 gr*	
**COMIDA COCINADA (2-1)**		*935 gr*	
3. Fiambrera VACÍA		*42 gr*	
4. Fiambrera con COMIDA COCINADA		*256 gr*	
**COMIDA ALMACENADA (4-3)**		*214 gr*	
5. Plato VACÍO		*296 gr*	
6. Plato con COMIDA COCINADA		*486 gr*	
**COMIDA QUE SIRVE A SU HIJO (6-5)**		*190 gr*	
7. Plato COMIDA QUE SU HIJO NO HA COMIDO		*302 gr*	*Peso del plato vacío después de comer*
**COMIDA QUE SU HIJO HA COMIDO (6-7)**		*184 gr*	

MÉTODO DE ELABORACIÓN:

Explicar detalladamente el proceso de elaboración del plato

FICHA INGREDIENTES/PESOS “PLATO HECHO EN CASA (HC)”

ID: SEV 04

DÍA DEL CONSUMO: 11 / 10 / 2014 **HORA DEL CONSUMO:** 14:00

MÉTODO DE ELABORACIÓN:

Explicar detalladamente el proceso de elaboración del plato:

Pelar y cortar las verduras.
Hervir durante 30 minutos y añadimos aceite y sal.
Triturar todo con un poco de agua de cocción.
Repartimos en fiambreras.

Fotos:

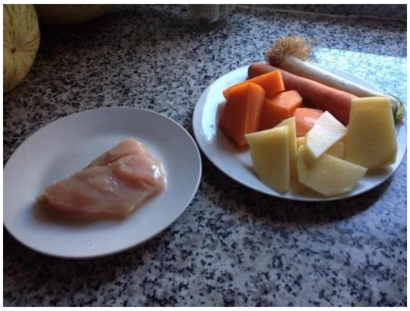

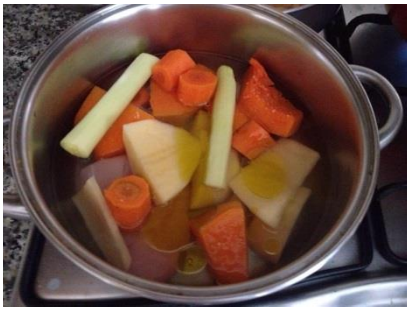

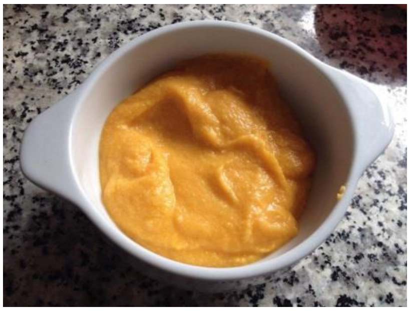

FICHA INGREDIENTES/PESOS "PLATO HECHO EN CASA (HC)" **ID: SEV 04**

DÍA DEL CONSUMO: *11 / 10 / 2014* HORA DEL CONSUMO: *17:50*

LA ELABOARACIÓN DEL PLATO SE HACE EL MISMO DÍA DEL CONSUMO [X] Sí [ ] No

Si la fecha de elaboración del plato NO es el mismo día del consumo NO se adjuntará foto y el peso de los ingredientes puede ser aproximado. Indique la fecha de elaboración: 11-10-14

TIPO DE PLATO: *PAPILLA DE FRUTAS*

	Tipo de ingrediente	Nº	Peso (en gramos)
Ingrediente 1	*Plátano*	*1*	*144 gr*
Ingrediente 2	*Manzana*	*1*	*113 gr*
Ingrediente 3	*Melocotón*	*1*	*214 gr*
Ingrediente 4			
Ingrediente 5			
Ingrediente 6			
Ingrediente 7			
Ingrediente 8			
Ingrediente 9			
Ingrediente 10			

Proceso culinario	Nº	Peso (en gramos)	Observaciones
1. Olla VACÍA			
2. Olla con COMIDA COCINADA			
**COMIDA COCINADA (2-1)**			
3. Fiambrera VACÍA			
4. Fiambrera con COMIDA COCINADA			
**COMIDA ALMACENADA (4-3)**			
5. Plato VACÍO		*297 gr*	
6. Plato con COMIDA COCINADA		*711 gr*	
**COMIDA QUE SIRVE A SU HIJO (6-5)**		*414 gr*	
7. Plato COMIDA QUE SU HIJO NO HA COMIDO		*560 gr*	
**COMIDA QUE SU HIJO HA COMIDO (6-7)**		*151 gr*	

MÉTODO DE ELABORACIÓN:
Explicar detalladamente el proceso de elaboración del plato

FICHA INGREDIENTES/PESOS "PLATO HECHO EN CASA (HC)" **ID: SEV 04**

DÍA DEL CONSUMO: 11 / 10 / 2014 **HORA DEL CONSUMO:** 17:50

MÉTODO DE ELABORACIÓN:
Explicar detalladamente el proceso de elaboración del plato:

Triturar la fruta y pasar al plato.

Fotos:

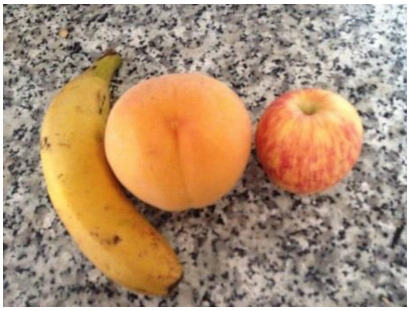

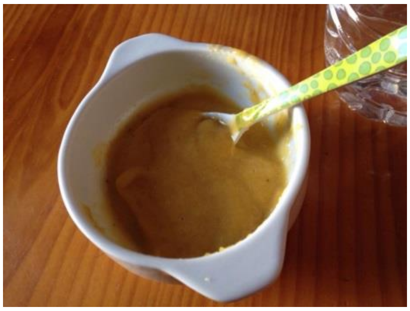

FICHA INGREDIENTES/PESOS “PLATO HECHO EN CASA (HC)” **ID: SEV 05**

DÍA DEL CONSUMO: *10 / 10 / 2014* HORA DEL CONSUMO: *16:00*

LA ELABOARACIÓN DEL PLATO SE HACE EL MISMO DÍA DEL CONSUMO [X] Sí [ ] No

Si la fecha de elaboración del plato NO es el mismo día del consumo NO se adjuntará foto y el peso de los ingredientes puede ser aproximado. Indique la fecha de elaboración: 10-10-14

TIPO DE PLATO: *PAPILLA DE FRUTAS*

	Tipo de ingrediente	Nº	Peso (en gramos)
Ingrediente 1	*Plátano*	*1*	*160 gr*
Ingrediente 2	*Naranja*	*1*	*139 gr*
Ingrediente 3	*Peras*	*2*	*359 gr*
Ingrediente 4			
Ingrediente 5			
Ingrediente 6			
Ingrediente 7			
Ingrediente 8			
Ingrediente 9			
Ingrediente 10			

Proceso culinario	Nº	Peso (en gramos)	Observaciones
1. Olla VACÍA			
2. Olla con COMIDA COCINADA			
**COMIDA COCINADA (2-1)**			
3. Fiambrera VACÍA			
4. Fiambrera con COMIDA COCINADA			
**COMIDA ALMACENADA (4-3)**			
5. Plato VACÍO	*1*	*110 gr*	
6. Plato con COMIDA COCINADA	*1*	*404 gr*	
**COMIDA QUE SIRVE A SU HIJO (6-5)**	*1*	*294 gr*	
7. Plato COMIDA QUE SU HIJO NO HA COMIDO	*1*	*200 gr*	
**COMIDA QUE SU HIJO HA COMIDO (6-7)**	*1*	*204 gr*	

MÉTODO DE ELABORACIÓN:
Explicar detalladamente el proceso de elaboración del plato

FICHA INGREDIENTES/PESOS "PLATO HECHO EN CASA (HC)" **ID: SEV 05**

DÍA DEL CONSUMO: 10 / 10 / 2014 **HORA DEL CONSUMO:** 16:00

MÉTODO DE ELABORACIÓN:
Explicar detalladamente el proceso de elaboración del plato:

Se lavan las peras y la naranja.
Pelo el plátano, lo parto en pedacitos.
Pelo las peras y se cortan a trocitos sin el centro.
Exprimo la naranja.
Todo en un vaso lo batimos muy bien en la batidora.

Fotos:

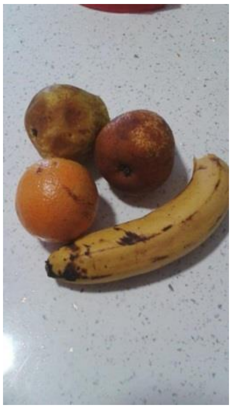

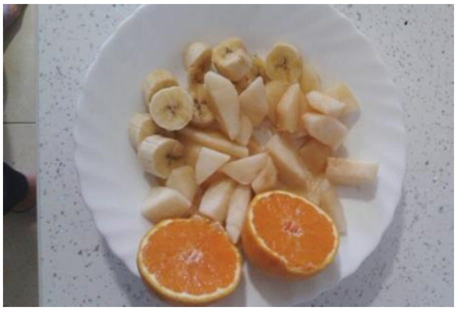

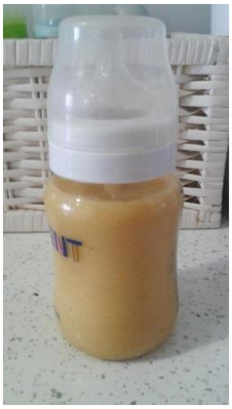

FICHA INGREDIENTES/PESOS "PLATO HECHO EN CASA (HC)" **ID: SEV 05**

DÍA DEL CONSUMO: *12 / 10 / 2014* HORA DEL CONSUMO: *12:50*

LA ELABOARACIÓN DEL PLATO SE HACE EL MISMO DÍA DEL CONSUMO [X] Sí [ ] No

Si la fecha de elaboración del plato NO es el mismo día del consumo NO se adjuntará foto y el peso de los ingredientes puede ser aproximado. Indique la fecha de elaboración: 12-10-14

TIPO DE PLATO: *PURÉ DE VERDURAS CON PAVO*

	Tipo de ingrediente	Nº	Peso (en gramos)
Ingrediente 1	*Calabacín*	*3*	*863 gr*
Ingrediente 2	*Zanahoria*	*3*	*292 gr*
Ingrediente 3	*Apio verde*	*3*	*115 gr*
Ingrediente 4	*Cebolla*	*1*	*195 gr*
Ingrediente 5	*Calabaza*	*1*	*344 gr*
Ingrediente 6	*Patatas*	*2*	*317 gr*
Ingrediente 7	*Tomate*	*1*	*151 gr*
Ingrediente 8	*Agua*		*1,766 ml*
Ingrediente 9	*Pavo*		*600 gr*
Ingrediente 10	*Aceite*		*150 ml*

Proceso culinario	Nº	Peso (en gramos)	Observaciones
1. Olla VACÍA		*731 gr*	
2. Olla con COMIDA COCINADA		*3185 gr*	
**COMIDA COCINADA (2-1)**		*2454 gr*	
3. Fiambrera VACÍA	*1*	*13 gr*	
4. Fiambrera con COMIDA COCINADA	*1*	*272 gr*	
**COMIDA ALMACENADA (4-3)**	*9*	*259 gr*	*Relleno 9 fiambreras*
5. Plato VACÍO	*1*	*110 gr*	
6. Plato con COMIDA COCINADA	*1*	*342 gr*	
**COMIDA QUE SIRVE A SU HIJO (6-5)**	*1*	*232 gr*	
7. Plato COMIDA QUE SU HIJO NO HA COMIDO	*1*	*148 gr*	
**COMIDA QUE SU HIJO HA COMIDO (6-7)**	*1*	*194 gr*	

MÉTODO DE ELABORACIÓN:

Explicar detalladamente el proceso de elaboración del plato

FICHA INGREDIENTES/PESOS "PLATO HECHO EN CASA (HC)"

ID: SEV 05

DÍA DEL CONSUMO: 12 / 10 / 2014 **HORA DEL CONSUMO:** 12:50

MÉTODO DE ELABORACIÓN:

Explicar detalladamente el proceso de elaboración del plato:

Lavar todas las verduras.
Pelar y trocear las verduras.
Hervir durante 30 minutos.
Agregar el pavo troceado.
Hervir durante 15 minutos más.
Agregar aceite y triturar todo con la batidora.

Fotos:

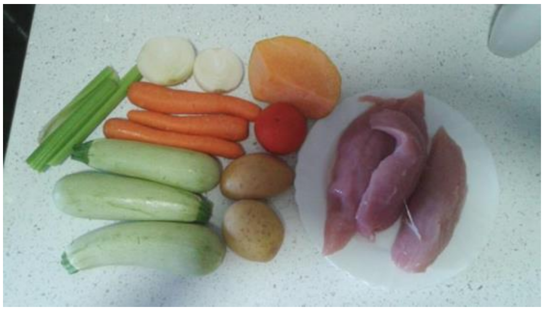

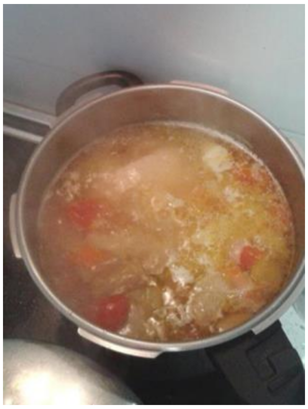

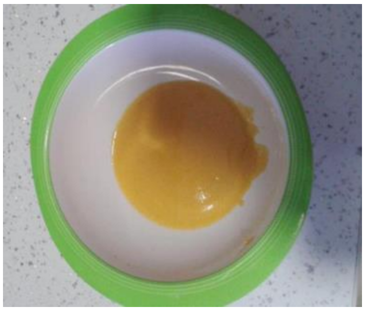

FICHA INGREDIENTES/PESOS “PLATO HECHO EN CASA (HC)” **ID: SEV 06**

DÍA DEL CONSUMO: *9 / 10 / 2014* HORA DEL CONSUMO: *13:45*

LA ELABOARACIÓN DEL PLATO SE HACE EL MISMO DÍA DEL CONSUMO [X] Sí [ ] No

Si la fecha de elaboración del plato NO es el mismo día del consumo NO se adjuntará foto y el peso de los ingredientes puede ser aproximado. Indique la fecha de elaboración: 9-10-14

TIPO DE PLATO: *POLLO CON VERDURAS*

	Tipo de ingrediente	Nº	Peso (en gramos)
Ingrediente 1	*Puerro y cebolla*	*1 Y ½*	*237 gr*
Ingrediente 2	*Judías verdes*	*20*	*230 gr*
Ingrediente 3	*Calabacín*	*1*	*327 gr*
Ingrediente 4	*Calabaza*	*1 trozo*	*180 gr*
Ingrediente 5	*Pollo*	*¼*	*252 gr*
Ingrediente 6	*Zanahoria*	*3*	*175 gr*
Ingrediente 7	*Papas*	*2*	*250 gr*
Ingrediente 8	*Apio*	*1 punta*	*80 gr*
Ingrediente 9	*Tomate*	*1*	*80 gr*
Ingrediente 10	*Pimiento*	*1*	*45 gr*

Proceso culinario	Nº	Peso (en gramos)	Observaciones
1. Olla VACÍA		*1550 gr*	
2. Olla con COMIDA COCINADA		*3570 gr*	
**COMIDA COCINADA (2-1)**		*2020 gr*	*Sin triturar*
3. Fiambrera VACÍA		*80 gr*	
4. Fiambrera con COMIDA COCINADA		*1115 gr*	*Triturada*
**COMIDA ALMACENADA (4-3)**		*1035 gr*	
5. Plato VACÍO		*51 gr*	
6. Plato con COMIDA COCINADA		*245 gr*	
**COMIDA QUE SIRVE A SU HIJO (6-5)**		*194 gr*	
7. Plato COMIDA QUE SU HIJO NO HA COMIDO		*51 gr*	
**COMIDA QUE SU HIJO HA COMIDO (6-7)**		*194 gr*	

MÉTODO DE ELABORACIÓN:
Explicar detalladamente el proceso de elaboración del plato

FICHA INGREDIENTES/PESOS "PLATO HECHO EN CASA (HC)"

ID: SEV 06

DÍA DEL CONSUMO: 9 / 10 / 2014 **HORA DEL CONSUMO:** 13:45

MÉTODO DE ELABORACIÓN:

Explicar detalladamente el proceso de elaboración del plato:

Pelar y cortar las verduras.
Trocear el pollo.
Se mete todo en la olla y se deja hervir hasta que las zanahorias estén tiernas, con aceite y un poco de sal.
Luego sacamos el caldo y trituramos la comida.

Fotos:

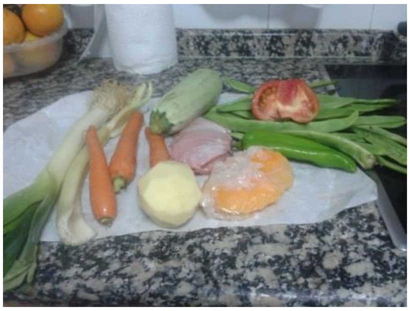

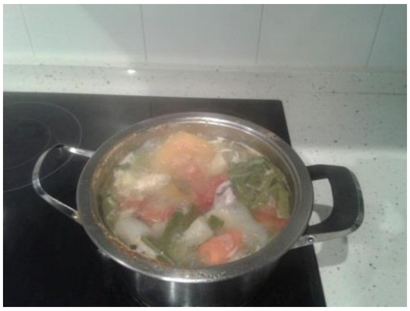

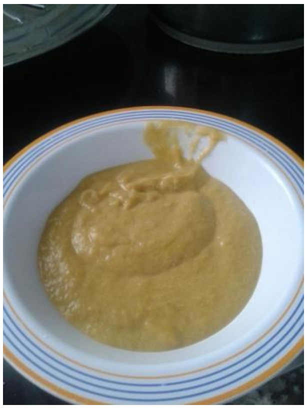

FICHA INGREDIENTES/PESOS "PLATO HECHO EN CASA (HC)" **ID: SEV 06**

DÍA DEL CONSUMO: *13 / 10 / 2014* HORA DEL CONSUMO: *13:50*

LA ELABOARACIÓN DEL PLATO SE HACE EL MISMO DÍA DEL CONSUMO ☐ Sí ☒ No

Si la fecha de elaboración del plato NO es el mismo día del consumo NO se adjuntará foto y el peso de los ingredientes puede ser aproximado. Indique la fecha de elaboración: 8-10-14

TIPO DE PLATO: *PURÉ DE VERDURAS Y PESCADO*

	Tipo de ingrediente	Nº	Peso (en gramos)
Ingrediente 1	*Zanahoria*		
Ingrediente 2	*Patata*		
Ingrediente 3	*Tomate*		
Ingrediente 4	*Pimiento*		
Ingrediente 5	*Cebolla*		
Ingrediente 6	*Merluza*		
Ingrediente 7			
Ingrediente 8			
Ingrediente 9			
Ingrediente 10			

Proceso culinario	Nº	Peso (en gramos)	Observaciones
1. Olla VACÍA			
2. Olla con COMIDA COCINADA			
**COMIDA COCINADA (2-1)**			
3. Fiambrera VACÍA			
4. Fiambrera con COMIDA COCINADA			
**COMIDA ALMACENADA (4-3)**			
5. Plato VACÍO		*51 gr*	
6. Plato con COMIDA COCINADA		*271 gr*	
**COMIDA QUE SIRVE A SU HIJO (6-5)**		*220 gr*	
7. Plato COMIDA QUE SU HIJO NO HA COMIDO		*106 gr*	
**COMIDA QUE SU HIJO HA COMIDO (6-7)**		*165 gr*	

MÉTODO DE ELABORACIÓN:
Explicar detalladamente el proceso de elaboración del plato

FICHA INGREDIENTES/PESOS "PLATO HECHO EN CASA (HC)"

ID: SEV 06

DÍA DEL CONSUMO: 13 / 10 / 2014 **HORA DEL CONSUMO:** 13:50

MÉTODO DE ELABORACIÓN:

Explicar detalladamente el proceso de elaboración del plato:

Pelar y cortas las verduras.
Meter en la olla todo.
Hervir.
Separar caldo que usaremos en otra comida con pasta.
Triturar el resto.

Fotos:

ALIMENTO PREPARADO ANTERIORMENTE Y SACADO DEL CONGELADOR

FICHA INGREDIENTES/PESOS "PLATO HECHO EN CASA (HC)" **ID: SEV 07**

DÍA DEL CONSUMO: *12 / 10 / 2014* **HORA DEL CONSUMO:** *13:45*

LA ELABOARACIÓN DEL PLATO SE HACE EL MISMO DÍA DEL CONSUMO ☐ Sí ☒ No

Si la fecha de elaboración del plato NO es el mismo día del consumo NO se adjuntará foto y el peso de los ingredientes puede ser aproximado. Indique la fecha de elaboración: Jueves 2-10-14

TIPO DE PLATO: *PURÉ DE VERDURAS Y PESCADO*

	Tipo de ingrediente	Nº	Peso (en gramos)
Ingrediente 1	*Puerro*	*1*	*50 gr*
Ingrediente 2	*Zanahorias*	*3*	*290 gr*
Ingrediente 3	*Patatas*	*2*	*350 gr*
Ingrediente 4	*Calabaza*	*1*	*150 gr*
Ingrediente 5	*Calabacín*	*2*	*520 gr*
Ingrediente 6	*Pescado (merluza)*	*1*	*400-500 gr*
Ingrediente 7			
Ingrediente 8			
Ingrediente 9			
Ingrediente 10			

Proceso culinario	Nº	Peso (en gramos)	Observaciones
1. Olla VACÍA			
2. Olla con COMIDA COCINADA			
**COMIDA COCINADA (2-1)**			
3. Fiambrera VACÍA	*1*	*41 gr*	
4. Fiambrera con COMIDA COCINADA	*1*	*280 gr*	
**COMIDA ALMACENADA (4-3)**		*239 gr*	
5. Plato VACÍO		*41 gr*	*Fiambrera (no usa plato)*
6. Plato con COMIDA COCINADA		*280 gr*	
**COMIDA QUE SIRVE A SU HIJO (6-5)**	*1*	*239 gr*	
7. Plato COMIDA QUE SU HIJO NO HA COMIDO	*1*	*80 gr*	
**COMIDA QUE SU HIJO HA COMIDO (6-7)**		*200 gr*	

MÉTODO DE ELABORACIÓN:
Explicar detalladamente el proceso de elaboración del plato

FICHA INGREDIENTES/PESOS "PLATO HECHO EN CASA (HC)"

ID: SEV 07

DÍA DEL CONSUMO: 12 / 10 / 2014 **HORA DEL CONSUMO:** 13:45

MÉTODO DE ELABORACIÓN:
Explicar detalladamente el proceso de elaboración del plato:

Pelar y cortar las verduras.
Echar el pescado.
Hervir todo en la olla rápida 6 minutos.
Batir y hacer cacitos con 250 gr.
Congelar.

Fotos:

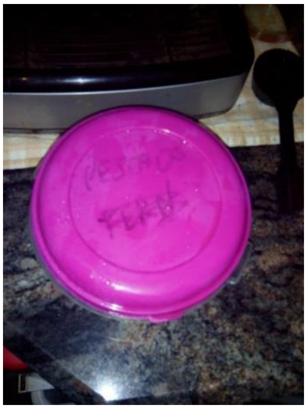

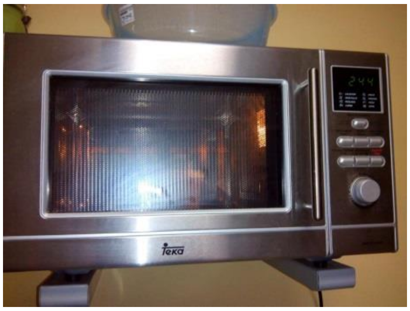

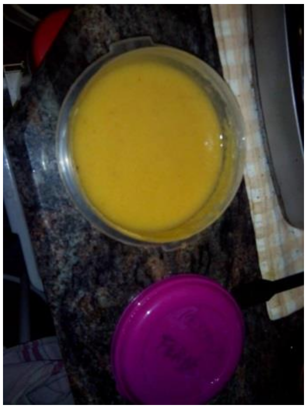

FICHA INGREDIENTES/PESOS “PLATO HECHO EN CASA (HC)” **ID: SEV 07**

DÍA DEL CONSUMO: *16 / 10 / 2014* HORA DEL CONSUMO: *13:45*

LA ELABOARACIÓN DEL PLATO SE HACE EL MISMO DÍA DEL CONSUMO ☐ Sí ☒ No

Si la fecha de elaboración del plato NO es el mismo día del consumo NO se adjuntará foto y el peso de los ingredientes puede ser aproximado. Indique la fecha de elaboración: Jueves 2-10-14

TIPO DE PLATO: *PURÉ DE VERDURAS Y POLLO*

	Tipo de ingrediente	Nº	Peso (en gramos)
Ingrediente 1	*Puerros*	*1*	*70 gr*
Ingrediente 2	*Zanahorias*	*3*	*250 gr*
Ingrediente 3	*Patatas*	*2*	*360 gr*
Ingrediente 4	*Calabaza*	*1*	*170 gr*
Ingrediente 5	*Calabacín*	*3*	*750 gr*
Ingrediente 6	*Pollo*	*1*	*500 gr*
Ingrediente 7			
Ingrediente 8			
Ingrediente 9			
Ingrediente 10			

Proceso culinario	Nº	Peso (en gramos)	Observaciones
1. Olla VACÍA			
2. Olla con COMIDA COCINADA			
**COMIDA COCINADA (2-1)**			
3. Fiambrera VACÍA	*1*	*41 gr*	
4. Fiambrera con COMIDA COCINADA	*1*	*280 gr*	
**COMIDA ALMACENADA (4-3)**		*239 gr*	
5. Plato VACÍO		*41 gr*	*Fiambrera*
6. Plato con COMIDA COCINADA		*280 gr*	
**COMIDA QUE SIRVE A SU HIJO (6-5)**	*1*	*239 gr*	
7. Plato COMIDA QUE SU HIJO NO HA COMIDO	*1*	*71 gr*	
**COMIDA QUE SU HIJO HA COMIDO (6-7)**		*209 gr*	

MÉTODO DE ELABORACIÓN:
Explicar detalladamente el proceso de elaboración del plato

FICHA INGREDIENTES/PESOS "PLATO HECHO EN CASA (HC)" **ID: SEV 07**

DÍA DEL CONSUMO: 13 / 10 / 2014 **HORA DEL CONSUMO:** 13:45

MÉTODO DE ELABORACIÓN:

Explicar detalladamente el proceso de elaboración del plato:

Pelar y cortar las verduras.
Echar el pollo.
Hervir todo en la olla rápida 10 minutos.
Batir y hacer cacitos con 250 gr.
Congelar.

Fotos:

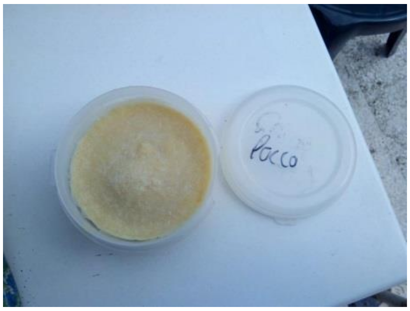

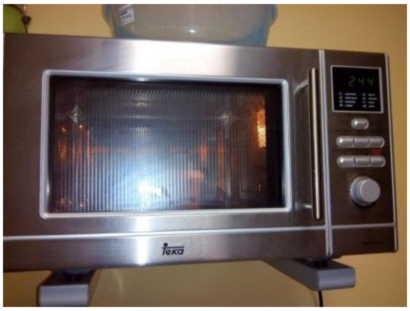

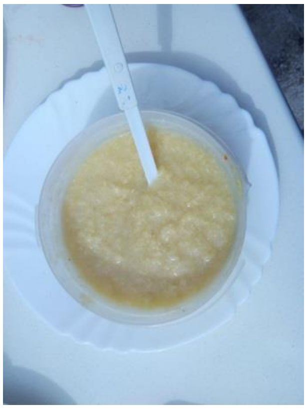

FICHA INGREDIENTES/PESOS “PLATO HECHO EN CASA (HC)” **ID: SEV 07**

DÍA DEL CONSUMO: *13 / 10 / 2014* **HORA DEL CONSUMO:** *18:30*

LA ELABOARACIÓN DEL PLATO SE HACE EL MISMO DÍA DEL CONSUMO [X] Sí [ ] No

Si la fecha de elaboración del plato NO es el mismo día del consumo NO se adjuntará foto y el peso de los ingredientes puede ser aproximado. Indique la fecha de elaboración:

TIPO DE PLATO: *PAPILLA DE FRUTAS*

	Tipo de ingrediente	Nº	Peso (en gramos)
Ingrediente 1	*Plátanos*	*2*	*181 gr*
Ingrediente 2	*Manzana*	*1*	*304 gr*
Ingrediente 3	*Galletas*	*2*	*14 gr*
Ingrediente 4			
Ingrediente 5			
Ingrediente 6			
Ingrediente 7			
Ingrediente 8			
Ingrediente 9			
Ingrediente 10			

Proceso culinario	Nº	Peso (en gramos)	Observaciones
1. Olla VACÍA			
2. Olla con COMIDA COCINADA			
**COMIDA COCINADA (2-1)**			
3. Fiambrera VACÍA	*1*	*43 gr*	
4. Fiambrera con COMIDA COCINADA	*1*	*250 gr*	
**COMIDA ALMACENADA (4-3)**		*207 gr*	
5. Plato VACÍO		*43 gr*	*Fiambrera*
6. Plato con COMIDA COCINADA		*250 gr*	
**COMIDA QUE SIRVE A SU HIJO (6-5)**	*1*	*207 gr*	
7. Plato COMIDA QUE SU HIJO NO HA COMIDO	*1*	*93 gr*	
**COMIDA QUE SU HIJO HA COMIDO (6-7)**		*157 gr*	

MÉTODO DE ELABORACIÓN:
Explicar detalladamente el proceso de elaboración del plato

FICHA INGREDIENTES/PESOS “PLATO HECHO EN CASA (HC)” **ID: SEV 07**

DÍA DEL CONSUMO: 13 / 10 / 2014 **HORA DEL CONSUMO:** 18:30

MÉTODO DE ELABORACIÓN:
Explicar detalladamente el proceso de elaboración del plato:

Pelar la manzana y los plátanos.
Trocear.
Echar galletas troceadas.
Batirlo todo.

Fotos:

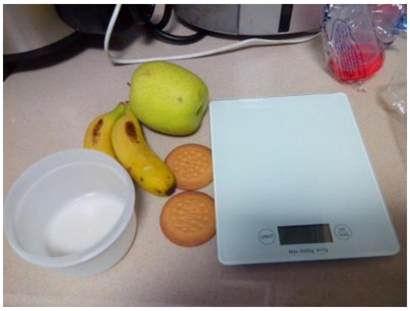

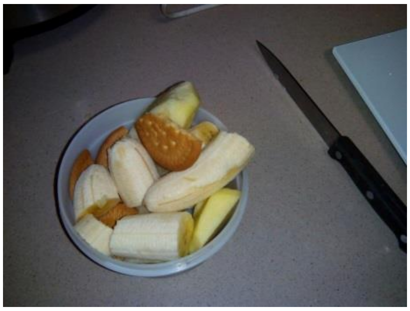

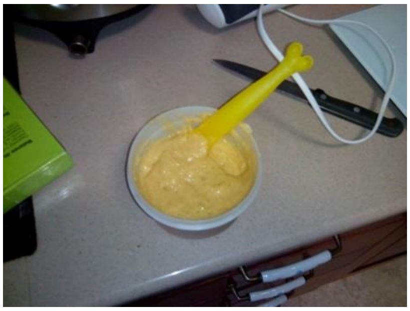

FICHA INGREDIENTES/PESOS “PLATO HECHO EN CASA (HC)” **ID: SEV 07**

DÍA DEL CONSUMO: *14 / 10 / 2014* HORA DEL CONSUMO: *14:45*

LA ELABOARACIÓN DEL PLATO SE HACE EL MISMO DÍA DEL CONSUMO ☐ Sí ☒ No

Si la fecha de elaboración del plato NO es el mismo día del consumo NO se adjuntará foto y el peso de los ingredientes puede ser aproximado. Indique la fecha de elaboración: Jueves 2-10-14

TIPO DE PLATO: *PURÉ DE VERDURAS Y TERNERA*

	Tipo de ingrediente	Nº	Peso (en gramos)
Ingrediente 1	*Puerros*	*1*	*75 gr*
Ingrediente 2	*Zanahoria*	*3*	*240 gr*
Ingrediente 3	*Patata*	*2*	*350 gr*
Ingrediente 4	*Calabaza*	*1*	*165 gr*
Ingrediente 5	*Calabacín*	*3*	*720 gr*
Ingrediente 6	*Ternera*	*1*	*450 gr*
Ingrediente 7			
Ingrediente 8			
Ingrediente 9			
Ingrediente 10			

Proceso culinario	Nº	Peso (en gramos)	Observaciones
1. Olla VACÍA			
2. Olla con COMIDA COCINADA			
**COMIDA COCINADA (2-1)**			
3. Fiambrera VACÍA	*1*	*40 gr*	
4. Fiambrera con COMIDA COCINADA	*1*	*280 gr*	
**COMIDA ALMACENADA (4-3)**		*240 gr*	
5. Plato VACÍO		*40 gr*	
6. Plato con COMIDA COCINADA		*280 gr*	
**COMIDA QUE SIRVE A SU HIJO (6-5)**	*1*	*240 gr*	
7. Plato COMIDA QUE SU HIJO NO HA COMIDO		*40 gr*	
**COMIDA QUE SU HIJO HA COMIDO (6-7)**		*240 gr*	

MÉTODO DE ELABORACIÓN:
Explicar detalladamente el proceso de elaboración del plato

FICHA INGREDIENTES/PESOS "PLATO HECHO EN CASA (HC)" **ID: SEV 07**

DÍA DEL CONSUMO: 14 / 10 / 2014 **HORA DEL CONSUMO:** 14:45

MÉTODO DE ELABORACIÓN:
Explicar detalladamente el proceso de elaboración del plato:

Pelar y cortar las verduras.
Echar la ternera.
Hervir todo en la olla rápida durante 12 minutos.
Batir y hacer cacitos con 250 gr.
Congelar.

Fotos:

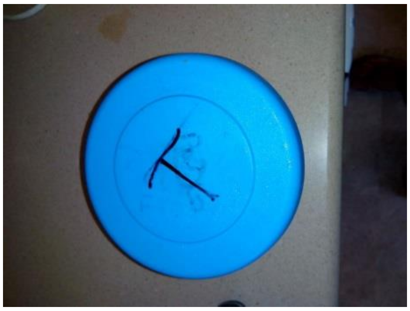

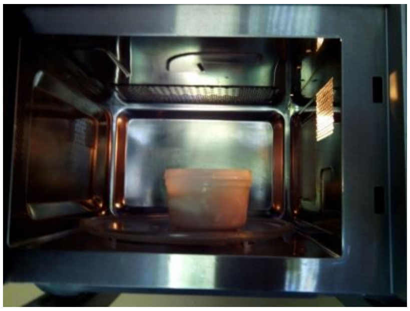

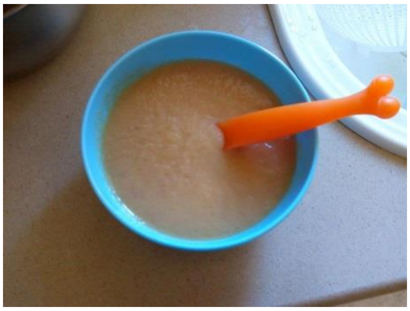

FICHA INGREDIENTES/PESOS "PLATO HECHO EN CASA (HC)" **ID: SEV 07**

DÍA DEL CONSUMO: *14 / 10 / 2014* HORA DEL CONSUMO: *18:40*

LA ELABOARACIÓN DEL PLATO SE HACE EL MISMO DÍA DEL CONSUMO [X] Sí [ ] No

Si la fecha de elaboración del plato NO es el mismo día del consumo NO se adjuntará foto y el peso de los ingredientes puede ser aproximado. Indique la fecha de elaboración: Jueves 14-10-14

TIPO DE PLATO: *PAPILLA DE FRUTAS*

	Tipo de ingrediente	Nº	Peso (en gramos)
Ingrediente 1	*Plátano*	*2*	*50 gr*
Ingrediente 2	*Pera*	*1*	*65 gr*
Ingrediente 3	*Galletas*	*2*	*14 gr*
Ingrediente 4			
Ingrediente 5			
Ingrediente 6			
Ingrediente 7			
Ingrediente 8			
Ingrediente 9			
Ingrediente 10			

Proceso culinario	Nº	Peso (en gramos)	Observaciones
1. Olla VACÍA			
2. Olla con COMIDA COCINADA			
**COMIDA COCINADA (2-1)**			
3. Fiambrera VACÍA	*1*	*43 gr*	
4. Fiambrera con COMIDA COCINADA	*1*	*283 gr*	
**COMIDA ALMACENADA (4-3)**		*240 gr*	
5. Plato VACÍO		*43 gr*	
6. Plato con COMIDA COCINADA		*283 gr*	
**COMIDA QUE SIRVE A SU HIJO (6-5)**	*1*	*240 gr*	
7. Plato COMIDA QUE SU HIJO NO HA COMIDO	*1*	*83 gr*	
**COMIDA QUE SU HIJO HA COMIDO (6-7)**		*200 gr*	

MÉTODO DE ELABORACIÓN:
Explicar detalladamente el proceso de elaboración del plato

FICHA INGREDIENTES/PESOS “PLATO HECHO EN CASA (HC)”

ID: SEV 07

DÍA DEL CONSUMO: 14 / 10 / 2014 **HORA DEL CONSUMO:** 18:40

MÉTODO DE ELABORACIÓN:

Explicar detalladamente el proceso de elaboración del plato:

Pelar la pera y los plátanos.
Trocear.
Echar las galletas troceadas.
Batirlo todo.

Fotos:

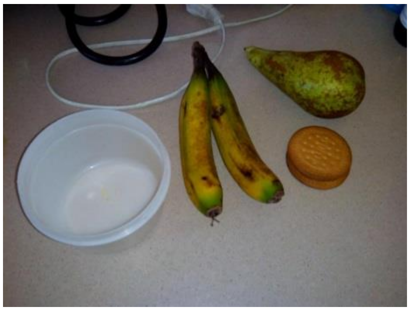

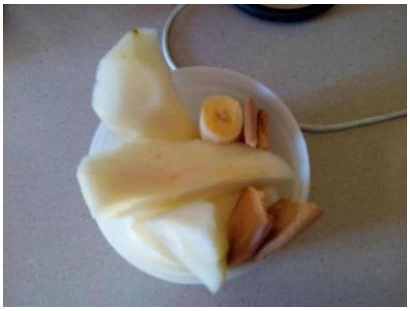

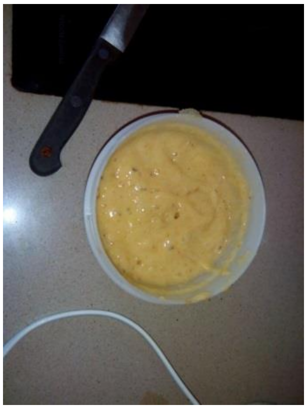

FICHA INGREDIENTES/PESOS “PLATO HECHO EN CASA (HC)” **ID: VAL 01**

DÍA DEL CONSUMO: *13 / 10 / 2014* HORA DEL CONSUMO: *12:40*

LA ELABOARACIÓN DEL PLATO SE HACE EL MISMO DÍA DEL CONSUMO ☐ Sí ☒ No

Si la fecha de elaboración del plato NO es el mismo día del consumo NO se adjuntará foto y el peso de los ingredientes puede ser aproximado. Indique la fecha de elaboración:

TIPO DE PLATO: *PURÉ DE POLLO CON VERDURAS*

	Tipo de ingrediente	Nº	Peso (en gramos)
Ingrediente 1	*Patatas*	*1*	*111 gr*
Ingrediente 2	*Zanahoria*	*1*	*72 gr*
Ingrediente 3	*Judía verde*	*1*	*58 gr*
Ingrediente 4	*Pollo (muslo)*	*¼*	*57 gr*
Ingrediente 5	*Aceite de oliva*	*1*	*1 chorrito al final*
Ingrediente 6			
Ingrediente 7			
Ingrediente 8			
Ingrediente 9			
Ingrediente 10			

Proceso culinario	Nº	Peso (en gramos)	Observaciones
1. Olla VACÍA	*1*	*705 gr*	
2. Olla con COMIDA COCINADA	*1*	*965 gr*	
**COMIDA COCINADA (2-1)**	*1*	*260 gr*	
3. Fiambrera VACÍA			
4. Fiambrera con COMIDA COCINADA			
**COMIDA ALMACENADA (4-3)**			
5. Plato VACÍO	*1*	*112 gr*	
6. Plato con COMIDA COCINADA	*1*	*224 gr*	
**COMIDA QUE SIRVE A SU HIJO (6-5)**	*1*	*112 gr*	
7. Plato COMIDA QUE SU HIJO NO HA COMIDO	*1*	*112 gr*	
**COMIDA QUE SU HIJO HA COMIDO (6-7)**		*112 gr*	

MÉTODO DE ELABORACIÓN:
Explicar detalladamente el proceso de elaboración del plato

FICHA INGREDIENTES/PESOS “PLATO HECHO EN CASA (HC)”

ID: VAL 01

DÍA DEL CONSUMO: 13 / 10 / 2014 **HORA DEL CONSUMO:** 12:40

MÉTODO DE ELABORACIÓN:

Explicar detalladamente el proceso de elaboración del plato:

Pelar y lavar las verduras.
Quitar hueso y piel al trozo de pollo.
Poner agua en la olla y hervir unos 20 minutos.
Triturar.
Añadir aceite de oliva, poco.

Fotos:

FICHA INGREDIENTES/PESOS "PLATO HECHO EN CASA (HC)" **ID: VAL 01**

DÍA DEL CONSUMO: *13 / 10 / 2014* HORA DEL CONSUMO: *20:15*

LA ELABOARACIÓN DEL PLATO SE HACE EL MISMO DÍA DEL CONSUMO ☒ Sí ☐ No

Si la fecha de elaboración del plato NO es el mismo día del consumo NO se adjuntará foto y el peso de los ingredientes puede ser aproximado. Indique la fecha de elaboración:

TIPO DE PLATO: *PURÉ DE MERLUZA CON VERDURAS*

	Tipo de ingrediente	Nº	Peso (en gramos)
Ingrediente 1	*Zanahoria*	*1*	*79,38 gr*
Ingrediente 2	*Cebolla*	*1*	*172,93 gr*
Ingrediente 3	*Calabacín*	*1*	*170,10 gr*
Ingrediente 4	*Judía verde*	*1*	*59,53 gr*
Ingrediente 5	*Patata*	*1*	*99,22 gr*
Ingrediente 6	*Merluza*	*2*	*586,84 gr*
Ingrediente 7	*Aceite de oliva*	*1*	*1 chorrito al final*
Ingrediente 8			
Ingrediente 9			
Ingrediente 10			

Proceso culinario	Nº	Peso (en gramos)	Observaciones
1. Olla VACÍA	*1*	*1131 gr*	
2. Olla con COMIDA COCINADA	*1*	*2639 gr*	
**COMIDA COCINADA (2-1)**	*1*	*1508 gr*	
3. Fiambrera VACÍA			
4. Fiambrera con COMIDA COCINADA			
**COMIDA ALMACENADA (4-3)**			
5. Plato VACÍO		*79,38 gr*	
6. Plato con COMIDA COCINADA		*331,69 gr*	
**COMIDA QUE SIRVE A SU HIJO (6-5)**		*252,31 gr*	
7. Plato COMIDA QUE SU HIJO NO HA COMIDO		*221,13 gr*	
**COMIDA QUE SU HIJO HA COMIDO (6-7)**		*110,56 gr*	

MÉTODO DE ELABORACIÓN:
Explicar detalladamente el proceso de elaboración del plato

FICHA INGREDIENTES/PESOS "PLATO HECHO EN CASA (HC)"

ID: VAL 01

DÍA DEL CONSUMO: 13 / 10 / 2014 **HORA DEL CONSUMO:** 20:15

MÉTODO DE ELABORACIÓN:

Explicar detalladamente el proceso de elaboración del plato:

Pelar y lavar las verduras.
Limpiar el pescado.
Agua y a la olla, hervir 20 minutos.
Aceite de oliva al final.

Fotos:

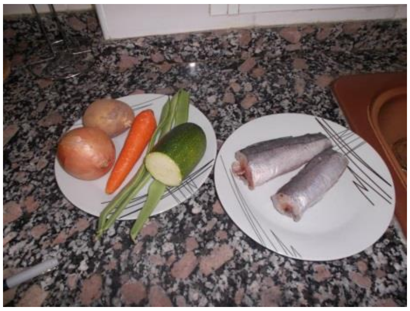

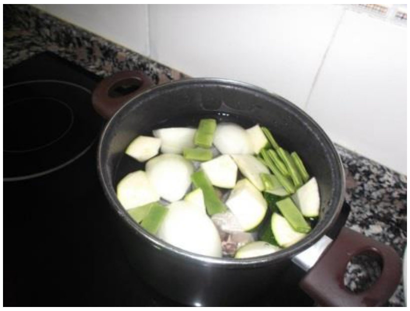

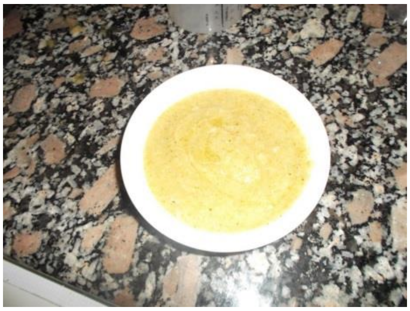

FICHA INGREDIENTES/PESOS “PLATO HECHO EN CASA (HC)” **ID: VAL 01**

DÍA DEL CONSUMO: *15 / 10 / 2014* HORA DEL CONSUMO: *12:35*

LA ELABOARACIÓN DEL PLATO SE HACE EL MISMO DÍA DEL CONSUMO [X] Sí [ ] No

Si la fecha de elaboración del plato NO es el mismo día del consumo NO se adjuntará foto y el peso de los ingredientes puede ser aproximado. Indique la fecha de elaboración:

TIPO DE PLATO: *PURÉ DE VERDURAS CON POLLO*

	Tipo de ingrediente	Nº	Peso (en gramos)
Ingrediente 1	*Zanahoria*	*1*	*39 gr*
Ingrediente 2	*Judía verde*	*1*	*64 gr*
Ingrediente 3	*Patata*	*1*	*212 gr*
Ingrediente 4	*Pollo*	*1*	*56 gr*
Ingrediente 5	*Aceite de oliva*	*1*	*Chorrito al final*
Ingrediente 6			
Ingrediente 7			
Ingrediente 8			
Ingrediente 9			
Ingrediente 10			

Proceso culinario	Nº	Peso (en gramos)	Observaciones
1. Olla VACÍA		*704 gr*	
2. Olla con COMIDA COCINADA		*1285 gr*	
**COMIDA COCINADA (2-1)**		*581 gr*	
3. Fiambrera VACÍA			
4. Fiambrera con COMIDA COCINADA			
**COMIDA ALMACENADA (4-3)**			
5. Plato VACÍO		*221 gr*	
6. Plato con COMIDA COCINADA		*390 gr*	
**COMIDA QUE SIRVE A SU HIJO (6-5)**		*169 gr*	
7. Plato COMIDA QUE SU HIJO NO HA COMIDO		*321 gr*	
**COMIDA QUE SU HIJO HA COMIDO (6-7)**		*69 gr*	

MÉTODO DE ELABORACIÓN:
Explicar detalladamente el proceso de elaboración del plato

FICHA INGREDIENTES/PESOS "PLATO HECHO EN CASA (HC)"

ID: VAL 01

DÍA DEL CONSUMO: 15 / 10 / 2014 **HORA DEL CONSUMO:** 12:35

MÉTODO DE ELABORACIÓN:

Explicar detalladamente el proceso de elaboración del plato:

Pelar y cortar todas las verduras.
Olla a hervir con la verdura y el pollo, 20 minutos.
Aceite de oliva al final.
Triturar.

Fotos:

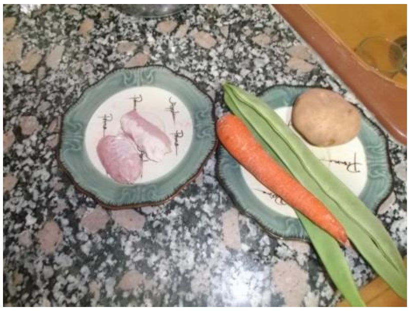

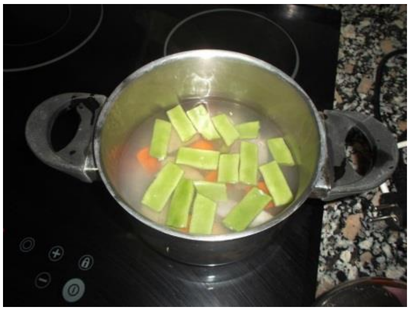

FICHA INGREDIENTES/PESOS “PLATO HECHO EN CASA (HC)” **ID: VAL 01**

DÍA DEL CONSUMO: *15 / 10 / 2014* HORA DEL CONSUMO: *20:00*

LA ELABOARACIÓN DEL PLATO SE HACE EL MISMO DÍA DEL CONSUMO ☐ Sí ☒ No

Si la fecha de elaboración del plato NO es el mismo día del consumo NO se adjuntará foto y el peso de los ingredientes puede ser aproximado. Indique la fecha de elaboración:

TIPO DE PLATO: SÉMOLA DE CALDO DE POLLO

	Tipo de ingrediente	Nº	Peso (en gramos)
Ingrediente 1	*Caldo de pollo*		
Ingrediente 2	*Sémola de arroz*		*18 gr*
Ingrediente 3			
Ingrediente 4			
Ingrediente 5			
Ingrediente 6			
Ingrediente 7			
Ingrediente 8			
Ingrediente 9			
Ingrediente 10			

Proceso culinario	Nº	Peso (en gramos)	Observaciones
1. Olla VACÍA			
2. Olla con COMIDA COCINADA			
**COMIDA COCINADA (2-1)**			
3. Fiambrera VACÍA			
4. Fiambrera con COMIDA COCINADA			
**COMIDA ALMACENADA (4-3)**			
5. Plato VACÍO		*110 gr*	
6. Plato con COMIDA COCINADA		*232 gr*	
**COMIDA QUE SIRVE A SU HIJO (6-5)**		*122 gr*	
7. Plato COMIDA QUE SU HIJO NO HA COMIDO		*110 gr*	
**COMIDA QUE SU HIJO HA COMIDO (6-7)**		*122 gr*	

MÉTODO DE ELABORACIÓN:
Explicar detalladamente el proceso de elaboración del plato

FICHA INGREDIENTES/PESOS "PLATO HECHO EN CASA (HC)" **ID: VAL 01**

DÍA DEL CONSUMO: 15 / 10 / 2014 **HORA DEL CONSUMO:** 20:00

MÉTODO DE ELABORACIÓN:

Explicar detalladamente el proceso de elaboración del plato:

Del caldo de la mañana del puré de verduras, aparto un vaso de caldo, lo pongo a hervir.
Añadir una cucharada de sémola de arroz.

Fotos:

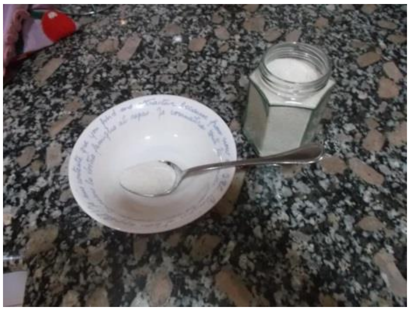

FICHA INGREDIENTES/PESOS "PLATO HECHO EN CASA (HC)" **ID: VAL 01**

DÍA DEL CONSUMO: *19 / 10 / 2014* HORA DEL CONSUMO: *13:50*

LA ELABOARACIÓN DEL PLATO SE HACE EL MISMO DÍA DEL CONSUMO ☒ Sí ☐ No

Si la fecha de elaboración del plato NO es el mismo día del consumo NO se adjuntará foto y el peso de los ingredientes puede ser aproximado. Indique la fecha de elaboración:

TIPO DE PLATO: *PURÉ DE PAVO, VERDURAS Y LENTEJAS*

	Tipo de ingrediente	Nº	Peso (en gramos)
Ingrediente 1	*Pavo*	*1*	*41 gr*
Ingrediente 2	*Patata*	*1*	*67 gr*
Ingrediente 3	*Guisantes*	*1*	*30 gr*
Ingrediente 4	*Cebolla*	*1*	*82 gr*
Ingrediente 5	*Lentejas*	*1*	*13 gr*
Ingrediente 6	*Tomate*	*1*	*142 gr*
Ingrediente 7	*Aceite de oliva*	*1*	*Chorrito al final*
Ingrediente 8			
Ingrediente 9			
Ingrediente 10			

Proceso culinario	Nº	Peso (en gramos)	Observaciones
1. Olla VACÍA	*1*	*1131 gr*	
2. Olla con COMIDA COCINADA	*1*	*2577 gr*	
**COMIDA COCINADA (2-1)**	*1*	*1446 gr*	
3. Fiambrera VACÍA			
4. Fiambrera con COMIDA COCINADA			
**COMIDA ALMACENADA (4-3)**			
5. Plato VACÍO	*1*	*111 gr*	
6. Plato con COMIDA COCINADA	*1*	*299 gr*	
**COMIDA QUE SIRVE A SU HIJO (6-5)**	*1*	*188 gr*	
7. Plato COMIDA QUE SU HIJO NO HA COMIDO	*1*	*207 gr*	
**COMIDA QUE SU HIJO HA COMIDO (6-7)**	*1*	*92 gr*	

MÉTODO DE ELABORACIÓN:
Explicar detalladamente el proceso de elaboración del plato

FICHA INGREDIENTES/PESOS “PLATO HECHO EN CASA (HC)”

ID: VAL 01

DÍA DEL CONSUMO: 19 / 10 / 2014 **HORA DEL CONSUMO:** 13:50

MÉTODO DE ELABORACIÓN:
Explicar detalladamente el proceso de elaboración del plato:

Pelar y pelar todas las verduras.
Cocer todo añadiendo un poco de lentejas sin piel.
Cocer 30 minutos.
Triturar.

Fotos:

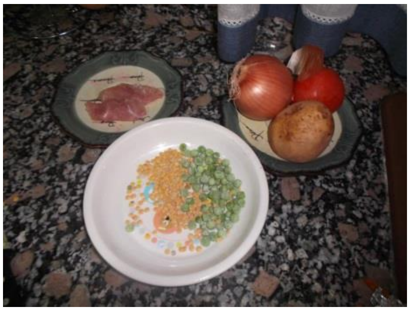
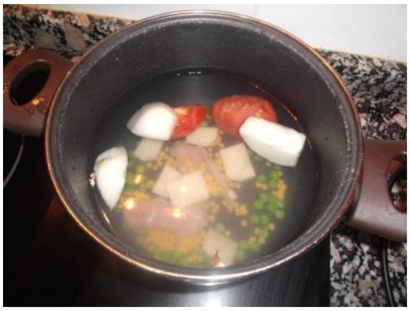
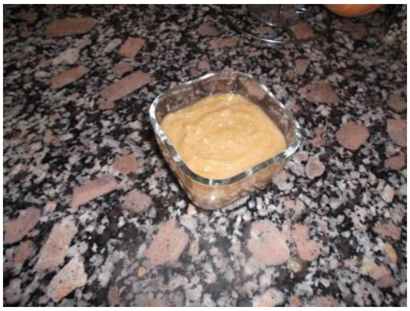

FICHA INGREDIENTES/PESOS "PLATO HECHO EN CASA (HC)" **ID: VAL 01**

DÍA DEL CONSUMO: *19 / 10 / 2014* **HORA DEL CONSUMO:** *20:00*

LA ELABOARACIÓN DEL PLATO SE HACE EL MISMO DÍA DEL CONSUMO ☐ Sí ☒ No

Si la fecha de elaboración del plato NO es el mismo día del consumo NO se adjuntará foto y el peso de los ingredientes puede ser aproximado. Indique la fecha de elaboración:

TIPO DE PLATO: *SÉMOLA DE CALDO DE PAVO*

	Tipo de ingrediente	Nº	Peso (en gramos)
Ingrediente 1	*Caldo de pavo*	*1*	
Ingrediente 2	*Sémola de arroz*	*1*	*16 gr*
Ingrediente 3			
Ingrediente 4			
Ingrediente 5			
Ingrediente 6			
Ingrediente 7			
Ingrediente 8			
Ingrediente 9			
Ingrediente 10			

Proceso culinario	Nº	Peso (en gramos)	Observaciones
1. Olla VACÍA			
2. Olla con COMIDA COCINADA			
**COMIDA COCINADA (2-1)**			
3. Fiambrera VACÍA			
4. Fiambrera con COMIDA COCINADA			
**COMIDA ALMACENADA (4-3)**			
5. Plato VACÍO	*1*	*110 gr*	
6. Plato con COMIDA COCINADA	*1*	*269 gr*	
**COMIDA QUE SIRVE A SU HIJO (6-5)**	*1*	*159 gr*	
7. Plato COMIDA QUE SU HIJO NO HA COMIDO		*110 gr*	
**COMIDA QUE SU HIJO HA COMIDO (6-7)**		*159 gr*	

MÉTODO DE ELABORACIÓN:
Explicar detalladamente el proceso de elaboración del plato

FICHA INGREDIENTES/PESOS "PLATO HECHO EN CASA (HC)"

ID: VAL 01

DÍA DEL CONSUMO: 19 / 10 / 2014 **HORA DEL CONSUMO:** 20:00

MÉTODO DE ELABORACIÓN:

Explicar detalladamente el proceso de elaboración del plato:

Del caldo de la mañana de pavo, hervir y añadir la sémola.

Fotos:

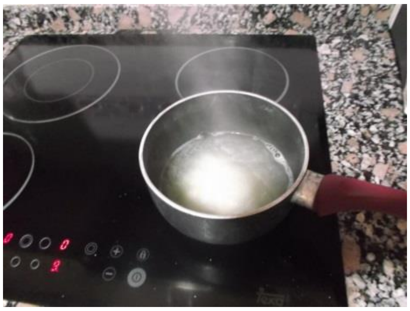

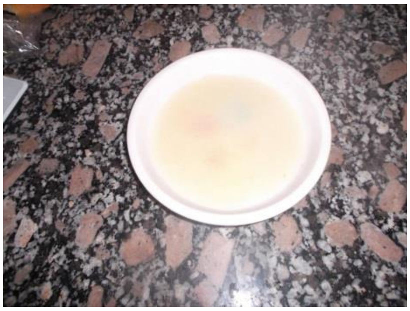

FICHA INGREDIENTES/PESOS “PLATO HECHO EN CASA (HC)” **ID: VAL 02**

DÍA DEL CONSUMO: *13 / 10 / 2014* HORA DEL CONSUMO: *15:15*

LA ELABOARACIÓN DEL PLATO SE HACE EL MISMO DÍA DEL CONSUMO [X] Sí [ ] No

Si la fecha de elaboración del plato NO es el mismo día del consumo NO se adjuntará foto y el peso de los ingredientes puede ser aproximado. Indique la fecha de elaboración:

TIPO DE PLATO: *LENTEJAS CON VERDURAS*

	Tipo de ingrediente	Nº	Peso (en gramos)
Ingrediente 1	*Aceite de oliva*	*1*	*1 chorro*
Ingrediente 2	*Sal*	*1*	*1 pizca*
Ingrediente 3	*Tarro lentejas Hacendado*	*1*	*400 gr*
Ingrediente 4	*Patatas*	*2*	*352 gr*
Ingrediente 5	*Zanahoria*	*2*	*153 gr*
Ingrediente 6	*Cebolla*	*1*	*176 gr*
Ingrediente 7	*Acelgas*	*1*	*69 gr*
Ingrediente 8	*Agua del grifo*	*1*	*1,5 l (1616 gr)*
Ingrediente 9	*Pimentón*	*1*	*1 pizca*
Ingrediente 10			

Proceso culinario	Nº	Peso (en gramos)	Observaciones
1. Olla VACÍA	*1*	*1300 gr*	
2. Olla con COMIDA COCINADA		*3496 gr*	
**COMIDA COCINADA (2-1)**		*2196 gr*	
3. Fiambrera VACÍA			
4. Fiambrera con COMIDA COCINADA			
**COMIDA ALMACENADA (4-3)**			
5. Plato VACÍO	*1*	*363 gr*	
6. Plato con COMIDA COCINADA		*598 gr*	
**COMIDA QUE SIRVE A SU HIJO (6-5)**		*235 gr*	
7. Plato COMIDA QUE SU HIJO NO HA COMIDO		*408 gr*	
**COMIDA QUE SU HIJO HA COMIDO (6-7)**		*190 gr*	

MÉTODO DE ELABORACIÓN:

Explicar detalladamente el proceso de elaboración del plato

FICHA INGREDIENTES/PESOS "PLATO HECHO EN CASA (HC)" **ID: VAL 02**

DÍA DEL CONSUMO: 13 / 10 / 2014 **HORA DEL CONSUMO:** 15:15

MÉTODO DE ELABORACIÓN:
Explicar detalladamente el proceso de elaboración del plato:

Pelar y cortar todas las verduras en pequeños trozos.
Se sofríen las verduras todas juntas con una pizca de sal y un chorro de aceite de oliva.
Ponemos a hervir agua en una cazuela y cuando llegue a ebullición, añadimos los ingredientes (todas las verduras).
Escurrimos las lentejas y las añadimos también a la cazuela.
Hervimos todo durante 15 minutos.
Después lo servimos en el plato listo para comer.

(*) Esto es para el consumo de toda la familia (padre, madre y niño).

Fotos:

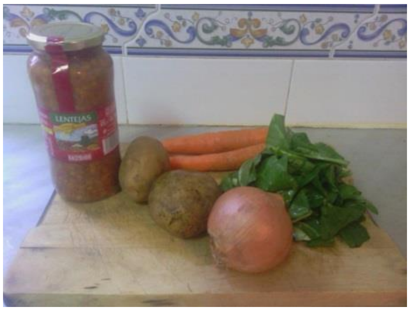

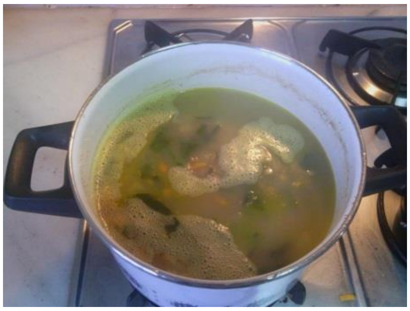

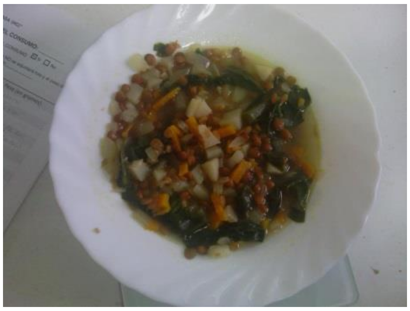

FICHA INGREDIENTES/PESOS "PLATO HECHO EN CASA (HC)" **ID: VAL 02**

DÍA DEL CONSUMO: *13 / 10 / 2014* HORA DEL CONSUMO: *18:45*

LA ELABOARACIÓN DEL PLATO SE HACE EL MISMO DÍA DEL CONSUMO [X] Sí [ ] No

Si la fecha de elaboración del plato NO es el mismo día del consumo NO se adjuntará foto y el peso de los ingredientes puede ser aproximado. Indique la fecha de elaboración:

TIPO DE PLATO: *PAPILLA DE FRUTAS CON GALLETA MARIA*

	Tipo de ingrediente	Nº	Peso (en gramos)
Ingrediente 1	*Plátano*	*1*	*75 gr*
Ingrediente 2	*Zumo de naranja natural*	*1*	*105 ml (103 gr)*
Ingrediente 3	*Manzana*	*1*	*109 gr*
Ingrediente 4	*Pera*	*1*	*66 gr*
Ingrediente 5	*Galletas maría doradas (Carrefour)*	*5*	*36 gr*
Ingrediente 6			
Ingrediente 7			
Ingrediente 8			
Ingrediente 9			

Proceso culinario	Nº	Peso (en gramos)	Observaciones
1. Olla VACÍA	*1*	*130*	*Recipiente triturar*
2. Olla con COMIDA COCINADA	*1*	*470*	
**COMIDA COCINADA (2-1)**		*340*	
3. Fiambrera VACÍA			
4. Fiambrera con COMIDA COCINADA			
**COMIDA ALMACENADA (4-3)**			
5. Plato VACÍO	*1*	*36*	
6. Plato con COMIDA COCINADA		*184*	
**COMIDA QUE SIRVE A SU HIJO (6-5)**		*148*	
7. Plato COMIDA QUE SU HIJO NO HA COMIDO		*36*	
**COMIDA QUE SU HIJO HA COMIDO (6-7)**		*148*	

MÉTODO DE ELABORACIÓN:
Explicar detalladamente el proceso de elaboración del plato

FICHA INGREDIENTES/PESOS “PLATO HECHO EN CASA (HC)” **ID: VAL 02**

DÍA DEL CONSUMO: 13 / 10 / 2014 **HORA DEL CONSUMO:** 18:45

MÉTODO DE ELABORACIÓN:
Explicar detalladamente el proceso de elaboración del plato:

Exprimir una naranja.
Pelar toda la fruta (naranja, plátano, pera y manzana).
Introducir toda la fruta pelada, el zumo de la naranja natural y las galletas en un recipiente.
Triturarlo todo con la batidora.
Servir en un bol.

Fotos:

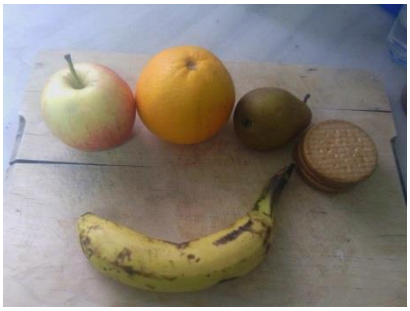
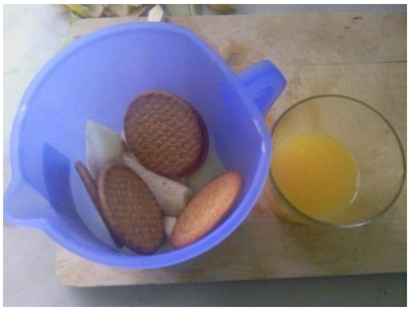
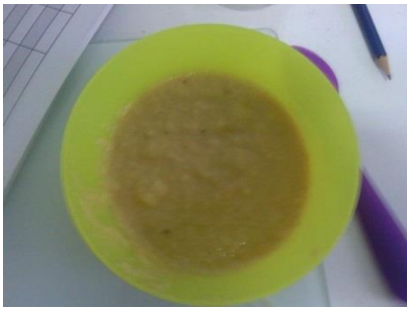

FICHA INGREDIENTES/PESOS "PLATO HECHO EN CASA (HC)" **ID: VAL 02**

DÍA DEL CONSUMO: *17 / 10 / 2014* HORA DEL CONSUMO: *15:20*

LA ELABOARACIÓN DEL PLATO SE HACE EL MISMO DÍA DEL CONSUMO [X] Sí [ ] No

Si la fecha de elaboración del plato NO es el mismo día del consumo NO se adjuntará foto y el peso de los ingredientes puede ser aproximado. Indique la fecha de elaboración:

TIPO DE PLATO: *ARROZ CON POLLO Y VERDURAS*

	Tipo de ingrediente	Nº	Peso (en gramos)
Ingrediente 1	*Calabacín*	*1*	*227 gr*
Ingrediente 2	*Zanahoria*	*2*	*197 gr*
Ingrediente 3	*Patata*	*3*	*424 gr*
Ingrediente 4	*Acelgas*		*216 gr*
Ingrediente 5	*Cebolla*	*1*	*227 gr*
Ingrediente 6	*Agua*		*1616 gr (1,5 litros)*
Ingrediente 7	*Pollo*	*4*	*537 gr*
Ingrediente 8	*Sal*		*3 pizcas*
Ingrediente 9	*Arroz (La Fallera)*		*516 gr*
Ingrediente 10	*Pimentón dulce Hacendado*		*1 pizca*

Proceso culinario	Nº	Peso (en gramos)	Observaciones
1. Olla VACÍA	*1*	*1300 gr*	
2. Olla con COMIDA COCINADA		*2527 gr*	
**COMIDA COCINADA (2-1)**		*1227 gr*	
3. Fiambrera VACÍA			
4. Fiambrera con COMIDA COCINADA			
**COMIDA ALMACENADA (4-3)**			
5. Plato VACÍO	*1*	*359 gr*	
6. Plato con COMIDA COCINADA		*719 gr*	
**COMIDA QUE SIRVE A SU HIJO (6-5)**		*360 gr*	
7. Plato COMIDA QUE SU HIJO NO HA COMIDO		*605 gr*	
**COMIDA QUE SU HIJO HA COMIDO (6-7)**		*114 gr*	

MÉTODO DE ELABORACIÓN:
Explicar detalladamente el proceso de elaboración del plato

FICHA INGREDIENTES/PESOS "PLATO HECHO EN CASA (HC)"

ID: VAL 02

DÍA DEL CONSUMO: 17 / 10 / 2014 **HORA DEL CONSUMO:** 15:20

MÉTODO DE ELABORACIÓN:

Explicar detalladamente el proceso de elaboración del plato:

Hacer un sofrito con aceite de oliva y todas las verduras (anteriormente peladas, cortas y lavadas) en una cazuela.
Añadir un litro y medio de agua y verter las acelgas.
Añadir una pizca de pimentón dulce.
Cuando llegue a ebullición, añadir el arroz y dejar cocer 20 minutos.
Servir y consumir.

(*) Esto es para el consumo de toda la familia (padre, madre y niño).

Fotos:

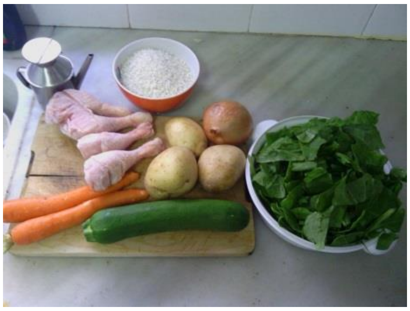

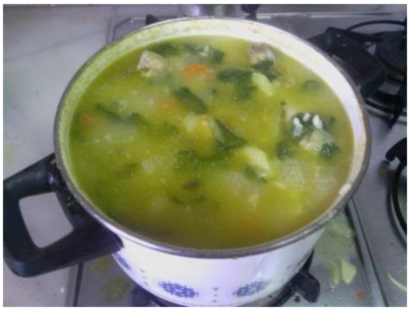

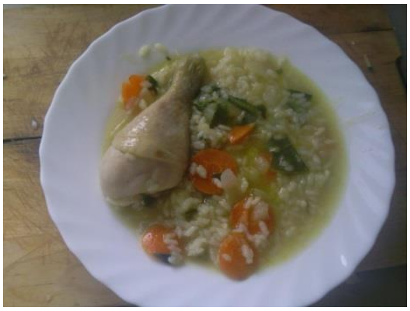

FICHA INGREDIENTES/PESOS "PLATO HECHO EN CASA (HC)" **ID: VAL 02**

DÍA DEL CONSUMO: *17 / 10 / 2014* HORA DEL CONSUMO: *18:55*

LA ELABOARACIÓN DEL PLATO SE HACE EL MISMO DÍA DEL CONSUMO [X] Sí [ ] No

Si la fecha de elaboración del plato NO es el mismo día del consumo NO se adjuntará foto y el peso de los ingredientes puede ser aproximado. Indique la fecha de elaboración:

TIPO DE PLATO: *PAPILLA DE FRUTAS CON GALLETA MARIA*

	Tipo de ingrediente	Nº	Peso (en gramos)
Ingrediente 1	*Manzana*	*2*	*233 gr*
Ingrediente 2	*Plátano*	*1*	*77 gr*
Ingrediente 3	*Pera*	*1*	*124 gr*
Ingrediente 4	*Galletas María (Hacendado)*	*5*	*36 gr*
Ingrediente 5	*Zumo de naranja natural*	*1 naranja*	*14 gr*
Ingrediente 6			
Ingrediente 7			
Ingrediente 8			
Ingrediente 9			
Ingrediente 10			

Proceso culinario	Nº	Peso (en gramos)	Observaciones
1. Olla VACÍA	*1*	*105 gr*	
2. Olla con COMIDA COCINADA	*1*	*589 gr*	
**COMIDA COCINADA (2-1)**		*484 gr*	
3. Fiambrera VACÍA			
4. Fiambrera con COMIDA COCINADA			
**COMIDA ALMACENADA (4-3)**			
5. Plato VACÍO	*1*	*35 gr*	
6. Plato con COMIDA COCINADA	*1*	*519 gr*	
**COMIDA QUE SIRVE A SU HIJO (6-5)**		*484 gr*	
7. Plato COMIDA QUE SU HIJO NO HA COMIDO		*35 gr*	
**COMIDA QUE SU HIJO HA COMIDO (6-7)**		*484 gr*	

MÉTODO DE ELABORACIÓN:
Explicar detalladamente el proceso de elaboración del plato

FICHA INGREDIENTES/PESOS "PLATO HECHO EN CASA (HC)" **ID: VAL 02**

DÍA DEL CONSUMO: 17 / 10 / 2014 **HORA DEL CONSUMO:** 18:55

MÉTODO DE ELABORACIÓN:
Explicar detalladamente el proceso de elaboración del plato:

Lavar, cortar y pelar las frutas.
Añadirlas a un recipiente para triturar.
Exprimir la naranja y verter el zumo en el mismo recipiente.
Añadir también las galletas maría y triturarlo todo.
Servir y consumir.

Fotos:

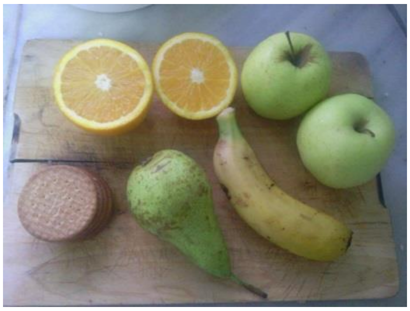

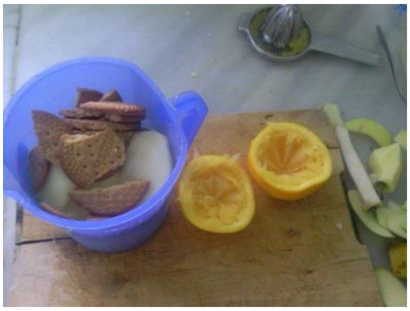

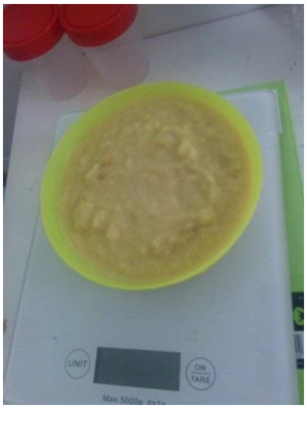

FICHA INGREDIENTES/PESOS “PLATO HECHO EN CASA (HC)” **ID: VAL 02**

DÍA DEL CONSUMO: *18 / 10 / 2014* HORA DEL CONSUMO: *17:45*

LA ELABOARACIÓN DEL PLATO SE HACE EL MISMO DÍA DEL CONSUMO [X] Sí [ ] No

Si la fecha de elaboración del plato NO es el mismo día del consumo NO se adjuntará foto y el peso de los ingredientes puede ser aproximado. Indique la fecha de elaboración:

TIPO DE PLATO: *PAPILLA DE FRUTAS CON GALLETA MARIA*

	Tipo de ingrediente	Nº	Peso (en gramos)
Ingrediente 1	*Plátano*	*1*	*93 gr*
Ingrediente 2	*Galletas Maria*	*5*	*35 gr*
Ingrediente 3	*Pera*	*1*	*111 gr*
Ingrediente 4	*Manzana*	*2*	*250 gr*
Ingrediente 5	*Zumo de naranja natural*	*1*	*14 gr*
Ingrediente 6			
Ingrediente 7			
Ingrediente 8			
Ingrediente 9			
Ingrediente 10			

Proceso culinario	Nº	Peso (en gramos)	Observaciones
1. Olla VACÍA	*1*	*105 gr*	*Recipiente triturar*
2. Olla con COMIDA COCINADA	*1*	*635 gr*	*Con comida triturada*
**COMIDA COCINADA (2-1)**		*530 gr*	
3. Fiambrera VACÍA			
4. Fiambrera con COMIDA COCINADA			
**COMIDA ALMACENADA (4-3)**			
5. Plato VACÍO		*35 gr*	
6. Plato con COMIDA COCINADA		*338 gr*	
**COMIDA QUE SIRVE A SU HIJO (6-5)**		*303 gr*	
7. Plato COMIDA QUE SU HIJO NO HA COMIDO		*35 gr*	
**COMIDA QUE SU HIJO HA COMIDO (6-7)**		*303 gr*	

MÉTODO DE ELABORACIÓN:
Explicar detalladamente el proceso de elaboración del plato

FICHA INGREDIENTES/PESOS "PLATO HECHO EN CASA (HC)"

ID: VAL 02

DÍA DEL CONSUMO: 18 / 10 / 2014 **HORA DEL CONSUMO:** 17:45

MÉTODO DE ELABORACIÓN:

Explicar detalladamente el proceso de elaboración del plato:

Lavar, pelar y cortas todas las frutas.
Exprimir la naranja.
Verter el zumo de naranja, las frutas y las galletas en un recipiente para triturar.
Triturar todo.
Servir y consumir.

Fotos:

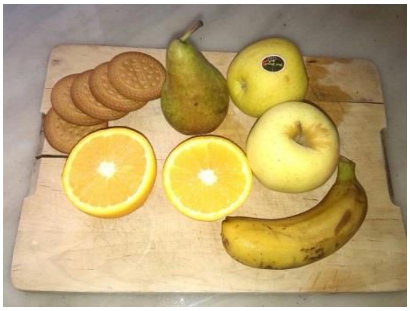
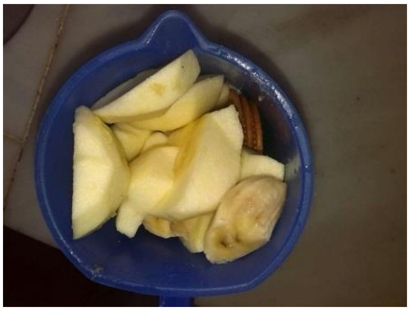
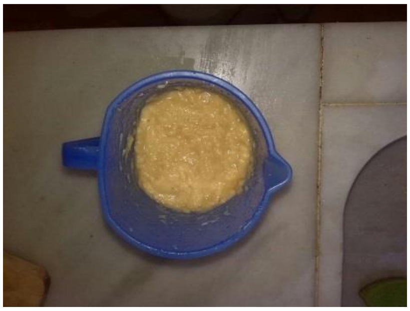

FICHA INGREDIENTES/PESOS "PLATO HECHO EN CASA (HC)" **ID: VAL 03**

DÍA DEL CONSUMO: *14 / 10 / 2014* HORA DEL CONSUMO: *13:00*

LA ELABOARACIÓN DEL PLATO SE HACE EL MISMO DÍA DEL CONSUMO [X] Sí [ ] No

Si la fecha de elaboración del plato NO es el mismo día del consumo NO se adjuntará foto y el peso de los ingredientes puede ser aproximado. Indique la fecha de elaboración:

TIPO DE PLATO: *POLLO CON ARROZ Y VERDURAS*

	Tipo de ingrediente	Nº	Peso (en gramos)
Ingrediente 1	*Pollo*		*20,5 gr*
Ingrediente 2	*Ternera*		*50 gr*
Ingrediente 3	*Arroz*		*40 gr*
Ingrediente 4	*Patata*		*166 gr*
Ingrediente 5	*Zanahoria*		*92 gr*
Ingrediente 6	*Cebolla*		*88 gr*
Ingrediente 7	*Judías verdes*		*55 gr*
Ingrediente 8	*Calabacín*		*100 gr*
Ingrediente 9	*Aceite*		*20 gr*
Ingrediente 10	*Sal*		*3 gr*

Proceso culinario	Nº	Peso (en gramos)	Observaciones
1. Olla VACÍA		*2486 gr*	
2. Olla con COMIDA COCINADA		*3420 gr*	
**COMIDA COCINADA (2-1)**		*934 gr*	
3. Fiambrera VACÍA		*32 gr*	
4. Fiambrera con COMIDA COCINADA		*330 gr*	
**COMIDA ALMACENADA (4-3)**		*298 gr*	
5. Plato VACÍO		*287 gr*	
6. Plato con COMIDA COCINADA		*573 gr*	
**COMIDA QUE SIRVE A SU HIJO (6-5)**		*286 gr*	
7. Plato COMIDA QUE SU HIJO NO HA COMIDO		*368 gr*	
**COMIDA QUE SU HIJO HA COMIDO (6-7)**		*205 gr*	

MÉTODO DE ELABORACIÓN:
Explicar detalladamente el proceso de elaboración del plato

FICHA INGREDIENTES/PESOS "PLATO HECHO EN CASA (HC)" **ID: VAL 03**

DÍA DEL CONSUMO: 14 / 10 / 2014 **HORA DEL CONSUMO:** 13:00

MÉTODO DE ELABORACIÓN:
Explicar detalladamente el proceso de elaboración del plato:

Se preparan los ingredientes, se limpia el pollo, se trocea la ternera, se pelan y se lavan las verduras.
Se coloca la olla al fuego con agua.
Cuando el agua está hirviendo se introducen los ingredientes por este orden: ternera, pollo, patata, cebolla, zanahoria, judías verdes, calabacín y sal.
Cuando los ingredientes anteriores han estado 40 minutos aproximadamente.
Se introduce el arroz durante 20 minutos.
Después de 1 hora se traspasan los ingredientes escurrido a la Thermomix (velocidad 10 durante 1 minuto).
Se añade el aceite y algo de caldo de la cocción de los alimentos (30 gr), para obtener la consistencia deseada.
Se sirve una parte de la comida y la otra se guarda en el frigorífico para el día siguiente.

Fotos:

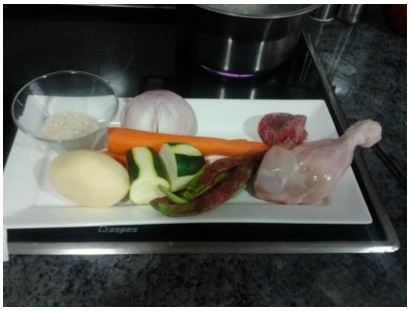

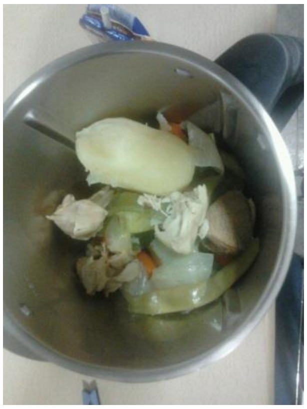

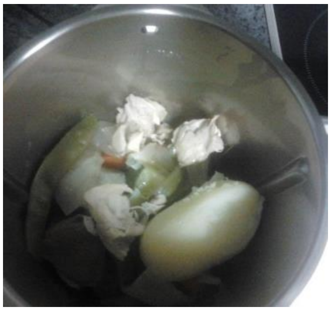

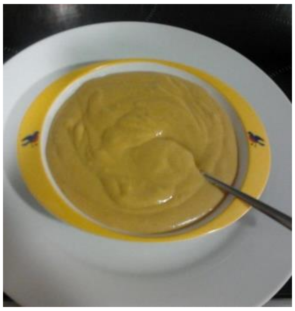

FICHA INGREDIENTES/PESOS “PLATO HECHO EN CASA (HC)” **ID: VAL 03**

DÍA DEL CONSUMO: *14 / 10 / 2014* HORA DEL CONSUMO: *20:00*

LA ELABOARACIÓN DEL PLATO SE HACE EL MISMO DÍA DEL CONSUMO ☒ Sí ☐ No

Si la fecha de elaboración del plato NO es el mismo día del consumo NO se adjuntará foto y el peso de los ingredientes puede ser aproximado. Indique la fecha de elaboración:

TIPO DE PLATO: *PESC ADILLA CON VERDURAS*

	Tipo de ingrediente	Nº	Peso (en gramos)
Ingrediente 1	*Pescadilla*		*157 gr*
Ingrediente 2	*Patata*		*138 gr*
Ingrediente 3	*Cebolla*		*78 gr*
Ingrediente 4	*Zanahoria*		*54 gr*
Ingrediente 5	*Judías verdes*		*81 gr*
Ingrediente 6	*Calabacín*		*78 gr*
Ingrediente 7	*Quesito*		*16 gr*
Ingrediente 8	*Sal*		*3 gr*
Ingrediente 9	*Aceite*		*15 gr*
Ingrediente 10			

Proceso culinario	Nº	Peso (en gramos)	Observaciones
1. Olla VACÍA		*1552 gr*	
2. Olla con COMIDA COCINADA		*2113 gr*	
**COMIDA COCINADA (2-1)**		*560 gr*	
3. Fiambrera VACÍA			
4. Fiambrera con COMIDA COCINADA			
**COMIDA ALMACENADA (4-3)**			
5. Plato VACÍO		*90 gr*	
6. Plato con COMIDA COCINADA		*398 gr*	
**COMIDA QUE SIRVE A SU HIJO (6-5)**		*308 gr*	
7. Plato COMIDA QUE SU HIJO NO HA COMIDO		*131 gr*	
**COMIDA QUE SU HIJO HA COMIDO (6-7)**		*267 gr*	

MÉTODO DE ELABORACIÓN:
Explicar detalladamente el proceso de elaboración del plato

FICHA INGREDIENTES/PESOS "PLATO HECHO EN CASA (HC)" **ID: VAL 03**

DÍA DEL CONSUMO: 14 / 10 / 2014 **HORA DEL CONSUMO:** 20:00

MÉTODO DE ELABORACIÓN:
Explicar detalladamente el proceso de elaboración del plato:

Se lavan y se pelan las verduras.
Cuando el agua está hirviendo se echan las verduras durante 30 minutos.
Después se echa la pescadilla y se tiene todo durante 10 minutos más.
Se quitan las espinas de la pescadilla y se tritura todo con el quesito, el aceite, la sal y un poco de agua de cocción.

Fotos:

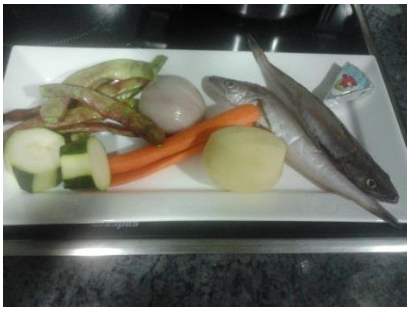

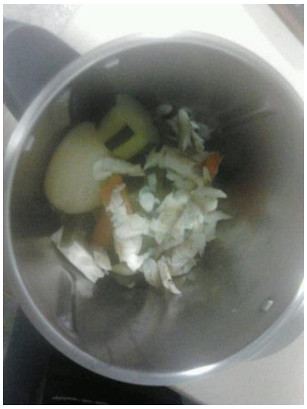

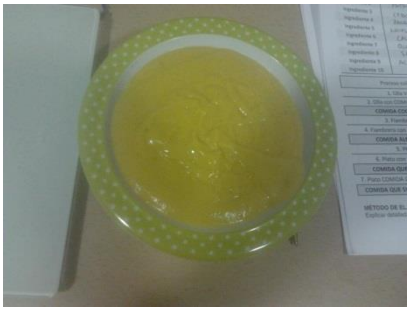

FICHA INGREDIENTES/PESOS "PLATO HECHO EN CASA (HC)" **ID: VAL 03**

DÍA DEL CONSUMO: *16 / 10 / 2014* HORA DEL CONSUMO: *20:00*

LA ELABOARACIÓN DEL PLATO SE HACE EL MISMO DÍA DEL CONSUMO [X] Sí [ ] No

Si la fecha de elaboración del plato NO es el mismo día del consumo NO se adjuntará foto y el peso de los ingredientes puede ser aproximado. Indique la fecha de elaboración:

TIPO DE PLATO: *HUEVO CON VERDURAS*

	Tipo de ingrediente	Nº	Peso (en gramos)
Ingrediente 1	*Huevo*	*2*	*107 gr*
Ingrediente 2	*Patata*	*1*	*176 gr*
Ingrediente 3	*Zanahoria*	*1*	*83 gr*
Ingrediente 4	*Cebolla*	*¼*	*101 gr*
Ingrediente 5	*Judías verdes*	*5*	*82 gr*
Ingrediente 6	*Quesito*	*1*	*15 gr*
Ingrediente 7	*Aceite*		*20 gr*
Ingrediente 8	*Sal*		*3 gr*
Ingrediente 9			
Ingrediente 10			

Proceso culinario	Nº	Peso (en gramos)	Observaciones
1. Olla VACÍA		*1550 gr*	
2. Olla con COMIDA COCINADA		*2150 gr*	
**COMIDA COCINADA (2-1)**		*600 gr*	
3. Fiambrera VACÍA			
4. Fiambrera con COMIDA COCINADA			
**COMIDA ALMACENADA (4-3)**			
5. Plato VACÍO		*92 gr*	
6. Plato con COMIDA COCINADA		*420 gr*	
**COMIDA QUE SIRVE A SU HIJO (6-5)**		*328 gr*	
7. Plato COMIDA QUE SU HIJO NO HA COMIDO		*164 gr*	
**COMIDA QUE SU HIJO HA COMIDO (6-7)**		*256 gr*	

MÉTODO DE ELABORACIÓN:
Explicar detalladamente el proceso de elaboración del plato

FICHA INGREDIENTES/PESOS "PLATO HECHO EN CASA (HC)"

ID: VAL 03

DÍA DEL CONSUMO: 16 / 10 / 2014 **HORA DEL CONSUMO:** 20:00

MÉTODO DE ELABORACIÓN:

Explicar detalladamente el proceso de elaboración del plato:

Se pelan y se lavan las verduras.
Cuando el agua está hirviendo se echan las verduras durante 30-40 minutos.
Aparte se hierve el huevo.
Se ponen las verduras, el huevo, el quesito, el aceite, la sal y un poco de agua de cocción.
Se tritura.

Fotos:

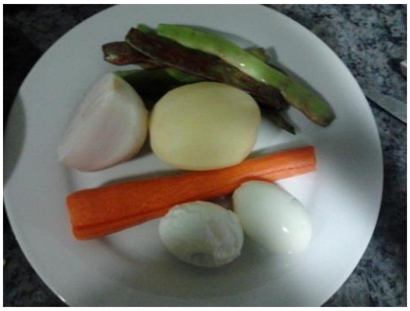

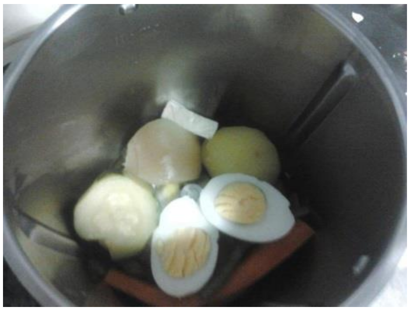

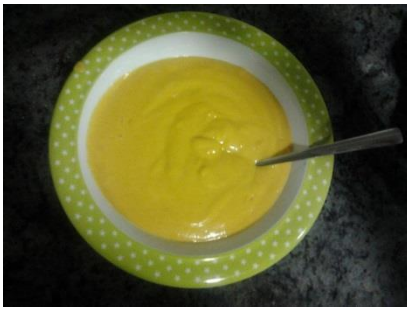

FICHA INGREDIENTES/PESOS "PLATO HECHO EN CASA (HC)" **ID: VAL 03**

DÍA DEL CONSUMO: *18 / 10 / 2014* HORA DEL CONSUMO: *13:30*

LA ELABOARACIÓN DEL PLATO SE HACE EL MISMO DÍA DEL CONSUMO [X] Sí [ ] No

Si la fecha de elaboración del plato NO es el mismo día del consumo NO se adjuntará foto y el peso de los ingredientes puede ser aproximado. Indique la fecha de elaboración:

TIPO DE PLATO: *POLLO CON FIDEOS Y VERDURAS*

	Tipo de ingrediente	Nº	Peso (en gramos)
Ingrediente 1	*Pollo*	*Muslo*	*244 gr*
Ingrediente 2	*Ternera*	*1*	*48 gr*
Ingrediente 3	*Judías*	*4*	*7 gr*
Ingrediente 4	*Judías verdes*	*5*	*72 gr*
Ingrediente 5	*Zanahoria*	*1*	*97 gr*
Ingrediente 6	*Patata*	*1*	*156 gr*
Ingrediente 7	*Calabacín*	*¼*	*127 gr*
Ingrediente 8	*Puerro*	*¼*	*43 gr*
Ingrediente 9	*Cebolla*	*¼*	*70 gr*
Ingrediente 10	*Fideos*	*Un puñado*	*24 gr*
Ingrediente 11	*Aceite*		*20 gr*

Proceso culinario	Nº	Peso (en gramos)	Observaciones
1. Olla VACÍA		*1550 gr*	
2. Olla con COMIDA COCINADA		*2410 gr*	
**COMIDA COCINADA (2-1)**		*860 gr*	
3. Fiambrera VACÍA		*30 gr*	
4. Fiambrera con COMIDA COCINADA		*329 gr*	
**COMIDA ALMACENADA (4-3)**		*299 gr*	
5. Plato VACÍO		*284 gr*	
6. Plato con COMIDA COCINADA		*585 gr*	
**COMIDA QUE SIRVE A SU HIJO (6-5)**		*301 gr*	
7. Plato COMIDA QUE SU HIJO NO HA COMIDO		*321 gr*	
**COMIDA QUE SU HIJO HA COMIDO (6-7)**		*264 gr*	

MÉTODO DE ELABORACIÓN:
Explicar detalladamente el proceso de elaboración del plato

FICHA INGREDIENTES/PESOS "PLATO HECHO EN CASA (HC)"

ID: VAL 03

DÍA DEL CONSUMO: 18 / 10 / 2014 **HORA DEL CONSUMO:** 13:30

MÉTODO DE ELABORACIÓN:

Explicar detalladamente el proceso de elaboración del plato:

Se lavan y pelan las verduras.
Se lava el pollo y la ternera.
Cuando el agua está hirviendo, se echa el pollo y la ternera.
Transcurridos unos 15 minutos se echan las verduras.
Cocer durante unos 40 minutos y finalmente se echan los fideos durante 5 minutos.
Se tritura todo con un poco de agua de cocción y se añade la sal y el aceite.

Fotos:

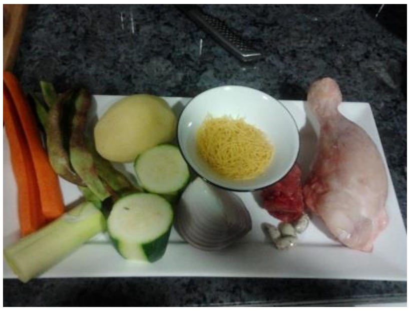

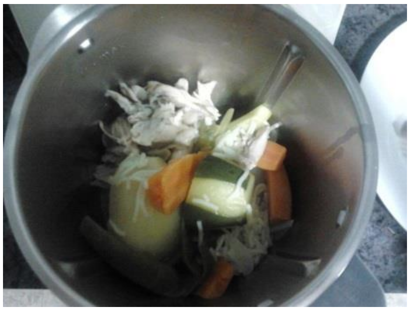

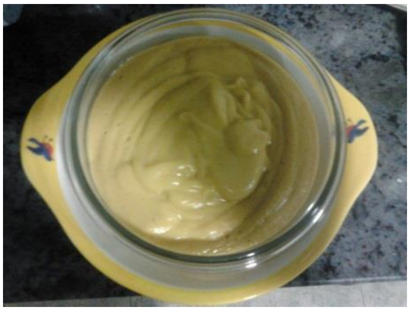

FICHA INGREDIENTES/PESOS “PLATO HECHO EN CASA (HC)” **ID: VAL 03**

DÍA DEL CONSUMO: *18 / 10 / 2014* HORA DEL CONSUMO: *20:30*

LA ELABOARACIÓN DEL PLATO SE HACE EL MISMO DÍA DEL CONSUMO [X] Sí [ ] No

Si la fecha de elaboración del plato NO es el mismo día del consumo NO se adjuntará foto y el peso de los ingredientes puede ser aproximado. Indique la fecha de elaboración:

TIPO DE PLATO: *MERLUZA CON ARROZ Y VERDURAS*

	Tipo de ingrediente	Nº	Peso (en gramos)
Ingrediente 1	*Merluza*		*77 gr*
Ingrediente 2	*Arroz*		*40 gr*
Ingrediente 3	*Judías verdes*	*8*	*92 gr*
Ingrediente 4	*Zanahoria*	*1*	*34 gr*
Ingrediente 5	*Puerro*	*¼*	*28 gr*
Ingrediente 6	*Calabacín*	*¼*	*68 gr*
Ingrediente 7	*Quesito*	*1*	*16 gr*
Ingrediente 8	*Aceite*		*15 gr*
Ingrediente 9	*Sal*		*3 gr*
Ingrediente 10			

Proceso culinario	Nº	Peso (en gramos)	Observaciones
1. Olla VACÍA		*1550 gr*	
2. Olla con COMIDA COCINADA		*2060 gr*	
**COMIDA COCINADA (2-1)**		*510 gr*	
3. Fiambrera VACÍA			
4. Fiambrera con COMIDA COCINADA			
**COMIDA ALMACENADA (4-3)**			
5. Plato VACÍO		*93 gr*	
6. Plato con COMIDA COCINADA		*360 gr*	
**COMIDA QUE SIRVE A SU HIJO (6-5)**		*267 gr*	
7. Plato COMIDA QUE SU HIJO NO HA COMIDO		*122 gr*	
**COMIDA QUE SU HIJO HA COMIDO (6-7)**		*238 gr*	

MÉTODO DE ELABORACIÓN:
Explicar detalladamente el proceso de elaboración del plato

FICHA INGREDIENTES/PESOS "PLATO HECHO EN CASA (HC)"

ID: VAL 03

DÍA DEL CONSUMO: 18 / 10 / 2014 **HORA DEL CONSUMO:** 20:30

MÉTODO DE ELABORACIÓN:

Explicar detalladamente el proceso de elaboración del plato:

Se pelan y cortan las verduras.

Cuando el agua está hirviendo, se echan las verduras, a los 20 minutos se echa el arroz y después de 10 minutos se echa la merluza, durante 10 minutos.

Transcurridos los 40 minutos, se tritura con un poco de agua de cocción y se le añade 1 quesito, el aceite y la sal.

Fotos:

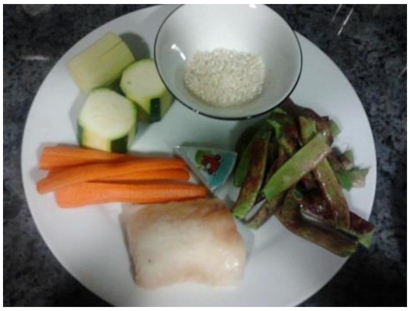

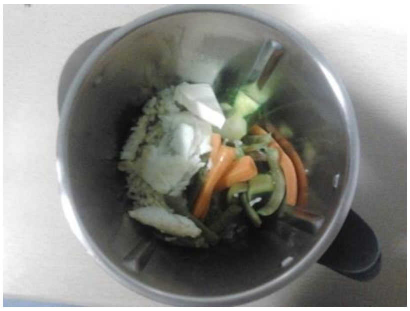

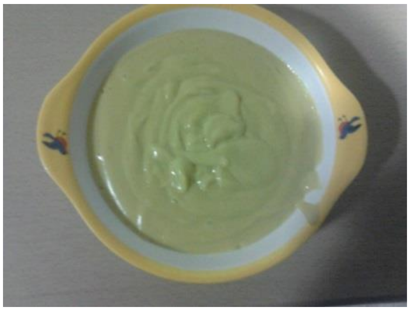

FICHA INGREDIENTES/PESOS "PLATO HECHO EN CASA (HC)" **ID: VAL 04**

DÍA DEL CONSUMO: *17 / 10 / 2014* HORA DEL CONSUMO: *12:30*

LA ELABOARACIÓN DEL PLATO SE HACE EL MISMO DÍA DEL CONSUMO [X] Sí [ ] No

Si la fecha de elaboración del plato NO es el mismo día del consumo NO se adjuntará foto y el peso de los ingredientes puede ser aproximado. Indique la fecha de elaboración:

TIPO DE PLATO: *ARROZ AL HORNO*

	Tipo de ingrediente	Nº	Peso (en gramos)
Ingrediente 1	*Aceite de oliva*	*1*	*1 chorro*
Ingrediente 2	*Patata*	*1*	*166 gr*
Ingrediente 3	*Tomate*	*1*	*75 gr*
Ingrediente 4	*Morcillas*	*2*	*80 gr*
Ingrediente 5	*Ajos*	*1 cabeza*	
Ingrediente 6	*Panceta*	*2 pequeñas*	*90 gr*
Ingrediente 7	*Costillas*		*105 gr*
Ingrediente 8	*Arroz*		*400 gr*
Ingrediente 9	*Garbanzos*		*220 gr*
Ingrediente 10	*Sal*		*1 pizca*

Proceso culinario	Nº	Peso (en gramos)	Observaciones
1. Olla VACÍA	*1*	*1580 gr*	
2. Olla con COMIDA COCINADA		*3529 gr*	
**COMIDA COCINADA (2-1)**		*1949 gr*	
3. Fiambrera VACÍA			
4. Fiambrera con COMIDA COCINADA			
**COMIDA ALMACENADA (4-3)**			
5. Plato VACÍO		*130 gr*	
6. Plato con COMIDA COCINADA		*230 gr*	
**COMIDA QUE SIRVE A SU HIJO (6-5)**		*100 gr*	
7. Plato COMIDA QUE SU HIJO NO HA COMIDO		*151 gr*	
**COMIDA QUE SU HIJO HA COMIDO (6-7)**		*79 gr*	

MÉTODO DE ELABORACIÓN:

Explicar detalladamente el proceso de elaboración del plato

FICHA INGREDIENTES/PESOS "PLATO HECHO EN CASA (HC)"

ID: VAL 04

DÍA DEL CONSUMO: 17 / 10 / 2014 **HORA DEL CONSUMO:** 12:30

MÉTODO DE ELABORACIÓN:

Explicar detalladamente el proceso de elaboración del plato:

Trocea la patata y el tomate.
Poner en la cazuela el aceite y el arroz y remover.
Añadir el caldo de polo y la sal.
Colocar el resto de ingredientes en la cazuela.
Meter la cazuela al horno durante 1 hora.

Fotos:

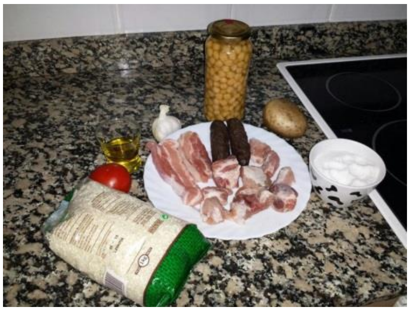

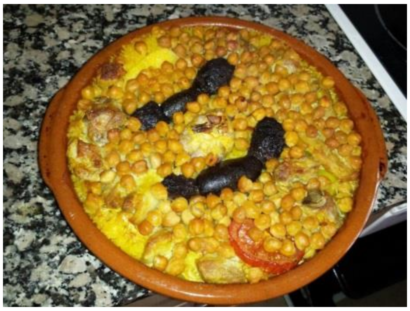

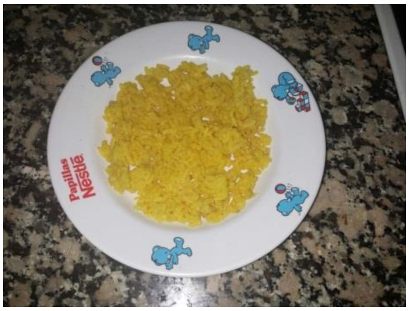

FICHA INGREDIENTES/PESOS "PLATO HECHO EN CASA (HC)" **ID: VAL 04**

DÍA DEL CONSUMO: *19 / 10 / 2014* HORA DEL CONSUMO: *13:20*

LA ELABOARACIÓN DEL PLATO SE HACE EL MISMO DÍA DEL CONSUMO ☐ Sí ☒ No

Si la fecha de elaboración del plato NO es el mismo día del consumo NO se adjuntará foto y el peso de los ingredientes puede ser aproximado. Indique la fecha de elaboración: 15-10-14

TIPO DE PLATO: *CALDO DE POLLO CON PASTA*

	Tipo de ingrediente	Nº	Peso (en gramos)
Ingrediente 1	*Letritas (pasta)*		*35 gr*
Ingrediente 2	*Sal*		*1 pizca*
Ingrediente 3	*Caldo*		*170 ml*
Ingrediente 4	*Zanahorias*	*3*	*165 gr*
Ingrediente 5	*Patata*	*1*	*100 gr*
Ingrediente 6	*Nabo*	*1*	*120 gr*
Ingrediente 7	*Chirivía*	*1*	*90 gr*
Ingrediente 8	*Puerro*	*1*	*75 gr*
Ingrediente 9	*Aceite*		*1 chorrito*
Ingrediente 10	*Pollo*		*400 gr*

Proceso culinario	Nº	Peso (en gramos)	Observaciones
1. Olla VACÍA	*1*	*427 gr*	
2. Olla con COMIDA COCINADA		*564 gr*	
**COMIDA COCINADA (2-1)**		*137 gr*	
3. Fiambrera VACÍA		*17 gr*	
4. Fiambrera con COMIDA COCINADA		*187 gr*	
**COMIDA ALMACENADA (4-3)**		*170 gr*	
5. Plato VACÍO		*130 gr*	
6. Plato con COMIDA COCINADA		*267 gr*	
**COMIDA QUE SIRVE A SU HIJO (6-5)**		*137 gr*	
7. Plato COMIDA QUE SU HIJO NO HA COMIDO		*130 gr*	
**COMIDA QUE SU HIJO HA COMIDO (6-7)**		*137 gr*	

MÉTODO DE ELABORACIÓN:

Explicar detalladamente el proceso de elaboración del plato

FICHA INGREDIENTES/PESOS "PLATO HECHO EN CASA (HC)"

ID: VAL 04

DÍA DEL CONSUMO: 19 / 10 / 2014 **HORA DEL CONSUMO:** 13:20

MÉTODO DE ELABORACIÓN:

Explicar detalladamente el proceso de elaboración del plato:

Trocea todas las verduras.
Llenar la olla de agua, hasta que cubra el pollo.
Añadir sal, aceite y las verduras troceadas.
Dejar cocer a fuego lento durante una hora y media.

Fotos:

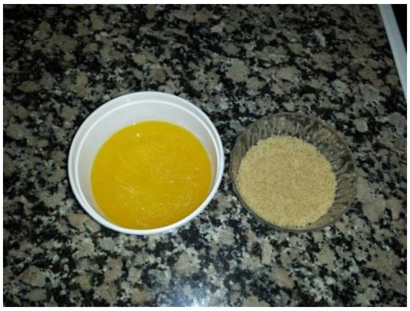

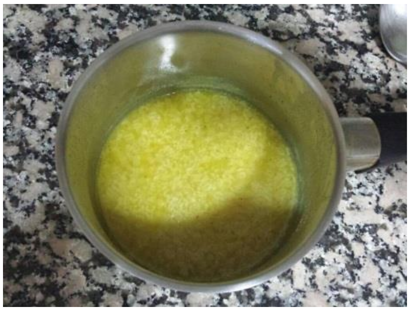

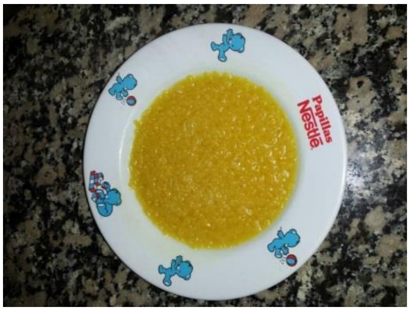

FICHA INGREDIENTES/PESOS "PLATO HECHO EN CASA (HC)" **ID: VAL 04**

DÍA DEL CONSUMO: *19 / 10 / 2014* HORA DEL CONSUMO: *21:00*

LA ELABOARACIÓN DEL PLATO SE HACE EL MISMO DÍA DEL CONSUMO [X] Sí [ ] No

Si la fecha de elaboración del plato NO es el mismo día del consumo NO se adjuntará foto y el peso de los ingredientes puede ser aproximado. Indique la fecha de elaboración:

TIPO DE PLATO: *VERDURA CON LOMO*

	Tipo de ingrediente	Nº	Peso (en gramos)
Ingrediente 1	*Zanahoria*	*1*	*71 gr*
Ingrediente 2	*Patata*	*1*	*135 gr*
Ingrediente 3	*Calabacín*	*½*	*158 gr*
Ingrediente 4	*Lomo*	*2 lonchas*	*20 gr*
Ingrediente 5			
Ingrediente 6			
Ingrediente 7			
Ingrediente 8			
Ingrediente 9			
Ingrediente 10			

Proceso culinario	Nº	Peso (en gramos)	Observaciones
1. Olla VACÍA	*1*	*1084 gr*	
2. Olla con COMIDA COCINADA		*1573 gr*	
**COMIDA COCINADA (2-1)**		*489 gr*	
3. Fiambrera VACÍA			
4. Fiambrera con COMIDA COCINADA			
**COMIDA ALMACENADA (4-3)**			
5. Plato VACÍO		*84 gr*	
6. Plato con COMIDA COCINADA		*190 gr*	
**COMIDA QUE SIRVE A SU HIJO (6-5)**		*106 gr*	
7. Plato COMIDA QUE SU HIJO NO HA COMIDO		*134 gr*	
**COMIDA QUE SU HIJO HA COMIDO (6-7)**		*56 gr*	

MÉTODO DE ELABORACIÓN:

Explicar detalladamente el proceso de elaboración del plato

FICHA INGREDIENTES/PESOS "PLATO HECHO EN CASA (HC)"

ID: VAL 04

DÍA DEL CONSUMO: 19 / 10 / 2014 **HORA DEL CONSUMO:** 21:00

MÉTODO DE ELABORACIÓN:

Explicar detalladamente el proceso de elaboración del plato:

Trocea las verduras.
Llenar la olla de agua y poner las verduras troceadas, aceite y sal.
Cocer a fuego lento hasta que estén tiernas.
Hacer el lomo a la plancha.

Fotos:

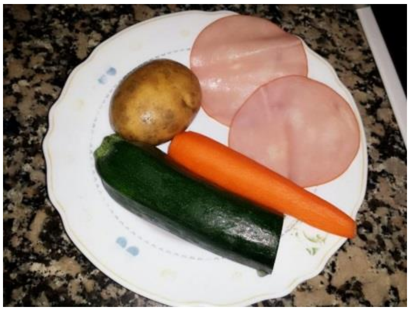

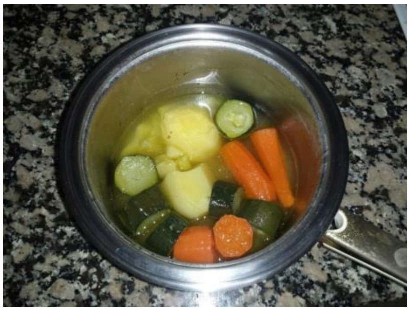

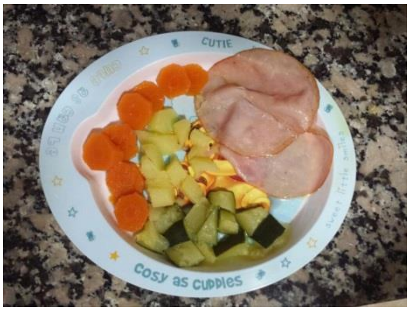

FICHA INGREDIENTES/PESOS "PLATO HECHO EN CASA (HC)" **ID: VAL 05**

DÍA DEL CONSUMO: *17 / 10 / 2014* HORA DEL CONSUMO: *13:00*

LA ELABOARACIÓN DEL PLATO SE HACE EL MISMO DÍA DEL CONSUMO [X] Sí [ ] No

Si la fecha de elaboración del plato NO es el mismo día del consumo NO se adjuntará foto y el peso de los ingredientes puede ser aproximado. Indique la fecha de elaboración:

TIPO DE PLATO: *PURÉ DE POLLO Y VERDURAS*

	Tipo de ingrediente	Nº	Peso (en gramos)
Ingrediente 1	*Muslo de pollo*	*1*	*250 gr*
Ingrediente 2	*Entrepierna de pollo*	*1*	*150 gr*
Ingrediente 3	*Ternera*	*1*	*100 gr*
Ingrediente 4	*Zanahorias*	*2*	*230 gr*
Ingrediente 5	*Bachoqueta*	*1*	*150 gr*
Ingrediente 6	*Patata*	*2*	*250 gr*
Ingrediente 7	*Cebollas*	*2*	*200 gr*
Ingrediente 8	*Calabacín*	*1*	*175 gr*
Ingrediente 9	*Aceite*	*1*	*Cucharada*
Ingrediente 10			

Proceso culinario	Nº	Peso (en gramos)	Observaciones
1. Olla VACÍA	*1*	*630 gr*	
2. Olla con COMIDA COCINADA	*1*	*3000 gr*	
**COMIDA COCINADA (2-1)**		*2370 gr*	
3. Fiambrera VACÍA	*3*	*28 gr*	
4. Fiambrera con COMIDA COCINADA	*3*	*130 gr*	
**COMIDA ALMACENADA (4-3)**	*3*	*102 gr*	
5. Plato VACÍO	*1*	*58 gr*	
6. Plato con COMIDA COCINADA	*1*	*160 gr*	
**COMIDA QUE SIRVE A SU HIJO (6-5)**	*1*	*102 gr*	
7. Plato COMIDA QUE SU HIJO NO HA COMIDO		*58 gr*	
**COMIDA QUE SU HIJO HA COMIDO (6-7)**		*102 gr*	

MÉTODO DE ELABORACIÓN:

Explicar detalladamente el proceso de elaboración del plato

FICHA INGREDIENTES/PESOS "PLATO HECHO EN CASA (HC)"

ID: VAL 05

DÍA DEL CONSUMO: 17 / 10 / 2014 **HORA DEL CONSUMO:** 13:00

MÉTODO DE ELABORACIÓN:

Explicar detalladamente el proceso de elaboración del plato:

Pelo y troceo las verduras.
Pongo en la olla agua del grifo (osmosis).
Le añado las verduras troceadas, la carne y una cucharada de aceite de oliva marca Hacendado.
Lo dejo en cocción durante 2 horas y medio o 3 horas.
Una vez cocido, procedo a pasar los ingredientes y los bato (trituro).
Una vez terminado todo el proceso, me guardo fiambreras en el congelador.

Fotos:

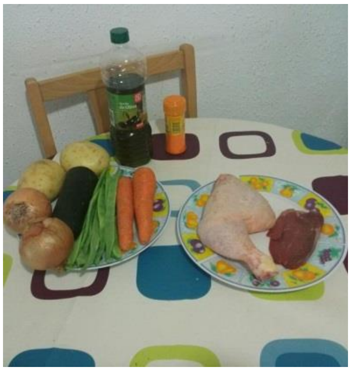

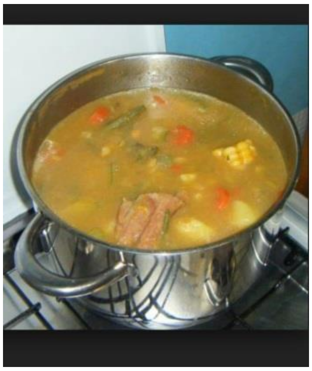

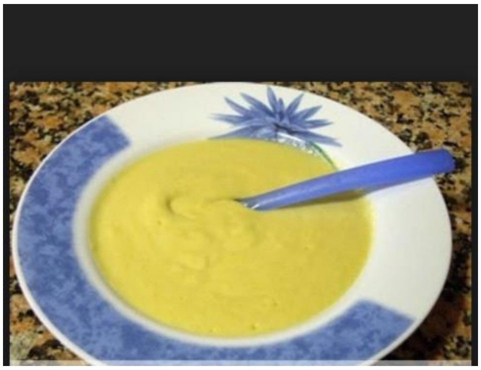

FICHA INGREDIENTES/PESOS “PLATO HECHO EN CASA (HC)” **ID: VAL 05**

DÍA DEL CONSUMO: *17 / 10 / 2014* HORA DEL CONSUMO: *21:00*

LA ELABOARACIÓN DEL PLATO SE HACE EL MISMO DÍA DEL CONSUMO [X] Sí [ ] No

Si la fecha de elaboración del plato NO es el mismo día del consumo NO se adjuntará foto y el peso de los ingredientes puede ser aproximado. Indique la fecha de elaboración:

TIPO DE PLATO: *PURÉ DE MERLUZA CON VERDURAS*

	Tipo de ingrediente	Nº	Peso (en gramos)
Ingrediente 1	*Merluza*	*1*	*250 gr*
Ingrediente 2	*Zanahorias*	*2*	*230 gr*
Ingrediente 3	*Bachoqueta*	*1*	*150 gr*
Ingrediente 4	*Patatas*	*1*	*250 gr*
Ingrediente 5	*Cebollas*	*1*	*200 gr*
Ingrediente 6	*Calabacín*	*1*	*175 gr*
Ingrediente 7	*Aceite*	*1*	*Cucharada*
Ingrediente 8			
Ingrediente 9			
Ingrediente 10			

Proceso culinario	Nº	Peso (en gramos)	Observaciones
1. Olla VACÍA	*1*	*630 gr*	
2. Olla con COMIDA COCINADA	*1*	*2420 gr*	
**COMIDA COCINADA (2-1)**	*1*	*1790 gr*	
3. Fiambrera VACÍA	*3*	*28 gr*	
4. Fiambrera con COMIDA COCINADA	*3*	*130 gr*	
**COMIDA ALMACENADA (4-3)**	*3*	*102 gr*	
5. Plato VACÍO	*1*	*58 gr*	
6. Plato con COMIDA COCINADA	*1*	*178 gr*	
**COMIDA QUE SIRVE A SU HIJO (6-5)**	*1*	*120 gr*	
7. Plato COMIDA QUE SU HIJO NO HA COMIDO		*58 gr*	
**COMIDA QUE SU HIJO HA COMIDO (6-7)**		*120 gr*	

MÉTODO DE ELABORACIÓN:
Explicar detalladamente el proceso de elaboración del plato

FICHA INGREDIENTES/PESOS “PLATO HECHO EN CASA (HC)” **ID: VAL 05**

DÍA DEL CONSUMO: 17 / 10 / 2014 **HORA DEL CONSUMO:** 21:00

MÉTODO DE ELABORACIÓN:

Explicar detalladamente el proceso de elaboración del plato:

Pelo y troceo las verduras, les añado una cucharada de aceite de oliva.

Dejo hirviendo las verduras.

A la hora y media añado los filetes de merluza y los dejo en cocción junto con las verduras, así durante 30-40 minutos.

Una vez terminada su cocción, las voy sacando al vaso de la batidora y lo bato todo junto.

Fotos:

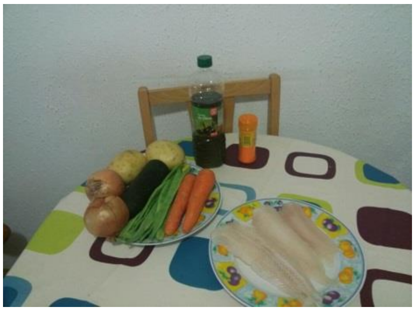

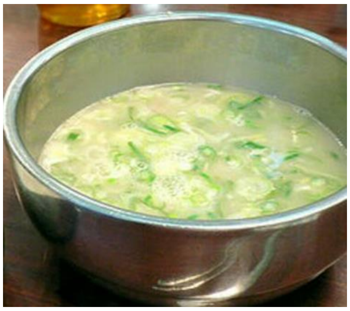

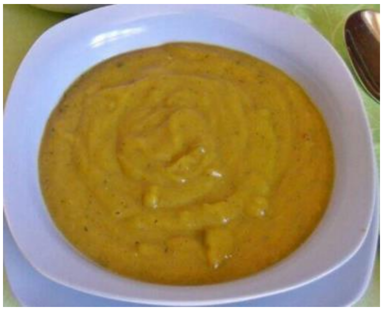

FICHA INGREDIENTES/PESOS “PLATO HECHO EN CASA (HC)” **ID: VAL 05**

DÍA DEL CONSUMO: *19 / 10 / 2014* HORA DEL CONSUMO: *13:00*

LA ELABOARACIÓN DEL PLATO SE HACE EL MISMO DÍA DEL CONSUMO ☐ Sí ☒ No

Si la fecha de elaboración del plato NO es el mismo día del consumo NO se adjuntará foto y el peso de los ingredientes puede ser aproximado. Indique la fecha de elaboración: 17-10-14

TIPO DE PLATO: *PURÉ DE POLLO Y VERDURAS*

	Tipo de ingrediente	Nº	Peso (en gramos)
Ingrediente 1	*Muslo de pollo*	*1*	*250 gr*
Ingrediente 2	*Entrepierna de pollo*	*1*	*150 gr*
Ingrediente 3	*Ternera*	*1*	*100 gr*
Ingrediente 4	*Zanahoria*	*2*	*230 gr*
Ingrediente 5	*Bachoqueta*	*1*	*150 gr*
Ingrediente 6	*Patata*	*2*	*250 gr*
Ingrediente 7	*Cebolla*	*2*	*200 gr*
Ingrediente 8	*Calabacín*	*1*	*175 gr*
Ingrediente 9	*Aceite*	*1*	*Cucharada*
Ingrediente 10			

Proceso culinario	Nº	Peso (en gramos)	Observaciones
1. Olla VACÍA			
2. Olla con COMIDA COCINADA			
**COMIDA COCINADA (2-1)**			
3. Fiambrera VACÍA			
4. Fiambrera con COMIDA COCINADA			
**COMIDA ALMACENADA (4-3)**			
5. Plato VACÍO			
6. Plato con COMIDA COCINADA			
**COMIDA QUE SIRVE A SU HIJO (6-5)**			
7. Plato COMIDA QUE SU HIJO NO HA COMIDO			
**COMIDA QUE SU HIJO HA COMIDO (6-7)**			

MÉTODO DE ELABORACIÓN:

Explicar detalladamente el proceso de elaboración del plato

FICHA INGREDIENTES/PESOS "PLATO HECHO EN CASA (HC)"

ID: VAL 05

DÍA DEL CONSUMO: 19 / 10 / 2014 **HORA DEL CONSUMO:** 13:00

MÉTODO DE ELABORACIÓN:

Explicar detalladamente el proceso de elaboración del plato:

ALIMENTO PREPARADO ANTERIORMENTE Y SACADO DEL CONGELADOR

Fotos:

FICHA INGREDIENTES/PESOS "PLATO HECHO EN CASA (HC)" **ID: VAL 05**

DÍA DEL CONSUMO: *19 / 10 / 2014* HORA DEL CONSUMO: *21:00*

LA ELABOARACIÓN DEL PLATO SE HACE EL MISMO DÍA DEL CONSUMO ☐ Sí ☒ No

Si la fecha de elaboración del plato NO es el mismo día del consumo NO se adjuntará foto y el peso de los ingredientes puede ser aproximado. Indique la fecha de elaboración: 17-10-14

TIPO DE PLATO: *PURÉ DE MERLUZA Y VERDURAS*

	Tipo de ingrediente	Nº	Peso (en gramos)
Ingrediente 1	*Merluza*	*1*	*250 gr*
Ingrediente 2	*Bachoqueta*	*2*	*230 gr*
Ingrediente 3	*Zanahoria*	*1*	*150 gr*
Ingrediente 4	*Patata*	*1*	*250 gr*
Ingrediente 5	*Cebolla*	*1*	*200 gr*
Ingrediente 6	*Calabacín*	*1*	*175 gr*
Ingrediente 7	*Aceite*	*1*	*Cucharada*
Ingrediente 8			
Ingrediente 9			
Ingrediente 10			

Proceso culinario	Nº	Peso (en gramos)	Observaciones
1. Olla VACÍA			
2. Olla con COMIDA COCINADA			
**COMIDA COCINADA (2-1)**			
3. Fiambrera VACÍA			
4. Fiambrera con COMIDA COCINADA			
**COMIDA ALMACENADA (4-3)**			
5. Plato VACÍO			
6. Plato con COMIDA COCINADA			
**COMIDA QUE SIRVE A SU HIJO (6-5)**			
7. Plato COMIDA QUE SU HIJO NO HA COMIDO			
**COMIDA QUE SU HIJO HA COMIDO (6-7)**			

MÉTODO DE ELABORACIÓN:
Explicar detalladamente el proceso de elaboración del plato

FICHA INGREDIENTES/PESOS “PLATO HECHO EN CASA (HC)”

ID: VAL 05

DÍA DEL CONSUMO: 19 / 10 / 2014 **HORA DEL CONSUMO:** 21:00

MÉTODO DE ELABORACIÓN:

Explicar detalladamente el proceso de elaboración del plato:

ALIMENTO PREPARADO ANTERIORMENTE Y SACADO DEL CONGELADOR

Fotos:

FICHA INGREDIENTES/PESOS "PLATO HECHO EN CASA (HC)" **ID: VAL 05**

DÍA DEL CONSUMO: *20 / 10 / 2014* HORA DEL CONSUMO: *13:00*

LA ELABOARACIÓN DEL PLATO SE HACE EL MISMO DÍA DEL CONSUMO ☐ Sí ☒ No

Si la fecha de elaboración del plato NO es el mismo día del consumo NO se adjuntará foto y el peso de los ingredientes puede ser aproximado. Indique la fecha de elaboración: 17/10/14

TIPO DE PLATO: *PURÉ DE POLLO Y VERDURAS*

	Tipo de ingrediente	Nº	Peso (en gramos)
Ingrediente 1	*Muslo de pollo*	*1*	*250 gr*
Ingrediente 2	*Entremuslo de pollo*	*1*	*150 gr*
Ingrediente 3	*Ternera*	*1*	*100 gr*
Ingrediente 4	*Zanahoria*	*2*	*230 gr*
Ingrediente 5	*Bachoqueta*	*1*	*150 gr*
Ingrediente 6	*Patata*	*2*	*250 gr*
Ingrediente 7	*Cebolla*	*2*	*200 gr*
Ingrediente 8	*Calabacín*	*1*	*175 gr*
Ingrediente 9	*Aceite*	*1*	*Cucharada*
Ingrediente 10			

Proceso culinario	Nº	Peso (en gramos)	Observaciones
1. Olla VACÍA			
2. Olla con COMIDA COCINADA			
**COMIDA COCINADA (2-1)**			
3. Fiambrera VACÍA			
4. Fiambrera con COMIDA COCINADA			
**COMIDA ALMACENADA (4-3)**			
5. Plato VACÍO			
6. Plato con COMIDA COCINADA			
**COMIDA QUE SIRVE A SU HIJO (6-5)**			
7. Plato COMIDA QUE SU HIJO NO HA COMIDO			
**COMIDA QUE SU HIJO HA COMIDO (6-7)**			

MÉTODO DE ELABORACIÓN:
Explicar detalladamente el proceso de elaboración del plato

FICHA INGREDIENTES/PESOS "PLATO HECHO EN CASA (HC)"

ID: VAL 05

DÍA DEL CONSUMO: 20 / 10 / 2014 **HORA DEL CONSUMO:** 13:00

MÉTODO DE ELABORACIÓN:

Explicar detalladamente el proceso de elaboración del plato:

ALIMENTO PREPARADO ANTERIORMENTE Y SACADO DEL CONGELADOR

Fotos:

FICHA INGREDIENTES/PESOS “PLATO HECHO EN CASA (HC)” **ID: VAL 05**

DÍA DEL CONSUMO: *20 / 10 / 2014* HORA DEL CONSUMO: *20:30*

LA ELABOARACIÓN DEL PLATO SE HACE EL MISMO DÍA DEL CONSUMO ☐ Sí ☒ No

Si la fecha de elaboración del plato NO es el mismo día del consumo NO se adjuntará foto y el peso de los ingredientes puede ser aproximado. Indique la fecha de elaboración:

TIPO DE PLATO: *PURÉ DE MERLUZA Y VERDURAS*

	Tipo de ingrediente	Nº	Peso (en gramos)
Ingrediente 1	*Merluza*	*1*	*250 gr*
Ingrediente 2	*Zanahoria*	*2*	*230 gr*
Ingrediente 3	*Bachoqueta*	*1*	*150 gr*
Ingrediente 4	*Patata*	*1*	*250 gr*
Ingrediente 5	*Cebolla*	*1*	*200 gr*
Ingrediente 6	*Calabacín*	*1*	*175 gr*
Ingrediente 7	*Aceite*	*1*	*Cucharada*
Ingrediente 8			
Ingrediente 9			
Ingrediente 10			

Proceso culinario	Nº	Peso (en gramos)	Observaciones
1. Olla VACÍA			
2. Olla con COMIDA COCINADA			
**COMIDA COCINADA (2-1)**			
3. Fiambrera VACÍA			
4. Fiambrera con COMIDA COCINADA			
**COMIDA ALMACENADA (4-3)**			
5. Plato VACÍO			
6. Plato con COMIDA COCINADA			
**COMIDA QUE SIRVE A SU HIJO (6-5)**			
7. Plato COMIDA QUE SU HIJO NO HA COMIDO			
**COMIDA QUE SU HIJO HA COMIDO (6-7)**			

MÉTODO DE ELABORACIÓN:

Explicar detalladamente el proceso de elaboración del plato

FICHA INGREDIENTES/PESOS “PLATO HECHO EN CASA (HC)”

ID: VAL 05

DÍA DEL CONSUMO: 20 / 10 / 2014 **HORA DEL CONSUMO:** 20:30

MÉTODO DE ELABORACIÓN:

Explicar detalladamente el proceso de elaboración del plato:

ALIMENTO PREPARADO ANTERIORMENTE Y SACADO DEL CONGELADOR

Fotos:

FICHA INGREDIENTES/PESOS "PLATO HECHO EN CASA (HC)" **ID: VAL 06**

DÍA DEL CONSUMO: *14 / 10 / 2014* HORA DEL CONSUMO: *13:00*

LA ELABOARACIÓN DEL PLATO SE HACE EL MISMO DÍA DEL CONSUMO [X] Sí [ ] No

Si la fecha de elaboración del plato NO es el mismo día del consumo NO se adjuntará foto y el peso de los ingredientes puede ser aproximado. Indique la fecha de elaboración:

TIPO DE PLATO: *PURÉ DE VERDURAS CON TERNERA*

	Tipo de ingrediente	Nº	Peso (en gramos)
Ingrediente 1	*Patata*	*1*	*290 gr*
Ingrediente 2	*Calabacín*	*½*	*176 gr*
Ingrediente 3	*Zanahoria*	*1*	*67 gr*
Ingrediente 4	*Cebolla*	*1*	*256 gr*
Ingrediente 5	*Judía verde*	*10*	*187 gr*
Ingrediente 6	*Ternera*	*2 cortadas*	*155 gr*
Ingrediente 7	*Aceite de oliva virgen extra*	*1*	*1 cucharada*
Ingrediente 8			
Ingrediente 9			
Ingrediente 10			

Proceso culinario	Nº	Peso (en gramos)	Observaciones
1. Olla VACÍA		*1695 gr*	
2. Olla con COMIDA COCINADA		*3417 gr*	
**COMIDA COCINADA (2-1)**		*1722 gr*	
3. Fiambrera VACÍA		*44 gr*	
4. Fiambrera con COMIDA COCINADA		*356 gr*	
**COMIDA ALMACENADA (4-3)**		*312 gr*	
5. Plato VACÍO		*93 gr*	
6. Plato con COMIDA COCINADA		*426 gr*	
**COMIDA QUE SIRVE A SU HIJO (6-5)**		*333 gr*	
7. Plato COMIDA QUE SU HIJO NO HA COMIDO		*276 gr*	
**COMIDA QUE SU HIJO HA COMIDO (6-7)**		*150 gr*	

MÉTODO DE ELABORACIÓN:

Explicar detalladamente el proceso de elaboración del plato

FICHA INGREDIENTES/PESOS "PLATO HECHO EN CASA (HC)" **ID: VAL 06**

DÍA DEL CONSUMO: 14 / 10 / 2014 **HORA DEL CONSUMO:** 13:00

MÉTODO DE ELABORACIÓN:
Explicar detalladamente el proceso de elaboración del plato:

Pelar y cortar todas las verduras.
Asamos la ternera en una sartén.
Hervir las verduras durante 30 minutos.
Trituramos verduras con agua de la cocción, añadiendo las dos cortadas de solomillo de ternera y una cucharada de aceite.

Fotos:

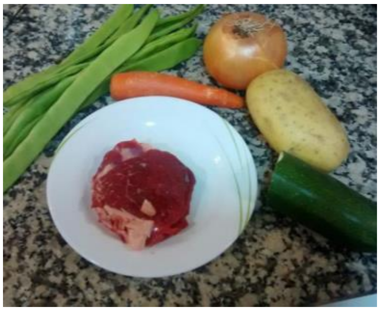
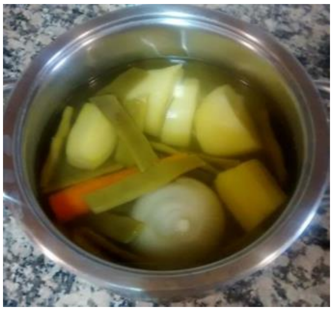
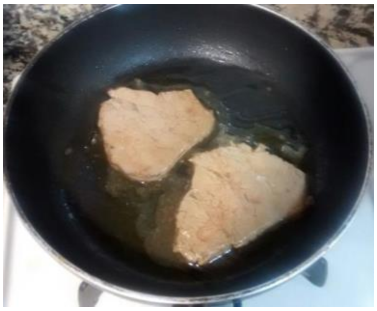
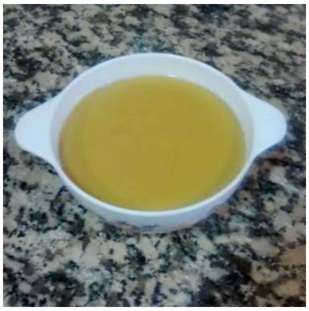

FICHA INGREDIENTES/PESOS "PLATO HECHO EN CASA (HC)" **ID: VAL 06**

DÍA DEL CONSUMO: *16 / 10 / 2014* HORA DEL CONSUMO: *14:30*

LA ELABOARACIÓN DEL PLATO SE HACE EL MISMO DÍA DEL CONSUMO ☒ Sí ☐ No

Si la fecha de elaboración del plato NO es el mismo día del consumo NO se adjuntará foto y el peso de los ingredientes puede ser aproximado. Indique la fecha de elaboración:

TIPO DE PLATO: *PURÉ DE VERDURAS CON POLLO*

	Tipo de ingrediente	Nº	Peso (en gramos)
Ingrediente 1	*Aceite de oliva virgen extra*	*1*	*1 cucharada y media*
Ingrediente 2	*Patata*	*1*	*150 gr*
Ingrediente 3	*Cebolla*	*1*	*195 gr*
Ingrediente 4	*Zanahoria*	*1*	*48 gr*
Ingrediente 5	*Tomate*	*1*	*136 gr*
Ingrediente 6	*Judías verdes*	*6*	*142 gr*
Ingrediente 7	*Calabacín*	*½*	*170 gr*
Ingrediente 8	*Muslos de pollo*	*2*	*304 gr*
Ingrediente 9			
Ingrediente 10			

Proceso culinario	Nº	Peso (en gramos)	Observaciones
1. Olla VACÍA		*1686 gr*	
2. Olla con COMIDA COCINADA		*3527 gr*	
**COMIDA COCINADA (2-1)**		*1841 gr*	
3. Fiambrera VACÍA		*35 gr*	
4. Fiambrera con COMIDA COCINADA		*249 gr*	
**COMIDA ALMACENADA (4-3)**		*214 gr*	
5. Plato VACÍO		*460 gr*	
6. Plato con COMIDA COCINADA		*704 gr*	
**COMIDA QUE SIRVE A SU HIJO (6-5)**		*244 gr*	
7. Plato COMIDA QUE SU HIJO NO HA COMIDO		*536 gr*	
**COMIDA QUE SU HIJO HA COMIDO (6-7)**		*168 gr*	

MÉTODO DE ELABORACIÓN:
Explicar detalladamente el proceso de elaboración del plato

FICHA INGREDIENTES/PESOS "PLATO HECHO EN CASA (HC)"

ID: VAL 06

DÍA DEL CONSUMO: 16 / 10 / 2014 **HORA DEL CONSUMO:** 14:30

MÉTODO DE ELABORACIÓN:

Explicar detalladamente el proceso de elaboración del plato:

Pelar y cortar todas las verduras.
Quitar la piel a los dos muslos de pollo.
Hervir el pollo y las verduras durante 30 minutos.
Triturar todo con un poco de agua de la cocción.
Añadir cucharada de aceite.

Fotos:

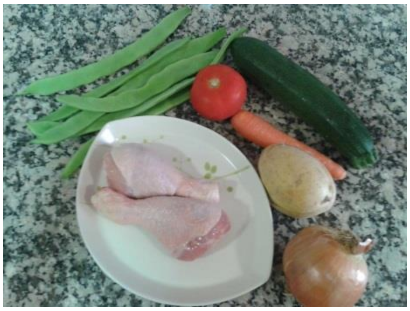
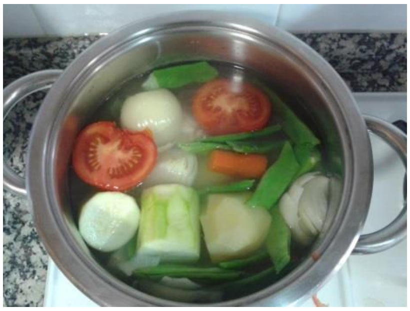
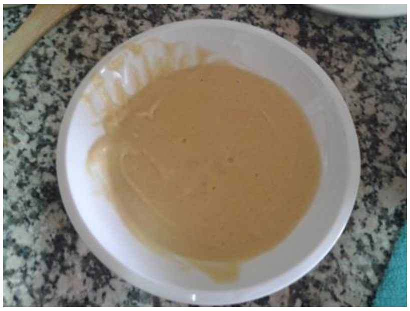

FICHA INGREDIENTES/PESOS "PLATO HECHO EN CASA (HC)" **ID: VAL 06**

DÍA DEL CONSUMO: *18 / 10 / 2014* HORA DEL CONSUMO: *13:45*

LA ELABOARACIÓN DEL PLATO SE HACE EL MISMO DÍA DEL CONSUMO [X] Sí [ ] No

Si la fecha de elaboración del plato NO es el mismo día del consumo NO se adjuntará foto y el peso de los ingredientes puede ser aproximado. Indique la fecha de elaboración:

TIPO DE PLATO: *PURÉ DE VERDURAS CON PESCADO*

	Tipo de ingrediente	Nº	Peso (en gramos)
Ingrediente 1	*Aceite de oliva virgen extra*	*1*	*Una cucharada*
Ingrediente 2	*Patata*	*1*	*244 gr*
Ingrediente 3	*Cebolla*	*1*	*177 gr*
Ingrediente 4	*Calabacín*	*½*	*131 gr*
Ingrediente 5	*Judías verdes*	*9*	*124 gr*
Ingrediente 6	*Merluza (lomos)*	*2*	*155 gr*
Ingrediente 7	*Zanahorias*	*2*	*57 gr*
Ingrediente 8	*Tomate*	*1*	*136 gr*
Ingrediente 9			
Ingrediente 10			

Proceso culinario	Nº	Peso (en gramos)	Observaciones
1. Olla VACÍA		*1679 gr*	
2. Olla con COMIDA COCINADA		*3462 gr*	
**COMIDA COCINADA (2-1)**		*1783 gr*	
3. Fiambrera VACÍA		*35 gr*	
4. Fiambrera con COMIDA COCINADA		*292 gr*	
**COMIDA ALMACENADA (4-3)**		*257 gr*	
5. Plato VACÍO		*475 gr*	
6. Plato con COMIDA COCINADA		*721 gr*	
**COMIDA QUE SIRVE A SU HIJO (6-5)**		*246 gr*	
7. Plato COMIDA QUE SU HIJO NO HA COMIDO		*535 gr*	
**COMIDA QUE SU HIJO HA COMIDO (6-7)**		*186 gr*	

MÉTODO DE ELABORACIÓN:
Explicar detalladamente el proceso de elaboración del plato

FICHA INGREDIENTES/PESOS "PLATO HECHO EN CASA (HC)"

ID: VAL 06

DÍA DEL CONSUMO: 18 / 10 / 2014 **HORA DEL CONSUMO:** 13:45

MÉTODO DE ELABORACIÓN:

Explicar detalladamente el proceso de elaboración del plato:

Pelar y cortar todas las verduras.

Hervir las verduras junto con los dos lomos de merluza durante 30 minutos.

Triturar todo con un poco de agua de la cocción, añadiendo una cucharada de aceite.

Fotos:

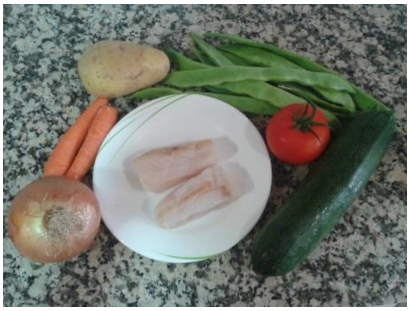

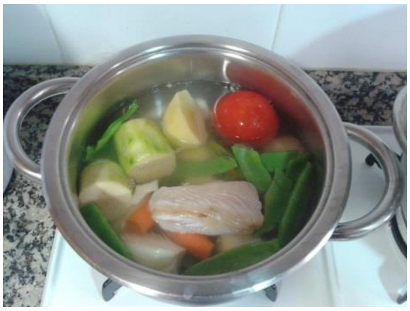

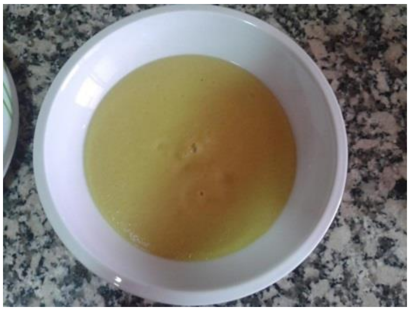

FICHA INGREDIENTES/PESOS "PLATO HECHO EN CASA (HC)" **ID: VAL 06**

DÍA DEL CONSUMO: *18 / 10 / 2014* **HORA DEL CONSUMO:** *18:00*

LA ELABOARACIÓN DEL PLATO SE HACE EL MISMO DÍA DEL CONSUMO [X] Sí [ ] No

Si la fecha de elaboración del plato NO es el mismo día del consumo NO se adjuntará foto y el peso de los ingredientes puede ser aproximado. Indique la fecha de elaboración:

TIPO DE PLATO: *PAPILLA DE FRUTAS*

	Tipo de ingrediente	Nº	Peso (en gramos)
Ingrediente 1	*Plátano*	*1*	*85 gr*
Ingrediente 2	*Pera*	*1*	*225 gr*
Ingrediente 3	*Manzana*	*½*	*120 gr*
Ingrediente 4	*Galleta*	*1*	*7 gr*
Ingrediente 5			
Ingrediente 6			
Ingrediente 7			
Ingrediente 8			
Ingrediente 9			
Ingrediente 10			

Proceso culinario	Nº	Peso (en gramos)	Observaciones
1. Olla VACÍA			
2. Olla con COMIDA COCINADA		*450 gr*	
**COMIDA COCINADA (2-1)**			
3. Fiambrera VACÍA			
4. Fiambrera con COMIDA COCINADA			
**COMIDA ALMACENADA (4-3)**			
5. Plato VACÍO		*121 gr*	
6. Plato con COMIDA COCINADA		*246 gr*	
**COMIDA QUE SIRVE A SU HIJO (6-5)**		*125 gr*	
7. Plato COMIDA QUE SU HIJO NO HA COMIDO		*170 gr*	
**COMIDA QUE SU HIJO HA COMIDO (6-7)**		*76 gr*	

MÉTODO DE ELABORACIÓN:

Explicar detalladamente el proceso de elaboración del plato

FICHA INGREDIENTES/PESOS "PLATO HECHO EN CASA (HC)"

ID: VAL 06

DÍA DEL CONSUMO: 18 / 10 / 2014 **HORA DEL CONSUMO:** 18:00

MÉTODO DE ELABORACIÓN:

Explicar detalladamente el proceso de elaboración del plato:

Pelar y trocear la fruta.
Triturar la fruta junto con la galleta.

Fotos:

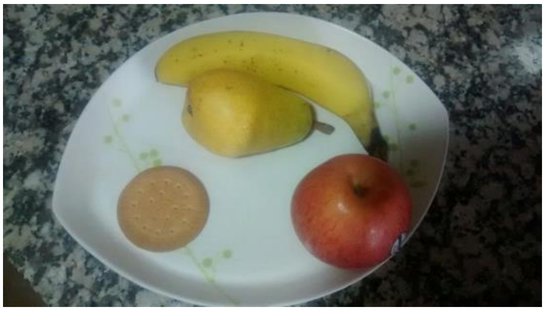

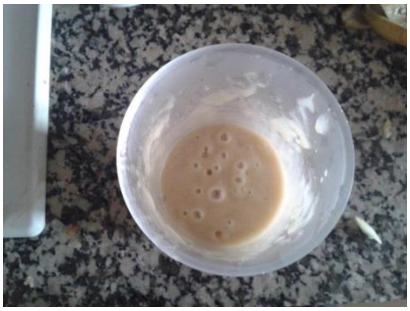

FICHA INGREDIENTES/PESOS “PLATO HECHO EN CASA (HC)” **ID: VAL 07**

DÍA DEL CONSUMO: *14 / 10 / 2014* HORA DEL CONSUMO: *12:00*

LA ELABOARACIÓN DEL PLATO SE HACE EL MISMO DÍA DEL CONSUMO [X] Sí [ ] No

Si la fecha de elaboración del plato NO es el mismo día del consumo NO se adjuntará foto y el peso de los ingredientes puede ser aproximado. Indique la fecha de elaboración:

TIPO DE PLATO: *PURÉ DE VERDURAS CON POLLO*

	Tipo de ingrediente	Nº	Peso (en gramos)
Ingrediente 1	*Calabacín*	*Medio*	*300 gr*
Ingrediente 2	*Judía baby*		*212 gr*
Ingrediente 3	*Patata*	*2*	*298 gr*
Ingrediente 4	*Cebolla*	*1*	*100 gr*
Ingrediente 5	*Pollo*	*1*	*253 gr*
Ingrediente 6	*Guisantes*		*62 gr*
Ingrediente 7			
Ingrediente 8			
Ingrediente 9			
Ingrediente 10			

Proceso culinario	Nº	Peso (en gramos)	Observaciones
1. Olla VACÍA	*1*	*1202 gr*	
2. Olla con COMIDA COCINADA		*1801 gr*	
**COMIDA COCINADA (2-1)**		*599 gr*	
3. Fiambrera VACÍA			
4. Fiambrera con COMIDA COCINADA			
**COMIDA ALMACENADA (4-3)**			
5. Plato VACÍO		*412 gr*	
6. Plato con COMIDA COCINADA		*672 gr*	*Con un chorrito de aceite de oliva*
**COMIDA QUE SIRVE A SU HIJO (6-5)**		*260 gr*	
7. Plato COMIDA QUE SU HIJO NO HA COMIDO		*412 gr*	
**COMIDA QUE SU HIJO HA COMIDO (6-7)**		*260 gr*	

MÉTODO DE ELABORACIÓN:

Explicar detalladamente el proceso de elaboración del plato

FICHA INGREDIENTES/PESOS "PLATO HECHO EN CASA (HC)"

ID: VAL 07

DÍA DEL CONSUMO: 14 / 10 / 2014 **HORA DEL CONSUMO:** 12:00

MÉTODO DE ELABORACIÓN:

Explicar detalladamente el proceso de elaboración del plato:

Pelar y cortar todas las verduras.
Poner al fuego 15 minutos.
Cuando hierva, añadir el pollo y hervir 5 ó 7 minutos más.
Triturar todo con un poco del agua de la cocción.
Añadir aceite de oliva cuando está todo triturado en el plato.

Fotos:

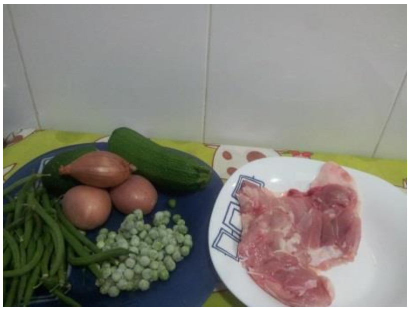
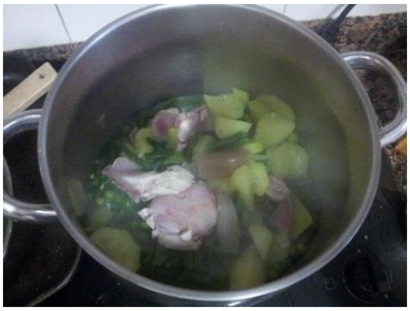
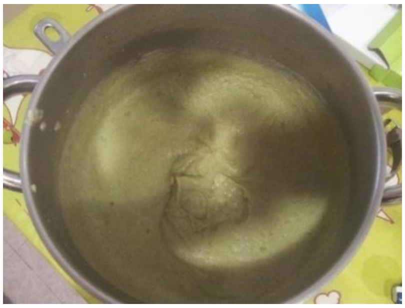

FICHA INGREDIENTES/PESOS "PLATO HECHO EN CASA (HC)" **ID: VAL 07**

DÍA DEL CONSUMO: *14 / 10 / 2014* HORA DEL CONSUMO: *20:14*

LA ELABOARACIÓN DEL PLATO SE HACE EL MISMO DÍA DEL CONSUMO [X] Sí [ ] No

Si la fecha de elaboración del plato NO es el mismo día del consumo NO se adjuntará foto y el peso de los ingredientes puede ser aproximado. Indique la fecha de elaboración:

TIPO DE PLATO: *PURÉ DE VERDURAS CON MERLUZA*

	Tipo de ingrediente	Nº	Peso (en gramos)
Ingrediente 1	*Zanahoria*	*2*	*155 gr*
Ingrediente 2	*Calabacín*	*½*	*358 gr*
Ingrediente 3	*Patata*	*2*	*228 gr*
Ingrediente 4	*Merluza*	*2*	*250 gr*
Ingrediente 5	*Tomate*	*Medio*	*100 gr*
Ingrediente 6	*Brocoli*		*30 gr*
Ingrediente 7			
Ingrediente 8			
Ingrediente 9			
Ingrediente 10			

Proceso culinario	Nº	Peso (en gramos)	Observaciones
1. Olla VACÍA		*1202 gr*	
2. Olla con COMIDA COCINADA		*2042 gr*	
**COMIDA COCINADA (2-1)**		*840 gr*	
3. Fiambrera VACÍA			
4. Fiambrera con COMIDA COCINADA			
**COMIDA ALMACENADA (4-3)**			
5. Plato VACÍO		*404 gr*	
6. Plato con COMIDA COCINADA		*668 gr*	
**COMIDA QUE SIRVE A SU HIJO (6-5)**		*264 gr*	
7. Plato COMIDA QUE SU HIJO NO HA COMIDO		*404 gr*	
**COMIDA QUE SU HIJO HA COMIDO (6-7)**		*264 gr*	

MÉTODO DE ELABORACIÓN:
Explicar detalladamente el proceso de elaboración del plato

FICHA INGREDIENTES/PESOS “PLATO HECHO EN CASA (HC)”

ID: VAL 07

DÍA DEL CONSUMO: 14 / 10 / 2014 **HORA DEL CONSUMO:** 20:14

MÉTODO DE ELABORACIÓN:

Explicar detalladamente el proceso de elaboración del plato:

Pelar y cortar las verduras.
Poner al fuego junto con la merluza 20 minutos.
Triturar con un poco de agua de la cocción.

Fotos:

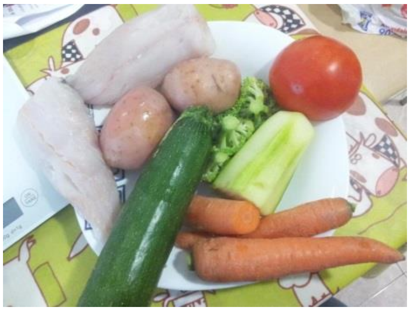

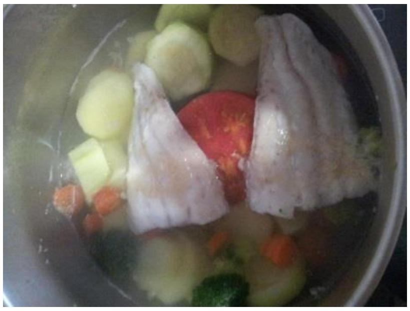

FICHA INGREDIENTES/PESOS "PLATO HECHO EN CASA (HC)" **ID: VAL 07**

DÍA DEL CONSUMO: *16 / 10 / 2014* HORA DEL CONSUMO: *11:45*

LA ELABOARACIÓN DEL PLATO SE HACE EL MISMO DÍA DEL CONSUMO ☒ Sí ☐ No

Si la fecha de elaboración del plato NO es el mismo día del consumo NO se adjuntará foto y el peso de los ingredientes puede ser aproximado. Indique la fecha de elaboración:

TIPO DE PLATO: *PURÉ DE CALABACÍN CON TERNERA Y VERDURAS*

	Tipo de ingrediente	Nº	Peso (en gramos)
Ingrediente 1	*Calabacín*	*2*	*541 gr*
Ingrediente 2	*Cebolla*	*1*	*150 gr*
Ingrediente 3	*Patata*	*2*	*240 gr*
Ingrediente 4	*Ternera*	*2*	*200 gr*
Ingrediente 5	*Zanahoria*	*1*	*72 gr*
Ingrediente 6			
Ingrediente 7			
Ingrediente 8			
Ingrediente 9			
Ingrediente 10			

Proceso culinario	Nº	Peso (en gramos)	Observaciones
1. Olla VACÍA		*1202 gr*	
2. Olla con COMIDA COCINADA		*2707 gr*	
**COMIDA COCINADA (2-1)**		*1505 gr*	
3. Fiambrera VACÍA			
4. Fiambrera con COMIDA COCINADA			
**COMIDA ALMACENADA (4-3)**			
5. Plato VACÍO		*418 gr*	
6. Plato con COMIDA COCINADA		*767 gr*	
**COMIDA QUE SIRVE A SU HIJO (6-5)**		*349 gr*	
7. Plato COMIDA QUE SU HIJO NO HA COMIDO		*518 gr*	
**COMIDA QUE SU HIJO HA COMIDO (6-7)**		*249 gr*	

MÉTODO DE ELABORACIÓN:
Explicar detalladamente el proceso de elaboración del plato

FICHA INGREDIENTES/PESOS "PLATO HECHO EN CASA (HC)"

ID: VAL 07

DÍA DEL CONSUMO: 16 / 10 / 2014 **HORA DEL CONSUMO:** 11:45

MÉTODO DE ELABORACIÓN:

Explicar detalladamente el proceso de elaboración del plato:

Pelar, cortar y lavar las verduras, poner a hervir.
Quitar la grasa de la ternera, trocear y añadir cuando hierva la verdura.
Después de 20 minutos, triturar con el caldo.

Fotos:

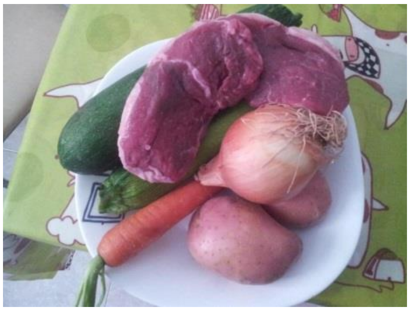
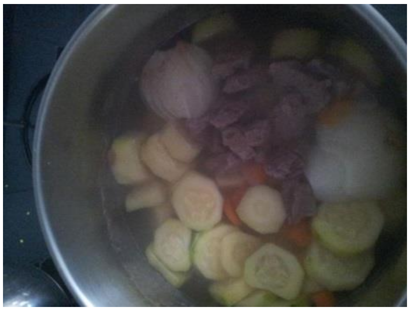
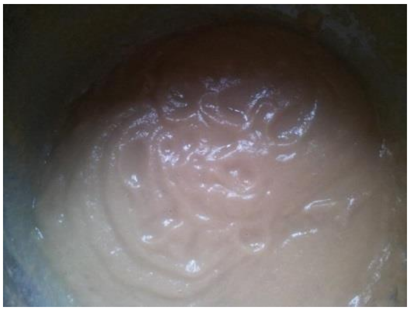

FICHA INGREDIENTES/PESOS "PLATO HECHO EN CASA (HC)" **ID: VAL 07**

DÍA DEL CONSUMO: *16 / 10 / 2014* HORA DEL CONSUMO: *20:30*

LA ELABOARACIÓN DEL PLATO SE HACE EL MISMO DÍA DEL CONSUMO [X] Sí [ ] No

Si la fecha de elaboración del plato NO es el mismo día del consumo NO se adjuntará foto y el peso de los ingredientes puede ser aproximado. Indique la fecha de elaboración: 16-10-14

TIPO DE PLATO: *PURÉ DE VERDURAS CON PESCADO*

	Tipo de ingrediente	Nº	Peso (en gramos)
Ingrediente 1	*Calabacines*	*3*	*800 gr*
Ingrediente 2	*Cebolla*	*1*	*153 gr*
Ingrediente 3	*Patatas*	*2*	*190 gr*
Ingrediente 4	*Rape*	*1 trozo*	*210 gr*
Ingrediente 5			
Ingrediente 6			
Ingrediente 7			
Ingrediente 8			
Ingrediente 9			
Ingrediente 10			

Proceso culinario	Nº	Peso (en gramos)	Observaciones
1. Olla VACÍA		*1202 gr*	
2. Olla con COMIDA COCINADA		*2600 gr*	
**COMIDA COCINADA (2-1)**		*1398 gr*	
3. Fiambrera VACÍA			
4. Fiambrera con COMIDA COCINADA			
**COMIDA ALMACENADA (4-3)**			
5. Plato VACÍO		*404 gr*	
6. Plato con COMIDA COCINADA		*724 gr*	
**COMIDA QUE SIRVE A SU HIJO (6-5)**		*320 gr*	
7. Plato COMIDA QUE SU HIJO NO HA COMIDO		*424 gr*	
**COMIDA QUE SU HIJO HA COMIDO (6-7)**		*300 gr*	

MÉTODO DE ELABORACIÓN:
Explicar detalladamente el proceso de elaboración del plato

FICHA INGREDIENTES/PESOS "PLATO HECHO EN CASA (HC)"

ID: VAL 07

DÍA DEL CONSUMO: 16 / 10 / 2014 **HORA DEL CONSUMO:** 20:30

MÉTODO DE ELABORACIÓN:

Explicar detalladamente el proceso de elaboración del plato:

Pelamos y cortamos la verdura y la ponemos a hervir 15 minutos.
Transcurrido el tiempo, añadimos el rape.
Trituramos todo con un poco de caldo.
Añadimos un poco de aceite de oliva.

Fotos:

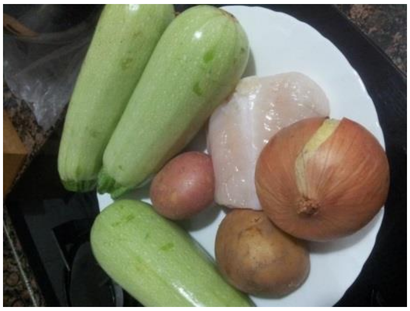

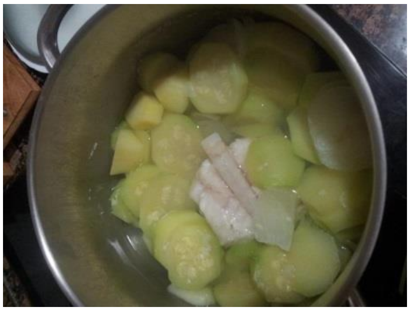

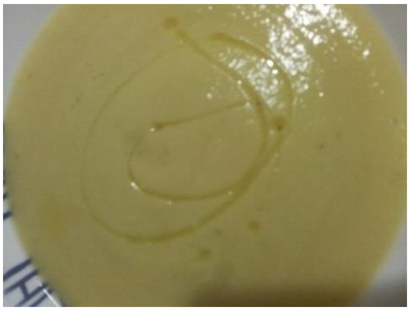

FICHA INGREDIENTES/PESOS "PLATO HECHO EN CASA (HC)" **ID: VAL 07**

DÍA DEL CONSUMO: *18 / 10 / 2014* HORA DEL CONSUMO: *11:45*

LA ELABOARACIÓN DEL PLATO SE HACE EL MISMO DÍA DEL CONSUMO [X] Sí [ ] No

Si la fecha de elaboración del plato NO es el mismo día del consumo NO se adjuntará foto y el peso de los ingredientes puede ser aproximado. Indique la fecha de elaboración:

TIPO DE PLATO: *PURÉ DE VERDURAS CON CORDERO*

	Tipo de ingrediente	Nº	Peso (en gramos)
Ingrediente 1	*Zanahoria*	*1*	*69 gr*
Ingrediente 2	*Guisantes*		*69 gr*
Ingrediente 3	*Judía baby*		*73 gr*
Ingrediente 4	*Calabacines*	*1/8*	*417 gr*
Ingrediente 5	*Patata*	*2*	*268 gr*
Ingrediente 6	*Cebolla*	*½*	*142 gr*
Ingrediente 7	*Cordero*	*2*	*200 gr*
Ingrediente 8			
Ingrediente 9			
Ingrediente 10			

Proceso culinario	Nº	Peso (en gramos)	Observaciones
1. Olla VACÍA		*1202 gr*	
2. Olla con COMIDA COCINADA		*2740 gr*	
**COMIDA COCINADA (2-1)**		*1538 gr*	
3. Fiambrera VACÍA			
4. Fiambrera con COMIDA COCINADA			
**COMIDA ALMACENADA (4-3)**			
5. Plato VACÍO		*404 gr*	
6. Plato con COMIDA COCINADA		*730 gr*	
**COMIDA QUE SIRVE A SU HIJO (6-5)**		*326 gr*	
7. Plato COMIDA QUE SU HIJO NO HA COMIDO		*439 gr*	
**COMIDA QUE SU HIJO HA COMIDO (6-7)**		*291 gr*	

MÉTODO DE ELABORACIÓN:
Explicar detalladamente el proceso de elaboración del plato

FICHA INGREDIENTES/PESOS "PLATO HECHO EN CASA (HC)"

ID: VAL 07

DÍA DEL CONSUMO: 18 / 10 / 2014 **HORA DEL CONSUMO:** 11:45

MÉTODO DE ELABORACIÓN:

Explicar detalladamente el proceso de elaboración del plato:

Pelamos, lavamos y ponemos a hervir.
Cuando pasan 15 minutos añadimos el cordero.
Transcurrido el tiempo, quitamos el hueso y la grasa.
Trituramos con el caldo.

Fotos:

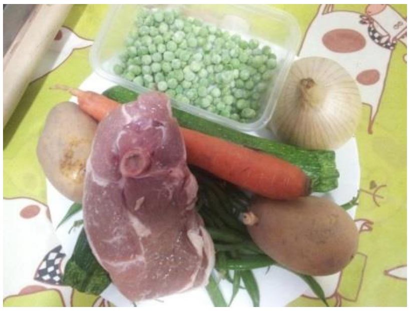

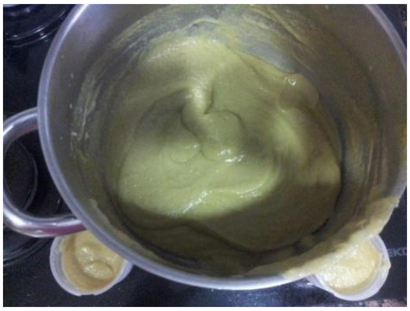

FICHA INGREDIENTES/PESOS "PLATO HECHO EN CASA (HC)" **ID: VAL 07**

DÍA DEL CONSUMO: *18 / 10 / 2014* **HORA DEL CONSUMO:** *16:35*

LA ELABOARACIÓN DEL PLATO SE HACE EL MISMO DÍA DEL CONSUMO [X] Sí [ ] No

Si la fecha de elaboración del plato NO es el mismo día del consumo NO se adjuntará foto y el peso de los ingredientes puede ser aproximado. Indique la fecha de elaboración: 18-10-14

TIPO DE PLATO: *PAPILLA DE FRUTAS*

	Tipo de ingrediente	Nº	Peso (en gramos)
Ingrediente 1	*Manzana*	*2*	*325 gr*
Ingrediente 2	*Plátanos*	*2*	*240 gr*
Ingrediente 3	*Peras*	*2*	*320 gr*
Ingrediente 4			
Ingrediente 5			
Ingrediente 6			
Ingrediente 7			
Ingrediente 8			
Ingrediente 9			
Ingrediente 10			

Proceso culinario	Nº	Peso (en gramos)	Observaciones
1. Olla VACÍA (donde trituro la fruta)		*185 gr*	
2. Olla con COMIDA COCINADA		*523 gr*	
**COMIDA COCINADA (2-1)**		*338 gr*	
3. Fiambrera VACÍA			
4. Fiambrera con COMIDA COCINADA			
**COMIDA ALMACENADA (4-3)**			
5. Plato VACÍO		*404 gr*	
6. Plato con COMIDA COCINADA		*573 gr*	
**COMIDA QUE SIRVE A SU HIJO (6-5)**		*169 gr*	
7. Plato COMIDA QUE SU HIJO NO HA COMIDO		*404 gr*	
**COMIDA QUE SU HIJO HA COMIDO (6-7)**		*169 gr*	

MÉTODO DE ELABORACIÓN:
Explicar detalladamente el proceso de elaboración del plato

FICHA INGREDIENTES/PESOS "PLATO HECHO EN CASA (HC)"

ID: VAL 07

DÍA DEL CONSUMO: 18 / 10 / 2014 **HORA DEL CONSUMO:** 16:35

MÉTODO DE ELABORACIÓN:

Explicar detalladamente el proceso de elaboración del plato:

Pelamos la fruta y la trituramos.

Fotos:

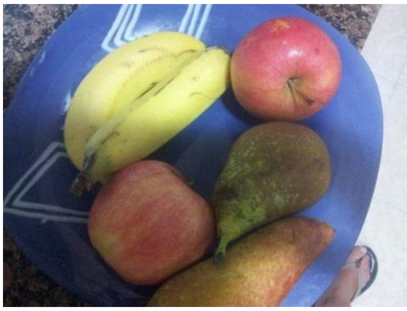

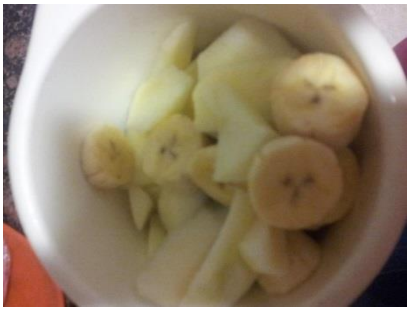

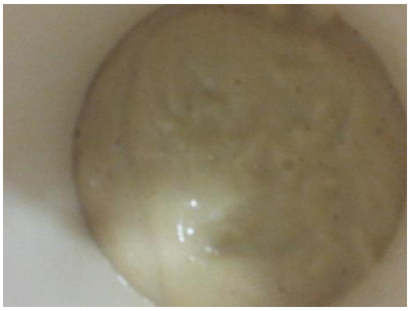

Supplement: Supplementary file 1 [file nutrients-13-00777-s001.pdf]
